# Far-Red Light-Mediated Seedling Development in *Arabidopsis* Involves FAR-RED INSENSITIVE 219/JASMONATE RESISTANT 1-Dependent and -Independent Pathways

**DOI:** 10.1371/journal.pone.0132723

**Published:** 2015-07-15

**Authors:** Huai-Ju Chen, Cheng-Ling Chen, Hsu-Liang Hsieh

**Affiliations:** Institute of Plant Biology, College of Life Science, National Taiwan University, Taipei, 106, Taiwan; National Taiwan University, TAIWAN

## Abstract

Plant growth and development is often regulated by the interaction of environmental factors such as light and various phytohormones. *Arabidopsis* FAR-RED INSENSITIVE 219 (FIN219)/JASMONATE RESISTANT 1 (JAR1) participates in phytochrome A-mediated far-red (FR) light signaling and interacts with different light signaling regulators. FIN219/JAR1 is a jasmonic acid (JA)-conjugating enzyme responsible for the formation of JA-isoleucine. However, how FIN219/JAR1 integrates FR light and JA signaling remains largely unknown. We used a microarray approach to dissect the effect of *fin219* mutation on the interaction of FR light and JA signaling. The *fin219-2* mutant was less sensitive than the wild type to various concentrations of methyl jasmonate (MeJA) under low and high FR light. High FR light reduced the sensitivity of *Arabidopsis* seedlings to MeJA likely through FIN219. Intriguingly, in response to MeJA, FIN219 levels showed a negative feedback regulation. Further microarray assay revealed that FR light could regulate gene expression by FIN219-dependent or -independent pathways. The expression profiles affected in *fin219-2* indicated that FIN219/JAR1 plays a critical role in the integration of multiple hormone-related signaling. In particular, FIN219 regulates a number of transcription factors (TFs), including 94 basic helix-loop-helix (bHLH) TFs, in response to FR light and MeJA. Loss-of-function mutants of some bHLH TFs affected by FIN219 showed altered responses to MeJA in the regulation of hypocotyl and root elongation. Thus, FIN219/JAR1 is tightly regulated in response to exogenous MeJA. It also interacts with multiple plant hormones to modulate hypocotyl and root elongation of *Arabidopsis* seedlings likely by regulating a group of TFs.

## Introduction

Light affects plant growth and development dramatically. In particular, seedling development is sensitive to the combined effects of light and various phytohormones, including auxin, gibberellins, cytokinin, abscisic acid (ABA) and jasmonic acid (JA). Studies of the interactions of light and these hormones have increased [1,2]. Understanding the molecular mechanisms underlying the interactions has progressed greatly, but the interaction of light and JA signaling has been little studied. Light and JA can interact to regulate different aspects of plant development, including seed germination, hypocotyl elongation, shade avoidance syndrome, development of floral organs and stomatal closure [3]. However, the molecular mechanisms responsible for these physiological responses remain to be elucidated.

FAR-RED INSENSITIVE 219 (FIN219) was isolated as an extragenic suppressor of CONSTITUTUVE PHOTOMORPHOGENIC 1 (COP1), and its mutant showed a hyposensitive long-hypocotyl phenotype under continuous far-red light (cFR). *FIN219* was cloned by a map-based method, encodes a GH3 protein, and can be induced rapidly by auxin in *Arabidopsis* [4]. FIN219 functions as a positive regulator in phytochrome A (phyA)-mediated FR signaling [4,5]. Moreover, JASMONATE-RESISTANT 1 (JAR1), a conjugating enzyme mainly responsible for the generation of JA-isoleucine (JA-Ile) in JA signaling, was later shown to have the same locus as FIN219 [6]. Thus, FIN219/JAR1 functions in a crosstalk between FR and JA signaling pathways in *Arabidopsis*.

FIN219/JAR1 can form a dimer to regulate hypocotyl elongation of *Arabidopsis* seedlings and physically interacts with COP1 in cytoplasm under FR light and dark conditions, for negative regulation of COP1 levels in FR light [7]. Further evidence revealed that greatly inducing FIN219 protein level could exclude COP1 from the nucleus to the cytoplasm even in the dark, thus leading to increased HY5 levels in the nucleus and a photomorphogenic phenotype [7]. Therefore, FIN219/JAR1 plays a vital role in the control of FR-mediated photomorphogenesis by modulating levels of the repressor COP1 and stability of the positive regulator HY5.

Recent studies revealed key components in the JA signaling pathway. CORONATINE INSENSITIVE 1 (COI1) is a major component of the receptor complex, including JA ZIM-domain proteins (JAZs), for perception of JA-Ile, a physiologically active form of JA [8–11]. COI1-perceived JA-Ile can enhance the interaction between COI1 and JAZ1, thus leading to JAZ1 degradation mediated by the ubiquitin/26S proteasome degradation pathway [9,12]. JAZ1 functions as a repressor of JA signaling to regulate the expression of downstream JA-responsive genes. In searching for positive transcription factors (TFs) with activities blocked by JAZs, MYC2 was found to interact with the C-terminal region of JAZ3 [9,12]. Ectopic expression of *MYC2* in the wild type resulted in constitutive expression of JA-responsive genes, including JAZ genes. Intriguingly, the *myc2* mutant also suppressed *JAZ* gene expression. JAZ3 and other JAZ proteins may mediate the JAZ—MYC2 interaction, thus inhibiting *MYC2* transcriptional activities. In addition to MYC2, other TFs participate in JA-mediated responses [13,14].

The *Arabidopsis* phytochrome chromophore mutants *hy1* and *hy2* show elevated levels of JA and constant activation of COI-dependent JA responses; moreover, JA inhibits the expression of a group of light-inducible photosynthetic genes [15]; thus, phytochrome chromophore-mediated light signaling and the JA signaling pathway may have a mutually antagonistic relationship. Recent studies revealed that FR irradiation could strongly reduce plant sensitivity to jasmonates. FR light also differentially regulated methyl jasmonate (MeJA)-induced gene expression [16]. Further evidence revealed that JA-Ile or coronatine-mediated JAZ1 degradation required functional phytochrome A (phyA), so phyA may play a vital role in the regulation of JA signaling; however, the molecular mechanism underlying this regulation remains unclear. Intriguingly, the interaction between FR/red light and JA signaling has been implicated in regulation of *Lotus japonicas* nodulation [17] and *Arabidopsis* resistance to the necrotrophic pathogen *Botrytis cinerea* [18]. The molecular mechanisms underlying the effects of JA biosynthesis and signaling on FR light signaling remain to be elucidated.

We used a *fin219*-null mutant, *fin219-2*, with or without MeJA treatment under FR light for microarray assay, to provide further insights into how FIN219/JAR1 as a JA-conjugating enzyme functions to modulate unique or common components downstream of both signalings. Our results provide evidence for the regulation of FIN219/JAR1 levels in a negative feedback mechanism in response to MeJA. We show that FIN219/JAR1 can modulate FR light signaling, largely in a JA-dependent and-independent manner, and regulate hypocotyl elongation and root development by controlling a group of basic helix-loop-helix (bHLH) TFs.

## Materials and Methods

### Plant materials and growth conditions

The wild type, *fin219-2* [7], and glucocorticoid-inducible FIN219 transgenic line (*pGR*:*FIN219*; *PGR219*) [7] were all of the *Arabidopsis thaliana* Columbia ecotype (Col-0). *PGR219* seedlings were harvested in GM plates with 1 μM dexamethasone (Dex) in order to induce *FIN219* expression [7]. T-DNA inserted mutants of some TFs [*cib1/bhlh63* (CS821043), *cib5/bhlh76* (CS815870), *bhlh27* (CS813379), *bhlh51* (51–1, SALK_084837; 51–2, SALK_084933), and *bhlh120* (CS821667)] affected by *fin219-2* were obtained from the Arabidopsis Biological Research Center (ABRC, Ohio State University, Columbus, OH). Seed sterilization and studies of the effects of MeJA on hypocotyl and root elongation were described previously [4,7].

### Protein extraction and protein gel blot analysis

Seedlings of wild-type and *PGR219* plants grown in continuous FR light (2 or 10 μmol m^-2^ s^-1^) for 3 days were harvested for protein extraction. Total proteins were extracted with extraction buffer (50 mM Tris-HCl, pH7.5, 150 mM NaCl, 10 mM MgCl_2_, 0.1% NP-40, 1 mM PMSF and 1X protease inhibitor) as described previously [4]. Total proteins, 70 μg, were loaded in each lane and separated on 10% SDS-PAGE and transferred to PVDF membrane (Millipore). Protein gel blot analyses involved standard methods [19] and expression was detected with FIN219 monoclonal and JAZ1 polyclonal antibodies.

### Microarray analysis

Total RNA (1 μg) was isolated from wild-type Col, *fin219-2* and *PGR219* seedlings treated with 0 and 50 μM MeJA. Wild-type and *fin219-2* (or *PGR219*) RNA samples were labeled with Cy3 and Cy5, respectively. Hybridization involved the Agilent *Arabidopsis* Oligo 4×44K V4 Microarray (Agilent Technologies, Palo Alto, CA, USA) containing about 44,000 oligonucleotides. We used GeneSpring GX Software to analyze microarray data. Cy5/Cy3 represents the ratio of Cy5 to Cy3 intensity; with Cy5/Cy3 > 1, expression was considered upregulated in *fin219-2* (or *PGR219*) and with Cy5/Cy3 < 1, expression was considered downregulated. The genes with > 2-fold difference in expression between Col and *fin219-2* (or *PGR219*) (Cy5/Cy3 > 2 or < 0.5) were selected for further analysis of Gene Ontology (GO) terms. We obtained GO terms and annotation of *A*. *thaliana* from the GeneSpring GX Software database and compiled lists of genes with a well-known GO term. The GeneSpring GX Software determines the significance of over- or under-representation of each GO term by a binomial exact test (*p* < 0.05). The levels of some genes from microarray data were further validated by quantitative real-time PCR analyses with 3 biological replicates.

### RNA extraction, cDNA synthesis and quantitative real-time PCR (qPCR)

Three-day-old FR-grown seedlings, 100 mg, were ground in 0.5 ml Buffer A (1 M Tris-HCl, pH7.3, 5 mM EDTA, pH 8.0, 1% SDS), then extracted twice with an equal volume of phenol and once with chloroform/isoamylalcohol (24:1). The supernatant was precipitated with LiCl (final working concentration 3 M) and incubated at -20°C overnight. After centrifugation, the pellet was dissolved completely in 0.5 ml 2% potassium acetate, then precipitated again with isopropanol. Total RNA (2 μg) was treated with DNase to prevent genomic DNA contamination, then used as a template for cDNA synthesis with the ABI cDNA transcription kit (#4368814). Real-time PCR reactions involved a Bio-Rad Real-Time PCR system (Bio-Rad). Gene-specific primers (S2 Table) were used for analyzing mRNA levels of UBQ10 (internal control), *bHLH18* (At2G22750), *bHLH27* (At4G29930), *bHLH32* (At3G25710), *bHLH51* (At2G40200), *bHLH63* (*CIB1*), *bHLH76* (*CIB5*), *bHLH85* (At4G33880), *bHLH86* (At5G37800), and *bHLH120* (At5G51790) by qPCR. qPCR reactions were carried out at 95°C for 3 min; 40 cycles of 95°C for 3 sec; annealing temperature for 20 sec; and 72°C for 20 sec.

### Generation of JAZ1 polyclonal antibody

A cDNA fragment corresponding to the coding region of *JAZ1* was amplified by PCR with primers containing the built-in BamHI site and cloned into the pRSET-A vector in-frame with an upstream His-tag. The His-JAZ1 pRSET-A recombinant vector was introduced into the BL21 strain of *Escherichia coli*. The recombinant His-JAZ1 fusion protein was purified by 12% SDS-PAGE and electro-eluted by use of Electro-Eluter (Model 422, BIO-RAD). The eluted His-JAZ1 recombinant proteins were sent to LTK BioLaboratories for producing polyclonal antibodies.

## Results

### High FR light reduces sensitivity of *Arabidopsis* seedlings to MeJA likely via FIN219

Recent studies have revealed interactions between FR light and JA signaling in plants [20], but the molecular mechanisms underlying these interactions remain largely unknown. We examined phenotypic responses of *Arabidopsis* seedlings treated with various concentrations of MeJA under low-fluence FR light (LFR, 2 μmol m^-2^ s^-1^) or high-fluence FR light (HFR, 10 μmol m^-2^ s^-1^). *Arabidopsis* wild-type Columbia (Col) seedlings showed decreased hypocotyl length under LFR light in response to different concentrations of MeJA (S1 Fig). Hypocotyl responses to various concentrations of MeJA were similar under HFR and LFR light. Moreover, MeJA at 50 μM minimum concentration greatly reduced hypocotyl length under LFR and HFR light (S1A Fig). As well, *fin219-2* mutant and *PGR219*, a glucocorticoid-inducible *FIN219* transgenic line [7], showed phenotypic responses to various concentrations of MeJA under LFR and HFR light similar to those in Col (S1A Fig). However, under white light, Col and *fin219-2* exhibited comparable hypocotyl responses to different concentrations of MeJA, and *PGR219* showed a constitutively shorter hypocotyl phenotype compared to both Col and *fin219-2* under all concentrations of MeJA examined (S1B Fig).

**Fig 1:**
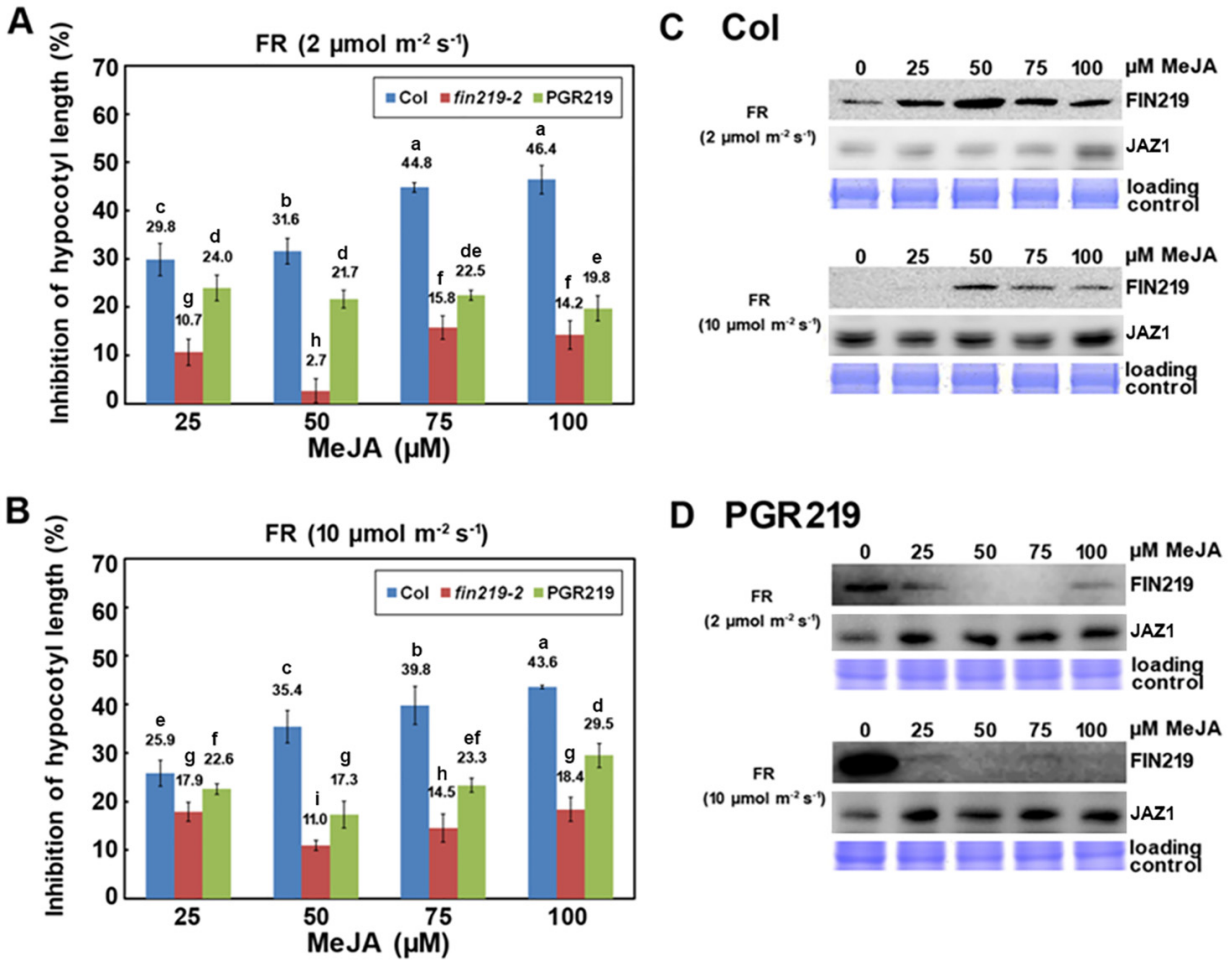
High far-red (FR) light irradiation reduces sensitivity of *Arabidopsis* seedlings to methyl jasmonate (MeJA) and a negative feedback regulation of FIN219 levels in response to MeJA. **A**. Hypocotyl response of wild-type Col, *fin219-2*, and *FIN219* inducible transgenic line (*PGR219*) to different concentrations of MeJA under low FR light. Seedlings were grown under low FR light (2 μmol m^-2^ s^-1^) for 3 days with 0, 25, 50, 75 and 100 μM MeJA. The hypocotyl length in response to MeJA was converted to inhibition percentage of that without MeJA. n = 35. Different lowercase letters represent significant differences by Tukey’s studentized range test at *P*< 0.05. **B**. Hypocotyl response of wild-type Col, *fin219-2*, and *PGR219* to different concentrations of MeJA under high FR light. Treatment was as in (A) except for high FR light (10 μmol m^-2^ s^-1^). Data are mean±SEM. n = 35. **C**. Gel blot analysis of FIN219 protein level in wild-type Col in response to various concentrations of MeJA under low and high FR light. Seedlings were grown under low and high FR light with different concentrations of MeJA for 3 days. Total protein, 70 μg, was loaded in each lane and probed with FIN219 monoclonal and JAZ1 polyclonal antibodies. Coomassie blue staining was a loading control. **D**. Gel blot analysis of FIN219 protein level in *PGR219* in response to various concentrations of MeJA under low and high FR light conditions. Seedlings of the *PGR219* transgenic line were used for detecting FIN219 levels as shown in **(C)**.

To further understand the molecular interaction of FR light and JA signaling, we examined the effect of the *fin219* mutation on hypocotyl elongation of *Arabidopsis* seedlings in the presence of MeJA under different LFR and HFR. Col seedlings showed a more sensitive response, with greater inhibition of hypocotyl elongation at 25, 75 and 100 μM MeJA under LFR than HFR light (Fig 1A and 1B). In contrast, *fin219-2*, with a null allele, showed less sensitivity, with lower inhibition of hypocotyl elongation to 25 and 50 μM MeJA under LFR than HFR light (Fig 1A and 1B) and more inhibition at 75 and 100 μM than 50 μM MeJA under LFR. In addition, *fin219-2* showed 4 times more inhibition of hypocotyl elongation at 50 μM MeJA under HFR than LFR light and greater inhibition of hypocotyl elongation at 75 and 100 μM than 50 μM MeJA under HFR light (Fig 1B). Thus, another component may respond to high concentrations of MeJA-mediated inhibition of hypocotyl elongation under HFR light.

Since *PGR219* exhibited a hypersensitive short-hypocotyl phenotype under FR light and dark conditions with strong expression of FIN219 induced by dexamethasone (Dex) [7], its hypocotyls were shorter than Col seedlings in the absence of MeJA under both LFR and HFR light (S1A and S1B Fig), and inhibition of hypocotyl elongation with different concentrations of MeJA under both LFR and HFR light was lower in *PGR219* than Col seedlings (Fig 1A and 1B). However, *PGR219* appeared to be less sensitive to MeJA at < 50 μM and more sensitive to MeJA at high concentrations such as 100 μM under HFR than LFR light (Fig 1A and 1B). This discrepancy of phenotype responses may be reflected by FIN219 levels being differentially regulated by various fluence rates of FR light [7] and possibly a negative feedback regulation at high concentrations of MeJA.

### FIN219 levels in response to MeJA show a negative feedback regulation

We further examined FIN219 levels in Col and *PGR219* by using FIN219 monoclonal antibodies (S2 Fig) under the previously described conditions [7]. Surprisingly, under LFR light, in Col seedlings, FIN219 levels peaked at 50 μM MeJA and then decreased to a certain level at 75 and 100 μM MeJA (Fig 1C); in contrast, under HFR light, in Col seedlings, FIN219 levels were barely detected at 25 μM MeJA and then peaked at a lower level than at LFR with 50 μM MeJA, followed by a reduced level at higher concentrations of MeJA (Fig 1C). To substantiate the regulated pattern of FIN219 levels by MeJA under LFR and HFR conditions, we examined the levels of JAZ1 by the use of JAZ1 polyclonal antibodies with detection specificity against JAZ1 (S3 Fig). It appeared that JAZ1 in Col was slightly increased along with increasing MeJA under LFR and comparable with different concentrations of MeJA under HFR (Fig 1C). Furthermore, in the absence of MeJA, FIN219 levels in *PGR219* were highly induced by Dex under HFR as compared with LFR, and with the addition of different concentrations of MeJA, especially 50 μM MeJA, were reduced to very low levels (Fig 1D), which suggests that FIN219 expression at higher levels is subject to a negative feedback regulation on exposure to exogenous MeJA. Likewise, under LFR, FIN219 levels in *PGR219* showed a similar trend, with a great reduction in level at 50 and 75 μM MeJA (Fig 1D). By contrast, JAZ1 levels in *PGR219* were significantly induced by MeJA and stayed unchanged over various concentrations of MeJA under LFR and HFR (Fig 1D). Thus, FIN219 levels are tightly regulated with a negative feedback loop in response to JA and FR light.

### Microarray analysis reveals that FR light can regulate gene expression via FIN219–dependent and—independent pathways

FIN219/JAR1 is a JA-conjugating enzyme and can be induced by FR light and the JA hormone [4,6]; moreover, FR light and JA signaling can antagonize each other [15]. However, the molecular mechanism underlying the interaction of both signaling pathways is unclear. Thus, we performed microarray analysis with the wild type and *fin219-2* mutant to understand the effect of *fin219* mutation on the interaction of FR light and JA signaling. With microarray experiments and normalization of the data, most of the genes were labeled equally by fluorescence dyes, and their expression could be detected at 10^−2^ to 10^6^ intensity (S4 Fig). We compared the expression profiles of the wild type and *fin219-2* with or without 50 μM MeJA treatment under LFR.

**Fig 2:**
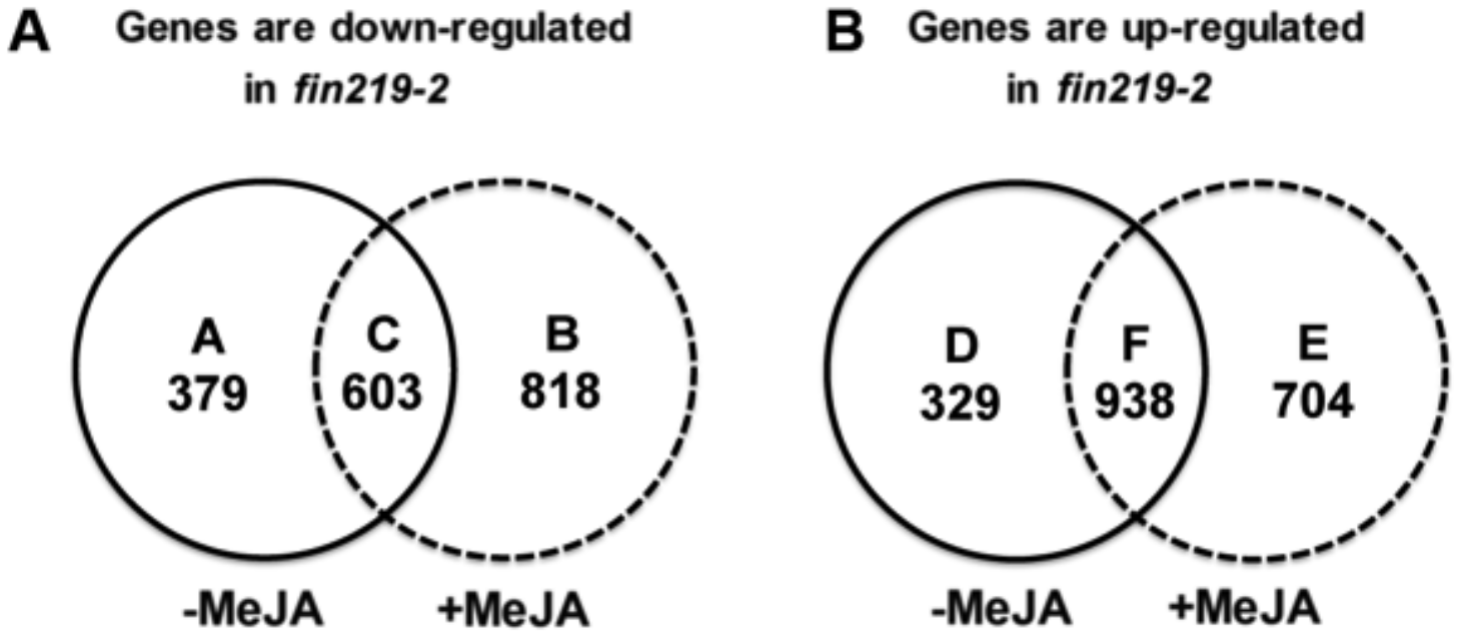
Microarray assay of genes with significant differences in transcript abundance between wild type and *fin219-2* in response to FR light with and without MeJA. Venn diagram of number of genes with significant difference (≥ 2-fold) in transcript abundance between *fin219-2* and the wild type without (-MeJA) or with 50 μM MeJA (+MeJA) under low FR light (2 μmol m^-2^ s^-1^).

Under LFR and no MeJA treatment, *fin219-2* showed differential expression of 2,249 genes, including 982 downregulated and 1,267 upregulated genes, classified into groups A, C, D, and F based on at least 2-fold difference in expression (Fig 2). Group A contained genes involved in sugar binding, lipid transport, binding and localization according to Gene Ontology (GO) analysis (S1 Table). Group D consisted of only one GO term, “response to water”, and included genes responding to cold and dehydration. The expression of group A and D genes was independent of the MeJA effect. Group C contained genes involved in catalytic activities, amino acid and derivative metabolic process, response to stress, defense response, response to UV, secondary metabolic process, glycosinolate biosynthetic and metabolic process as well as nitrogen, ketone, amine and sulfur metabolic processes (S1 Table). These genes were commonly affected by the absence or presence of MeJA under LFR in *fin219-2* (Fig 2A).

In contrast, in the presence of MeJA under LFR, the *fin219-2* mutant showed differential expression of 3,063 genes, including 1,421 downregulated and 1,642 upregulated genes, classified into groups B, C, E, and F (Fig 2). Group B contained a variety of GO terms and included genes involved in amino acid and derivative biosynthetic and metabolic processes; sulfur metabolic process; iron ion transport; response to stress, cold, external stimulus, wounding, abiotic stress, and endogenous stimulus; response to hormones such as ABA, salicylic acid (SA), and jasmonic acid (JA); phenylpropanoid biosynthetic and metabolic processes; glycoside biosynthetic and metabolic processes; glycosinolate biosynthetic and metabolic processes; plastid and chloroplast stroma; jasmonic acid/oxylipin biosynthetic and metabolic processes; oxygen and heme binding; and response to chemical stimulus (Fig 2 and S1 Table). In contrast, group E contained genes responsible for the regulation of TF activity and gene expression and modulation of macromolecular biosynthesis and metabolic processes with involvement of the JA effect (S1 Table). Group F contained genes involved in receptor activities and programmed cell death (S1 Table). Thus, FIN219 appeared to positively regulate lipid biosynthetic and metabolic processes and negatively modulate cold and dehydration responses in response to FR light independent of JA. However, in a JA-dependent manner, FIN219 positively regulates amino-acid biosynthesis and derivative metabolic processes, stress-related metabolism, and JA biosynthesis and metabolic processes and negatively regulates TF activities and macromolecular biosynthesis and metabolism.

### FIN219/JAR1 plays a vital role in the integration of multiple signaling pathways

**Fig 3:**
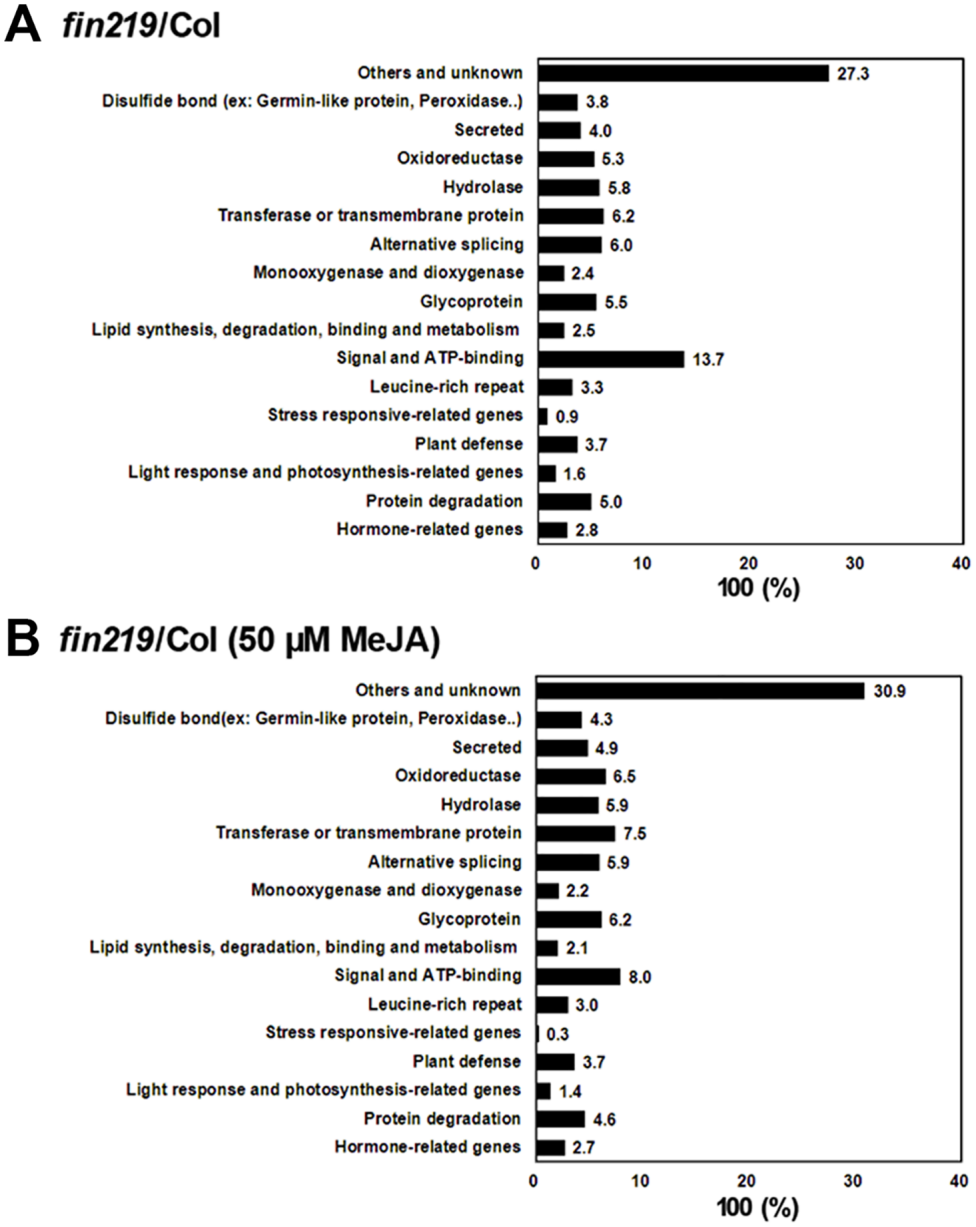
Relative ratio of Gene Ontology (GO) groups among gene expression profiles affected by *FIN219* levels under low FR light without or with MeJA treatment. Relative ratio of GO groups among gene expression profiles in *fin219-2* versus wild-type Col with at least 2-fold difference in expression under low FR light (2 μmol m^-2^ s^-1^) (A) and with 50 μM MeJA under low FR light (B). Relative ratio calculated as gene number in each GO group / total affected genes (2,249 in *fin219*/Col and 3,063 in *fin219*/Col with MeJA) * 100%.

To further confirm the role of FIN219 in integrating FR light and JA signaling, we analyzed the relative ratio of gene expression affected by *FIN219* loss-of-function (*fin219-2*) and gain-of-function (*PGR219*) with and without MeJA under LFR light (S4 and S5 Tables, S5 Fig). The expression profiles indicated that *FIN219* affected various groups of genes, especially signal and ATP-binding components, accounting for 14% of differentially regulated genes in *fin219*-2 (Fig 3A). Surprisingly, 5% and 6% of differentially regulated genes in *fin219*-2 were protein-degradation and alternative splicing—related genes, respectively (Fig 3A, S4 Table). In the presence of 50 μM MeJA, *fin219-2* showed a substantially reduced proportion of signal and ATP-binding components, by 6%. The proportion of light-response and photosynthesis-related genes was reduced by 1%; however, that of oxidoreductase and transferase/transmembrane protein genes was increased by 2% and 1%, respectively. Other groups of genes remained largely unchanged in proportion (Fig 3B, S5 Table). As well, with *FIN219* overexpression in *fin219-2* (*pGR219*) under LFR light, new groups of genes were induced, including transcriptional regulation—related (5%), metal-binding proteins (8%), and transit peptide—related (5%) genes (S5A Fig, S4 Table). The proportion of other genes, such as light-response and photosynthesis—related genes and transferase/transmembrane proteins was increased by 1% and 3%, respectively. However, that of protein degradation, plant defense, lipid-synthesis/degradation/binding/metabolism and glycoprotein genes was lower by 1% to 2% with *FIN219* overexpression than in *fin219-2* under LFR light (S5A Fig, S4 Table). Intriguingly, *PGR219* with 50 μM MeJA did not show further enhanced gene expression but rather showed decreased proportion of 2 groups of genes such as signal and ATP-binding and transferase/transmembrane protein—related genes. The proportion of other groups of genes remained largely the same in *fin219-2* as in *PGR219* without MeJA (S5B Fig, S5 Table). Thus, these data are consistent with Fig 1 showing that *FIN219* expression responding to JA may involve a negative feedback regulation.

Recently, the JA hormone was found to interact with other hormones to regulate plant development [21]. Here, we further found that FIN219/JAR1, as a JA-conjugating enzyme, showed crosstalk with other hormones. Its mutation (*fin219-2*) mainly affected auxin (0.67%), ABA (0.62%), ethylene (0.58%) and JA (0.36%) biosynthesis and signaling-related genes (Table 1, S6 Table); however, its overexpression (*PGR219*) substantially increased the number and proportion of auxin-biosynthesis and signaling-related genes, most downregulated by *PGR219* (51 of 73). Moreover, the number of ethylene biosynthesis and signaling-related genes was significantly increased. In addition, genes related to other hormones, including ABA and JA, were increased in number, although the proportion of genes affected by *PGR219* was largely decreased (Table 1, S7 Table). In contrast, with MeJA, *fin219-2* increased the proportion of auxin- and JA-related genes, with an increase in genes affected by *fin219-2*; however, the proportion of ABA-related genes was decreased and that of other hormone-related genes remained comparable to that in *fin219-2* without MeJA (Table 1, S6 Table). In addition, with MeJA under LFR, *PGR219* showed substantially increased proportion of auxin, ethylene, ABA and gibberellic acid (GA)-related genes. The proportion of genes related to other hormones, especially JA, remained largely unchanged, even with the addition of 50 μM MeJA as compared to without MeJA (Table 1, S7 Table). Thus, FIN219/JAR1, responsible for the formation of JA-Ile, may involve a negative feedback regulation of JA biosynthesis, thereby leading to JA signaling. FIN219/JAR1, as a JA-conjugating enzyme, may be a module integrating with multiple hormone signaling pathways in response to FR light to control seedling development.

**Table 1:** Number and percentage of the components involved in multiple hormone biosynthesis and signaling pathways changed in loss-of-function (*fin219-2*) or gain-of-function (*PGR219*) *FIN219/JAR1* in *Arabidopsis* seedlings with or without 50 μM methyl jasmonate (MeJA). Data are derived from microarray assay shown in Fig 3. up, upregulated; down, downregulated.

	*fin219-2*/Col								*PGR219*/Col							
	0 MeJA				50 MeJA				0 MeJA				50 MeJA			
	up	down	total	(%)	up	down	total	(%)	up	down	total	(%)	up	down	total	(%)
**ABA**	**10**	**4**	**14**	**0.62**	**6**	**3**	**9**	**0.29**	**12**	**8**	**20**	**0.26**	**19**	**15**	**34**	**0.47**
**Ethylene**	**10**	**3**	**13**	**0.58**	**9**	**8**	**17**	**0.56**	**28**	**11**	**39**	**0.51**	**35**	**12**	**47**	**0.64**
**GA**	**1**	**4**	**5**	**0.22**	**0**	**6**	**6**	**0.20**	**4**	**8**	**12**	**0.16**	**13**	**12**	**25**	**0.34**
**AUXIN**	**9**	**6**	**15**	**0.67**	**21**	**7**	**28**	**0.91**	**22**	**51**	**73**	**0.96**	**32**	**56**	**88**	**1.20**
**JA**	**2**	**6**	**8**	**0.36**	**1**	**14**	**14**	**0.46**	**10**	**7**	**17**	**0.22**	**13**	**3**	**16**	**0.22**
**SA**	**1**	**0**	**1**	**0.04**	**1**	**0**	**1**	**0.03**	**1**	**0**	**1**	**0.01**	**1**	**1**	**2**	**0.03**
**BR**	**1**	**3**	**4**	**0.18**	**4**	**1**	**5**	**0.16**	**7**	**3**	**10**	**0.13**	**7**	**5**	**12**	**0.16**
**Cytokinin**	**1**	**2**	**3**	**0.13**	**2**	**1**	**3**	**0.10**	**3**	**5**	**8**	**0.11**	**3**	**5**	**8**	**0.11**
**Hormone-related**	**35**	**28**	**63**	**2.80**	**43**	**40**	**83**	**2.71**	**87**	**93**	**180**	**2.38**	**123**	**109**	**232**	**3.17**
**total affected genes**	**1267**	**982**	**2249**		**1642**	**1421**	**3063**		**3967**	**3610**	**7577**		**3698**	**3613**	**7311**	

### FIN219 regulates a group of bHLH TFs in response to FR light and MeJA

**Fig 4:**
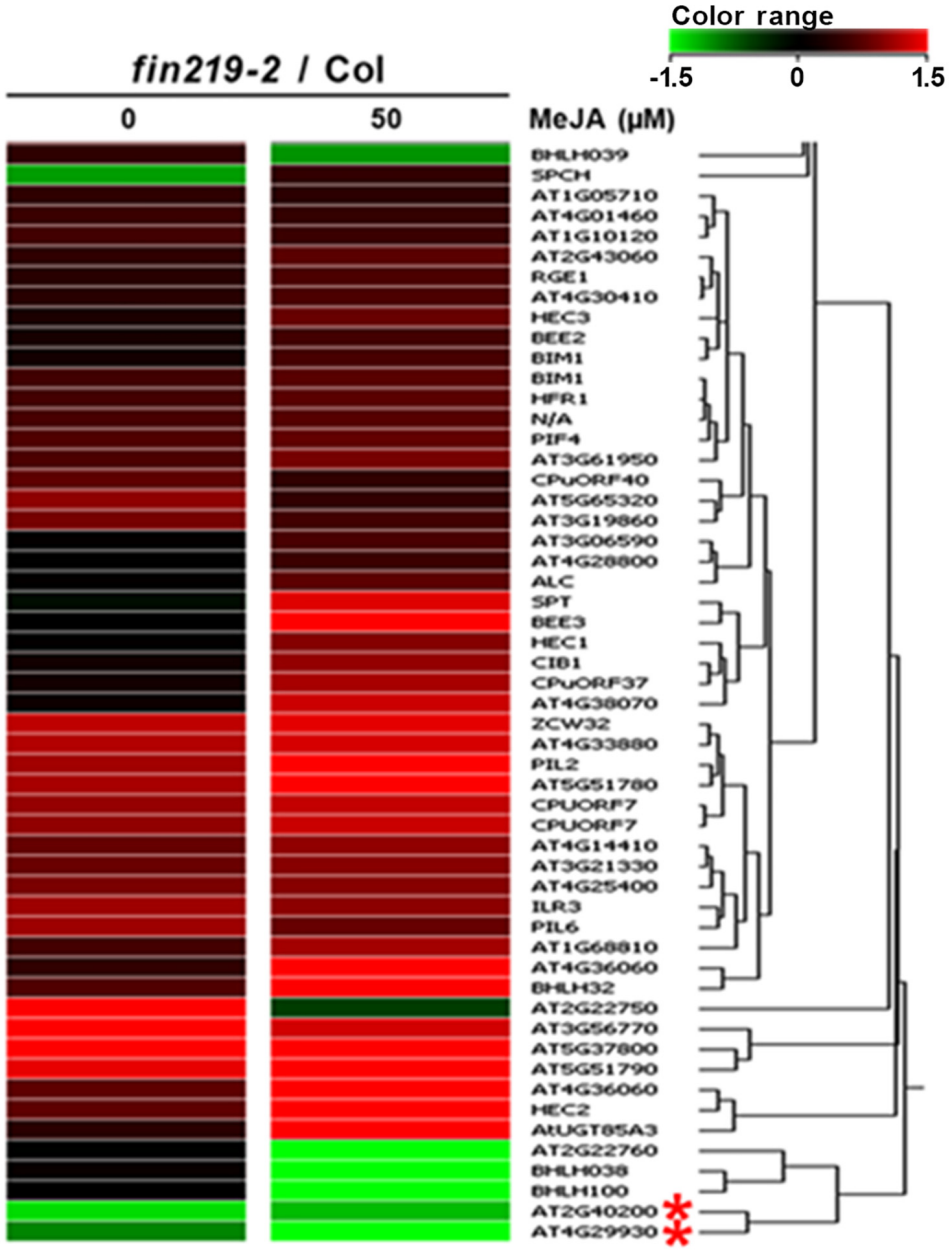
The *fin219-2* mutant shows altered expression of a number of transcription factors (TFs) under low FR light with or without MeJA. Hierarchical clustering analysis of the basic helix-loop-helix (bHLH) TFs in *fin219-2* under low FR light without or with 50 μM MeJA. Seedlings of wild type and *fin219-2* mutant were grown under low FR light without or with 50 μM MeJA for 3 days and subjected to microarray assay. Red asterisk marks the genes *At2g40200* (bHLH51) and *At4g29930* (bHLH27) that were downregulated in *fin219-2* under both low FR light and MeJA.

Expression profiling by microarray assay revealed that the *fin219* mutation with the interaction of FR light and JA signaling affected more than 200 TFs. Among them were 94 bHLH TFs with expression changes in *fin219-2* without MeJA or with MeJA treatment compared to Col (Fig 4 and S6 Fig). The bHLH TFs participate in regulating many physiological responses, including photomorphogenesis and JA signaling [14, 22]. FIN219 positively or negatively regulated a group of TFs independent of or depending on the MeJA effect under LFR light (Fig 4). MYC2, a bHLH TF involved in light- and JA-regulated defense responses and plant development [8,13,23,24, 25], was downregulated in *fin219-2* with and without MeJA under LFR (S6 Fig, red asterisk). Several other bHLH TFs such as At5g43175 (bHLH139), At2g40200 (bHLH51) and At4g29930 (bHLH27) were also downregulated in *fin219-2* (Fig 4 and S6 Fig, red asterisk) and likely serve as positive regulators in addition to MYC2 participating in JA-regulated responses. Intriguingly, *FIN219* negatively regulated *PIF4*, *PIL2* and *PIL6* transcript levels under both LFR and MeJA treatment (Fig 4). However, FIN219 only slightly affected *PIF3* and *PIF7* with LFR or MeJA treatment (S6 Fig). Furthermore, FIN219, especially with MeJA treatment, negatively regulated brassinosteroid (BR) signaling components such as *BEE3* and *BEE2* (Fig 4 and Table 1) and negatively modulated a gene family, including *HEC1*, *HEC2* and *HEC3* (Fig 4), involved in regulating development of the female reproductive tract [26].

**Fig 5:**
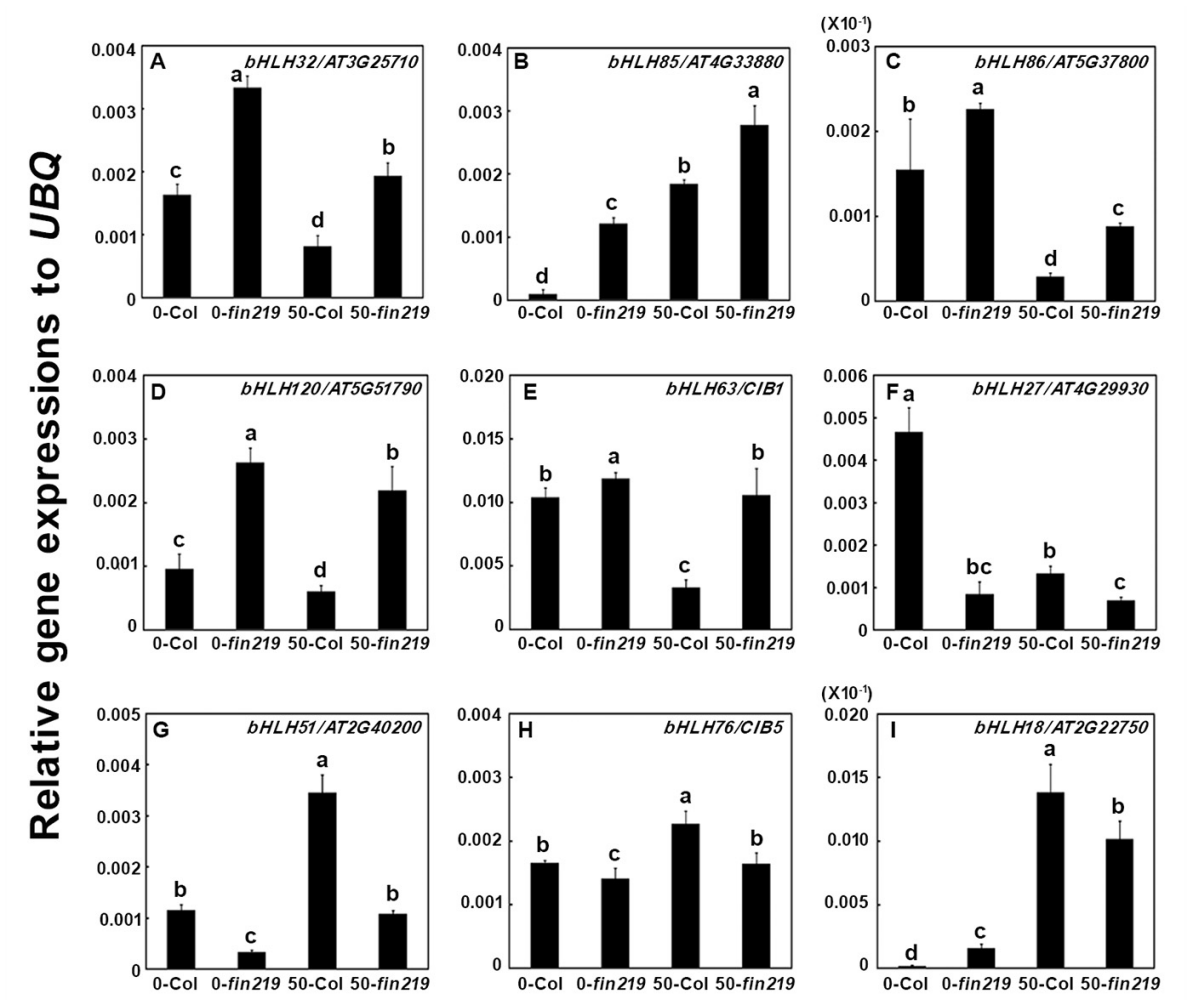
Validation of selected bHLH TFs in *fin219-2* mutant. **(A)**-**(I)** Quantitative real-time PCR analysis of bHLH TF expression in seedlings of wild type and *fin219-2* grown without or with 50 μM MeJA under low FR light for 3 days. Data are mean±SEM from 3 biological replicates. Different lowercase letters represent significant differences by Tukey’s studentized range test at *P*< 0.05.

To further validate the expression profiles of some TFs affected in *fin219-2*, we selected several TFs for further characterization by quantitative PCR (Fig 5 and S2 Table) according to their expression levels substantially affected by *fin219* mutation or known intriguing genes (S8 Table). The results were consistent with those from microarray assays. Some bHLH TFs genes, including *bHLH32/At3g25710*, *bHLH85/At4g33880*, *bHLH86/At5g37800*, *bHLH120/At5g51790* and *bHLH63/cryptochrome-interacting basic-helix-loop-helix1* (*CIB1*), were negatively regulated by *FIN219* under both LFR and MeJA conditions (Fig 5A–5E). Others, such as *bHLH27/At4g29930*, *bHLH51/At2g40200*, and *bHLH76/CIB5*, were positively regulated by *FIN219* under both conditions (Fig 5F–5H). One TF, *bHLH18/At2g22750*, was negatively regulated by *FIN219* under LFR but positively with MeJA treatment (Fig 5I). Thus, FIN219 may regulate diverse aspects of seedling development by modulating the expression of a group of bHLH TFs.

### Loss-of-function mutants of bHLH TFs regulated by FIN219/JAR1 show altered responses to MeJA

**Fig 6:**
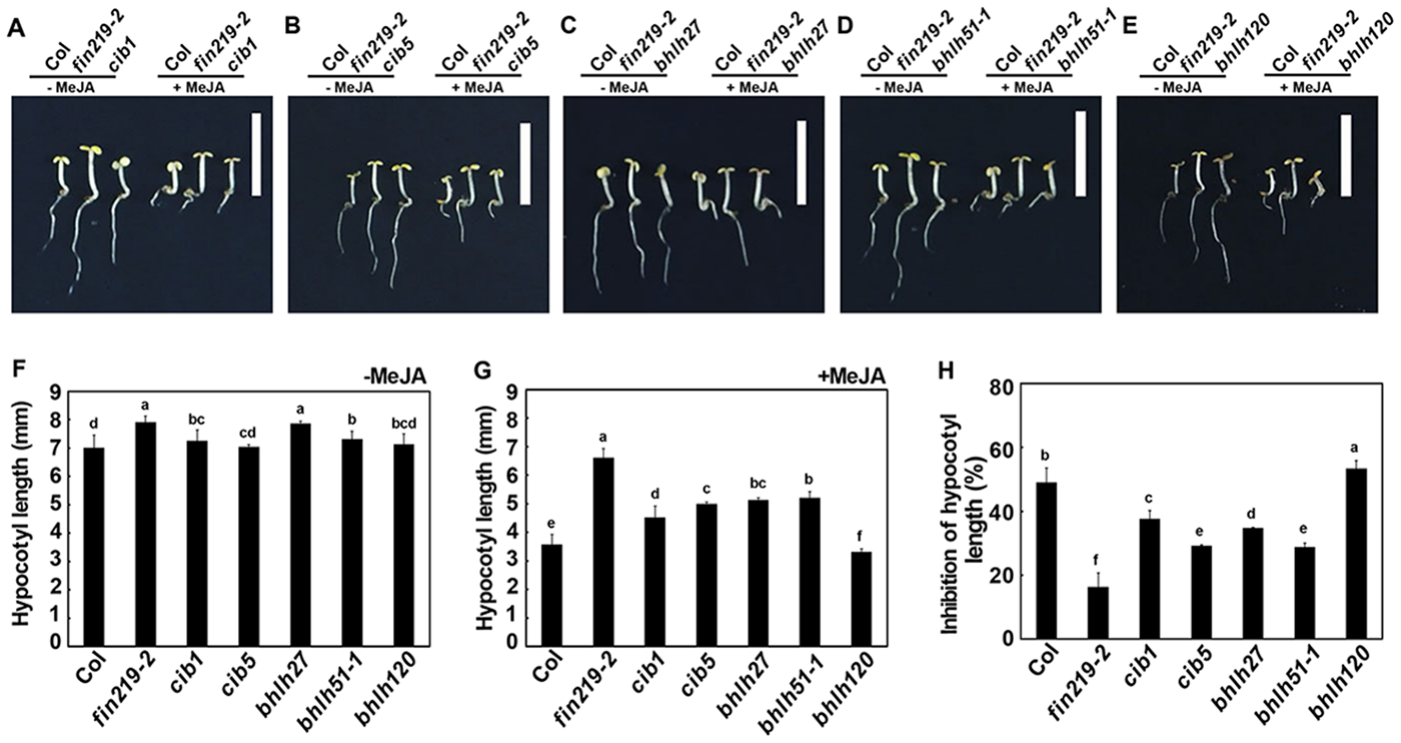
Loss-of-function mutants of selected bHLH TFs show altered hypocotyl responses to exogenous MeJA. **A-E**. Hypocotyl elongation of wild-type Col-0 (Col), *fin219-1*, *fin219-2* and selected bHLH TF mutants without or with MeJA under FR light. Seedlings were grown in low FR light (2 μmol m^-2^ s^-1^) without MeJA (-MeJA) or with 50 μM MeJA (+MeJA) for 3 days. Scale bar is 5 mm. F-G. Quantitative analysis of hypocotyl lengths of Col, *fin219-2* and selected bHLH TF mutants without MeJA (F) or with MeJA (G) under low FR light. Different lowercase letters represent significant differences by Tukey’s studentized range test at *P*< 0.05. **H**. Quantitative analysis of MeJA-mediated inhibition of hypocotyl elongation shown in (A-**E**). Data are mean±SEM from 3 biological replicates. Different lowercase letters represent significant differences by Tukey’s studentized range test at *P*< 0.05.

To further understand whether these bHLH TFs affected by *fin219-2* participate in JA signaling, we obtained some loss-of-function T-DNA insertion lines—*cib1*, *cib5*, *bhlh27*, *bhlh51*, and *bhlh120*—from the *Arabidopsis* Biological Resource Center. After confirmation of the T-DNA insertion in respective mutant lines (S8 Fig, S3 Table), homozygous transgenic lines of these TFs underwent phenotypic analyses in the presence or absence of MeJA. Both CIB1 and CIB5 can interact with cryptochrome 2 in a blue light—specific manner in *Arabidopsis* to regulate flowering [27]. Here, we found that *cib1* showed altered responses to MeJA under LFR, including less sensitivity of both hypocotyl and root elongation to exogenous MeJA (Fig 6A and 6F–6H, S7 Fig). In contrast, the *cib5* mutant showed reduced sensitivity of hypocotyls but not root elongation in response to MeJA as compared with the wild type (Fig 6B and 6F–6H, S7 Fig). Mutant *bhlh27* exhibited similar sensitivity of root elongation but significant insensitivity of hypocotyl elongation to exogenous MeJA as compared with the wild type (Fig 6C and 6F–6H, S7 Fig). Mutant *bhlh51* showed insensitivity of both root and hypocotyl elongation to MeJA (Fig 6D and 6F–6H, S7 Fig), and *bhlh120* showed comparable sensitivity of root elongation but even more sensitivity of hypocotyl elongation to MeJA as compared with the wild type (Fig 6E and 6F–6H, S7 Fig). Thus, FIN219/JAR1-regulated TFs may participate in a JA-mediated signaling pathway and have differential roles in regulating seedling development in response to JA.

## Discussion

Information on the interplay between FR light and jasmonate signaling is emerging. Previous studies indicated that the *Arabidopsis* chromophore mutants *hy1* and *hy2* showed increased levels of JA and constant activation of COI1-dependent JA responses; moreover, JA inhibited the expression of some light-inducible photosynthetic genes [15], which suggests that phytochrome chromophore-mediated light signaling and JA signaling may have an antagonistic effect with each other. Further study indicated that FR irradiation reduces plant sensitivity to JA [16], and recent studies revealed that PHYA regulated JA signaling by modulating JAZ1 stability in response to JA [20]. The *phyA* mutant showed reduced sensitivity of root elongation to 10 μM MeJA as compared with the wild type, with substantially decreased expression of JA-responsive genes such as *VSP1* and significantly increased OPDA levels in the dark and FR light [20]. Thus, the photoreceptor of FR light perception, phyA, appears to negatively modulate JA biosynthesis and positively regulate JA signaling to control seedling development. However, the molecular mechanism underlying the mutual integrated regulation of FR light and JA signaling remains unclear.

Our previous data showed that FIN219 levels positively regulated by phyA are greater under low FR light and greatly reduced under high FR light [7]. Here, our data indicate that high FR light irradiation reduced the sensitivity of wild-type seedlings to various concentrations of MeJA, except for 50 μM, as compared with low FR light (Fig 1A and 1B). This discrepancy may be reflected in low levels of endogenous FIN219 in high FR light under different concentrations of MeJA (Fig 1C). Intriguingly, FIN219 levels in wild-type Col peaked at 50 μM MeJA under both low and high FR light (Fig 1C). As well, high levels of FIN219 in *pGR219* were reduced to a very low level with the addition of MeJA (Fig 1D), which is consistent with less inhibition of hypocotyl length under low and high FR light (Fig 1A and 1B). Intriguingly, JAZ1 levels in Col and *PGR219* under LFR and HFR are stable in response to increasing MeJA concentrations (Fig 1C and 1D). Although JAZ1 functions as a repressor in JA signaling, JAZ1-GUS is stabilized in aerial parts and degraded in roots of *phyA-211* seedlings without hormone treatment [20], which suggests that JAZ1 stability is differentially regulated in aerial parts and roots of *Arabidopsis* seedlings, and light-dependent. It is worthy to notice that JAZ1-GUS is detected stronger in aerial parts than roots of *35S*:*JAZ1-GUS* seedlings [20]. Therefore, the molecular mechanisms underlying JAZ1 levels in Col and *fin219-2* or *PGR219* with or without MeJA treatment under light condition remain to be elucidated. In addition, FIN219/JAR1 is mainly responsible for the formation of JA-Ile, which accounts for about 90% of total JA-Ile in *Arabidopsis* seedlings under FR light [7]. Thus, the physiological responses FIN219/JAR1 mediates may involve a negative feedback regulation of JA-Ile. This finding differs from previous studies showing JA biosynthesis regulated by a positive feedback loop based on the transcriptional upregulation of *LOX*, *AOS*, *AOC* and *OPR3* on treatment with JA or biotic and abiotic stresses [28]. However, several concerns exist in the positive feedback regulation of JA biosynthesis. First, increased levels of the transcripts of these JA biosynthetic genes do not correspond to their protein levels and JA production. Second, although *AOS* mRNA, protein and activity are increased by JA treatment in *Arabidopsis* [29], OPDA is not correspondingly formed downstream [30]. A recent report revealed that 4 *AOC* genes in *Arabidopsis* showed tissue-specific expression [31]; *AOC1* and *AOC2* were more abundant in leaves, and the others were mainly expressed in roots. JA production may be highly tissue- or cell-type specific, for difficult detection in whole plants or seedlings. Alternatively, the levels of the physiologically active form of jasmonate, JA-Ile, which is converted by FIN219/JAR1, are tightly regulated in cells, as shown by the FIN219-overexpressing line *PGR219*, in a negative feedback manner (Fig 1D).

Our previous results suggested that FIN219/JAR1 might have a dual function, nonenzymatic and enzymatic activity, in modulating COP1 activity in response to FR light [7]. Furthermore, microarray assay revealed that FIN219/JAR1, independent of JA, regulated the lipid metabolic process positively and cold and dehydration responses negatively. In contrast, in a JA-dependent manner, FIN219/JAR1 positively regulated the biosynthesis or metabolic process of amino acids and their derivatives as well as stress-related metabolism and JA biosynthesis/metabolism but negatively modulated activities of some TFs and macromolecular biosynthesis and metabolism genes (Fig 2). We found that FIN219/JAR1 positively regulated the expression of multiple genes involved in fatty acid synthesis, including fatty acid desaturase (FAD8), elongase/acyltransferase (FAE1), very-long-chain fatty acid condensing enzyme (At1g25450) and fatty acid hydroxylase (FAH1), which may lead to the production of α-linolenic acid for generation of JA. FAD8 is localized in plastid and is temperature-sensitive and light-regulated [32]. Moreover, the light-regulated expression patterns of *FAD8* transcripts positively correspond to the level of trienoic fatty acid in cells [32]. The triple mutant *fad3-2fad7-2fad8* is deficient in α-linolenic acid, which results in undetected levels of JA, and shows extremely high mortality (almost 80%) caused by insect attack [33]. Moreover, photosynthetic functions in this triple mutant are more thermotolerant than the wild type in the short term at 33°C. In contrast, in the long term, at 33°C, photosynthetic functions are substantially reduced in the mutant [34], which suggests that the abundance of α-linolenic acid (18:3) may contribute to the photosynthetic function of *Arabidopsis* in the light. Very likely, FIN219/JAR1 induced by FR light can regulate the composition of the chloroplast membrane by increasing the levels of unsaturated fatty acids, especially α-linolenic acid, for sustaining photostability and photosynthetic function during earlier development of *Arabidopsis* seedlings on exposure to light.

Moreover, in a JA-dependent manner, FIN219/JAR1 positively regulated JA biosynthesis and signaling. In particular, it modulated the expression of genes encoding the first 3 enzymes, such as LOX2, LOX3, AOS, AOC2 and AOC4 in the JA biosynthetic pathway, likely leading to the production of JA. It also positively regulated the expression of JA-responsive genes, including coronatine-induced 3 (*CORI3*), *VSP1/2* and *JAZ*. Because *VSP* and *JAZ* genes are rapidly induced by JA [8,35], FIN219/JAR1, responsible for the production of JA-Ile, may regulate JA signaling, including the induction of the *JAZ* genes in the early stage of the signaling.

Although previous studies indicated that exogenous MeJA reduced the transcript levels of *HY1* and photosynthesis-related genes such as *RBCS* (At1g67090), *RCA* (At1g73110) and *CAB* (At2g40100) in 2-week-old wild-type seedlings grown under white light [15], our microarray data revealed that FIN219/JAR1, via a JA-dependent pathway, positively regulated different sets of photosynthesis-related genes, including *RBCS* (At5g38420), *RCA* (At2g39730), *CAB1* (At1g29930), *CAB2* (At1g29920), *CAB3* (At1g29910), *CAB1*.*5* (At2g34420) and *CAB2*.*3* (At3g27690). Thus, JA may positively modulate the expression of photosynthesis-related genes in the early stage of seedling development and then negatively regulate different photosynthesis-related genes at the later stage of plant development or under high light by reducing FIN219/JAR1 level.

Hormone interactions have been well documented in the regulation of plant growth, development and defense [21,36,37]. JA also crosstalks with auxin and gibberellin signaling pathways by targeting the AUX/IAA-interacting partner TOPLESS (TPL) and DELLA proteins, respectively [38,39,40]. Moreover, JA, SA and ethylene play important roles in regulating plant defense responses against various pathogens, pests, and abiotic stresses such as wounding and ozone [41,42]. Recent evidence indicates that JA enhances the transcriptional activities of EIN3/EIL1 in ethylene signaling by degrading JAZ proteins that physically interact with EIN3/EIL1 and repress their function [43]. As well, MeJA can antagonize GA-mediated plant growth by enhancing the levels of DELLA proteins in rice and *Arabidopsis* [40]. Moreover, JAZ repressors directly interfere with the DELLA—PIF interaction, so JA hormone and triggered signaling can hinder a GA signaling cascade [40]. Here, our microarray studies revealed that ectopic expression of *FIN219/JAR1* (*pGR21*9) significantly upregulated ethylene-related genes and signaling and downregulated auxin-related genes and signaling (Table 1), which is consistent with recent findings [43]. Intriguingly, in *fin219-2*, MeJA treatment increased the number of downregulated genes involved in ethylene-related activities and signaling and the number of upregulated genes involved in auxin-related activities and signaling, which suggests that JA crosstalks with multiple hormone signaling in a FIN219/JAR1-independent or-dependent manner under FR light. This implication is consistent with the expression profiles of FIN219/JAR1 with and without MeJA under FR light (Fig 2).

JA regulates a wide variety of plant physiological processes, from growth and development to reproduction and defense [3,12,44]. With JA-triggered signaling, JAZ-regulated TFs play important roles in these physiological responses. A number of TFs, including AP2/ERF, bHLH, and WRKY, are involved in JA-mediated secondary metabolite biosynthesis [45]. MYC2 is a bHLH TF that is the master regulator involved in JA-mediated plant development, lateral and adventitious root formation, flowering time and shade avoidance syndrome [13,20,25]. Moreover, it participates in resistant responses to pathogens [3,14, 24] and crosstalk between JA and other hormones such as ABA, SA, GAs and auxin [3,46,47] as well as light signaling and the circadian clock [23,48]. Recent evidence indicates that MYC3 and MYC4 are targets of JAZ repressors and function synergistically with MYC2 in regulating JA-mediated responses such as root elongation, and herbivoral and pathogen responses [14]. Our microarray data revealed that FIN219/JAR1 regulates a great number of TFs. FIN219 positively regulated 3 other bHLH TFs (bHLH139; bHLH51; bHLH27) in addition to MYC2 in the absence or presence of MeJA. Further validation of gene expression also confirmed that FIN219 indeed positively regulated the expression of *bHLH27* and *bHLH51* in FR only or in FR with MeJA (Fig 5F and 5G). Moreover, both *bhlh27* and *bhlh51* mutants showed a less sensitive response to MeJA-mediated inhibition of root and hypocotyl elongation (Fig 6 and S7 Fig), so both bHLH27 and bHLH51 may function as positive regulators in modulating hypocotyl and root elongation. This finding is consistent with a recent report showing that bHLH25 and bHLH27 interact with each other to participate in regulating primary and lateral root development as well as susceptibility to the cyst nematode *Heterodera schachtii* [49]. However, the molecular mechanism of how FIN219 regulates both bHLH27 and bHLH51 functions in control of root development in response to MeJA remains elusive.

In addition, FIN219 negatively regulated some bHLH TFs under LFR in the absence or presence of MeJA (Figs 4 and 5, S6 Fig). Intriguingly, 3 of 9 validated bHLH TFs (bHLH32, bHLH85 and bHLH86) can regulate root hair development [5,50,51,52]. Thus, FIN219 likely can modulate root hair development by suppressing these bHLH TFs in response to MeJA. This speculation is supported by a previous report showing that JA and MeJA can significantly promote root hair formation [53]. As well, FIN219 differentially regulated CIB1 and CIB5 under LFR light in the presence of MeJA. CIB1 is involved in blue-light—mediated modulation of floral initiation and interacts with the blue-light photoreceptor cryptochrome 2 under blue light but not red light or darkness [27]. Moreover, CIB5 can interact with CIB1 [27]. Thus, how CIB1 and CIB5 regulated by FIN219 under MeJA stimulation are involved in FR light signaling remains to be elucidated.

## Conclusions

**Fig 7:**
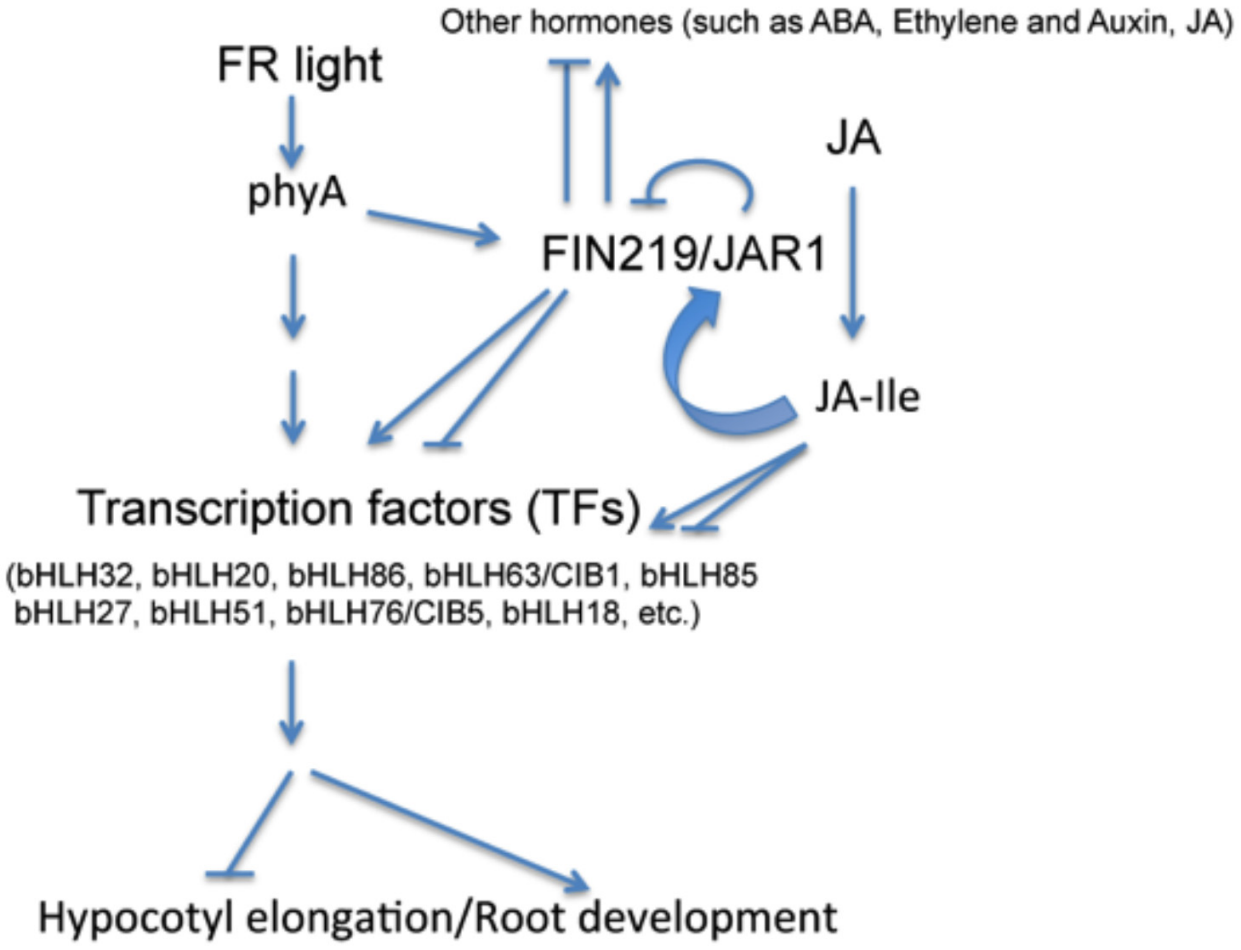
A model to illustrate FIN219/JAR1 functions in the integration of FR light and JA signaling. FR light perceived by the photoreceptor phyA regulates gene expression likely via FIN219/JAR1-mediated positive or negative modulation of TFs, including bHLH TFs. FIN219/JAR1 as a JA-conjugating enzyme is mainly responsible for the production of JA-Ile, an active form of JA hormone involved in regulating various physiological responses, and crosstalks positively and negatively with other hormones such as ABA, ethylene, auxin and JA, for regulation of hypocotyl elongation and root development. Regular arrow, positive effect; inverted T, negative effect.

Integration of FR light and JA signaling regulates *Arabidopsis* seedling development. FIN219/JAR1 as a JA-conjugating enzyme shows a negative feedback regulation in response to MeJA to modulate hypocotyl elongation under FR light. High FR light can reduce the sensitivity of *Arabidopsis* seedlings to exogenous MeJA. Moreover, FR light regulates a great number of genes likely via FIN219/JAR1 crosstalk with multiple hormones that may modulate many TFs, including bHLH TFs, in a FIN219/JAR1-dependent or-independent manner, for regulation of hypocotyl elongation and root development (Fig 7).

## Supporting Information

S1 Fig The seedlings of wild-type Columbia (Col), *fin219-2* and *PGR219* showed reduced hypocotyl length with increasing methyl jasmonate (JA) concentrations under low and high far-red (FR) light. Hypocotyl lengths of Col (**A, left**), *fin219-2* (**A, middle**) and *PGR219* (**A, right**) seedlings grown on GM plates containing different concentrations of methyl JA (M0~M100 μM) under low FR light (2 μmol m^-2^ s^-1^), high FR light (10 μmol m^-2^ s^-1^) **(A)** or white light (70 μmol m^-2^ s^-1^) **(B)** were measured by use of ImageJ. Data are mean±SEM from 3 biological replicates. Different lowercase letters represent significant differences by Tukey’s studentized range test at *P*< 0.05.
(PDF)

S2 Fig Gel blot analysis of FIN219/JAR1 protein detection by monoclonal antibodies raised against the full-length HPLC-purified recombinant protein FIN219. Seedlings of wild-type Col-0 and *fin219-2* mutant were grown in FR light (1.5 μmol m^-2^s^-1^) for 3 days. The extracted total proteins were used for gel blot analysis. Each lane contains 80 μg total proteins. The dilution factors (5000X, 10000X, 20000X and 40000X) are shown above the blot.
(PDF)

S3 Fig Gel blot analysis of detection specificity for polyclonal antibodies raised against JAZ1 protein. Seedlings of wild-type Col, *fin219-2* and *jaz1* mutants grown under FR light (1.5 μmol m^-2^s^-1^) for 3 days were used for extraction of total proteins and then protein gel blot analysis. Total proteins 100 μg were loaded in each lane and the resulting protein blot was probed with the polyclonal antibodies against JAZ1 at dilution ratio 10000X.
(PDF)

S4 Fig MeJA-dependent changes in transcript levels in *fin219-2* mutant under FR light (10 μmol m^-2^ s^-1^). Changes in mRNA levels before (**A**) and after (**B**) 50 μM MeJA treatment. The intensity of expression ratios from the wild type (gBGSubSignal) and *fin219-2* mutant (rBGSubSignal) are shown on the x- and y-axis, respectively.
(PDF)

S5 Fig Relative ratio of gene ontology (GO) groups among gene expression profiles affected by *FIN219* levels under low FR light without or with MeJA treatment. Relative ratio of GO groups among gene expression profiles affected by *PGR219* levels versus wild-type Col with at least 2-fold difference under low FR light (A) or with 50 μM MeJA (B). Relative ratio calculated as gene number in each GO group / total affected genes (7,577 in *PGR219*/Col and 7,311 in *PGR219*/Col with MeJA) * 100%.
(PDF)

S6 Fig The *fin219-2* mutant shows altered expression of a number of transcription factors (TFs) under low FR light with or without MeJA. Hierarchical clustering analysis of basic helix-loop-helix (bHLH) TFs in *fin219-2* under low FR light without or with 50 μM MeJA from microarray assay of seedlings of wild type and *fin219-2* mutant grown under low FR light without or with 50 μM MeJA for 3 days. Red asterisk marks the genes *MYC2*, *At5g43175* (bHLH139), *At2g40200* (bHLH51) and *At4g29930* (bHLH27) downregulated in *fin219-2* under both low FR light and MeJA.
(PDF)

S7 Fig Loss-of-function mutants of selected bHLH TFs show altered root responses to exogenous MeJA. (**A-E)** Root elongation of wild-type Col-0, *fin219-2* and selected bHLH TF mutants without or with MeJA under white light. Three-day-old seedlings were transferred to GM plates without (-MeJA) or with 5 μM MeJA (+MeJA) and then grown for another 7 days. In each panel, 2 represented seedlings were shown for each TF mutant. Scale bar is 5 mm. (**F-G)** Quantification of hypocotyl lengths of seedlings shown in **(A-E)** without **(F)** or with **(G)** MeJA treatment. (**H)** MeJA-mediated inhibition of root elongation shown in **(A-E)**. Data are mean±SEM from 3 biological replicates. Different lowercase letters represent significant differences by Tukey’s studentized range test at *P*< 0.05.
(PDF)

S8 Fig Characterization of selected *bhlh* mutants. Confirmation of the homozygous *bhlh* mutants used for studies involved genotyping. Seedlings of wild-type Col and selected *bhlh* mutants were grown under white light for 5 days and then used for extraction of genomic DNA. Genomic PCR was used to amplify genomic fragments of selected TF genes with gene-specific primers (See S3 Table) **(A)** or the T-DNA primer and gene-specific primer combined **(B)**.
(PDF)

S1 Table Gene ontology (GO) groups derived from expression profiles in *fin219-2* mutant without and with 50 μM methyl jasmonate (MeJA) treatment under low far-red (FR) light. (PDF)

S2 Table Primer pairs for quantitative real-time PCR. (PDF)

S3 Table Some characteristics of T-DNA insertion mutants for selected basic helix-loop-helix (bHLH) transcription factors. (PDF)

S4 Table Gene list and gene expression in *fin219-2*/Col without MeJA treatment. (PDF)

S5 Table Gene list and gene expression in *fin219-2*/Col in the presence of 50 μM MeJA. (PDF)

S6 Table List of hormone-related genes in *fin219*/Col. (PDF)

S7 Table List of hormone-related genes in *PGR219*/Col. (PDF)

S8 Table Gene list and expression data for bHLH TFs affected in *fin219-2* mutant derived from microarray studies. The selected TFs for further characterization are highlighted in grey color.
(PDF)
