# Supplementary material for: Far-Red Light-Mediated Seedling Development in Arabidopsis Involves FAR-RED INSENSITIVE 219/JASMONATE RESISTANT 1-Dependent and -Independent Pathways
Source: PLoS One. 2015 Jul 15;10(7):e0132723. doi: 10.1371/journal.pone.0132723 (PMC4503420; doi:10.1371/journal.pone.0132723)
Supplement: S1 Fig — Hypocotyl lengths of Col (A, left), fin219-2 (A, middle) and PGR219 (A, right) seedlings grown on GM plates containing different concentrations of methyl JA (M0~M100 μM) under low FR light (2 μmol m-2 s-1), high FR light (10 μmol m-2 s-1) (A) or white light (70 μmol m-2 s-1) (B) were measured by use of ImageJ. Data are mean±SEM from 3 biological replicates. Different lowercase letters represent significant differences by Tukey’s studentized range test at P< 0.05. (PDF) [file pone.0132723.s001.pdf]

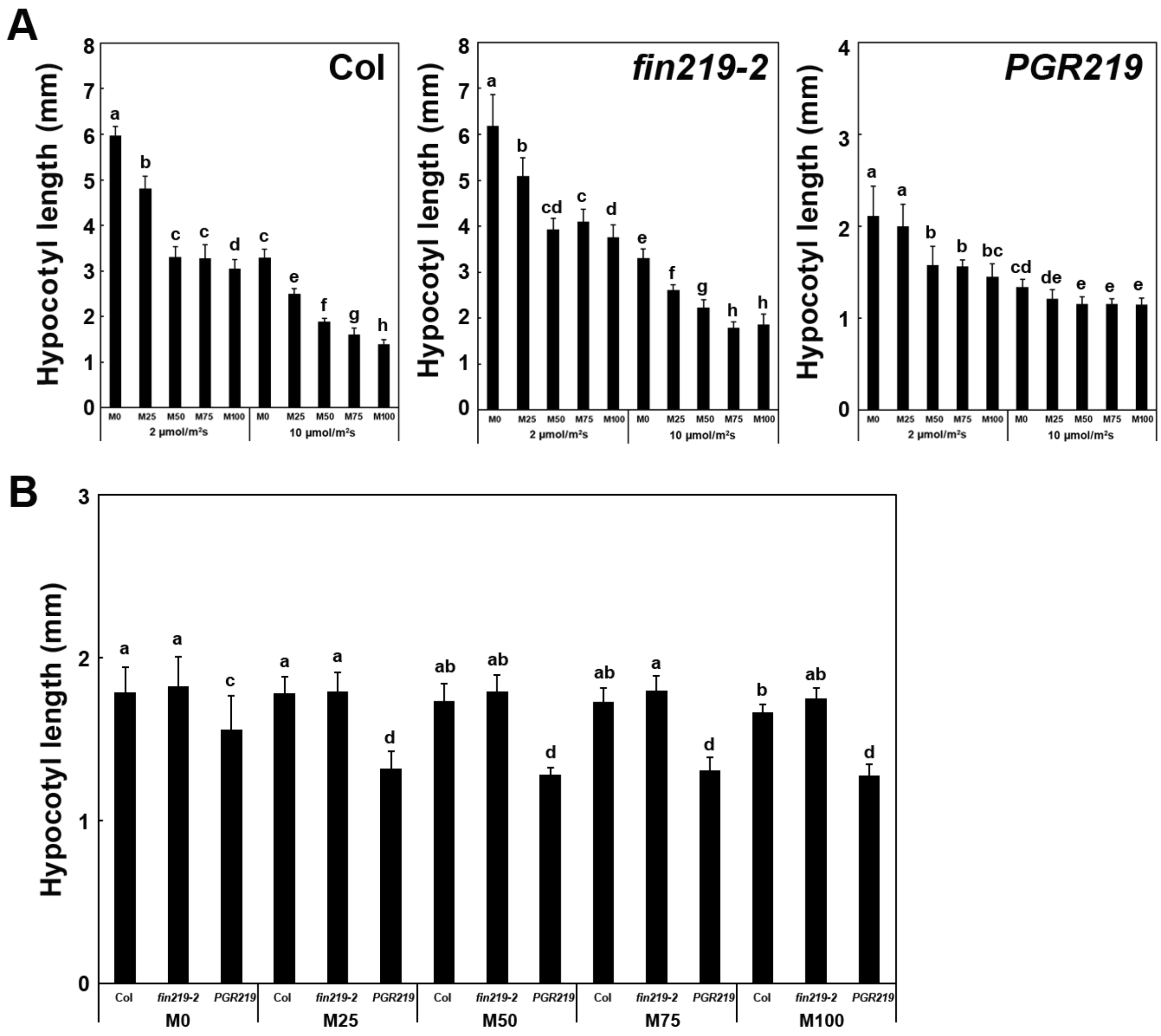

S1 Fig. The seedlings of wild-type Columbia (Col), *fin219-2* and *PGR219* showed reduced hypocotyl length with increasing methyl jasmonate (JA) concentrations under low and high far-red (FR) light. Hypocotyl lengths of Col (**A, left**), *fin219-2* (**A, middle**) and *PGR219* (**A, right**) seedlings grown on GM plates containing different concentrations of methyl JA (M0~M100 μM) under low FR light (2 μmol $m^{-2} s^{-1}$), high FR light (10 μmol $m^{-2} s^{-1}$) **(A)** or white light (70 μmol $m^{-2} s^{-1}$) **(B)** were measured by use of ImageJ. Data are mean±SEM from 3 biological replicates. Different lowercase letters represent significant differences by Tukey's studentized range test at $P< 0.05$.
