# Supplementary material for: Far-Red Light-Mediated Seedling Development in Arabidopsis Involves FAR-RED INSENSITIVE 219/JASMONATE RESISTANT 1-Dependent and -Independent Pathways
Source: PLoS One. 2015 Jul 15;10(7):e0132723. doi: 10.1371/journal.pone.0132723 (PMC4503420; doi:10.1371/journal.pone.0132723)
Supplement: S2 Fig — Seedlings of wild-type Col-0 and fin219-2 mutant were grown in FR light (1.5 μmol m-2s-1) for 3 days. The extracted total proteins were used for gel blot analysis. Each lane contains 80 μg total proteins. The dilution factors (5000X, 10000X, 20000X and 40000X) are shown above the blot. (PDF) [file pone.0132723.s002.pdf]

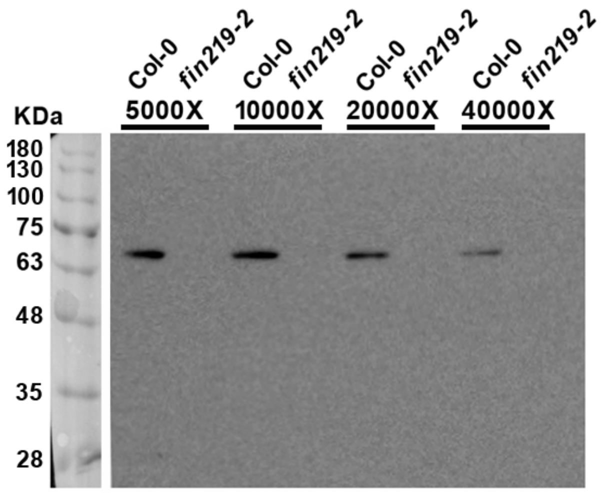

S2 Fig. Gel blot analysis of FIN219/JAR1 protein detection by monoclonal antibodies raised against the full-length HPLC-purified recombinant protein FIN219. Seedlings of wild-type Col-0 and *fin219-2* mutant were grown in FR light (1.5 μmol $m^{-2}s^{-1}$) for 3 days. The extracted total proteins were used for gel blot analysis. Each lane contains 80 μg total proteins. The dilution factors (5000X, 10000X, 20000X and 40000X) are shown above the blot.
