# Supplementary material for: Far-Red Light-Mediated Seedling Development in Arabidopsis Involves FAR-RED INSENSITIVE 219/JASMONATE RESISTANT 1-Dependent and -Independent Pathways
Source: PLoS One. 2015 Jul 15;10(7):e0132723. doi: 10.1371/journal.pone.0132723 (PMC4503420; doi:10.1371/journal.pone.0132723)
Supplement: S3 Fig — Seedlings of wild-type Col, fin219-2 and jaz1 mutants grown under FR light (1.5 μmol m-2s-1) for 3 days were used for extraction of total proteins and then protein gel blot analysis. Total proteins 100 μg were loaded in each lane and the resulting protein blot was probed with the polyclonal antibodies against JAZ1 at dilution ratio 10000X. (PDF) [file pone.0132723.s003.pdf]

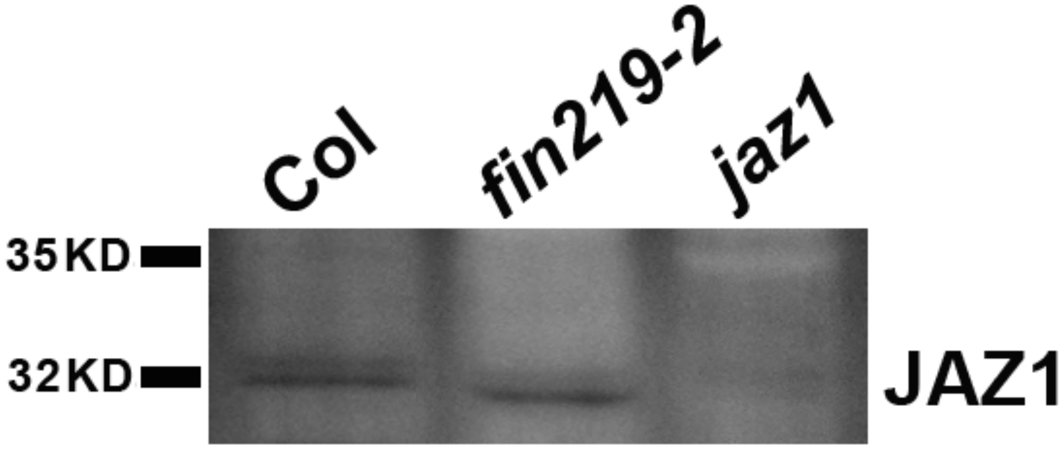

S3 Fig. Gel blot analysis of detection specificity for polyclonal antibodies raised against JAZ1 protein. Seedlings of wild-type Col, *fin219-2* and *jaz1* mutants grown under FR light (1.5 μmol $m^{-2}s^{-1}$) for 3 days were used for extraction of total proteins and then protein gel blot analysis. Total proteins 100 μg were loaded in each lane and the resulting protein blot was probed with the polyclonal antibodies against JAZ1 at dilution ratio 10000X.
