# Supplementary material for: Far-Red Light-Mediated Seedling Development in Arabidopsis Involves FAR-RED INSENSITIVE 219/JASMONATE RESISTANT 1-Dependent and -Independent Pathways
Source: PLoS One. 2015 Jul 15;10(7):e0132723. doi: 10.1371/journal.pone.0132723 (PMC4503420; doi:10.1371/journal.pone.0132723)
Supplement: S4 Fig — Changes in mRNA levels before (A) and after (B) 50 μM MeJA treatment. The intensity of expression ratios from the wild type (gBGSubSignal) and fin219-2 mutant (rBGSubSignal) are shown on the x- and y-axis, respectively. (PDF) [file pone.0132723.s004.pdf]

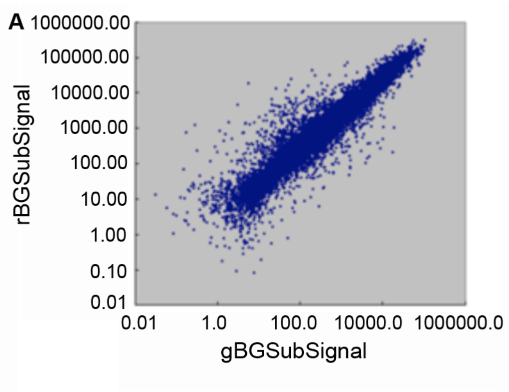

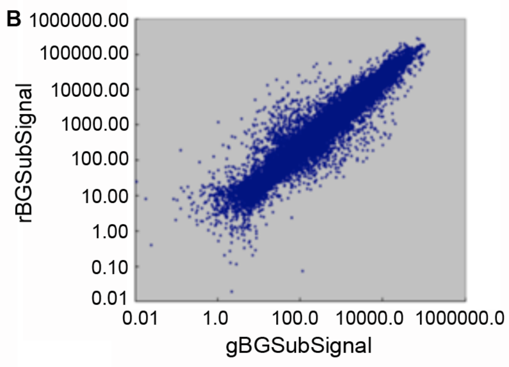

S4 Fig. MeJA-dependent changes in transcript levels in *fin219-2* mutant under FR light (10 µmol m^{-2} s^{-1}). Changes in mRNA levels before (**A**) and after (**B**) 50 µM MeJA treatment. The intensity of expression ratios from the wild type (gBGSubSignal) and *fin219-2* mutant (rBGSubSignal) are shown on the x- and y-axis, respectively.
