# Supplementary material for: Far-Red Light-Mediated Seedling Development in Arabidopsis Involves FAR-RED INSENSITIVE 219/JASMONATE RESISTANT 1-Dependent and -Independent Pathways
Source: PLoS One. 2015 Jul 15;10(7):e0132723. doi: 10.1371/journal.pone.0132723 (PMC4503420; doi:10.1371/journal.pone.0132723)
Supplement: S5 Fig — Relative ratio of GO groups among gene expression profiles affected by PGR219 levels versus wild-type Col with at least 2-fold difference under low FR light (A) or with 50 μM MeJA (B). Relative ratio calculated as gene number in each GO group / total affected genes (7,577 in PGR219/Col and 7,311 in PGR219/Col with MeJA) * 100%. (PDF) [file pone.0132723.s005.pdf]

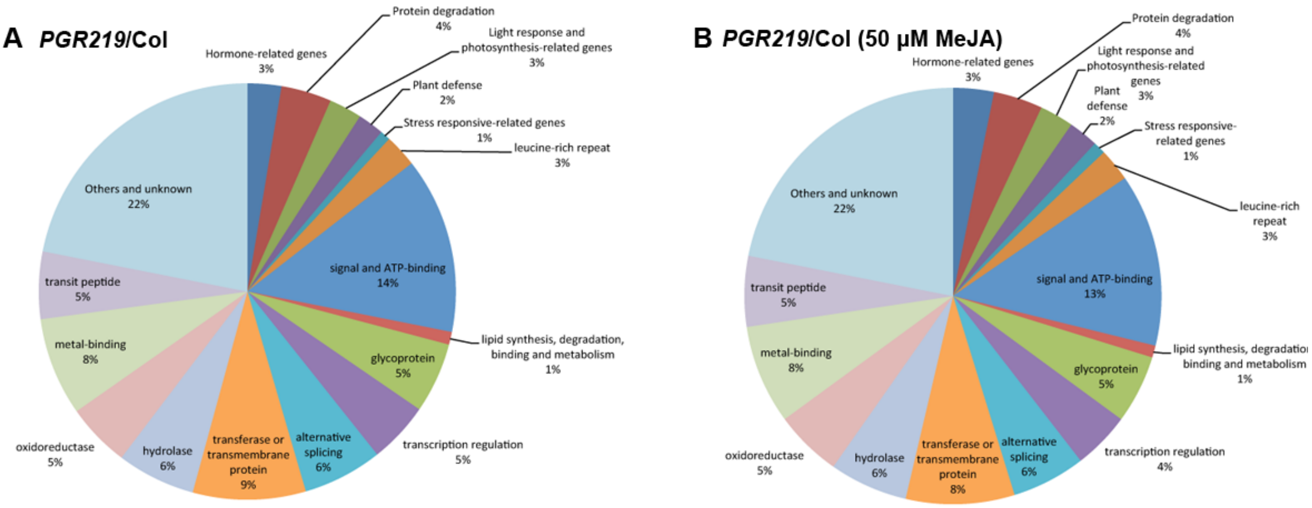

S5 Fig. Relative ratio of gene ontology (GO) groups among gene expression profiles affected by *FIN219* levels under low FR light without or with MeJA treatment. Relative ratio of GO groups among gene expression profiles affected by *PGR219* levels versus wild-type Col with at least 2-fold difference under low FR light (A) or with 50 µM MeJA (B). Relative ratio calculated as gene number in each GO group / total affected genes (7,577 in *PGR219*/Col and 7,311 in *PGR219*/Col with MeJA) * 100%.
