# Supplementary material for: Far-Red Light-Mediated Seedling Development in Arabidopsis Involves FAR-RED INSENSITIVE 219/JASMONATE RESISTANT 1-Dependent and -Independent Pathways
Source: PLoS One. 2015 Jul 15;10(7):e0132723. doi: 10.1371/journal.pone.0132723 (PMC4503420; doi:10.1371/journal.pone.0132723)
Supplement: S6 Fig — Hierarchical clustering analysis of basic helix-loop-helix (bHLH) TFs in fin219-2 under low FR light without or with 50 μM MeJA from microarray assay of seedlings of wild type and fin219-2 mutant grown under low FR light without or with 50 μM MeJA for 3 days. Red asterisk marks the genes MYC2, At5g43175 (bHLH139), At2g40200 (bHLH51) and At4g29930 (bHLH27) downregulated in fin219-2 under both low FR light and MeJA. (PDF) [file pone.0132723.s006.pdf]

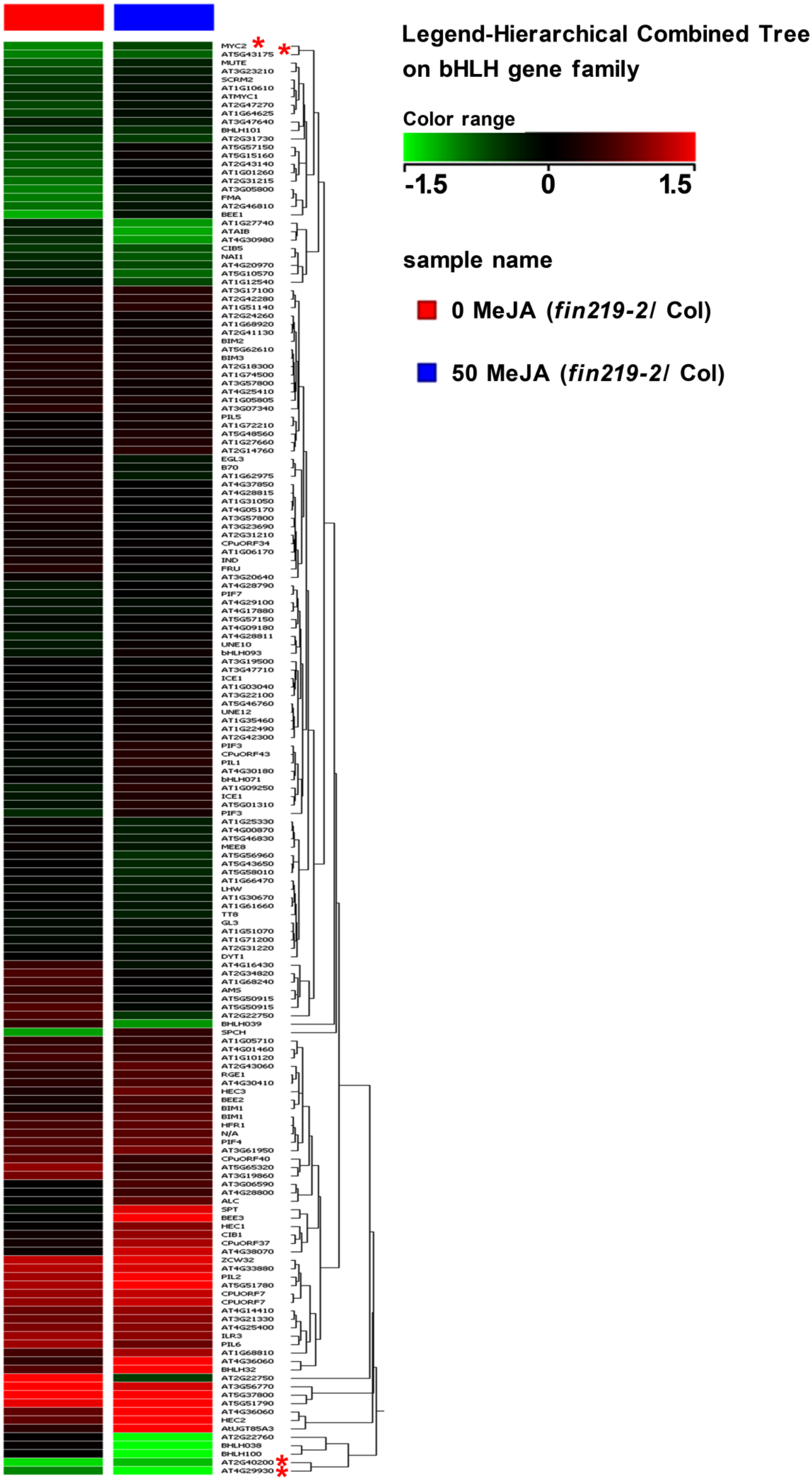
Legend-Hierarchical Combined Tree
on bHLH gene family
Color range
-1.5
0
1.5
sample name
0 MeJA (*fin219-2*/ Col)
50 MeJA (*fin219-2*/ Col)

S6 Fig. The *fin219-2* mutant shows altered expression of a number of transcription factors (TFs) under low FR light with or without MeJA. Hierarchical clustering analysis of basic helix-loop-helix (bHLH) TFs in *fin219-2* under low FR light without or with 50 μM MeJA from microarray assay of seedlings of wild type and *fin219-2* mutant grown under low FR light without or with 50 μM MeJA for 3 days. Red asterisk marks the genes *MYC2*, *At5g43175* (bHLH139), *At2g40200* (bHLH51) and *At4g29930* (bHLH27) downregulated in *fin219-2* under both low FR light and MeJA.
