# Supplementary material for: Far-Red Light-Mediated Seedling Development in Arabidopsis Involves FAR-RED INSENSITIVE 219/JASMONATE RESISTANT 1-Dependent and -Independent Pathways
Source: PLoS One. 2015 Jul 15;10(7):e0132723. doi: 10.1371/journal.pone.0132723 (PMC4503420; doi:10.1371/journal.pone.0132723)
Supplement: S7 Fig — (A-E) Root elongation of wild-type Col-0, fin219-2 and selected bHLH TF mutants without or with MeJA under white light. Three-day-old seedlings were transferred to GM plates without (-MeJA) or with 5 μM MeJA (+MeJA) and then grown for another 7 days. In each panel, 2 represented seedlings were shown for each TF mutant. Scale bar is 5 mm. (F-G) Quantification of hypocotyl lengths of seedlings shown in (A-E) without (F) or with (G) MeJA treatment. (H) MeJA-mediated inhibition of root elongation shown in (A-E). Data are mean±SEM from 3 biological replicates. Different lowercase letters represent significant differences by Tukey’s studentized range test at P< 0.05. (PDF) [file pone.0132723.s007.pdf]

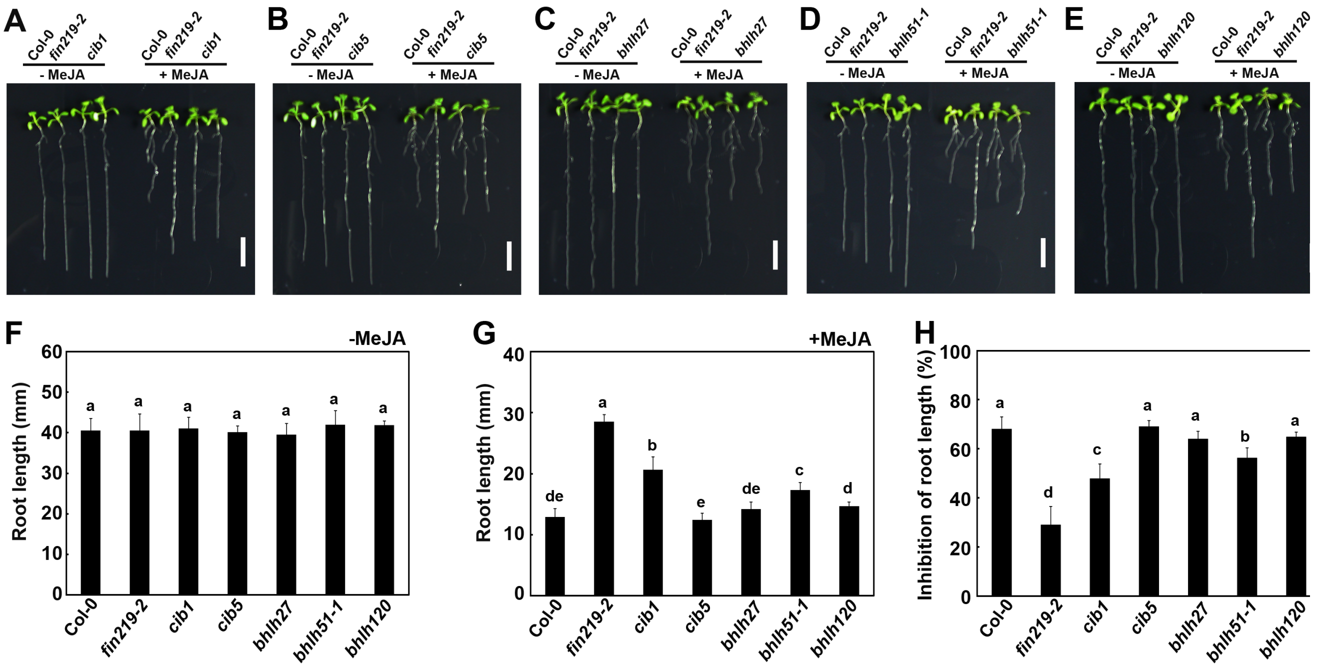

S7 Fig. Loss-of-function mutants of selected bHLH TFs show altered root responses to exogenous MeJA. (**A-E**) Root elongation of wild-type Col-0, *fin219-2* and selected bHLH TF mutants without or with MeJA under white light. Three-day-old seedlings were transferred to GM plates without (-MeJA) or with 5 μM MeJA (+MeJA) and then grown for another 7 days. In each panel, 2 represented seedlings were shown for each TF mutant. Scale bar is 5 mm. (**F-G**) Quantification of hypocotyl lengths of seedlings shown in (**A-E**) without (**F**) or with (**G**) MeJA treatment. (**H**) MeJA-mediated inhibition of root elongation shown in (**A-E**). Data are mean±SEM from 3 biological replicates. Different lowercase letters represent significant differences by Tukey's studentized range test at $P < 0.05$.
