# Supplementary material for: Far-Red Light-Mediated Seedling Development in Arabidopsis Involves FAR-RED INSENSITIVE 219/JASMONATE RESISTANT 1-Dependent and -Independent Pathways
Source: PLoS One. 2015 Jul 15;10(7):e0132723. doi: 10.1371/journal.pone.0132723 (PMC4503420; doi:10.1371/journal.pone.0132723)
Supplement: S8 Fig — Confirmation of the homozygous bhlh mutants used for studies involved genotyping. Seedlings of wild-type Col and selected bhlh mutants were grown under white light for 5 days and then used for extraction of genomic DNA. Genomic PCR was used to amplify genomic fragments of selected TF genes with gene-specific primers (See S3 Table) (A) or the T-DNA primer and gene-specific primer combined (B). (PDF) [file pone.0132723.s008.pdf]

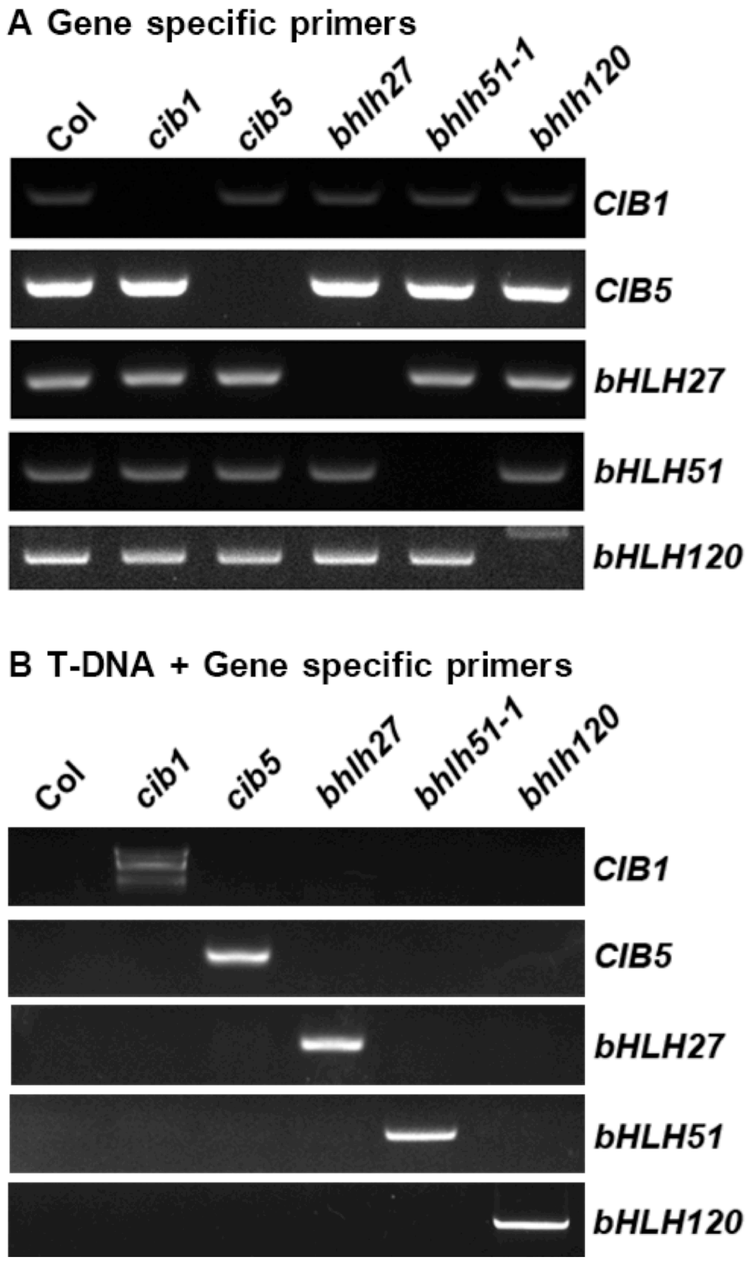

S8 Fig. Characterization of selected *bhlh* mutants. Confirmation of the homozygous *bhlh* mutants used for studies involved genotyping. Seedlings of wild-type Col and selected *bhlh* mutants were grown under white light for 5 days and then used for extraction of genomic DNA. Genomic PCR was used to amplify genomic fragments of selected TF genes with gene-specific primers (See Table S3) **(A)** or the T-DNA primer and gene-specific primer combined **(B)**.
