# Supplementary material for: Far-Red Light-Mediated Seedling Development in Arabidopsis Involves FAR-RED INSENSITIVE 219/JASMONATE RESISTANT 1-Dependent and -Independent Pathways
Source: PLoS One. 2015 Jul 15;10(7):e0132723. doi: 10.1371/journal.pone.0132723 (PMC4503420; doi:10.1371/journal.pone.0132723)
Supplement: S1 Table — (PDF) [file pone.0132723.s009.pdf]

S1 Table. Gene ontology (GO) groups derived from expression profiles in *fin219-2* mutant without and with 50 µM methyl jasmonate (MeJA) treatment under low far-red (FR) light.

GO ID	GO ACCESSION	GO Term	Corrected p-value	Count in Selection	% Count in Selection
group A					
3707	GO:0005529	sugar binding	0.0943	7	36.8
4790	GO:0006869	lipid transport	0.0170	12	63.2
5654	GO:0008289	lipid binding	0.0943	11	57.9
7959	GO:0010876	lipid localization	0.0302	12	63.2
group B					
2475	GO:0003824	catalytic activity	0.0000	112	31.8
2610	GO:0003993	acid phosphatase activity	0.0273	7	2.0
2841	GO:0004356	glutamate-ammonia ligase activity	0.0962	3	0.9
2958	GO:0004497	monooxygenase activity	0.0001	22	6.3
3540	GO:0005215|GO:0005478	transporter activity	0.0779	20	5.7
3648	GO:0005381|GO:0005382|GO:0016033	iron ion transmembrane transporter activity	0.0620	3	0.9
3691	GO:0005506	iron ion binding	0.0003	25	7.1
3723	GO:0005576	extracellular region	0.0809	5	1.4
3904	GO:0005811	lipid particle	0.0071	5	1.4
4034	GO:0005975	carbohydrate metabolic process	0.0391	24	6.8
4141	GO:0006082	organic acid metabolic process	0.0000	15	4.3
4480	GO:0006519	cellular amino acid and derivative metabolic process	0.0000	6	1.7
4481	GO:0006520	cellular amino acid metabolic process	0.0452	3	0.9
4536	GO:0006575	cellular amino acid derivative metabolic process	0.0002	3	0.9
4677	GO:0006725	cellular aromatic compound metabolic process	0.0000	9	2.6
4729	GO:0006790	sulfur metabolic process	0.0002	11	3.1
4757	GO:0006826|GO:0015681	iron ion transport	0.0732	3	0.9
4855	GO:0006950	response to stress	0.0000	64	18.2
4856	GO:0006952|GO:0002217|GO:0042829	defense response	0.0024	18	5.1

4870	GO:0006970	response to osmotic stress	0.0809	18	5.1
5520	GO:0008113|GO:0033742	peptide-methionine-(S)-S-oxide reductase activity	0.0582	4	1.1
5544	GO:0008146	sulfotransferase activity	0.0348	5	1.4
5930	GO:0008652	cellular amino acid biosynthetic process	0.0009	2	0.6
6281	GO:0009055|GO:0009053|GO:0009054	electron carrier activity	0.0107	28	8.0
6284	GO:0009058	biosynthetic process	0.0067	31	8.8
6298	GO:0009072	aromatic amino acid family metabolic process	0.0038	2	0.6
6299	GO:0009073|GO:0016089	aromatic amino acid family biosynthetic process	0.0091	2	0.6
6487	GO:0009266	response to temperature stimulus	0.0487	17	4.8
6520	GO:0009308	amine metabolic process	0.0273	3	0.9
6521	GO:0009309	amine biosynthetic process	0.0003	2	0.6
6606	GO:0009409	response to cold	0.0019	16	4.5
6611	GO:0009415	response to water	0.0620	2	0.6
6681	GO:0009532	plastid stroma	0.0620	23	6.5
6716	GO:0009570	chloroplast stroma	0.0348	23	6.5
6743	GO:0009605	response to external stimulus	0.0457	15	4.3
6745	GO:0009607	response to biotic stimulus	0.0000	6	1.7
6749	GO:0009611|GO:0002245	response to wounding	0.0017	15	4.3
6760	GO:0009628	response to abiotic stimulus	0.0019	34	9.7
6780	GO:0009651	response to salt stress	0.0322	18	5.1
6821	GO:0009694	jasmonic acid metabolic process	0.0726	5	1.4
6822	GO:0009695	jasmonic acid biosynthetic process	0.0420	5	1.4
6823	GO:0009696	salicylic acid metabolic process	0.0324	4	1.1
6824	GO:0009697	salicylic acid biosynthetic process	0.0620	3	0.9
6825	GO:0009698	phenylpropanoid metabolic process	0.0024	3	0.9
6826	GO:0009699	phenylpropanoid biosynthetic process	0.0124	2	0.6
6846	GO:0009719	response to endogenous stimulus	0.0000	40	11.4
6852	GO:0009725	response to hormone stimulus	0.0076	20	5.7
6864	GO:0009737	response to abscisic acid stimulus	0.0014	20	5.7
6880	GO:0009753	response to jasmonic acid stimulus	0.0000	22	6.3
7136	GO:0010033	response to organic substance	0.0000	40	11.4
7434	GO:0010344	seed oilbody biogenesis	0.0173	3	0.9

7959	GO:0010876	lipid localization	0.0891	5	1.4
8085	GO:0012505	endomembrane system	0.0001	151	42.9
8091	GO:0012511|GO:0009520	monolayer-surrounded lipid storage body	0.0071	5	1.4
9187	GO:0016051|GO:0006093	carbohydrate biosynthetic process	0.0004	12	3.4
9189	GO:0016053	organic acid biosynthetic process	0.0000	12	3.4
9263	GO:0016137	glycoside metabolic process	0.0001	9	2.6
9264	GO:0016138	glycoside biosynthetic process	0.0006	8	2.3
9269	GO:0016143	S-glycoside metabolic process	0.0001	9	2.6
9270	GO:0016144	S-glycoside biosynthetic process	0.0003	8	2.3
9288	GO:0016165	lipoxygenase activity	0.0962	3	0.9
9291	GO:0016168	chlorophyll binding	0.0726	5	1.4
9504	GO:0016491	oxidoreductase activity	0.0000	45	12.8
9674	GO:0016701	oxidoreductase activity, acting on single donors with incorporation of molecular oxygen	0.0186	3	0.9
9675	GO:0016702	oxidoreductase activity, acting on single donors with incorporation of molecular oxygen, incorporation of two atoms of oxygen	0.0420	3	0.9
9721	GO:0016757|GO:0016932	transferase activity, transferring glycosyl groups	0.0057	19	5.4
11369	GO:0019438	aromatic compound biosynthetic process	0.0000	7	2.0
11659	GO:0019748	secondary metabolic process	0.0000	13	3.7
11663	GO:0019752	carboxylic acid metabolic process	0.0000	15	4.3
11666	GO:0019757	glycosinolate metabolic process	0.0001	9	2.6
11667	GO:0019758	glycosinolate biosynthetic process	0.0003	8	2.3
11726	GO:0019825	oxygen binding	0.0003	22	6.3
11792	GO:0019915	lipid storage	0.0226	5	1.4
11881	GO:0020037	heme binding	0.0008	22	6.3
12499	GO:0022857|GO:0005386|GO:0015646	transmembrane transporter activity	0.0366	6	1.7
12587	GO:0030076	light-harvesting complex	0.0726	5	1.4
13770	GO:0031407	oxylipin metabolic process	0.0023	7	2.0
13771	GO:0031408	oxylipin biosynthetic process	0.0080	6	1.7
15140	GO:0032787	monocarboxylic acid metabolic process	0.0002	12	3.4
16970	GO:0034637	cellular carbohydrate biosynthetic process	0.0024	8	2.3
16974	GO:0034641	cellular nitrogen compound metabolic process	0.0076	3	0.9

17833	GO:0042180	cellular ketone metabolic process	0.0000	15	4.3
17874	GO:0042221	response to chemical stimulus	0.0000	42	11.9
18040	GO:0042398	cellular amino acid derivative biosynthetic process	0.0019	2	0.6
19004	GO:0043436	oxoacid metabolic process	0.0000	15	4.3
19216	GO:0043648	dicarboxylic acid metabolic process	0.0124	2	0.6
19665	GO:0044106	cellular amine metabolic process	0.0080	3	0.9
19808	GO:0044249	cellular biosynthetic process	0.0233	22	6.3
19821	GO:0044262|GO:0006092	cellular carbohydrate metabolic process	0.0335	9	2.6
19830	GO:0044271	cellular nitrogen compound biosynthetic process	0.0005	2	0.6
19831	GO:0044272	sulfur compound biosynthetic process	0.0012	8	2.3
19840	GO:0044281	small molecule metabolic process	0.0000	6	1.7
19842	GO:0044283	small molecule biosynthetic process	0.0000	4	1.1
20585	GO:0045735	nutrient reservoir activity	0.0000	13	3.7
21179	GO:0046394	carboxylic acid biosynthetic process	0.0000	12	3.4
21200	GO:0046417	chorismate metabolic process	0.0091	2	0.6
21667	GO:0046906	tetrapyrrole binding	0.0000	27	7.7
24460	GO:0050896|GO:0051869	response to stimulus	0.0000	89	25.3
24776	GO:0051213	dioxygenase activity	0.0098	3	0.9
25254	GO:0051704|GO:0051706	multi-organism process	0.0006	4	1.1
25256	GO:0051707|GO:0009613|GO:0042828	response to other organism	0.0000	4	1.1
groupC					
2418	GO:0003674|GO:0005554	molecular_function	0.0641	223	80.8
2475	GO:0003824	catalytic activity	0.0001	42	15.2
2967	GO:0004506	squalene monooxygenase activity	0.0521	3	1.1
4141	GO:0006082	organic acid metabolic process	0.0369	4	1.4
4480	GO:0006519	cellular amino acid and derivative metabolic process	0.0003	13	4.7
4481	GO:0006520	cellular amino acid metabolic process	0.0336	4	1.4
4536	GO:0006575	cellular amino acid derivative metabolic process	0.0368	8	2.9
4729	GO:0006790	sulfur metabolic process	0.0011	7	2.5
4855	GO:0006950	response to stress	0.0303	29	10.5

4856	GO:0006952|GO:0002217|GO:0042829	defense response	0.0001	18	6.5
5548	GO:0008152	metabolic process	0.0641	54	19.6
5930	GO:0008652	cellular amino acid biosynthetic process	0.0977	3	1.1
6323	GO:0009098	leucine biosynthetic process	0.0341	3	1.1
6520	GO:0009308	amine metabolic process	0.0139	6	2.2
6521	GO:0009309	amine biosynthetic process	0.0703	3	1.1
6608	GO:0009411	response to UV	0.0041	8	2.9
6745	GO:0009607	response to biotic stimulus	0.0304	3	1.1
6749	GO:0009611|GO:0002245	response to wounding	0.0041	13	4.7
6825	GO:0009698	phenylpropanoid metabolic process	0.0546	8	2.9
6826	GO:0009699	phenylpropanoid biosynthetic process	0.0380	7	2.5
6880	GO:0009753	response to jasmonic acid stimulus	0.0122	12	4.3
6935	GO:0009812	flavonoid metabolic process	0.0303	6	2.2
6936	GO:0009813	flavonoid biosynthetic process	0.0181	6	2.2
7320	GO:0010224	response to UV-B	0.0346	6	2.2
9263	GO:0016137	glycoside metabolic process	0.0001	7	2.5
9264	GO:0016138	glycoside biosynthetic process	0.0080	7	2.5
9269	GO:0016143	S-glycoside metabolic process	0.0000	7	2.5
9270	GO:0016144	S-glycoside biosynthetic process	0.0001	7	2.5
9291	GO:0016168	chlorophyll binding	0.0419	5	1.8
9504	GO:0016491	oxidoreductase activity	0.0006	20	7.2
9677	GO:0016705	oxidoreductase activity, acting on paired donors, with incorporation or reduction of molecular oxygen	0.0027	3	1.1
9780	GO:0016829	lyase activity	0.0117	4	1.4
11369	GO:0019438	aromatic compound biosynthetic process	0.0425	7	2.5
11659	GO:0019748	secondary metabolic process	0.0000	15	5.4
11663	GO:0019752	carboxylic acid metabolic process	0.0368	4	1.4
11666	GO:0019757	glycosinolate metabolic process	0.0000	7	2.5
11667	GO:0019758	glycosinolate biosynthetic process	0.0001	7	2.5
16974	GO:0034641	cellular nitrogen compound metabolic process	0.0012	4	1.4
17833	GO:0042180	cellular ketone metabolic process	0.0304	4	1.4
18040	GO:0042398	cellular amino acid derivative biosynthetic process	0.0315	7	2.5

19004	GO:0043436	oxoacid metabolic process	0.0368	4	1.4
19665	GO:0044106	cellular amine metabolic process	0.0304	4	1.4
19830	GO:0044271	cellular nitrogen compound biosynthetic process	0.0067	3	1.1
19831	GO:0044272	sulfur compound biosynthetic process	0.0304	7	2.5
19840	GO:0044281	small molecule metabolic process	0.0004	13	4.7
19842	GO:0044283	small molecule biosynthetic process	0.0006	10	3.6
24460	GO:0050896|GO:0051869	response to stimulus	0.0070	40	14.5
25256	GO:0051707|GO:0009613|GO:0042828	response to other organism	0.0380	1	0.4
groupD					
6611	GO:0009415	response to water	0.0394	2	100.0
group E					
2420	GO:0003677	DNA binding	0.0283	83	79.8
2435	GO:0003700|GO:0000130	transcription factor activity	0.0003	76	73.1
2958	GO:0004497	monooxygenase activity	0.0788	17	16.3
4855	GO:0006950	response to stress	0.0570	3	2.9
7005	GO:0009889	regulation of biosynthetic process	0.0151	36	34.6
7558	GO:0010468	regulation of gene expression	0.0198	36	34.6
7642	GO:0010556	regulation of macromolecule biosynthetic process	0.0151	36	34.6
11164	GO:0019219	regulation of nucleobase, nucleoside, nucleotide and nucleic acid metabolic process	0.0121	36	34.6
11167	GO:0019222	regulation of metabolic process	0.0198	36	34.6
12951	GO:0030528	transcription regulator activity	0.0013	77	74.0
13687	GO:0031323	regulation of cellular metabolic process	0.0151	36	34.6
13690	GO:0031326	regulation of cellular biosynthetic process	0.0151	36	34.6
20308	GO:0045449	regulation of transcription	0.0121	36	34.6
24359	GO:0050789|GO:0050791	regulation of biological process	0.0229	36	34.6
24363	GO:0050794|GO:0051244	regulation of cellular process	0.0570	36	34.6
24460	GO:0050896|GO:0051869	response to stimulus	0.0121	3	2.9
24734	GO:0051171	regulation of nitrogen compound metabolic process	0.0151	36	34.6
26369	GO:0060255	regulation of macromolecule metabolic process	0.0162	36	34.6

27138	GO:0065007	biological regulation	0.0038	36	34.6
29159	GO:0080090	regulation of primary metabolic process	0.0121	36	34.6
group F					
3285	GO:0004872	receptor activity	0.0036	12	75.0
3299	GO:0004888	transmembrane receptor activity	0.0018	11	68.8
4827	GO:0006915|GO:0008632	apoptosis	0.0526	9	56.3
8083	GO:0012501|GO:0016244	programmed cell death	0.0526	9	56.3
