# Supplementary material for: Far-Red Light-Mediated Seedling Development in Arabidopsis Involves FAR-RED INSENSITIVE 219/JASMONATE RESISTANT 1-Dependent and -Independent Pathways
Source: PLoS One. 2015 Jul 15;10(7):e0132723. doi: 10.1371/journal.pone.0132723 (PMC4503420; doi:10.1371/journal.pone.0132723)
Supplement: S2 Table — (PDF) [file pone.0132723.s010.pdf]

S2 Table. Primer pairs for quantitative real-time PCR.

Gene	Accession number	primer sequences (5’ →3’)
UBQ10	AT4G05320	F -TCCGGATCAGCAGAGGCTTA<br>R -TCAGAACTCTCCACCTCAAG
bHLH18	AT2G22750	F - TGCCCTTTGGACCCACTTTTGACA<br>R - TCCGTCCCAAATGAAACTGGAGAGA
bHLH27	AT4G29930	F - TGACATGGATGGGAGAGAAGACGG<br>R - TGGAGAGACGAGAGGTGAAGGAAG
bHLH32	AT3G25710	F - GTCTCTCCTCCTCCTTCCATGG<br>R - CTTGACTTAATTAACTAATTATAACATCG
bHLH51	AT2G40200	F - AGCGGAGAAAAGACGCCGTGAC<br>R - GCTTCGGGTTGATCTTCGCAGC
bHLH63 (*CIB1*)	AT4G34530	F - TCCACTCTCTCTCTCTCTCGTTGC<br>R - AATCACCTTCCACCGTCGTCTC
bHLH76 (*CIB5*)	AT1G26260	F - ACCACAATCTTGTCCACATGAACC<br>R - TTCCTCCTCTGTTTCGTTTTCACTC
bHLH85	AT4G33880	F - CTCATCACAACCACCTTAGCCTC<br>R -ACGTCACACTCACTTGTCTCCTC
bHLH86	AT5G37800	F - ACATCTCAACCCCAAATTCTTC<br>R -TGTAACTCATGTTCCCGTCC
bHLH120	AT5G51790	F - TTTCATCAAAGACACGCAAACAAGG<br>R - TGCTAAGCTGCTCACAACCAC
