# Supplementary material for: Far-Red Light-Mediated Seedling Development in Arabidopsis Involves FAR-RED INSENSITIVE 219/JASMONATE RESISTANT 1-Dependent and -Independent Pathways
Source: PLoS One. 2015 Jul 15;10(7):e0132723. doi: 10.1371/journal.pone.0132723 (PMC4503420; doi:10.1371/journal.pone.0132723)
Supplement: S3 Table — (PDF) [file pone.0132723.s011.pdf]

S3 Table. Some characteristics of T-DNA insertion mutants for selected basic helix-loop-helix (bHLH) transcription factors.

Gene name	Accession no.	T-DNA line	Position of T-DNA	Primer pairs for genotyping (5’→3’)
CIB1	At4G34530	CS821043	2' exon	F : CTTTCTCCACTCTCTCTCTCTC<br>R : TTTCTCTTCTAACTCGTTCTGC
CIB5	At1G26260	CS815870	5' exon	F : AGTGAAAGGATGAAGTTCTTGC<br>R : GCAGGTTAAAATCGAGCACAG
bHLH27	At4G29930	CS813379	4' exon	F : GAAAGTGACATGGATGGGAG<br>R : ACCAAAACAAGACACGTACAG
bHLH51	At2G40200	SALK_084837	1' intron	F : GGCTTCTCTTCTTCCTCCTTC<br>R : GGTGATTCTGCTGCTTTTTG
bHLH120	At5G51790	CS832667	1' exon	F : CAATCCTAAAAAGACAAGGCAC<br>R : CCAATCTCTCGCTTTAGCTC
