# Supplementary material for: Far-Red Light-Mediated Seedling Development in Arabidopsis Involves FAR-RED INSENSITIVE 219/JASMONATE RESISTANT 1-Dependent and -Independent Pathways
Source: PLoS One. 2015 Jul 15;10(7):e0132723. doi: 10.1371/journal.pone.0132723 (PMC4503420; doi:10.1371/journal.pone.0132723)
Supplement: S4 Table — (PDF) [file pone.0132723.s012.pdf]

S4 Table. Gene list and gene expression in *fin219-2*/Col without MeJA treatment.

GO Term	Count	Gene list (Expression ratio: without MeJA/ with MeJA*)					
**oxidoreductase**	**119**	AT5G24150(0.37/0.31*)	AT4G39510(0.31/0.86*)	AT5G17820(3.00/1.92*)	AT1G59900(12.08/12.15*)	AT1G62540(0.25/0.04*)	AT3G48000(0.44/0.64*)
		AT1G19250(8.13/2.01*)	AT4G03140(0.46/0.89*)	AT5G05580(0.38/0.27*)	AT5G22500(4.94/3.47*)	AT4G37310(0.13/0.16*)	AT2G41510(0.48/0.62*)
		AT1G72680(2.16/2.10*)	AT1G63460(3.45/3.29*)	AT5G23190(0.47/0.81*)	AT5G24160(0.22/0.23*)	AT5G21482(0.44/0.58*)	AT1G60740(3.01/5.18*)
		AT2G14170(2.18/1.96*)	AT4G15300(6.06/3.74*)	AT3G30775(0.46/0.17*)	AT1G06080(2.78/1.25*)	AT4G25090(2.11/3.81*)	AT5G08640(0.14/0.11*)
		AT1G58290(0.40/0.60*)	AT5G02540(1.03/0.78*)	AT5G25130(0.37/0.41*)	AT5G42590(2.32/1.38*)	AT2G37540(0.32/0.21*)	AT5G39190(68.20/51.10*)
		AT3G30180(3.42/2.29*)	AT1G62560(0.36/1.07*)	AT4G13770(0.05/0.06*)	AT5G64120(0.46/0.84*)	AT4G33010(0.59/0.43*)	AT3G49620(0.31/0.68*)
		AT3G45140(0.06/0.11*)	AT1G79470(0.43/0.47*)	AT5G58390(0.48/0.74*)	AT4G37430(1.45/0.88*)	AT3G55310(3.90/0.80*)	AT1G65340(2.48/1.36*)
		AT5G63600(2.89/2.12*)	AT4G36220(0.28/0.20*)	AT5G23980(0.20/0.66*)	AT5G67400(0.47/0.48*)	AT5G54080(4.55/2.45*)	AT3G28740(2.62/11.74*)
		AT3G56350(0.44/0.11*)	AT1G64940(5.46/5.50*)	AT5G54000(3.17/2.09*)	AT5G38430(0.82/0.69*)	AT4G26010(0.46/0.58*)	AT1G78440(2.11/1.00*)
		AT1G24470(0.47/0.40*)	AT5G54190(1.82/2.60*)	AT2G21890(2.23/2.42*)	AT3G43600(0.46/0.50*)	AT3G46480(0.11/0.13*)	AT2G24190(0.72/0.74*)
		AT1G12010(12.46/23.01*)	AT2G34770(2.47/1.86*)	AT5G64100(2.27/5.21*)	AT3G44560(4.03/4.79*)	AT5G05340(0.20/0.06*)	AT1G64950(102.53/150.78*)
		AT4G20235(2.22/2.14*)	AT1G06640(2.39/0.94*)	AT5G01600(2.40/2.72*)	AT2G46950(0.34/0.26*)	AT3G59890(1.88/1.78*)	AT3G12120(1.81/2.25*)
		AT3G26300(50.20/12.69*)	AT1G65860(0.48/0.17*)	AT5G57220(2.49/1.19*)	AT1G43800(1.64/1.82*)	AT2G30750(2.04/2.02*)	AT2G22330(0.42/0.34*)
		AT3G26060(0.72/0.65*)	AT2G29130(2.65/2.01*)	AT3G44550(0.36/0.30*)	AT4G32810(4.71/3.64*)	AT5G43440(9.42/17.51*)	AT1G04380(0.24/0.89*)
		AT1G55020(0.32/0.60*)	AT5G38420(0.89/0.78*)	AT1G09420(0.32/0.26*)	AT4G12310(2.50/2.77*)	AT2G28860(2.99/0.59*)	AT3G47360(5.91/10.65*)
		AT2G21730(2.05/1.37*)	AT2G18450(0.44/0.43*)	AT3G21770(0.40/0.71*)	AT1G30100(0.48/0.97*)	AT1G31230(0.57/0.48*)	AT2G12190(8.79/9.34*)
		AT2G29090(0.48/0.60*)	AT3G46500(0.21/0.22*)	AT1G06570(2.66/1.84*)	AT2G27010(0.37/0.21*)	AT2G17420(2.25/1.92*)	AT1G64900(0.41/0.83*)
		AT2G29290(2.15/1.22*)	AT2G14100(63.98/5.82*)	AT1G62610(1.19/1.37*)	AT4G19170(2.15/3.66*)	AT3G51240(0.42/0.23*)	AT3G26220(2.15/2.57*)
		AT1G72610(0.38/0.25*)	AT4G12280(0.26/0.14*)	AT1G14120(0.40/0.44*)	AT1G17890(1.45/1.41*)	AT1G18020(1.65/2.40*)	AT5G07990(0.52/0.95*)
		AT2G27690(0.39/0.42*)	AT5G65165(2.13/0.34*)	AT1G54870(6.54/0.07*)	AT3G27060(2.38/1.18*)	AT3G45300(6.18/3.19*)	
**Plant_defense**	**83**	AT1G61190(0.43/0.47*)	AT2G33340(2.23/2.83*)	AT2G02130(0.38/0.39*)	AT3G44400(6.94/10.91*)	AT5G48620(15.99/14.59*)	AT1G19250(8.13/2.01*)
		AT5G40060(1.16/1.03*)	AT1G56520(0.26/0.31*)	AT1G61310(6.16/49.29*)	AT5G46260(9.81/16.45*)	AT1G59218(61.60/117.99*)	AT1G15890(2.21/2.12*)
		AT5G18407(2.16/1.62*)	AT5G05400(2.32/4.71*)	AT4G22230(2.44/0.55*)	AT5G47250(0.27/0.22*)	AT1G50180(0.24/0.35*)	AT2G43550(0.19/0.20*)
		AT4G23130(0.19/0.61*)	AT5G46490(13.93/7.46*)	AT4G19050(0.49/0.43*)	AT5G64890(3.94/0.29*)	AT3G44480(0.34/0.34*)	AT2G40220(4.88/0.68*)
		AT1G61300(0.33/0.36*)	AT5G33355(10.61/47.95*)	AT5G38350(2.46/3.93*)	AT4G16900(1.04/0.85*)	AT5G45490(40.88/33.10*)	AT3G07040(0.50/0.48*)
		AT3G46530(30.77/53.76*)	AT5G25980(13.03/33.60*)	AT4G39030(2.16/0.61*)	AT1G72850(3.14/1.85*)	AT2G43520(0.49/0.63*)	AT5G36930(3.05/2.28*)
		AT4G12020(0.46/0.69*)	AT4G16960(71.96/6.89*)	AT4G16860(1.57/1.40*)	AT1G72840(19.87/16.64*)	AT5G47220(0.15/0.23*)	AT1G75830(4.52/1.08*)
		AT1G58400(5.00/3.77*)	AT5G18360(6.12/3.71*)	AT5G40910(5.49/4.20*)	AT4G22210(4.54/0.95*)	AT5G17890(28.19/28.15*)	AT5G40090(2.62/2.89*)
		AT1G63360(2.36/2.77*)	AT2G31230(0.34/0.55*)	AT5G45510(1.20/1.28*)	AT2G05117(0.04/0.06*)	AT5G48780(2.72/2.51*)	AT3G44630(19.64/13.71*)
		AT3G59930(11.12/97.99*)	AT1G12220(2.01/3.53*)	AT1G58807(92.90/68.68*)	AT1G32860(0.26/0.53*)	AT5G46520(0.26/0.38*)	AT3G44670(0.84/0.77*)
		AT5G46470(2.45/2.31*)	AT4G16950(1.15/1.32*)	AT3G05730(3.07/2.70*)	AT1G59780(3.13/3.29*)	AT1G59124(7.61/11.45*)	AT1G69545(0.47/0.26*)

		AT4G09430(0.48/0.58*)	AT1G63880(23.34/47.41*)	AT5G17880(6.71/10.80*)	AT1G72450(0.14/0.36*)	AT1G58602(4.40/11.24*)	AT5G64905(2.17/1.17*)
		AT5G63020(9.47/8.43*)	AT1G19610(0.36/0.35*)	AT5G36910(0.36/0.53*)	AT5G35450(2.24/2.37*)	AT5G41750(0.22/0.19*)	AT2G43535(4.42/6.34*)
		AT5G43740(8.34/7.08*)	AT1G51480(0.04/0.06*)	AT1G58848(6.64/11.54*)	AT5G43470(49.01/45.79*)	AT5G26000(2.29/1.74*)	
**leucine-rich_repeat**	**74**	AT4G00160(1.01/0.94*)	AT1G61190(0.43/0.47*)	AT1G45616(2.51/3.96*)	AT3G44400(6.94/10.91*)	AT5G48620(15.99/14.59*)	AT1G61310(6.16/49.29*)
		AT5G46260(9.81/16.45*)	AT2G25470(0.11/0.77*)	AT1G17250(0.46/0.47*)	AT1G59218(61.60/117.99*)	AT5G07280(0.47/0.65*)	AT1G15890(2.21/2.12*)
		AT5G05400(2.32/4.71*)	AT5G47250(0.27/0.22*)	AT1G50180(0.24/0.35*)	AT2G40920(3.08/4.76*)	AT5G46490(13.93/7.46*)	AT1G51820(0.38/0.29*)
		AT4G19050(0.49/0.43*)	AT1G06630(2.01/1.71*)	AT3G44480(0.34/0.34*)	AT1G61300(0.33/0.36*)	AT4G16900(1.04/0.85*)	AT4G20940(0.46/0.79*)
		AT3G07040(0.50/0.48*)	AT3G46530(30.77/53.76*)	AT1G78230(0.64/0.57*)	AT5G22660(2.82/2.40*)	AT1G78760(0.27/0.89*)	AT5G36930(3.05/2.28*)
		AT4G12020(0.46/0.69*)	AT5G44980(5.88/5.47*)	AT4G16960(71.96/6.89*)	AT3G12145(0.93/0.68*)	AT4G16860(1.57/1.40*)	AT1G72840(19.87/16.64*)
		AT1G07550(3.13/4.36*)	AT1G58400(5.00/3.77*)	AT5G18360(6.12/3.71*)	AT3G13380(0.43/0.39*)	AT5G40910(5.49/4.20*)	AT2G13790(27.39/16.44*)
		AT1G07560(2.67/2.86*)	AT1G63360(2.36/2.77*)	AT3G52680(9.60/6.63*)	AT5G45510(1.20/1.28*)	AT1G09650(0.50/0.81*)	AT3G49670(0.32/0.53*)
		AT3G13065(0.48/0.40*)	AT2G13800(0.88/0.80*)	AT3G43740(22.70/32.58*)	AT1G80570(2.40/2.17*)	AT5G59680(2.24/3.61*)	AT3G44630(19.64/13.71*)
		AT1G58807(92.90/68.68*)	AT1G12220(2.01/3.53*)	AT3G44670(0.84/0.77*)	AT5G22700(2.33/2.84*)	AT5G46520(0.26/0.38*)	AT5G46470(2.45/2.31*)
		AT4G16950(1.15/1.32*)	AT1G69545(0.47/0.26*)	AT1G59780(3.13/3.29*)	AT1G59124(7.61/11.45*)	AT1G63880(23.34/47.41*)	AT1G17240(2.46/2.45*)
		AT1G58602(4.40/11.24*)	AT5G63020(9.47/8.43*)	AT5G35450(2.24/2.37*)	AT5G43740(8.34/7.08*)	AT1G51480(0.04/0.06*)	AT2G29910(0.42/0.46*)
		AT1G58848(6.64/11.54*)	AT5G43470(49.01/45.79*)				
**glycoprotein**	**124**	AT1G52400(0.10/0.10*)	AT2G23200(0.45/0.62*)	AT4G11320(6.67/12.10*)	AT1G60270(0.27/0.30*)	AT1G73300(1.40/1.28*)	AT5G57530(0.27/0.23*)
		AT2G41510(0.48/0.62*)	AT5G03810(0.29/0.14*)	AT1G28670(6.61/11.17*)	AT5G39160(16.59/25.74*)	AT1G66270(1.29/0.76*)	AT5G39180(2.94/1.38*)
		AT4G27520(0.37/0.45*)	AT4G36880(0.45/3.97*)	AT4G28850(0.96/0.94*)	AT5G40730(1.89/2.73*)	AT1G53920(0.49/0.71*)	AT3G14210(4.68/10.72*)
		AT1G04680(1.74/1.37*)	AT1G28010(0.45/0.69*)	AT4G23220(6.85/5.09*)	AT1G11370(0.05/0.05*)	AT5G46150(2.59/3.18*)	AT4G02330(2.46/2.03*)
		AT2G26440(0.30/0.34*)	AT5G20860(0.39/0.89*)	AT1G73280(0.35/0.31*)	AT4G20940(0.46/0.79*)	AT1G53990(2.48/4.16*)	AT5G25980(13.03/33.60*)
		AT5G54570(0.82/0.70*)	AT5G61350(0.26/0.43*)	AT3G05950(2.20/0.01*)	AT5G39190(68.20/51.10*)	AT4G10310(0.69/0.82*)	AT1G28610(0.41/0.79*)
		AT5G64120(0.46/0.84*)	AT4G10250(2.81/3.89*)	AT2G13790(27.39/16.44*)	AT5G58390(0.48/0.74*)	AT3G47295(0.61/0.63*)	AT4G23210(0.35/0.22*)
		AT2G26450(0.36/0.24*)	AT4G23290(0.12/0.27*)	AT5G55630(1.03/1.24*)	AT2G13800(0.88/0.80*)	AT4G11310(6.44/5.46*)	AT3G45960(1.01/0.68*)
		AT3G28345(0.47/0.94*)	AT5G67400(0.47/0.48*)	AT5G53250(2.18/1.90*)	AT5G20710(39.08/10.61*)	AT3G11210(48.15/29.70*)	AT1G16260(7.77/13.20*)
		AT4G30140(3.54/1.48*)	AT4G31370(0.40/0.55*)	AT3G62740(0.46/0.82*)	AT3G62280(3.88/2.74*)	AT5G26000(2.29/1.74*)	AT5G64100(2.27/5.21*)
		AT2G12480(0.12/0.19*)	AT1G54000(1.16/0.48*)	AT5G05340(0.20/0.06*)	AT4G23320(0.34/0.59*)	AT4G13420(0.20/0.49*)	AT4G23260(0.55/0.41*)
		AT1G76930(2.06/3.56*)	AT3G50400(0.46/0.65*)	AT4G12420(2.97/2.94*)	AT1G16160(2.12/1.92*)	AT2G44470(0.55/1.11*)	AT3G19620(0.19/0.68*)
		AT2G19150(2.65/2.30*)	AT5G45960(0.43/0.45*)	AT4G37450(2.07/1.31*)	AT1G61810(0.54/0.28*)	AT3G12220(2.06/2.25*)	AT1G05200(2.35/2.96*)
		AT2G29130(2.65/2.01*)	AT4G23130(0.19/0.61*)	AT1G31550(0.34/0.39*)	AT1G51820(0.38/0.29*)	AT2G22470(2.46/2.02*)	AT4G27820(1.06/1.79*)
		AT3G04290(0.42/0.12*)	AT3G60140(4.30/3.53*)	AT1G19670(0.31/0.21*)	AT5G39130(9.24/11.30*)	AT3G10450(2.21/2.00*)	AT5G41300(8.09/16.73*)
		AT3G52780(4.27/2.64*)	AT3G09930(0.33/2.52*)	AT3G16370(3.80/2.81*)	AT5G44130(0.39/0.27*)	AT3G59010(0.80/0.85*)	AT2G06850(2.48/1.15*)
		AT1G65240(0.17/0.17*)	AT3G21770(0.40/0.71*)	AT1G07550(3.13/4.36*)	AT3G13380(0.43/0.39*)	AT3G21370(0.44/0.10*)	AT1G07560(2.67/2.86*)
		AT1G26560(0.48/0.72*)	AT5G06390(0.07/0.05*)	AT4G14130(13.87/8.32*)	AT4G30610(0.48/0.36*)	AT1G64390(1.47/1.15*)	AT5G46240(2.17/3.43*)

		AT4G37800(0.25/0.50*)	AT4G30280(2.35/2.12*)	AT3G13065(0.48/0.40*)	AT5G59680(2.24/3.61*)	AT1G32860(0.26/0.53*)	AT2G42990(4.59/4.60*)
		AT2G23130(0.40/0.25*)	AT1G72610(0.38/0.25*)	AT4G24890(0.74/1.18*)	AT1G17890(1.45/1.41*)	AT1G27940(0.05/0.07*)	AT5G39150(2.60/1.08*)
		AT1G43780(0.24/0.15*)	AT4G27300(0.26/0.64*)	AT1G65310(2.77/2.75*)	AT3G14820(0.41/0.58*)		
**Monooxygenase**	**41**	AT5G05320(2.87/3.02*)	AT5G25130(0.37/0.41*)	AT2G35660(0.35/0.33*)	AT2G34770(2.47/1.86*)	AT4G39510(0.31/0.86*)	AT5G42590(2.32/1.38*)
		AT3G30180(3.42/2.29*)	AT1G62540(0.25/0.04*)	AT1G64950(102.53/150.78*)	AT4G13770(0.05/0.06*)	AT1G62560(0.36/1.07*)	AT1G19250(8.13/2.01*)
		AT4G20235(2.22/2.14*)	AT4G37310(0.13/0.16*)	AT4G37430(1.45/0.88*)	AT2G46950(0.34/0.26*)	AT2G12190(8.79/9.34*)	AT2G29090(0.48/0.60*)
		AT2G27010(0.37/0.21*)	AT3G26300(50.20/12.69*)	AT1G65860(0.48/0.17*)	AT1G65340(2.48/1.36*)	AT1G64900(0.41/0.83*)	AT5G57220(2.49/1.19*)
		AT2G30750(2.04/2.02*)	AT2G22330(0.42/0.34*)	AT5G23190(0.47/0.81*)	AT2G14100(63.98/5.82*)	AT3G26220(2.15/2.57*)	AT4G36220(0.28/0.20*)
		AT5G61290(2.31/0.92*)	AT5G38420(0.89/0.78*)	AT5G07990(0.52/0.95*)	AT3G28740(2.62/11.74*)	AT2G27690(0.39/0.42*)	AT4G15300(6.06/3.74*)
		AT1G64940(5.46/5.50*)	AT1G12130(2.00/0.49*)	AT5G38430(0.82/0.69*)	AT4G12310(2.50/2.77*)	AT4G12330(55.28/68.16*)	
**signal**	**158**	AT1G52400(0.10/0.10*)	AT2G23200(0.45/0.62*)	AT5G17820(3.00/1.92*)	AT4G11320(6.67/12.10*)	AT1G60270(0.27/0.30*)	AT3G14220(4.49/7.00*)
		AT1G73300(1.40/1.28*)	AT5G57530(0.27/0.23*)	AT5G24090(2.03/2.62*)	AT2G41510(0.48/0.62*)	AT1G44350(0.35/0.48*)	AT5G07280(0.47/0.65*)
		AT5G03810(0.29/0.14*)	AT4G22230(2.44/0.55*)	AT1G28670(6.61/11.17*)	AT5G39160(16.59/25.74*)	AT1G66270(1.29/0.76*)	AT5G39180(2.94/1.38*)
		AT4G27520(0.37/0.45*)	AT4G36880(0.45/3.97*)	AT4G28850(0.96/0.94*)	AT5G40730(1.89/2.73*)	AT1G53920(0.49/0.71*)	AT3G21900(0.39/0.53*)
		AT3G14210(4.68/10.72*)	AT1G75900(2.80/0.60*)	AT1G04680(1.74/1.37*)	AT4G23220(6.85/5.09*)	AT1G11370(0.05/0.05*)	AT4G02330(2.46/2.03*)
		AT2G26440(0.30/0.34*)	AT5G20860(0.39/0.89*)	AT1G73280(0.35/0.31*)	AT3G22060(0.22/0.23*)	AT4G01630(2.63/1.27*)	AT2G36870(0.40/0.63*)
		AT1G53990(2.48/4.16*)	AT5G25980(13.03/33.60*)	AT5G61350(0.26/0.43*)	AT5G54570(0.82/0.70*)	AT5G13900(0.33/0.94*)	AT3G05950(2.20/0.01*)
		AT5G39190(68.20/51.10*)	AT1G28610(0.41/0.79*)	AT5G64120(0.46/0.84*)	AT4G10250(2.81/3.89*)	AT2G13790(27.39/16.44*)	AT5G58390(0.48/0.74*)
		AT3G47295(0.61/0.63*)	AT4G23210(0.35/0.22*)	AT2G05117(0.04/0.06*)	AT4G23290(0.12/0.27*)	AT2G13800(0.88/0.80*)	AT4G11310(6.44/5.46*)
		AT3G45960(1.01/0.68*)	AT3G05730(3.07/2.70*)	AT3G47300(0.99/0.68*)	AT3G08770(0.35/0.61*)	AT5G67400(0.47/0.48*)	AT5G53250(2.18/1.90*)
		AT5G20710(39.08/10.61*)	AT3G11210(48.15/29.70*)	AT1G16260(7.77/13.20*)	AT4G30140(3.54/1.48*)	AT4G31370(0.40/0.55*)	AT1G19610(0.36/0.35*)
		AT3G62740(0.46/0.82*)	AT4G26010(0.46/0.58*)	AT3G55500(1.00/0.92*)	AT5G01540(0.29/0.41*)	AT3G62280(3.88/2.74*)	AT5G26000(2.29/1.74*)
		AT2G12480(0.12/0.19*)	AT3G05470(0.48/0.66*)	AT5G64100(2.27/5.21*)	AT5G05340(0.20/0.06*)	AT1G54000(1.16/0.48*)	AT2G02130(0.38/0.39*)
		AT4G23320(0.34/0.59*)	AT4G23260(0.55/0.41*)	AT1G05300(0.42/0.60*)	AT1G76930(2.06/3.56*)	AT3G50400(0.46/0.65*)	AT4G12420(2.97/2.94*)
		AT1G16160(2.12/1.92*)	AT2G44470(0.55/1.11*)	AT3G19620(0.19/0.68*)	AT2G19150(2.65/2.30*)	AT5G45960(0.43/0.45*)	AT1G61810(0.54/0.28*)
		AT4G37450(2.07/1.31*)	AT3G12220(2.06/2.25*)	AT5G18407(2.16/1.62*)	AT1G72970(0.59/1.01*)	AT5G05280(0.24/0.91*)	AT1G05200(2.35/2.96*)
		AT3G21920(0.34/0.46*)	AT2G15050(12.90/17.49*)	AT2G29130(2.65/2.01*)	AT2G43550(0.19/0.20*)	AT4G23130(0.19/0.61*)	AT1G31550(0.34/0.39*)
		AT2G22470(2.46/2.02*)	AT1G51820(0.38/0.29*)	AT4G27820(1.06/1.79*)	AT4G27160(0.35/0.14*)	AT3G04290(0.42/0.12*)	AT4G27170(0.35/0.14*)
		AT3G60140(4.30/3.53*)	AT5G33355(10.61/47.95*)	AT5G39130(9.24/11.30*)	AT3G10450(2.21/2.00*)	AT3G21930(0.39/0.47*)	AT5G41300(8.09/16.73*)
		AT3G52780(4.27/2.64*)	AT3G09930(0.33/2.52*)	AT3G16370(3.80/2.81*)	AT2G43520(0.49/0.63*)	AT2G06850(2.48/1.15*)	AT3G59010(0.80/0.85*)
		AT5G44130(0.39/0.27*)	AT1G65240(0.17/0.17*)	AT3G21770(0.40/0.71*)	AT1G07550(3.13/4.36*)	AT1G25054(6.37/6.65*)	AT1G75830(4.52/1.08*)
		AT3G13380(0.43/0.39*)	AT2G19990(2.21/0.57*)	AT3G21370(0.44/0.10*)	AT1G24880(6.03/7.30*)	AT1G07560(2.67/2.86*)	AT4G22210(4.54/0.95*)
		AT2G43860(0.29/0.33*)	AT1G26560(0.48/0.72*)	AT5G06390(0.07/0.05*)	AT4G14130(13.87/8.32*)	AT1G64390(1.47/1.15*)	AT4G30610(0.48/0.36*)
		AT4G37800(0.25/0.50*)	AT4G30280(2.35/2.12*)	AT3G04720(0.44/0.60*)	AT3G13065(0.48/0.40*)	AT5G59680(2.24/3.61*)	AT3G59930(11.12/97.99*)

		AT1G32860(0.26/0.53*)	AT2G42990(4.59/4.60*)	AT2G23130(0.40/0.25*)	AT1G72610(0.38/0.25*)	AT1G02800(0.31/0.86*)	AT4G24890(0.74/1.18*)
		AT1G17890(1.45/1.41*)	AT5G39150(2.60/1.08*)	AT1G03880(0.34/0.07*)	AT5G36910(0.36/0.53*)	AT1G43780(0.24/0.15*)	AT2G43535(4.42/6.34*)
		AT1G65310(2.77/2.75*)	AT3G14820(0.41/0.58*)				
**dioxygenase**	**14**	AT4G32810(4.71/3.64*)	AT4G19170(2.15/3.66*)	AT3G51240(0.42/0.23*)	AT1G55020(0.32/0.60*)	AT1G14120(0.40/0.44*)	AT5G54080(4.55/2.45*)
		AT3G45140(0.06/0.11*)	AT5G54000(3.17/2.09*)	AT1G30100(0.48/0.97*)	AT1G78440(2.11/1.00*)	AT1G06570(2.66/1.84*)	AT5G08640(0.14/0.11*)
		AT5G59540(6.92/12.68*)	AT4G33910(1.89/1.80*)				
**iron**	**74**	AT2G34770(2.47/1.86*)	AT1G80830(2.15/2.43*)	AT5G64100(2.27/5.21*)	AT4G39510(0.31/0.86*)	AT5G17820(3.00/1.92*)	AT5G05340(0.20/0.06*)
		AT3G10520(0.35/0.42*)	AT4G15393(2.06/1.46*)	AT1G64950(102.53/150.78*)	AT2G46650(0.26/0.21*)	AT4G37310(0.13/0.16*)	AT4G20235(2.22/2.14*)
		AT5G01600(2.40/2.72*)	AT1G06640(2.39/0.94*)	AT2G46950(0.34/0.26*)	AT3G26300(50.20/12.69*)	AT5G57220(2.49/1.19*)	AT2G22330(0.42/0.34*)
		AT2G30750(2.04/2.02*)	AT5G23190(0.47/0.81*)	AT4G32810(4.71/3.64*)	AT5G43440(9.42/17.51*)	AT1G04380(0.24/0.89*)	AT1G55020(0.32/0.60*)
		AT4G19210(2.74/1.93*)	AT4G15300(6.06/3.74*)	AT1G06080(2.78/1.25*)	AT4G12310(2.50/2.77*)	AT5G08640(0.14/0.11*)	AT2G28860(2.99/0.59*)
		AT5G25130(0.37/0.41*)	AT5G42590(2.32/1.38*)	AT3G52780(4.27/2.64*)	AT3G30180(3.42/2.29*)	AT3G21770(0.40/0.71*)	AT4G13770(0.05/0.06*)
		AT4G24120(0.48/0.54*)	AT5G64120(0.46/0.84*)	AT3G49620(0.31/0.68*)	AT3G45140(0.06/0.11*)	AT5G58390(0.48/0.74*)	AT4G37430(1.45/0.88*)
		AT2G12190(8.79/9.34*)	AT1G30100(0.48/0.97*)	AT2G29090(0.48/0.60*)	AT3G46500(0.21/0.22*)	AT2G27010(0.37/0.21*)	AT1G06570(2.66/1.84*)
		AT1G65340(2.48/1.36*)	AT5G63600(2.89/2.12*)	AT1G64900(0.41/0.83*)	AT2G14100(63.98/5.82*)	AT4G19170(2.15/3.66*)	AT3G51240(0.42/0.23*)
		AT3G26220(2.15/2.57*)	AT4G36220(0.28/0.20*)	AT4G24890(0.74/1.18*)	AT5G67400(0.47/0.48*)	AT1G14120(0.40/0.44*)	AT5G54080(4.55/2.45*)
		AT5G07990(0.52/0.95*)	AT3G28740(2.62/11.74*)	AT2G27690(0.39/0.42*)	AT5G65165(2.13/0.34*)	AT1G64940(5.46/5.50*)	AT5G54000(3.17/2.09*)
		AT5G45040(0.50/0.55*)	AT3G27060(2.38/1.18*)	AT4G26010(0.46/0.58*)	AT1G78440(2.11/1.00*)	AT3G43600(0.46/0.50*)	AT4G12330(55.28/68.16*)
		AT5G59540(6.92/12.68*)	AT3G46480(0.11/0.13*)				
**Lectin**	**16**	AT1G15530(0.46/0.42*)	AT2G25980(2.08/1.33*)	AT1G53060(0.44/0.45*)	AT5G03350(0.16/0.51*)	AT3G59740(0.35/0.29*)	AT4G28350(0.48/1.17*)
		AT3G16530(0.48/0.97*)	AT5G28520(2.62/2.17*)	AT1G52040(0.27/0.29*)	AT5G54490(0.48/0.75*)	AT3G16410(35.08/44.01*)	AT1G52030(0.25/0.21*)
		AT3G16400(0.53/0.66*)	AT2G33070(0.49/0.56*)	AT3G16390(0.31/0.31*)	AT5G01540(0.29/0.41*)		
**lipid_degradation**	**24**	AT1G53990(2.48/4.16*)	AT3G47290(6.87/6.07*)	AT3G09930(0.33/2.52*)	AT3G16370(3.80/2.81*)	AT1G54000(1.16/0.48*)	AT1G31550(0.34/0.39*)
		AT2G42990(4.59/4.60*)	AT3G14220(4.49/7.00*)	AT1G28610(0.41/0.79*)	AT1G53920(0.49/0.71*)	AT3G50400(0.46/0.65*)	AT3G14210(4.68/10.72*)
		AT3G11210(48.15/29.70*)	AT3G04290(0.42/0.12*)	AT1G75900(2.80/0.60*)	AT4G30140(3.54/1.48*)	AT4G29800(0.47/0.99*)	AT3G47220(3.82/2.44*)
		AT5G45960(0.43/0.45*)	AT4G11850(2.04/2.09*)	AT5G03810(0.29/0.14*)	AT3G14820(0.41/0.58*)	AT1G28670(6.61/11.17*)	AT3G62280(3.88/2.74*)
**glycosidase**	**41**	AT4G26830(0.29/0.65*)	AT2G36870(0.40/0.63*)	AT5G25980(13.03/33.60*)	AT1G52400(0.10/0.10*)	AT2G43620(0.46/0.89*)	AT5G54570(0.82/0.70*)
		AT2G06850(2.48/1.15*)	AT1G60270(0.27/0.30*)	AT5G57530(0.27/0.23*)	AT5G24090(2.03/2.62*)	AT2G43570(2.14/0.90*)	AT5G55180(0.28/0.19*)
		AT3G21370(0.44/0.10*)	AT3G19620(0.19/0.68*)	AT3G04010(2.19/2.39*)	AT2G43860(0.29/0.33*)	AT2G44470(0.55/1.11*)	AT1G26560(0.48/0.72*)
		AT1G58370(2.69/2.71*)	AT1G64390(1.47/1.15*)	AT4G14130(13.87/8.32*)	AT1G61810(0.54/0.28*)	AT4G37800(0.25/0.50*)	AT3G49670(0.32/0.53*)
		AT1G66270(1.29/0.76*)	AT4G30280(2.35/2.12*)	AT4G16260(2.34/2.41*)	AT4G28850(0.96/0.94*)	AT1G32860(0.26/0.53*)	AT1G65570(3.81/0.92*)
		AT1G60590(0.22/0.17*)	AT4G27820(1.06/1.79*)	AT1G02800(0.31/0.86*)	AT5G20710(39.08/10.61*)	AT3G60140(4.30/3.53*)	AT3G62740(0.46/0.82*)
		AT1G65310(2.77/2.75*)	AT3G61490(0.44/0.47*)	AT1G10640(0.39/0.28*)	AT1G61820(0.89/0.75*)	AT5G26000(2.29/1.74*)	
**alternative_splicing**	**135**	AT5G24150(0.37/0.31*)	AT1G52400(0.10/0.10*)	AT1G54040(1.00/0.04*)	AT4G16845(1.14/1.11*)	AT5G44560(2.40/1.72*)	AT1G45249(76.99/43.96*)

		AT4G39260(3.03/2.39*)	AT2G18700(1.42/1.30*)	AT2G22240(0.43/0.40*)	AT4G22230(2.44/0.55*)	AT5G39160(16.59/25.74*)	AT2G23310(1.49/1.76*)
		AT3G49055(0.48/0.58*)	AT5G64520(1.43/1.68*)	AT1G66270(1.29/0.76*)	AT4G18730(0.73/0.91*)	AT5G14740(3.15/3.16*)	AT3G59060(1.66/1.74*)
		AT1G16070(0.40/0.35*)	AT4G01150(0.50/0.53*)	AT5G48720(2.57/1.19*)	AT2G14170(2.18/1.96*)	AT5G61530(3.53/3.29*)	AT4G08870(0.50/0.36*)
		AT5G20730(1.06/1.07*)	AT4G23220(6.85/5.09*)	AT1G68910(2.36/2.11*)	AT1G24490(2.24/2.45*)	AT5G25980(13.03/33.60*)	AT4G05320(2.25/1.94*)
		AT5G22660(2.82/2.40*)	AT5G39190(68.20/51.10*)	AT3G08940(1.53/1.79*)	AT4G12020(0.46/0.69*)	AT1G28610(0.41/0.79*)	AT2G19640(10.94/14.39*)
		AT2G22750(1.81/0.69*)	AT4G33010(0.59/0.43*)	AT1G59640(2.39/2.68*)	AT3G18520(2.12/2.63*)	AT5G20620(0.73/0.68*)	AT4G23210(0.35/0.22*)
		AT1G53790(1.75/3.02*)	AT4G23290(0.12/0.27*)	AT1G80570(2.40/2.17*)	AT5G66400(9.77/0.28*)	AT3G45960(1.01/0.68*)	AT1G80960(6.94/9.51*)
		AT1G59124(7.61/11.45*)	AT1G62800(0.59/0.33*)	AT1G52150(2.63/2.63*)	AT5G65020(2.86/1.44*)	AT1G10060(1.84/2.96*)	AT3G61060(2.06/1.06*)
		AT5G54190(1.82/2.60*)	AT3G56770(3.38/2.54*)	AT3G61160(2.38/1.61*)	AT5G43470(49.01/45.79*)	AT2G24190(0.72/0.74*)	AT5G26000(2.29/1.74*)
		AT2G23240(21.70/0.16*)	AT2G12480(0.12/0.19*)	AT2G33770(2.44/1.91*)	AT2G33340(2.23/2.83*)	AT2G29890(0.47/0.55*)	AT4G13850(0.15/0.15*)
		AT1G05300(0.42/0.60*)	AT1G76930(2.06/3.56*)	AT2G39730(0.48/0.45*)	AT1G24260(0.24/0.35*)	AT1G66610(0.17/0.22*)	AT1G06640(2.39/0.94*)
		AT2G44470(0.55/1.11*)	AT3G59890(1.88/1.78*)	AT2G22540(2.49/4.10*)	AT3G26300(50.20/12.69*)	AT1G10070(3.23/2.09*)	AT3G62090(2.19/2.85*)
		AT5G18407(2.16/1.62*)	AT1G72970(0.59/1.01*)	AT2G03710(40.60/37.25*)	AT1G05200(2.35/2.96*)	AT2G26980(1.51/1.65*)	AT2G15050(12.90/17.49*)
		AT5G28080(0.28/0.45*)	AT5G10140(19.31/28.22*)	AT2G40920(3.08/4.76*)	AT4G23130(0.19/0.61*)	AT2G46450(5.61/8.00*)	AT1G31550(0.34/0.39*)
		AT5G52470(0.96/1.35*)	AT1G17960(2.49/2.98*)	AT4G19850(0.49/0.35*)	AT2G35635(11.46/11.45*)	AT1G23970(2.12/2.99*)	AT3G01500(2.67/2.48*)
		AT5G21170(2.23/1.85*)	AT3G17950(5.47/7.04*)	AT3G46320(0.28/0.26*)	AT2G38620(5.68/2.97*)	AT3G10450(2.21/2.00*)	AT5G62890(0.44/0.46*)
		AT4G04750(0.12/0.39*)	AT3G62030(0.49/0.49*)	AT3G52780(4.27/2.64*)	AT2G44140(1.11/1.13*)	AT2G39470(0.36/0.41*)	AT1G12250(0.45/0.51*)
		AT1G25054(6.37/6.65*)	AT4G21210(0.72/0.75*)	AT5G19210(0.46/0.53*)	AT1G24880(6.03/7.30*)	AT1G06570(2.66/1.84*)	AT5G45510(1.20/1.28*)
		AT5G02500(1.02/0.96*)	AT2G02390(2.06/2.03*)	AT2G46830(1.08/0.88*)	AT1G65390(0.33/0.29*)	AT1G58807(92.90/68.68*)	AT3G48380(2.64/2.89*)
		AT5G48880(0.63/1.07*)	AT3G18500(32.47/71.98*)	AT2G23130(0.40/0.25*)	AT1G17890(1.45/1.41*)	AT1G18020(1.65/2.40*)	AT1G34760(5.45/4.08*)
		AT1G28960(1.05/1.38*)	AT1G65060(1.23/0.93*)	AT5G57655(2.08/1.77*)	AT1G17810(3.96/0.95*)	AT4G21280(0.48/0.58*)	AT2G40430(2.11/2.25*)
		AT5G09820(0.42/0.42*)	AT1G05230(0.49/0.76*)	AT4G29170(2.94/4.39*)			
**lipid_synthesis**	**18**	AT1G43800(1.64/1.82*)	AT3G48530(1.94/1.55*)	AT3G44550(0.36/0.30*)	AT3G44560(4.03/4.79*)	AT1G65290(14.54/9.93*)	AT5G48880(0.63/1.07*)
		AT1G55020(0.32/0.60*)	AT1G25054(6.37/6.65*)	AT5G05580(0.38/0.27*)	AT5G22500(4.94/3.47*)	AT1G24880(6.03/7.30*)	AT3G45140(0.06/0.11*)
		AT5G55360(0.02/0.04*)	AT5G27200(0.14/0.19*)	AT5G21170(2.23/1.85*)	AT3G12120(1.81/2.25*)	AT1G06080(2.78/1.25*)	AT5G55340(0.45/0.62*)
**disulfide_bond**	**85**	AT5G64100(2.27/5.21*)	AT2G12480(0.12/0.19*)	AT1G52400(0.10/0.10*)	AT5G17820(3.00/1.92*)	AT1G45145(0.26/0.32*)	AT2G02130(0.38/0.39*)
		AT5G05340(0.20/0.06*)	AT4G11320(6.67/12.10*)	AT1G60270(0.27/0.30*)	AT1G73300(1.40/1.28*)	AT1G16160(2.12/1.92*)	AT5G57530(0.27/0.23*)
		AT5G24090(2.03/2.62*)	AT2G44470(0.55/1.11*)	AT5G53370(0.46/0.39*)	AT1G61810(0.54/0.28*)	AT3G12220(2.06/2.25*)	AT5G18407(2.16/1.62*)
		AT4G22230(2.44/0.55*)	AT5G39160(16.59/25.74*)	AT3G26060(0.72/0.65*)	AT2G15050(12.90/17.49*)	AT5G39180(2.94/1.38*)	AT1G66270(1.29/0.76*)
		AT4G36880(0.45/3.97*)	AT4G28850(0.96/0.94*)	AT2G43550(0.19/0.20*)	AT4G27820(1.06/1.79*)	AT4G27160(0.35/0.14*)	AT1G60740(3.01/5.18*)
		AT3G51030(0.50/0.36*)	AT4G27170(0.35/0.14*)	AT1G09420(0.32/0.26*)	AT3G60140(4.30/3.53*)	AT5G39130(9.24/11.30*)	AT5G33355(10.61/47.95*)
		AT3G10450(2.21/2.00*)	AT4G02330(2.46/2.03*)	AT2G26440(0.30/0.34*)	AT1G73280(0.35/0.31*)	AT5G20860(0.39/0.89*)	AT2G36870(0.40/0.63*)
		AT5G25980(13.03/33.60*)	AT3G62030(0.49/0.49*)	AT5G54570(0.82/0.70*)	AT3G05950(2.20/0.01*)	AT2G43520(0.49/0.63*)	AT5G39190(68.20/51.10*)
		AT2G06850(2.48/1.15*)	AT3G21770(0.40/0.71*)	AT5G64120(0.46/0.84*)	AT1G75830(4.52/1.08*)	AT5G63030(1.45/1.22*)	AT3G21370(0.44/0.10*)

		AT4G22210(4.54/0.95*)	AT5G58390(0.48/0.74*)	AT1G26560(0.48/0.72*)	AT4G14130(13.87/8.32*)	AT4G30610(0.48/0.36*)	AT1G06570(2.66/1.84*)
		AT2G26450(0.36/0.24*)	AT2G05117(0.04/0.06*)	AT2G17420(2.25/1.92*)	AT4G37800(0.25/0.50*)	AT3G04720(0.44/0.60*)	AT4G30280(2.35/2.12*)
		AT3G59930(11.12/97.99*)	AT4G11310(6.44/5.46*)	AT3G05730(3.07/2.70*)	AT3G47300(0.99/0.68*)	AT1G72610(0.38/0.25*)	AT5G67400(0.47/0.48*)
		AT3G08770(0.35/0.61*)	AT1G17890(1.45/1.41*)	AT5G39150(2.60/1.08*)	AT1G16260(7.77/13.20*)	AT1G19610(0.36/0.35*)	AT1G03880(0.34/0.07*)
		AT5G36910(0.36/0.53*)	AT1G43780(0.24/0.15*)	AT4G26010(0.46/0.58*)	AT1G65310(2.77/2.75*)	AT2G43535(4.42/6.34*)	AT1G04310(2.81/3.80*)
		AT1G61820(0.89/0.75*)					
**heme**	**43**	AT5G25130(0.37/0.41*)	AT2G34770(2.47/1.86*)	AT5G64100(2.27/5.21*)	AT4G39510(0.31/0.86*)	AT5G42590(2.32/1.38*)	AT5G17820(3.00/1.92*)
		AT5G05340(0.20/0.06*)	AT3G30180(3.42/2.29*)	AT3G10520(0.35/0.42*)	AT4G15393(2.06/1.46*)	AT3G21770(0.40/0.71*)	AT1G64950(102.53/150.78*)
		AT4G13770(0.05/0.06*)	AT5G64120(0.46/0.84*)	AT2G46650(0.26/0.21*)	AT4G20235(2.22/2.14*)	AT4G37310(0.13/0.16*)	AT5G58390(0.48/0.74*)
		AT2G12190(8.79/9.34*)	AT2G46950(0.34/0.26*)	AT4G37430(1.45/0.88*)	AT2G29090(0.48/0.60*)	AT2G27010(0.37/0.21*)	AT3G26300(50.20/12.69*)
		AT1G65340(2.48/1.36*)	AT1G64900(0.41/0.83*)	AT5G57220(2.49/1.19*)	AT2G30750(2.04/2.02*)	AT2G22330(0.42/0.34*)	AT5G23190(0.47/0.81*)
		AT2G14100(63.98/5.82*)	AT3G26220(2.15/2.57*)	AT4G36220(0.28/0.20*)	AT5G67400(0.47/0.48*)	AT5G07990(0.52/0.95*)	AT3G28740(2.62/11.74*)
		AT2G27690(0.39/0.42*)	AT4G15300(6.06/3.74*)	AT1G64940(5.46/5.50*)	AT5G45040(0.50/0.55*)	AT4G26010(0.46/0.58*)	AT4G12310(2.50/2.77*)
		AT4G12330(55.28/68.16*)					
**nadp**	**23**	AT2G22330(0.42/0.34*)	AT2G17420(2.25/1.92*)	AT1G43800(1.64/1.82*)	AT3G44550(0.36/0.30*)	AT3G44560(4.03/4.79*)	AT5G39190(68.20/51.10*)
		AT1G72610(0.38/0.25*)	AT1G19250(8.13/2.01*)	AT1G17890(1.45/1.41*)	AT1G18020(1.65/2.40*)	AT5G22500(4.94/3.47*)	AT5G07990(0.52/0.95*)
		AT4G34520(1.06/1.29*)	AT1G09420(0.32/0.26*)	AT1G31230(0.57/0.48*)	AT3G59890(1.88/1.78*)	AT2G29090(0.48/0.60*)	AT2G21890(2.23/2.42*)
		AT5G54190(1.82/2.60*)	AT4G25090(2.11/3.81*)	AT2G21730(2.05/1.37*)	AT1G58290(0.40/0.60*)	AT1G72680(2.16/2.10*)	
**flavonoid_biosynthesis**	**6**	AT5G07990(0.52/0.95*)	AT3G51240(0.42/0.23*)	AT2G47460(0.33/0.23*)	AT3G55120(0.27/0.30*)	AT5G08640(0.14/0.11*)	AT5G13930(0.14/0.10*)
**Flavoprotein**	**18**	AT2G17420(2.25/1.92*)	AT5G24150(0.37/0.31*)	AT2G18450(0.44/0.43*)	AT5G24160(0.22/0.23*)	AT1G48605(0.27/0.45*)	AT1G62540(0.25/0.04*)
		AT1G62560(0.36/1.07*)	AT5G21482(0.44/0.58*)	AT1G19250(8.13/2.01*)	AT1G18020(1.65/2.40*)	AT3G45780(2.57/3.01*)	AT3G30775(0.46/0.17*)
		AT2G41510(0.48/0.62*)	AT3G45300(6.18/3.19*)	AT5G51930(0.31/0.39*)	AT1G72970(0.59/1.01*)	AT1G65860(0.48/0.17*)	AT3G43600(0.46/0.50*)
**Fatty_acid_biosynthesis**	**11**	AT1G43800(1.64/1.82*)	AT5G05580(0.38/0.27*)	AT3G45140(0.06/0.11*)	AT3G48530(1.94/1.55*)	AT5G27200(0.14/0.19*)	AT5G21170(2.23/1.85*)
		AT1G65290(14.54/9.93*)	AT3G12120(1.81/2.25*)	AT1G06080(2.78/1.25*)	AT5G48880(0.63/1.07*)	AT1G55020(0.32/0.60*)	
**metalloprotein**	**22**	AT2G34770(2.47/1.86*)	AT1G64900(0.41/0.83*)	AT2G30750(2.04/2.02*)	AT2G14100(63.98/5.82*)	AT3G52780(4.27/2.64*)	AT3G51240(0.42/0.23*)
		AT3G26220(2.15/2.57*)	AT4G36220(0.28/0.20*)	AT1G64950(102.53/150.78*)	AT4G13770(0.05/0.06*)	AT2G46650(0.26/0.21*)	AT3G28740(2.62/11.74*)
		AT5G07990(0.52/0.95*)	AT3G56350(0.44/0.11*)	AT1G64940(5.46/5.50*)	AT4G37430(1.45/0.88*)	AT2G46950(0.34/0.26*)	AT2G12190(8.79/9.34*)
		AT2G27010(0.37/0.21*)	AT4G12310(2.50/2.77*)	AT3G43600(0.46/0.50*)	AT4G12330(55.28/68.16*)		
**coiled_coil**	**51**	AT1G61190(0.43/0.47*)	AT1G72150(1.14/1.02*)	AT3G47120(2.13/2.44*)	AT5G44560(2.40/1.72*)	AT5G48620(15.99/14.59*)	AT1G24260(0.24/0.35*)
		AT1G73870(0.27/0.75*)	AT1G61310(6.16/49.29*)	AT1G59218(61.60/117.99*)	AT1G13330(2.09/1.95*)	AT5G58550(0.66/0.61*)	AT1G15890(2.21/2.12*)
		AT5G05400(2.32/4.71*)	AT5G47250(0.27/0.22*)	AT1G50180(0.24/0.35*)	AT4G01150(0.50/0.53*)	AT3G28270(0.19/0.15*)	AT3G18480(3.20/3.25*)
		AT1G68910(2.36/2.11*)	AT2G20840(3.11/2.93*)	AT5G45490(40.88/33.10*)	AT1G24490(2.24/2.45*)	AT3G46530(30.77/53.76*)	AT4G39030(2.16/0.61*)
		AT5G20240(2.78/0.92*)	AT3G24890(5.48/7.96*)	AT1G58400(5.00/3.77*)	AT1G63360(2.36/2.77*)	AT2G46340(2.02/2.68*)	AT5G45510(1.20/1.28*)
		AT5G23570(0.22/0.17*)	AT4G27040(0.90/0.96*)	AT1G12220(2.01/3.53*)	AT1G58807(92.90/68.68*)	AT3G28290(126.74/64.20*)	AT5G41620(2.05/3.03*)

		AT1G59780(3.13/3.29*)	AT1G59124(7.61/11.45*)	AT1G52150(2.63/2.63*)	AT1G21810(0.36/0.43*)	AT1G58602(4.40/11.24*)	AT5G63020(9.47/8.43*)
		AT5G35450(2.24/2.37*)	AT5G43740(8.34/7.08*)	AT1G51480(0.04/0.06*)	AT1G05230(0.49/0.76*)	AT1G58848(6.64/11.54*)	AT1G01670(0.47/0.76*)
		AT4G29170(2.94/4.39*)	AT5G43470(49.01/45.79*)	AT5G26980(8.29/6.75*)			
**FAD**	**15**	AT5G24150(0.37/0.31*)	AT2G17420(2.25/1.92*)	AT2G18450(0.44/0.43*)	AT5G24160(0.22/0.23*)	AT1G62540(0.25/0.04*)	AT1G62560(0.36/1.07*)
		AT5G21482(0.44/0.58*)	AT1G19250(8.13/2.01*)	AT3G30775(0.46/0.17*)	AT2G41510(0.48/0.62*)	AT3G45300(6.18/3.19*)	AT4G25090(2.11/3.81*)
		AT1G72970(0.59/1.01*)	AT1G65860(0.48/0.17*)	AT3G43600(0.46/0.50*)			
**apoplast**	**17**	AT2G36870(0.40/0.63*)	AT4G37800(0.25/0.50*)	AT2G29130(2.65/2.01*)	AT5G39180(2.94/1.38*)	AT4G30280(2.35/2.12*)	AT4G28850(0.96/0.94*)
		AT3G05950(2.20/0.01*)	AT5G39190(68.20/51.10*)	AT2G06850(2.48/1.15*)	AT1G72610(0.38/0.25*)	AT5G57530(0.27/0.23*)	AT1G17890(1.45/1.41*)
		AT5G20710(39.08/10.61*)	AT5G39150(2.60/1.08*)	AT4G14130(13.87/8.32*)	AT5G39130(9.24/11.30*)	AT1G65310(2.77/2.75*)	
**Secreted**	**91**	AT5G64100(2.27/5.21*)	AT2G12480(0.12/0.19*)	AT5G17820(3.00/1.92*)	AT2G02130(0.38/0.39*)	AT1G54000(1.16/0.48*)	AT5G05340(0.20/0.06*)
		AT1G76930(2.06/3.56*)	AT1G73300(1.40/1.28*)	AT3G50400(0.46/0.65*)	AT3G14220(4.49/7.00*)	AT4G12420(2.97/2.94*)	AT5G57530(0.27/0.23*)
		AT5G24090(2.03/2.62*)	AT3G19620(0.19/0.68*)	AT2G19150(2.65/2.30*)	AT5G45960(0.43/0.45*)	AT5G03810(0.29/0.14*)	AT5G18407(2.16/1.62*)
		AT3G12220(2.06/2.25*)	AT4G22230(2.44/0.55*)	AT1G28670(6.61/11.17*)	AT5G39160(16.59/25.74*)	AT3G21920(0.34/0.46*)	AT2G29130(2.65/2.01*)
		AT5G39180(2.94/1.38*)	AT4G28850(0.96/0.94*)	AT2G43550(0.19/0.20*)	AT1G31550(0.34/0.39*)	AT1G53920(0.49/0.71*)	AT3G14210(4.68/10.72*)
		AT3G21900(0.39/0.53*)	AT3G04290(0.42/0.12*)	AT1G75900(2.80/0.60*)	AT5G39130(9.24/11.30*)	AT5G33355(10.61/47.95*)	AT1G11370(0.05/0.05*)
		AT3G10450(2.21/2.00*)	AT4G02330(2.46/2.03*)	AT2G26440(0.30/0.34*)	AT3G21930(0.39/0.47*)	AT1G73280(0.35/0.31*)	AT5G20860(0.39/0.89*)
		AT3G22060(0.22/0.23*)	AT4G01630(2.63/1.27*)	AT2G36870(0.40/0.63*)	AT1G53990(2.48/4.16*)	AT3G09930(0.33/2.52*)	AT3G52780(4.27/2.64*)
		AT3G16370(3.80/2.81*)	AT3G05950(2.20/0.01*)	AT2G43520(0.49/0.63*)	AT3G59010(0.80/0.85*)	AT5G39190(68.20/51.10*)	AT2G06850(2.48/1.15*)
		AT3G21770(0.40/0.71*)	AT1G28610(0.41/0.79*)	AT5G64120(0.46/0.84*)	AT1G75830(4.52/1.08*)	AT4G22210(4.54/0.95*)	AT5G58390(0.48/0.74*)
		AT2G43860(0.29/0.33*)	AT5G06390(0.07/0.05*)	AT4G14130(13.87/8.32*)	AT1G64390(1.47/1.15*)	AT4G30610(0.48/0.36*)	AT3G47295(0.61/0.63*)
		AT2G05117(0.04/0.06*)	AT4G37800(0.25/0.50*)	AT4G30280(2.35/2.12*)	AT3G59930(11.12/97.99*)	AT1G32860(0.26/0.53*)	AT2G42990(4.59/4.60*)
		AT3G05730(3.07/2.70*)	AT3G45960(1.01/0.68*)	AT1G02800(0.31/0.86*)	AT1G72610(0.38/0.25*)	AT4G24890(0.74/1.18*)	AT5G67400(0.47/0.48*)
		AT1G17890(1.45/1.41*)	AT5G20710(39.08/10.61*)	AT3G11210(48.15/29.70*)	AT5G39150(2.60/1.08*)	AT4G30140(3.54/1.48*)	AT1G19610(0.36/0.35*)
		AT5G36910(0.36/0.53*)	AT1G43780(0.24/0.15*)	AT4G26010(0.46/0.58*)	AT1G65310(2.77/2.75*)	AT2G43535(4.42/6.34*)	AT3G55500(1.00/0.92*)
		AT3G14820(0.41/0.58*)					
**transferase**	**139**	AT3G07020(0.33/0.39*)	AT1G58080(0.32/0.37*)	AT2G23200(0.45/0.62*)	AT5G37950(0.43/0.26*)	AT2G14255(2.65/3.32*)	AT2G32530(0.40/0.62*)
		AT3G19710(0.31/0.05*)	AT1G60940(2.38/2.77*)	AT5G57530(0.27/0.23*)	AT5G23010(0.76/0.44*)	AT5G03490(3.90/5.30*)	AT1G02920(3.39/2.19*)
		AT3G29670(6.85/5.09*)	AT5G17220(2.21/2.03*)	AT5G07280(0.47/0.65*)	AT2G18700(1.42/1.30*)	AT3G63110(0.49/0.70*)	AT5G55340(0.45/0.62*)
		AT4G17360(0.44/0.26*)	AT1G72680(2.16/2.10*)	AT1G10210(2.66/2.48*)	AT1G78270(1.02/1.63*)	AT4G28850(0.96/0.94*)	AT1G17050(0.48/0.60*)
		AT1G78320(0.17/0.16*)	AT5G51690(81.05/183.70*)	AT3G02020(0.24/0.05*)	AT4G23220(6.85/5.09*)	AT5G13930(0.14/0.10*)	AT4G20940(0.46/0.79*)
		AT2G01450(1.99/1.29*)	AT2G36870(0.40/0.63*)	AT5G37300(2.81/1.37*)	AT5G47980(0.25/0.80*)	AT5G61350(0.26/0.43*)	AT5G20830(1.16/0.96*)
		AT4G23600(0.15/0.02*)	AT4G12020(0.46/0.69*)	AT1G06520(0.38/0.38*)	AT2G19640(10.94/14.39*)	AT2G13790(27.39/16.44*)	AT5G12210(2.50/2.81*)
		AT4G23210(0.35/0.22*)	AT2G46340(2.02/2.68*)	AT4G02390(2.15/1.02*)	AT1G77060(3.27/8.23*)	AT3G26840(0.24/0.11*)	AT4G23290(0.12/0.27*)
		AT2G29460(0.36/0.33*)	AT2G13800(0.88/0.80*)	AT5G43910(2.25/2.17*)	AT3G03190(0.41/0.12*)	AT3G28340(0.53/0.51*)	AT1G48260(2.01/2.37*)

		AT1G73880(2.22/5.45*)	AT1G62800(0.59/0.33*)	AT1G31910(4.13/3.65*)	AT5G43780(0.84/0.67*)	AT4G04670(2.25/2.76*)	AT1G16260(7.77/13.20*)
		AT3G32040(0.49/0.48*)	AT1G78340(2.19/4.97*)	AT3G07800(2.54/0.93*)	AT1G10060(1.84/2.96*)	AT3G61160(2.38/1.61*)	AT1G01390(3.10/7.50*)
		AT1G04310(2.81/3.80*)	AT1G10370(0.44/0.38*)	AT5G01540(0.29/0.41*)	AT1G09240(0.44/0.55*)	AT5G17050(0.44/0.42*)	AT5G14470(2.19/1.81*)
		AT4G23320(0.34/0.59*)	AT3G29430(0.21/0.09*)	AT4G23260(0.55/0.41*)	AT1G16160(2.12/1.92*)	AT2G26420(0.42/0.49*)	AT1G13430(11.44/33.42*)
		AT1G10070(3.23/2.09*)	AT2G31790(0.34/0.39*)	AT2G26980(1.51/1.65*)	AT5G28080(0.28/0.45*)	AT3G16520(0.67/1.04*)	AT4G23130(0.19/0.61*)
		AT5G41820(0.20/0.10*)	AT4G01770(3.07/6.16*)	AT5G52470(0.96/1.35*)	AT3G23630(2.20/3.29*)	AT1G51820(0.38/0.29*)	AT2G29480(2.87/2.48*)
		AT1G04640(0.38/0.45*)	AT4G15480(0.11/0.09*)	AT1G05560(2.27/2.01*)	AT5G19220(2.38/2.29*)	AT4G27560(1.56/2.07*)	AT5G62480(3.61/2.47*)
		AT2G38620(5.68/2.97*)	AT5G43760(1.75/1.35*)	AT2G30140(0.28/0.40*)	AT2G36770(2.32/0.96*)	AT3G53150(2.46/1.61*)	AT3G59480(3.42/3.10*)
		AT2G06850(2.48/1.15*)	AT1G21100(0.62/0.89*)	AT1G07550(3.13/4.36*)	AT4G21210(0.72/0.75*)	AT3G13380(0.43/0.39*)	AT3G27440(5.17/4.29*)
		AT2G29730(2.10/2.02*)	AT5G55360(0.02/0.04*)	AT3G14530(3.94/3.86*)	AT1G07560(2.67/2.86*)	AT4G34520(1.06/1.29*)	AT3G45780(2.57/3.01*)
		AT5G38010(0.48/0.40*)	AT1G31230(0.57/0.48*)	AT4G14130(13.87/8.32*)	AT3G11240(2.94/2.63*)	AT4G04740(1.14/1.10*)	AT1G78370(0.18/0.18*)
		AT2G02500(0.49/0.44*)	AT2G02390(2.06/2.03*)	AT4G37800(0.25/0.50*)	AT4G30280(2.35/2.12*)	AT1G79000(3.20/3.85*)	AT5G59680(2.24/3.61*)
		AT5G48880(0.63/1.07*)	AT1G02930(2.98/2.00*)	AT2G23740(0.45/0.72*)	AT1G21110(0.44/0.60*)	AT3G21560(0.21/0.33*)	AT2G30360(1.77/1.10*)
		AT1G65310(2.77/2.75*)	AT1G05680(2.24/4.32*)	AT5G53970(2.56/2.21*)	AT5G41040(0.43/0.59*)	AT3G51630(0.99/1.02*)	AT2G16890(0.03/0.03*)
		AT5G23020(0.08/0.17*)					
**cysteine_proteinase**	**5**	AT3G48350(0.42/0.38*)	AT4G36880(0.45/3.97*)	AT4G11310(6.44/5.46*)	AT4G16190(2.33/1.87*)	AT4G11320(6.67/12.10*)	
**cell_wall**	**20**	AT2G36870(0.40/0.63*)	AT4G37800(0.25/0.50*)	AT4G30280(2.35/2.12*)	AT4G28850(0.96/0.94*)	AT2G06850(2.48/1.15*)	AT3G59010(0.80/0.85*)
		AT1G32860(0.26/0.53*)	AT1G76930(2.06/3.56*)	AT4G12420(2.97/2.94*)	AT5G57530(0.27/0.23*)	AT2G43860(0.29/0.33*)	AT2G19150(2.65/2.30*)
		AT4G14130(13.87/8.32*)	AT1G65310(2.77/2.75*)	AT3G55500(1.00/0.92*)	AT1G11370(0.05/0.05*)	AT5G20860(0.39/0.89*)	AT4G02330(2.46/2.03*)
		AT2G26440(0.30/0.34*)	AT4G01630(2.63/1.27*)				
**hydrolase**	**131**	AT1G49630(2.10/2.92*)	AT1G04110(0.21/0.48*)	AT1G52400(0.10/0.10*)	AT4G11320(6.67/12.10*)	AT2G32150(2.02/1.46*)	AT3G52500(0.46/0.45*)
		AT1G60270(0.27/0.30*)	AT1G49050(2.31/1.57*)	AT1G73300(1.40/1.28*)	AT3G14220(4.49/7.00*)	AT5G57530(0.27/0.23*)	AT5G24090(2.03/2.62*)
		AT5G09650(0.53/0.49*)	AT3G27730(0.35/0.54*)	AT1G31190(0.54/0.60*)	AT1G44350(0.35/0.48*)	AT2G28040(0.43/0.68*)	AT4G11850(2.04/2.09*)
		AT5G03810(0.29/0.14*)	AT1G28670(6.61/11.17*)	AT1G66270(1.29/0.76*)	AT4G36880(0.45/3.97*)	AT4G28850(0.96/0.94*)	AT4G16260(2.34/2.41*)
		AT4G20070(0.17/0.18*)	AT5G50260(7.95/4.15*)	AT1G53920(0.49/0.71*)	AT3G14210(4.68/10.72*)	AT1G75900(2.80/0.60*)	AT4G08870(0.50/0.36*)
		AT1G11370(0.05/0.05*)	AT4G02330(2.46/2.03*)	AT2G26440(0.30/0.34*)	AT5G20860(0.39/0.89*)	AT1G73280(0.35/0.31*)	AT2G36870(0.40/0.63*)
		AT4G26830(0.29/0.65*)	AT1G53990(2.48/4.16*)	AT5G26860(0.43/0.52*)	AT5G25980(13.03/33.60*)	AT2G43620(0.46/0.89*)	AT2G07750(0.49/0.63*)
		AT5G54570(0.82/0.70*)	AT1G28610(0.41/0.79*)	AT1G20160(4.11/2.11*)	AT2G43570(2.14/0.90*)	AT3G18520(2.12/2.63*)	AT5G51760(2.16/0.54*)
		AT2G26450(0.36/0.24*)	AT2G04530(0.44/0.57*)	AT3G47290(6.87/6.07*)	AT3G47010(2.89/2.33*)	AT4G11310(6.44/5.46*)	AT4G21650(0.27/0.73*)
		AT1G70170(2.08/2.50*)	AT5G20710(39.08/10.61*)	AT3G11210(48.15/29.70*)	AT4G30140(3.54/1.48*)	AT3G62740(0.46/0.82*)	AT3G62280(3.88/2.74*)
		AT5G26000(2.29/1.74*)	AT2G12480(0.12/0.19*)	AT3G48350(0.42/0.38*)	AT1G51380(0.41/0.47*)	AT1G54000(1.16/0.48*)	AT4G16190(2.33/1.87*)
		AT2G27420(0.22/1.07*)	AT3G50400(0.46/0.65*)	AT5G55180(0.28/0.19*)	AT5G53370(0.46/0.39*)	AT2G44470(0.55/1.11*)	AT3G19620(0.19/0.68*)
		AT2G19150(2.65/2.30*)	AT1G58370(2.69/2.71*)	AT5G45960(0.43/0.45*)	AT1G61810(0.54/0.28*)	AT3G12220(2.06/2.25*)	AT1G31550(0.34/0.39*)
		AT2G47800(0.37/0.47*)	AT1G47380(2.56/2.34*)	AT1G65570(3.81/0.92*)	AT1G60590(0.22/0.17*)	AT4G27820(1.06/1.79*)	AT3G04290(0.42/0.12*)

		AT3G60140(4.30/3.53*)	AT1G19670(0.31/0.21*)	AT3G47220(3.82/2.44*)	AT3G13090(0.40/0.23*)	AT3G61490(0.44/0.47*)	AT3G10450(2.21/2.00*)
		AT3G09930(0.33/2.52*)	AT3G16370(3.80/2.81*)	AT3G59010(0.80/0.85*)	AT2G06850(2.48/1.15*)	AT2G44140(1.11/1.13*)	AT1G65240(0.17/0.17*)
		AT1G25054(6.37/6.65*)	AT3G47040(2.01/1.62*)	AT5G19210(0.46/0.53*)	AT3G21370(0.44/0.10*)	AT1G24880(6.03/7.30*)	AT4G37270(26.36/27.90*)
		AT3G04010(2.19/2.39*)	AT2G43860(0.29/0.33*)	AT1G26560(0.48/0.72*)	AT4G14130(13.87/8.32*)	AT4G30610(0.48/0.36*)	AT1G64390(1.47/1.15*)
		AT1G34750(2.05/1.98*)	AT5G24410(2.68/1.84*)	AT3G44260(2.52/1.73*)	AT4G37800(0.25/0.50*)	AT3G49670(0.32/0.53*)	AT3G26690(2.39/3.34*)
		AT4G30280(2.35/2.12*)	AT5G17780(0.30/0.71*)	AT2G22310(3.65/3.48*)	AT3G48380(2.64/2.89*)	AT1G32860(0.26/0.53*)	AT1G79330(0.19/0.14*)
		AT3G18500(32.47/71.98*)	AT2G42990(4.59/4.60*)	AT1G02800(0.31/0.86*)	AT1G28960(1.05/1.38*)	AT4G29800(0.47/0.99*)	AT1G43780(0.24/0.15*)
		AT1G65310(2.77/2.75*)	AT1G79890(0.40/0.42*)	AT3G14820(0.41/0.58*)	AT1G10640(0.39/0.28*)	AT1G61820(0.89/0.75*)	
**Pyrrolidone_carboxylic_acid**	**9**	AT5G17820(3.00/1.92*)	AT5G58390(0.48/0.74*)	AT4G26010(0.46/0.58*)	AT4G37450(2.07/1.31*)	AT2G22470(2.46/2.02*)	AT3G21770(0.40/0.71*)
		AT2G23130(0.40/0.25*)	AT5G67400(0.47/0.48*)	AT1G75830(4.52/1.08*)			
**stress_response**	**21**	AT5G51440(5.34/13.25*)	AT5G15960(2.09/0.59*)	AT5G15970(3.49/0.90*)	AT5G66400(9.77/0.28*)	AT1G48605(0.27/0.45*)	AT1G02930(2.98/2.00*)
		AT2G04030(0.55/0.86*)	AT5G62490(4.07/0.52*)	AT3G22830(0.37/0.83*)	AT2G19310(2.20/3.98*)	AT4G10250(2.81/3.89*)	AT3G61890(3.11/3.32*)
		AT4G25480(2.15/2.67*)	AT4G13980(2.19/2.45*)	AT2G40220(4.88/0.68*)	AT3G30775(0.46/0.17*)	AT4G25490(2.44/1.68*)	AT2G29090(0.48/0.60*)
		AT1G66390(0.24/0.40*)	AT5G13550(2.05/1.74*)	AT5G02500(1.02/0.96*)			
**thiol_protease**	**11**	AT3G48350(0.42/0.38*)	AT4G36880(0.45/3.97*)	AT4G11310(6.44/5.46*)	AT4G16190(2.33/1.87*)	AT2G22310(3.65/3.48*)	AT3G48380(2.64/2.89*)
		AT4G11320(6.67/12.10*)	AT5G50260(7.95/4.15*)	AT2G44140(1.11/1.13*)	AT1G79330(0.19/0.14*)	AT2G27420(0.22/1.07*)	
**lyase**	**21**	AT1G16540(2.12/2.11*)	AT3G23490(2.71/1.96*)	AT2G23590(0.47/0.83*)	AT5G04310(2.01/2.57*)	AT1G48605(0.27/0.45*)	AT3G11750(0.44/0.41*)
		AT5G14740(3.15/3.16*)	AT5G38420(0.89/0.78*)	AT4G26530(3.13/2.10*)	AT1G52410(0.44/0.11*)	AT1G04680(1.74/1.37*)	AT3G01500(2.67/2.48*)
		AT1G08250(0.38/0.33*)	AT5G38430(0.82/0.69*)	AT4G13280(0.47/0.54*)	AT4G37150(0.44/0.47*)	AT5G04230(0.46/0.40*)	AT5G51930(0.31/0.39*)
		AT5G15950(0.29/0.46*)	AT3G15620(4.14/4.19*)	AT4G27070(0.34/0.23*)			
**atp-binding**	**150**	AT2G23200(0.45/0.62*)	AT3G44400(6.94/10.91*)	AT1G60940(2.38/2.77*)	AT1G56520(0.26/0.31*)	AT1G33770(2.01/2.73*)	AT4G08470(0.48/0.54*)
		AT2G18193(19.28/20.80*)	AT3G27730(0.35/0.54*)	AT5G46260(9.81/16.45*)	AT5G07280(0.47/0.65*)	AT1G73500(1.72/3.80*)	AT3G63110(0.49/0.70*)
		AT1G10210(2.66/2.48*)	AT5G49780(2.46/2.01*)	AT1G73860(0.16/1.03*)	AT5G45930(0.46/0.62*)	AT4G15233(0.41/0.34*)	AT4G19210(2.74/1.93*)
		AT4G15236(0.37/0.39*)	AT1G28010(0.45/0.69*)	AT3G44480(0.34/0.34*)	AT3G02020(0.24/0.05*)	AT4G23220(6.85/5.09*)	AT4G20940(0.46/0.79*)
		AT5G45490(40.88/33.10*)	AT2G01450(1.99/1.29*)	AT3G07040(0.50/0.48*)	AT3G46530(30.77/53.76*)	AT5G26860(0.43/0.52*)	AT2G07750(0.49/0.63*)
		AT5G61350(0.26/0.43*)	AT4G16960(71.96/6.89*)	AT4G12020(0.46/0.69*)	AT4G16860(1.57/1.40*)	AT1G53780(2.34/2.85*)	AT5G40910(5.49/4.20*)
		AT2G13790(27.39/16.44*)	AT5G40090(2.62/2.89*)	AT1G63360(2.36/2.77*)	AT3G55130(0.45/0.68*)	AT4G23210(0.35/0.22*)	AT2G46340(2.02/2.68*)
		AT3G48530(1.94/1.55*)	AT4G23290(0.12/0.27*)	AT3G44730(0.41/0.30*)	AT2G13800(0.88/0.80*)	AT3G44630(19.64/13.71*)	AT5G45430(36.85/29.20*)
		AT1G12220(2.01/3.53*)	AT5G46470(2.45/2.31*)	AT4G16950(1.15/1.32*)	AT1G48260(2.01/2.37*)	AT3G28540(0.33/0.33*)	AT3G28345(0.47/0.94*)
		AT1G69545(0.47/0.26*)	AT1G59124(7.61/11.45*)	AT1G31910(4.13/3.65*)	AT1G63880(23.34/47.41*)	AT1G16260(7.77/13.20*)	AT3G07800(2.54/0.93*)
		AT5G35450(2.24/2.37*)	AT5G02270(0.50/0.57*)	AT5G41750(0.22/0.19*)	AT5G24080(0.47/0.98*)	AT4G34030(2.10/1.56*)	AT3G61160(2.38/1.61*)
		AT1G04310(2.81/3.80*)	AT5G01540(0.29/0.41*)	AT5G43470(49.01/45.79*)	AT1G58848(6.64/11.54*)	AT1G61190(0.43/0.47*)	AT2G33770(2.44/1.91*)
		AT1G51380(0.41/0.47*)	AT5G14470(2.19/1.81*)	AT4G23320(0.34/0.59*)	AT4G23260(0.55/0.41*)	AT2G39730(0.48/0.45*)	AT5G48620(15.99/14.59*)
		AT2G26420(0.42/0.49*)	AT1G16160(2.12/1.92*)	AT5G40060(1.16/1.03*)	AT4G34500(4.40/4.63*)	AT5G64050(0.41/0.39*)	AT1G61310(6.16/49.29*)

		AT1G59218(61.60/117.99*)	AT4G11890(2.05/0.52*)	AT1G15890(2.21/2.12*)	AT5G05400(2.32/4.71*)	AT1G18350(0.42/0.44*)	AT5G47250(0.27/0.22*)
		AT2G26980(1.51/1.65*)	AT5G28080(0.28/0.45*)	AT3G55110(0.40/0.69*)	AT1G50180(0.24/0.35*)	AT1G15530(0.46/0.42*)	AT4G23130(0.19/0.61*)
		AT5G46490(13.93/7.46*)	AT2G47800(0.37/0.47*)	AT3G23630(2.20/3.29*)	AT1G17960(2.49/2.98*)	AT1G51820(0.38/0.29*)	AT1G66920(0.23/0.45*)
		AT2G25840(0.49/0.54*)	AT5G38210(0.43/0.62*)	AT3G59740(0.35/0.29*)	AT4G19050(0.49/0.43*)	AT5G19220(2.38/2.29*)	AT5G21170(2.23/1.85*)
		AT1G61300(0.33/0.36*)	AT3G13090(0.40/0.23*)	AT2G38620(5.68/2.97*)	AT5G38350(2.46/3.93*)	AT3G59480(3.42/3.10*)	AT3G47760(0.73/2.24*)
		AT4G28350(0.48/1.17*)	AT1G07550(3.13/4.36*)	AT1G58400(5.00/3.77*)	AT4G21210(0.72/0.75*)	AT3G13380(0.43/0.39*)	AT5G18360(6.12/3.71*)
		AT5G19210(0.46/0.53*)	AT3G27440(5.17/4.29*)	AT4G37270(26.36/27.90*)	AT5G17890(28.19/28.15*)	AT1G07560(2.67/2.86*)	AT3G45780(2.57/3.01*)
		AT1G31230(0.57/0.48*)	AT4G04740(1.14/1.10*)	AT5G45510(1.20/1.28*)	AT5G02500(1.02/0.96*)	AT3G13065(0.48/0.40*)	AT5G59680(2.24/3.61*)
		AT1G58807(92.90/68.68*)	AT5G52860(0.38/0.95*)	AT3G51240(0.42/0.23*)	AT3G44670(0.84/0.77*)	AT5G46520(0.26/0.38*)	AT1G59780(3.13/3.29*)
		AT5G43320(1.22/1.06*)	AT1G27940(0.05/0.07*)	AT1G65060(1.23/0.93*)	AT1G58602(4.40/11.24*)	AT3G59350(2.12/1.67*)	AT5G63020(9.47/8.43*)
		AT2G30360(1.77/1.10*)	AT1G64110(1.97/0.93*)	AT1G79890(0.40/0.42*)	AT1G51480(0.04/0.06*)	AT3G51630(0.99/1.02*)	AT5G43740(8.34/7.08*)
**proteoglycan**	**8**	AT4G31370(0.40/0.55*)	AT5G06390(0.07/0.05*)	AT5G44130(0.39/0.27*)	AT5G40730(1.89/2.73*)	AT4G37450(2.07/1.31*)	AT2G22470(2.46/2.02*)
		AT2G23130(0.40/0.25*)	AT5G53250(2.18/1.90*)				
**lipid-binding**	**8**	AT2G44290(2.56/3.65*)	AT2G15050(12.90/17.49*)	AT5G13900(0.33/0.94*)	AT1G72150(1.14/1.02*)	AT2G44300(2.36/3.27*)	AT4G09160(1.64/2.62*)
		AT3G08770(0.35/0.61*)	AT3G13380(0.43/0.39*)				
**lipid_metabolism**	**6**	AT5G22500(4.94/3.47*)	AT3G44550(0.36/0.30*)	AT3G44560(4.03/4.79*)	AT5G55360(0.02/0.04*)	AT5G48880(0.63/1.07*)	AT5G55340(0.45/0.62*)
**seed**	**3**	AT4G27170(0.35/0.14*)	AT1G03880(0.34/0.07*)	AT4G27160(0.35/0.14*)			
**Seed_storage_protein**	**3**	AT4G27170(0.35/0.14*)	AT1G03880(0.34/0.07*)	AT4G27160(0.35/0.14*)			
