# Supplementary material for: Far-Red Light-Mediated Seedling Development in Arabidopsis Involves FAR-RED INSENSITIVE 219/JASMONATE RESISTANT 1-Dependent and -Independent Pathways
Source: PLoS One. 2015 Jul 15;10(7):e0132723. doi: 10.1371/journal.pone.0132723 (PMC4503420; doi:10.1371/journal.pone.0132723)
Supplement: S5 Table — (PDF) [file pone.0132723.s013.pdf]

S5 Table. Gene list and gene expression in *fin219-2*/Col in the presence of 50 µM MeJA.

GO Term	Count	Gene list (Expression ratio: without MeJA/ with MeJA*) in *fin219-2*/Col (50 µM MeJA)					
oxidoreductase	200	AT5G24150(0.37/0.31*)	AT4G38540(1.34/1.72*)	AT1G59900(12.08/12.15*)	AT1G19250(8.13/2.01*)	AT3G61220(0.41/0.39*)	AT4G33790(0.86/0.45*)
		AT1G49570(1.42/0.28*)	AT1G72680(2.16/2.10*)	AT5G42580(0.97/0.35*)	AT1G63460(3.45/3.29*)	AT5G24160(0.22/0.23*)	AT1G32780(0.60/0.45*)
		AT2G07727(0.60/0.40*)	AT3G48320(0.68/0.45*)	AT1G60740(3.01/5.18*)	AT3G59710(1.61/2.34*)	AT1G16400(0.67/0.25*)	AT4G25090(2.11/3.81*)
		AT3G26330(0.84/0.22*)	AT5G08640(0.14/0.11*)	AT5G02540(1.03/0.78*)	AT5G25130(0.37/0.41*)	AT2G37540(0.32/0.21*)	AT3G30180(3.42/2.29*)
		AT3G61400(0.66/0.14*)	AT1G34510(0.94/0.48*)	AT3G45140(0.06/0.11*)	AT5G05260(1.03/0.44*)	AT5G24070(0.81/0.43*)	AT1G14540(1.29/0.44*)
		AT5G63600(2.89/2.12*)	AT5G25120(0.50/0.43*)	AT5G40610(1.90/2.09*)	AT4G36220(0.28/0.20*)	AT2G38240(0.98/0.14*)	AT4G27440(1.24/2.26*)
		AT1G30040(0.87/0.94*)	AT5G54000(3.17/2.09*)	AT5G38430(0.82/0.69*)	AT1G24470(0.47/0.40*)	AT2G36690(1.48/4.23*)	AT1G12010(12.46/23.01*)
		AT3G46490(0.96/0.23*)	AT5G14200(0.56/0.41*)	AT5G06730(0.57/0.34*)	AT3G51680(0.94/2.08*)	AT4G18350(0.58/0.47*)	AT1G64950(102.53/150.78*)
		AT1G48130(1.71/0.41*)	AT5G05690(1.92/2.08*)	AT1G78490(1.20/0.29*)	AT4G20235(2.22/2.14*)	AT2G46950(0.34/0.26*)	AT5G59530(1.74/4.99*)
		AT3G26300(50.20/12.69*)	AT1G65860(0.48/0.17*)	AT3G01900(0.58/0.24*)	AT2G30750(2.04/2.02*)	AT1G43800(1.64/1.82*)	AT3G26060(0.72/0.65*)
		AT2G29130(2.65/2.01*)	AT1G18140(0.61/0.14*)	AT3G10920(0.97/0.92*)	AT2G25160(0.88/0.39*)	AT1G14550(1.12/0.38*)	AT4G22690(1.06/1.16*)
		AT3G47360(5.91/10.65*)	AT2G18450(0.44/0.43*)	AT5G63590(0.60/0.49*)	AT2G02580(1.56/2.01*)	AT5G24960(1.71/2.22*)	AT1G17745(0.74/0.54*)
		AT4G08770(1.68/0.39*)	AT4G33870(1.02/2.97*)	AT3G26320(0.78/0.49*)	AT2G30840(0.79/0.43*)	AT2G12190(8.79/9.34*)	AT1G31230(0.57/0.48*)
		AT3G46500(0.21/0.22*)	AT2G27010(0.37/0.21*)	AT3G20940(1.37/0.40*)	AT3G20950(0.63/0.24*)	AT2G29290(2.15/1.22*)	AT2G14100(63.98/5.82*)
		AT1G62610(1.19/1.37*)	AT4G19170(2.15/3.66*)	AT1G72610(0.38/0.25*)	AT2G24800(0.60/3.36*)	AT2G21910(1.31/2.74*)	AT4G08780(3.25/0.23*)
		AT1G14120(0.40/0.44*)	AT1G17890(1.45/1.41*)	AT1G18020(1.65/2.40*)	AT2G38080(1.41/2.19*)	AT2G27690(0.39/0.42*)	AT5G65165(2.13/0.34*)
		AT1G54870(6.54/0.07*)	AT5G58910(0.74/0.34*)	AT2G27000(0.92/0.50*)	AT4G12330(55.28/68.16*)	AT4G22880(1.00/0.23*)	AT5G59540(6.92/12.68*)
		AT5G19890(0.96/0.26*)	AT1G75450(1.18/2.90*)	AT1G16700(0.62/0.40*)	AT1G62540(0.25/0.04*)	AT5G05580(0.38/0.27*)	AT4G17260(1.55/2.08*)
		AT5G22500(4.94/3.47*)	AT4G37310(0.13/0.16*)	AT1G77120(1.20/3.06*)	AT1G09090(1.04/0.64*)	AT5G14130(0.91/0.37*)	AT5G38970(1.66/2.81*)
		AT5G50600(0.89/0.23*)	AT4G31870(0.59/0.33*)	AT5G19880(1.26/0.42*)	AT5G42250(1.36/1.92*)	AT3G13610(1.66/0.47*)	AT3G61040(1.00/0.16*)
		AT4G15300(6.06/3.74*)	AT3G30775(0.46/0.17*)	AT5G13430(0.63/0.50*)	AT4G37400(1.14/0.32*)	AT3G48270(0.92/0.27*)	AT5G39190(68.20/51.10*)
		AT1G01190(1.26/3.72*)	AT4G13770(0.05/0.06*)	AT3G44540(0.56/0.21*)	AT3G56700(1.67/2.34*)	AT4G32170(1.00/0.46*)	AT4G33010(0.59/0.43*)
		AT1G17990(1.65/2.62*)	AT3G26770(0.98/0.61*)	AT3G49110(0.60/2.51*)	AT1G79470(0.43/0.47*)	AT2G29350(1.00/0.09*)	AT1G20020(0.59/0.45*)
		AT4G15330(0.89/0.29*)	AT5G06720(1.83/0.13*)	AT3G01190(1.42/3.23*)	AT1G17420(0.94/1.15*)	AT1G01580(0.82/0.21*)	AT2G18980(1.38/2.54*)
		AT5G04330(1.92/2.24*)	AT5G67400(0.47/0.48*)	AT5G54080(4.55/2.45*)	AT3G28740(2.62/11.74*)	AT2G38390(0.56/0.19*)	AT3G56350(0.44/0.11*)
		AT1G64940(5.46/5.50*)	AT5G54190(1.82/2.60*)	AT2G21890(2.23/2.42*)	AT1G76680(1.92/3.18*)	AT3G46480(0.11/0.13*)	AT2G24190(0.72/0.74*)
		AT2G34770(2.47/1.86*)	AT3G26230(1.31/2.31*)	AT5G64100(2.27/5.21*)	AT3G44560(4.03/4.79*)	AT5G05340(0.20/0.06*)	AT5G05600(0.54/0.48*)
		AT5G01600(2.40/2.72*)	AT3G59890(1.88/1.78*)	AT3G12120(1.81/2.25*)	AT1G17010(0.77/0.34*)	AT3G05260(1.96/0.22*)	AT2G22330(0.42/0.34*)
		AT3G44550(0.36/0.30*)	AT4G32810(4.71/3.64*)	AT2G29330(0.67/0.48*)	AT5G43440(9.42/17.51*)	AT4G39950(0.80/0.34*)	AT2G24580(0.59/0.42*)
		AT5G38420(0.89/0.78*)	AT5G52320(0.56/0.28*)	AT4G04610(0.77/0.37*)	AT2G41480(1.02/0.12*)	AT4G37970(0.73/0.32*)	AT1G09420(0.32/0.26*)
		AT2G28860(2.99/0.59*)	AT5G07390(1.00/1.00*)	AT4G22110(1.11/0.43*)	AT2G44990(1.00/0.23*)	AT2G29300(0.09/14.48*)	AT3G19270(1.55/2.57*)
		AT5G21100(1.51/3.00*)	AT1G16410(0.52/0.25*)	AT4G37330(1.83/2.22*)	AT5G24140(0.66/0.40*)	AT3G26280(1.23/2.06*)	AT1G63940(0.69/0.61*)
		AT4G13310(1.42/0.33*)	AT2G19800(0.69/0.44*)	AT3G26125(1.86/3.75*)	AT3G51240(0.42/0.23*)	AT3G26220(2.15/2.57*)	AT2G19900(0.84/0.40*)
		AT4G12280(0.26/0.14*)	AT4G37320(1.34/2.20*)				
heme	87	AT3G26230(1.31/2.31*)	AT2G34770(2.47/1.86*)	AT5G19890(0.96/0.26*)	AT5G64100(2.27/5.21*)	AT5G05340(0.20/0.06*)	AT5G06730(0.57/0.34*)
		AT3G10520(0.35/0.42*)	AT1G64950(102.53/150.78*)	AT2G46650(0.26/0.21*)	AT5G05690(1.92/2.08*)	AT1G78490(1.20/0.29*)	AT4G37310(0.13/0.16*)

		AT4G20235(2.22/2.14*)	AT2G46950(0.34/0.26*)	AT5G14130(0.91/0.37*)	AT3G26300(50.20/12.69*)	AT1G49570(1.42/0.28*)	AT5G38970(1.66/2.81*)
		AT3G01900(0.58/0.24*)	AT2G22330(0.42/0.34*)	AT2G30750(2.04/2.02*)	AT5G42580(0.97/0.35*)	AT5G19880(1.26/0.42*)	AT2G25160(0.88/0.39*)
		AT4G39950(0.80/0.34*)	AT2G07727(0.60/0.40*)	AT3G48320(0.68/0.45*)	AT5G52320(0.56/0.28*)	AT4G15440(1.00/0.78*)	AT3G61040(1.00/0.16*)
		AT2G41480(1.02/0.12*)	AT4G15300(6.06/3.74*)	AT1G16400(0.67/0.25*)	AT1G19630(1.98/3.61*)	AT1G14550(1.12/0.38*)	AT3G26330(0.84/0.22*)
		AT4G22690(1.06/1.16*)	AT2G28860(2.99/0.59*)	AT4G37400(1.14/0.32*)	AT3G48270(0.92/0.27*)	AT5G25130(0.37/0.41*)	AT2G02580(1.56/2.01*)
		AT3G30180(3.42/2.29*)	AT5G24960(1.71/2.22*)	AT3G19270(1.55/2.57*)	AT1G01190(1.26/3.72*)	AT4G13770(0.05/0.06*)	AT4G08770(1.68/0.39*)
		AT1G16410(0.52/0.25*)	AT4G32170(1.00/0.46*)	AT1G34510(0.94/0.48*)	AT4G33870(1.02/2.97*)	AT3G26320(0.78/0.49*)	AT4G37330(1.83/2.22*)
		AT5G05260(1.03/0.44*)	AT3G49110(0.60/2.51*)	AT3G44970(1.42/2.67*)	AT5G24070(0.81/0.43*)	AT2G12190(8.79/9.34*)	AT1G14540(1.29/0.44*)
		AT2G27010(0.37/0.21*)	AT4G15330(0.89/0.29*)	AT3G26280(1.23/2.06*)	AT5G06720(1.83/0.13*)	AT3G20940(1.37/0.40*)	AT3G01190(1.42/3.23*)
		AT3G20950(0.63/0.24*)	AT4G13310(1.42/0.33*)	AT5G25120(0.50/0.43*)	AT2G14100(63.98/5.82*)	AT3G26125(1.86/3.75*)	AT3G26220(2.15/2.57*)
		AT4G36220(0.28/0.20*)	AT2G18980(1.38/2.54*)	AT2G24800(0.60/3.36*)	AT2G21910(1.31/2.74*)	AT5G04330(1.92/2.24*)	AT4G08780(3.25/0.23*)
		AT5G67400(0.47/0.48*)	AT2G38390(0.56/0.19*)	AT3G28740(2.62/11.74*)	AT4G37320(1.34/2.20*)	AT2G27690(0.39/0.42*)	AT1G64940(5.46/5.50*)
		AT5G45040(0.50/0.55*)	AT5G42650(0.50/0.33*)	AT2G27000(0.92/0.50*)			
iron	132	AT1G80830(2.15/2.43*)	AT5G19890(0.96/0.26*)	AT1G16700(0.62/0.40*)	AT4G37310(0.13/0.16*)	AT5G14130(0.91/0.37*)	AT1G49570(1.42/0.28*)
		AT5G38970(1.66/2.81*)	AT2G36490(1.58/1.73*)	AT5G42580(0.97/0.35*)	AT5G19880(1.26/0.42*)	AT2G07727(0.60/0.40*)	AT3G48320(0.68/0.45*)
		AT3G13610(1.66/0.47*)	AT3G61040(1.00/0.16*)	AT4G15440(1.00/0.78*)	AT1G16400(0.67/0.25*)	AT4G15300(6.06/3.74*)	AT1G19630(1.98/3.61*)
		AT5G13430(0.63/0.50*)	AT3G26330(0.84/0.22*)	AT5G08640(0.14/0.11*)	AT4G37400(1.14/0.32*)	AT3G48270(0.92/0.27*)	AT5G25130(0.37/0.41*)
		AT3G30180(3.42/2.29*)	AT1G01190(1.26/3.72*)	AT4G13770(0.05/0.06*)	AT3G61400(0.66/0.14*)	AT4G32170(1.00/0.46*)	AT1G34510(0.94/0.48*)
		AT3G45140(0.06/0.11*)	AT5G05260(1.03/0.44*)	AT3G49110(0.60/2.51*)	AT5G24070(0.81/0.43*)	AT1G14540(1.29/0.44*)	AT4G15330(0.89/0.29*)
		AT5G06720(1.83/0.13*)	AT5G63600(2.89/2.12*)	AT3G01190(1.42/3.23*)	AT1G17420(0.94/1.15*)	AT2G18130(0.81/0.48*)	AT5G25120(0.50/0.43*)
		AT4G36220(0.28/0.20*)	AT2G18980(1.38/2.54*)	AT2G38240(0.98/0.14*)	AT5G04330(1.92/2.24*)	AT5G67400(0.47/0.48*)	AT5G54080(4.55/2.45*)
		AT2G38390(0.56/0.19*)	AT3G28740(2.62/11.74*)	AT1G30040(0.87/0.94*)	AT1G64940(5.46/5.50*)	AT5G54000(3.17/2.09*)	AT5G42650(0.50/0.33*)
		AT2G36690(1.48/4.23*)	AT3G46480(0.11/0.13*)	AT1G12010(12.46/23.01*)	AT3G26230(1.31/2.31*)	AT2G34770(2.47/1.86*)	AT5G64100(2.27/5.21*)
		AT3G46490(0.96/0.23*)	AT5G06730(0.57/0.34*)	AT5G05340(0.20/0.06*)	AT3G10520(0.35/0.42*)	AT4G18350(0.58/0.47*)	AT1G64950(102.53/150.78*)
		AT2G46650(0.26/0.21*)	AT5G05690(1.92/2.08*)	AT1G78490(1.20/0.29*)	AT4G20235(2.22/2.14*)	AT5G05600(0.54/0.48*)	AT5G01600(2.40/2.72*)
		AT2G46950(0.34/0.26*)	AT5G59530(1.74/4.99*)	AT3G26300(50.20/12.69*)	AT1G17010(0.77/0.34*)	AT3G01900(0.58/0.24*)	AT2G30750(2.04/2.02*)
		AT2G22330(0.42/0.34*)	AT4G32810(4.71/3.64*)	AT5G43440(9.42/17.51*)	AT2G01880(0.89/0.11*)	AT2G25160(0.88/0.39*)	AT4G39950(0.80/0.34*)
		AT5G24380(0.58/0.47*)	AT5G52320(0.56/0.28*)	AT2G41480(1.02/0.12*)	AT1G14550(1.12/0.38*)	AT1G14700(0.61/0.50*)	AT4G22690(1.06/1.16*)
		AT2G28860(2.99/0.59*)	AT5G63590(0.60/0.49*)	AT2G02580(1.56/2.01*)	AT3G52780(4.27/2.64*)	AT2G44990(1.00/0.23*)	AT5G24960(1.71/2.22*)
		AT3G19270(1.55/2.57*)	AT4G08770(1.68/0.39*)	AT1G16410(0.52/0.25*)	AT4G33870(1.02/2.97*)	AT3G26320(0.78/0.49*)	AT2G30840(0.79/0.43*)
		AT4G37330(1.83/2.22*)	AT3G44970(1.42/2.67*)	AT2G12190(8.79/9.34*)	AT3G46500(0.21/0.22*)	AT2G27010(0.37/0.21*)	AT3G26280(1.23/2.06*)
		AT3G20940(1.37/0.40*)	AT3G20950(0.63/0.24*)	AT4G13310(1.42/0.33*)	AT2G14100(63.98/5.82*)	AT2G19800(0.69/0.44*)	AT4G19170(2.15/3.66*)
		AT3G26125(1.86/3.75*)	AT3G26220(2.15/2.57*)	AT3G51240(0.42/0.23*)	AT4G19690(0.74/0.06*)	AT2G24800(0.60/3.36*)	AT3G52820(0.63/0.45*)
		AT2G21910(1.31/2.74*)	AT4G24890(0.74/1.18*)	AT4G08780(3.25/0.23*)	AT1G14120(0.40/0.44*)	AT4G37320(1.34/2.20*)	AT2G27690(0.39/0.42*)
		AT5G65165(2.13/0.34*)	AT5G45040(0.50/0.55*)	AT4G19680(0.51/0.18*)	AT2G27000(0.92/0.50*)	AT4G12330(55.28/68.16*)	AT5G59540(6.92/12.68*)
glycoprotein	189	AT5G19240(0.78/0.79*)	AT4G18290(1.14/2.05*)	AT1G75450(1.18/2.90*)	AT5G19890(0.96/0.26*)	AT1G52400(0.10/0.10*)	AT1G66280(0.91/0.40*)
		AT2G22980(0.72/0.49*)	AT2G28990(1.31/2.54*)	AT4G11320(6.67/12.10*)	AT5G27100(1.07/0.96*)	AT1G60270(0.27/0.30*)	AT2G47550(0.67/0.31*)
		AT1G10550(1.01/2.41*)	AT5G57530(0.27/0.23*)	AT4G23160(1.00/1.00*)	AT3G62750(1.64/2.24*)	AT1G75910(0.53/0.48*)	AT4G04570(0.60/0.32*)

		AT4G23560(0.78/2.68*)	AT5G14130(0.91/0.37*)	AT1G49570(1.42/0.28*)	AT5G03810(0.29/0.14*)	AT5G39160(16.59/25.74*)	AT1G54030(0.72/0.43*)
		AT5G25090(0.75/0.45*)	AT2G32400(0.66/0.44*)	AT5G19880(1.26/0.42*)	AT4G27520(0.37/0.45*)	AT4G36880(0.45/3.97*)	AT4G28850(0.96/0.94*)
		AT2G35770(0.77/0.25*)	AT5G40730(1.89/2.73*)	AT1G51470(0.78/0.30*)	AT5G56540(1.95/2.19*)	AT3G14210(4.68/10.72*)	AT1G71990(0.50/0.48*)
		AT5G10180(1.13/0.44*)	AT3G30875(1.00/0.34*)	AT4G23220(6.85/5.09*)	AT1G11370(0.05/0.05*)	AT5G46150(2.59/3.18*)	AT4G02330(2.46/2.03*)
		AT2G26440(0.30/0.34*)	AT1G73280(0.35/0.31*)	AT1G53990(2.48/4.16*)	AT5G25980(13.03/33.60*)	AT1G35710(2.14/2.07*)	AT5G61350(0.26/0.43*)
		AT3G05950(2.20/0.01*)	AT5G39190(68.20/51.10*)	AT4G16230(1.77/3.07*)	AT3G45970(1.00/0.40*)	AT2G38180(1.51/2.16*)	AT1G34510(0.94/0.48*)
		AT4G10250(2.81/3.89*)	AT2G13790(27.39/16.44*)	AT3G49110(0.60/2.51*)	AT4G15100(0.86/0.15*)	AT5G24070(0.81/0.43*)	AT1G14540(1.29/0.44*)
		AT3G47295(0.61/0.63*)	AT4G02320(0.64/0.44*)	AT4G23210(0.35/0.22*)	AT5G06720(1.83/0.13*)	AT2G26450(0.36/0.24*)	AT3G01190(1.42/3.23*)
		AT4G23290(0.12/0.27*)	AT2G18130(0.81/0.48*)	AT5G55630(1.03/1.24*)	AT2G13800(0.88/0.80*)	AT4G11310(6.44/5.46*)	AT2G24560(0.94/0.36*)
		AT1G18980(0.51/0.49*)	AT3G45960(1.01/0.68*)	AT5G46330(0.84/2.08*)	AT5G67400(0.47/0.48*)	AT4G40090(0.75/0.47*)	AT2G38390(0.56/0.19*)
		AT1G77110(0.68/3.94*)	AT5G20710(39.08/10.61*)	AT3G11210(48.15/29.70*)	AT1G16260(7.77/13.20*)	AT2G47930(1.32/2.12*)	AT2G46330(1.92/2.06*)
		AT3G62280(3.88/2.74*)	AT4G11460(1.00/11.11*)	AT2G12480(0.12/0.19*)	AT5G64100(2.27/5.21*)	AT3G20270(0.61/0.46*)	AT5G38930(1.09/0.42*)
		AT5G06870(0.54/0.29*)	AT5G06730(0.57/0.34*)	AT5G05340(0.20/0.06*)	AT1G54000(1.16/0.48*)	AT1G53830(0.99/0.48*)	AT4G13420(0.20/0.49*)
		AT4G27830(1.33/2.60*)	AT1G02310(0.94/0.49*)	AT4G23260(0.55/0.41*)	AT1G76930(2.06/3.56*)	AT4G12420(2.97/2.94*)	AT5G24780(1.03/0.15*)
		AT3G06830(1.66/0.50*)	AT2G19150(2.65/2.30*)	AT1G27190(1.80/2.15*)	AT5G45960(0.43/0.45*)	AT1G61810(0.54/0.28*)	AT1G02850(1.79/2.35*)
		AT3G12220(2.06/2.25*)	AT1G05200(2.35/2.96*)	AT5G20630(1.47/4.16*)	AT5G38940(0.94/0.44*)	AT2G29130(2.65/2.01*)	AT4G17030(1.00/0.42*)
		AT1G18140(0.61/0.14*)	AT5G65390(1.49/2.08*)	AT5G57560(1.02/0.35*)	AT2G01880(0.89/0.11*)	AT1G31550(0.34/0.39*)	AT2G22470(2.46/2.02*)
		AT1G51820(0.38/0.29*)	AT1G47600(0.80/0.37*)	AT3G04290(0.42/0.12*)	AT1G18690(1.46/1.30*)	AT5G28510(1.26/6.24*)	AT2G41480(1.02/0.12*)
		AT5G24770(1.44/0.89*)	AT5G63180(1.20/2.66*)	AT2G44480(1.36/2.39*)	AT3G60140(4.30/3.53*)	AT1G19670(0.31/0.21*)	AT1G14550(1.12/0.38*)
		AT5G39130(9.24/11.30*)	AT1G14700(0.61/0.50*)	AT3G10450(2.21/2.00*)	AT5G55050(1.02/2.55*)	AT5G41300(8.09/16.73*)	AT1G28580(1.92/2.28*)
		AT5G58150(0.53/0.41*)	AT1G51890(1.22/3.57*)	AT3G52780(4.27/2.64*)	AT3G16370(3.80/2.81*)	AT5G44130(0.39/0.27*)	AT1G65240(0.17/0.17*)
		AT4G08770(1.68/0.39*)	AT1G54020(0.72/0.07*)	AT1G07550(3.13/4.36*)	AT2G22920(1.01/0.76*)	AT4G33870(1.02/2.97*)	AT3G21370(0.44/0.10*)
		AT2G44450(0.64/0.35*)	AT1G07560(2.67/2.86*)	AT4G18990(1.22/0.19*)	AT5G06390(0.07/0.05*)	AT3G52370(1.04/0.59*)	AT1G64390(1.47/1.15*)
		AT4G30610(0.48/0.36*)	AT5G46240(2.17/3.43*)	AT4G30810(0.79/0.39*)	AT5G24550(1.00/0.41*)	AT4G29180(0.52/0.48*)	AT2G15370(0.85/0.47*)
		AT2G18800(0.44/0.29*)	AT3G51740(0.73/0.36*)	AT1G18120(1.00/0.15*)	AT4G30280(2.35/2.12*)	AT3G13065(0.48/0.40*)	AT5G59680(2.24/3.61*)
		AT1G32860(0.26/0.53*)	AT2G42990(4.59/4.60*)	AT2G24800(0.60/3.36*)	AT2G23130(0.40/0.25*)	AT1G72610(0.38/0.25*)	AT3G52820(0.63/0.45*)
		AT4G24890(0.74/1.18*)	AT4G08780(3.25/0.23*)	AT1G17890(1.45/1.41*)	AT4G26466(1.13/0.23*)	AT1G27940(0.05/0.07*)	AT2G44460(1.19/2.53*)
		AT4G25810(1.15/0.43*)	AT5G01240(1.41/1.84*)	AT2G38080(1.41/2.19*)	AT1G28660(1.62/3.20*)	AT5G03820(0.75/0.32*)	AT1G43780(0.24/0.15*)
		AT5G58910(0.74/0.34*)	AT1G05700(1.34/2.39*)	AT1G65310(2.77/2.75*)			
Monooxygenase	66	AT2G34770(2.47/1.86*)	AT2G35660(0.35/0.33*)	AT3G26230(1.31/2.31*)	AT4G38540(1.34/1.72*)	AT1G62540(0.25/0.04*)	AT1G64950(102.53/150.78*)
		AT1G19250(8.13/2.01*)	AT5G05690(1.92/2.08*)	AT1G78490(1.20/0.29*)	AT4G20235(2.22/2.14*)	AT4G37310(0.13/0.16*)	AT2G46950(0.34/0.26*)
		AT3G26300(50.20/12.69*)	AT5G38970(1.66/2.81*)	AT1G65860(0.48/0.17*)	AT3G01900(0.58/0.24*)	AT2G22330(0.42/0.34*)	AT2G30750(2.04/2.02*)
		AT5G42580(0.97/0.35*)	AT2G25160(0.88/0.39*)	AT4G39950(0.80/0.34*)	AT3G48320(0.68/0.45*)	AT5G38420(0.89/0.78*)	AT5G52320(0.56/0.28*)
		AT3G61040(1.00/0.16*)	AT4G15300(6.06/3.74*)	AT1G16400(0.67/0.25*)	AT1G12130(2.00/0.49*)	AT3G26330(0.84/0.22*)	AT4G22690(1.06/1.16*)
		AT4G37400(1.14/0.32*)	AT2G28860(2.99/0.59*)	AT3G48270(0.92/0.27*)	AT5G25130(0.37/0.41*)	AT5G05320(2.87/3.02*)	AT2G02580(1.56/2.01*)
		AT3G30180(3.42/2.29*)	AT5G24960(1.71/2.22*)	AT1G01190(1.26/3.72*)	AT3G19270(1.55/2.57*)	AT4G13770(0.05/0.06*)	AT1G16410(0.52/0.25*)
		AT4G32170(1.00/0.46*)	AT3G26320(0.78/0.49*)	AT4G37330(1.83/2.22*)	AT5G05260(1.03/0.44*)	AT2G12190(8.79/9.34*)	AT2G27010(0.37/0.21*)
		AT4G15330(0.89/0.29*)	AT3G26280(1.23/2.06*)	AT3G20940(1.37/0.40*)	AT3G20950(0.63/0.24*)	AT4G13310(1.42/0.33*)	AT5G25120(0.50/0.43*)

		AT2G14100(63.98/5.82*)	AT3G26125(1.86/3.75*)	AT3G26220(2.15/2.57*)	AT4G36220(0.28/0.20*)	AT2G21910(1.31/2.74*)	AT5G04330(1.92/2.24*)
		AT3G28740(2.62/11.74*)	AT2G27690(0.39/0.42*)	AT4G37320(1.34/2.20*)	AT1G64940(5.46/5.50*)	AT5G38430(0.82/0.69*)	AT2G27000(0.92/0.50*)
signal	244	AT1G52400(0.10/0.10*)	AT2G15010(1.40/0.07*)	AT4G11320(6.67/12.10*)	AT3G44990(0.98/5.25*)	AT3G14220(4.49/7.00*)	AT1G10550(1.01/2.41*)
		AT5G57530(0.27/0.23*)	AT4G23160(1.00/1.00*)	AT1G75910(0.53/0.48*)	AT4G23560(0.78/2.68*)	AT1G49570(1.42/0.28*)	AT1G18250(0.60/0.34*)
		AT5G03810(0.29/0.14*)	AT4G36880(0.45/3.97*)	AT4G28850(0.96/0.94*)	AT5G40730(1.89/2.73*)	AT1G72260(0.99/0.55*)	AT1G51470(0.78/0.30*)
		AT5G56540(1.95/2.19*)	AT3G14210(4.68/10.72*)	AT1G16400(0.67/0.25*)	AT1G73280(0.35/0.31*)	AT3G22060(0.22/0.23*)	AT5G25980(13.03/33.60*)
		AT4G29305(1.00/0.21*)	AT5G61350(0.26/0.43*)	AT4G16230(1.77/3.07*)	AT3G45970(1.00/0.40*)	AT2G37640(1.11/2.12*)	AT5G43980(1.03/2.50*)
		AT1G34510(0.94/0.48*)	AT5G24070(0.81/0.43*)	AT1G14540(1.29/0.44*)	AT3G47295(0.61/0.63*)	AT2G05117(0.04/0.06*)	AT2G18130(0.81/0.48*)
		AT4G23290(0.12/0.27*)	AT2G13800(0.88/0.80*)	AT4G11310(6.44/5.46*)	AT3G45960(1.01/0.68*)	AT5G46330(0.84/2.08*)	AT4G02290(1.20/1.39*)
		AT5G20710(39.08/10.61*)	AT1G16260(7.77/13.20*)	AT1G19610(0.36/0.35*)	AT3G55500(1.00/0.92*)	AT5G01540(0.29/0.41*)	AT2G46330(1.92/2.06*)
		AT4G11460(1.00/11.11*)	AT2G12480(0.12/0.19*)	AT3G20270(0.61/0.46*)	AT4G13235(0.98/0.36*)	AT5G05290(0.40/2.19*)	AT5G38930(1.09/0.42*)
		AT5G06870(0.54/0.29*)	AT5G06730(0.57/0.34*)	AT4G13230(0.55/0.17*)	AT1G02310(0.94/0.49*)	AT4G23260(0.55/0.41*)	AT3G29030(1.36/1.16*)
		AT4G12420(2.97/2.94*)	AT5G24780(1.03/0.15*)	AT3G06830(1.66/0.50*)	AT4G18970(0.92/0.98*)	AT1G27190(1.80/2.15*)	AT1G02850(1.79/2.35*)
		AT1G05200(2.35/2.96*)	AT1G20190(1.44/2.07*)	AT3G21920(0.34/0.46*)	AT2G29130(2.65/2.01*)	AT5G38940(0.94/0.44*)	AT3G06985(0.64/0.49*)
		AT5G44120(1.51/0.08*)	AT2G43550(0.19/0.20*)	AT1G18140(0.61/0.14*)	AT5G57560(1.02/0.35*)	AT2G22470(2.46/2.02*)	AT1G51820(0.38/0.29*)
		AT1G11580(0.71/0.33*)	AT4G27160(0.35/0.14*)	AT5G24770(1.44/0.89*)	AT4G27170(0.35/0.14*)	AT5G63180(1.20/2.66*)	AT1G14550(1.12/0.38*)
		AT3G10450(2.21/2.00*)	AT3G21930(0.39/0.47*)	AT5G55050(1.02/2.55*)	AT1G28580(1.92/2.28*)	AT2G02990(1.24/4.93*)	AT5G58150(0.53/0.41*)
		AT3G52780(4.27/2.64*)	AT3G16370(3.80/2.81*)	AT5G44130(0.39/0.27*)	AT4G08770(1.68/0.39*)	AT1G07550(3.13/4.36*)	AT1G25054(6.37/6.65*)
		AT4G33870(1.02/2.97*)	AT1G24880(6.03/7.30*)	AT2G32270(1.32/2.48*)	AT1G07560(2.67/2.86*)	AT4G18990(1.22/0.19*)	AT2G43860(0.29/0.33*)
		AT3G52370(1.04/0.59*)	AT4G30610(0.48/0.36*)	AT5G24550(1.00/0.41*)	AT1G13590(1.30/2.59*)	AT1G18120(1.00/0.15*)	AT4G30280(2.35/2.12*)
		AT1G62080(0.61/0.27*)	AT5G59680(2.24/3.61*)	AT3G59930(11.12/97.99*)	AT1G32860(0.26/0.53*)	AT1G64195(1.36/2.40*)	AT2G42990(4.59/4.60*)
		AT2G24800(0.60/3.36*)	AT1G72610(0.38/0.25*)	AT2G23130(0.40/0.25*)	AT4G08780(3.25/0.23*)	AT1G17890(1.45/1.41*)	AT2G38080(1.41/2.19*)
		AT4G25810(1.15/0.43*)	AT1G34047(1.06/2.05*)	AT1G03880(0.34/0.07*)	AT5G03820(0.75/0.32*)	AT1G43780(0.24/0.15*)	AT5G58910(0.74/0.34*)
		AT1G05700(1.34/2.39*)	AT2G43535(4.42/6.34*)	AT1G65310(2.77/2.75*)	AT5G56320(1.08/0.37*)	AT5G19240(0.78/0.79*)	AT5G19890(0.96/0.26*)
		AT1G75450(1.18/2.90*)	AT1G66280(0.91/0.40*)	AT2G22980(0.72/0.49*)	AT2G28990(1.31/2.54*)	AT5G27100(1.07/0.96*)	AT1G60270(0.27/0.30*)
		AT2G47550(0.67/0.31*)	AT3G62750(1.64/2.24*)	AT4G04570(0.60/0.32*)	AT1G44350(0.35/0.48*)	AT5G14130(0.91/0.37*)	AT4G27140(0.67/0.04*)
		AT5G39160(16.59/25.74*)	AT1G54030(0.72/0.43*)	AT5G25090(0.75/0.45*)	AT2G32400(0.66/0.44*)	AT5G19880(1.26/0.42*)	AT4G27520(0.37/0.45*)
		AT2G35770(0.77/0.25*)	AT4G29285(1.00/0.04*)	AT4G23220(6.85/5.09*)	AT4G29283(0.78/0.10*)	AT1G11370(0.05/0.05*)	AT4G02330(2.46/2.03*)
		AT2G26440(0.30/0.34*)	AT4G27150(0.83/0.13*)	AT4G29280(0.70/0.03*)	AT1G53990(2.48/4.16*)	AT1G35710(2.14/2.07*)	AT3G05950(2.20/0.01*)
		AT5G39190(68.20/51.10*)	AT2G38180(1.51/2.16*)	AT3G05727(0.74/2.18*)	AT4G10250(2.81/3.89*)	AT2G13790(27.39/16.44*)	AT3G49110(0.60/2.51*)
		AT4G15100(0.86/0.15*)	AT4G02320(0.64/0.44*)	AT5G48540(0.56/0.44*)	AT4G23210(0.35/0.22*)	AT5G06720(1.83/0.13*)	AT3G01190(1.42/3.23*)
		AT1G18980(0.51/0.49*)	AT2G24560(0.94/0.36*)	AT4G11650(1.76/4.04*)	AT2G18980(1.38/2.54*)	AT3G05730(3.07/2.70*)	AT3G47300(0.99/0.68*)
		AT5G67400(0.47/0.48*)	AT4G40090(0.75/0.47*)	AT2G38390(0.56/0.19*)	AT3G11210(48.15/29.70*)	AT2G47930(1.32/2.12*)	AT3G62280(3.88/2.74*)
		AT5G64100(2.27/5.21*)	AT5G59320(0.98/0.32*)	AT5G05340(0.20/0.06*)	AT1G54000(1.16/0.48*)	AT2G02130(0.38/0.39*)	AT1G53830(0.99/0.48*)
		AT4G27830(1.33/2.60*)	AT1G76930(2.06/3.56*)	AT2G19150(2.65/2.30*)	AT5G45960(0.43/0.45*)	AT3G21250(1.85/2.25*)	AT1G61810(0.54/0.28*)
		AT3G12220(2.06/2.25*)	AT5G20630(1.47/4.16*)	AT2G15050(12.90/17.49*)	AT4G17030(1.00/0.42*)	AT2G01880(0.89/0.11*)	AT5G65390(1.49/2.08*)
		AT1G31550(0.34/0.39*)	AT2G22121(0.42/0.37*)	AT1G47600(0.80/0.37*)	AT3G04290(0.42/0.12*)	AT5G28510(1.26/6.24*)	AT2G41480(1.02/0.12*)
		AT2G44480(1.36/2.39*)	AT3G60140(4.30/3.53*)	AT5G33355(10.61/47.95*)	AT5G39130(9.24/11.30*)	AT1G14700(0.61/0.50*)	AT5G41300(8.09/16.73*)

		AT1G51890(1.22/3.57*)	AT1G65240(0.17/0.17*)	AT2G01660(1.54/2.06*)	AT4G22217(1.59/3.07*)	AT1G54020(0.72/0.07*)	AT1G16410(0.52/0.25*)
		AT2G22920(1.01/0.76*)	AT3G21370(0.44/0.10*)	AT4G09610(0.66/0.29*)	AT2G44450(0.64/0.35*)	AT5G06390(0.07/0.05*)	AT1G64390(1.47/1.15*)
		AT4G22214(3.08/0.16*)	AT4G30810(0.79/0.39*)	AT4G29180(0.52/0.48*)	AT1G62000(0.54/0.38*)	AT2G18800(0.44/0.29*)	AT2G02120(0.86/0.43*)
		AT3G51740(0.73/0.36*)	AT3G13065(0.48/0.40*)	AT4G19690(0.74/0.06*)	AT3G52820(0.63/0.45*)	AT4G24890(0.74/1.18*)	AT4G26466(1.13/0.23*)
		AT4G09600(0.97/0.04*)	AT2G44460(1.19/2.53*)	AT1G28660(1.62/3.20*)	AT4G19680(0.51/0.18*)		
Plant_defense	112	AT1G72870(1.72/4.27*)	AT5G44210(1.35/2.58*)	AT2G15010(1.40/0.07*)	AT3G44400(6.94/10.91*)	AT1G19250(8.13/2.01*)	AT3G61220(0.41/0.39*)
		AT5G13220(0.86/0.35*)	AT1G56520(0.26/0.31*)	AT5G46260(9.81/16.45*)	AT2G44840(0.65/0.34*)	AT5G09980(0.85/0.31*)	AT1G72260(0.99/0.55*)
		AT5G64890(3.94/0.29*)	AT3G44480(0.34/0.34*)	AT4G29285(1.00/0.04*)	AT4G29283(0.78/0.10*)	AT4G16900(1.04/0.85*)	AT5G45490(40.88/33.10*)
		AT3G07040(0.50/0.48*)	AT3G46530(30.77/53.76*)	AT4G29280(0.70/0.03*)	AT1G69550(1.99/3.47*)	AT5G25980(13.03/33.60*)	AT4G29305(1.00/0.21*)
		AT1G12210(1.25/7.25*)	AT4G16960(71.96/6.89*)	AT4G16860(1.57/1.40*)	AT3G46730(1.11/2.17*)	AT5G40910(5.49/4.20*)	AT3G05727(0.74/2.18*)
		AT5G41740(1.71/2.77*)	AT5G40090(2.62/2.89*)	AT1G63360(2.36/2.77*)	AT1G74710(0.55/0.34*)	AT5G58120(0.53/0.22*)	AT3G50950(0.55/0.41*)
		AT2G05117(0.04/0.06*)	AT4G19510(1.08/1.07*)	AT3G44630(19.64/13.71*)	AT1G12220(2.01/3.53*)	AT5G46470(2.45/2.31*)	AT4G16950(1.15/1.32*)
		AT3G05730(3.07/2.70*)	AT5G46330(0.84/2.08*)	AT1G59124(7.61/11.45*)	AT1G69545(0.47/0.26*)	AT1G63880(23.34/47.41*)	AT3G45290(1.26/2.28*)
		AT1G72450(0.14/0.36*)	AT1G19610(0.36/0.35*)	AT5G35450(2.24/2.37*)	AT5G41750(0.22/0.19*)	AT1G26700(1.30/2.04*)	AT1G30135(1.00/0.14*)
		AT1G58848(6.64/11.54*)	AT5G43470(49.01/45.79*)	AT1G61190(0.43/0.47*)	AT4G13235(0.98/0.36*)	AT2G33340(2.23/2.83*)	AT2G02130(0.38/0.39*)
		AT1G09770(1.92/2.02*)	AT5G45440(0.79/0.38*)	AT5G48620(15.99/14.59*)	AT2G34600(0.68/0.37*)	AT1G61310(6.16/49.29*)	AT1G59218(61.60/117.99*)
		AT1G15890(2.21/2.12*)	AT5G47250(0.27/0.22*)	AT1G61180(0.74/0.92*)	AT3G06985(0.64/0.49*)	AT1G50180(0.24/0.35*)	AT2G43550(0.19/0.20*)
		AT5G46490(13.93/7.46*)	AT1G11580(0.71/0.33*)	AT2G22121(0.42/0.37*)	AT4G19050(0.49/0.43*)	AT1G61300(0.33/0.36*)	AT5G33355(10.61/47.95*)
		AT5G38350(2.46/3.93*)	AT1G62630(1.00/13.38*)	AT4G39030(2.16/0.61*)	AT5G36930(3.05/2.28*)	AT1G72840(19.87/16.64*)	AT4G22217(1.59/3.07*)
		AT5G47220(0.15/0.23*)	AT4G09420(0.99/0.49*)	AT1G58400(5.00/3.77*)	AT5G18360(6.12/3.71*)	AT5G17890(28.19/28.15*)	AT4G22214(3.08/0.16*)
		AT5G45510(1.20/1.28*)	AT1G56540(1.35/2.14*)	AT2G02120(0.86/0.43*)	AT5G48780(2.72/2.51*)	AT3G59930(11.12/97.99*)	AT1G58807(92.90/68.68*)
		AT1G32860(0.26/0.53*)	AT3G44670(0.84/0.77*)	AT1G63750(1.27/1.51*)	AT1G66090(1.54/3.29*)	AT1G64195(1.36/2.40*)	AT1G59780(3.13/3.29*)
		AT4G16990(4.06/5.22*)	AT1G34047(1.06/2.05*)	AT5G17880(6.71/10.80*)	AT1G58602(4.40/11.24*)	AT5G63020(9.47/8.43*)	AT2G33670(1.17/2.03*)
		AT2G43535(4.42/6.34*)	AT1G51480(0.04/0.06*)	AT5G43740(8.34/7.08*)	AT5G64900(0.83/0.12*)		
disulfide_bond	133	AT5G19890(0.96/0.26*)	AT1G52400(0.10/0.10*)	AT1G66280(0.91/0.40*)	AT2G22980(0.72/0.49*)	AT2G15010(1.40/0.07*)	AT1G45145(0.26/0.32*)
		AT4G11320(6.67/12.10*)	AT3G44990(0.98/5.25*)	AT1G60270(0.27/0.30*)	AT2G47550(0.67/0.31*)	AT1G10550(1.01/2.41*)	AT5G57530(0.27/0.23*)
		AT1G66340(1.43/2.07*)	AT5G14130(0.91/0.37*)	AT1G18250(0.60/0.34*)	AT1G49570(1.42/0.28*)	AT4G27140(0.67/0.04*)	AT5G39160(16.59/25.74*)
		AT5G19880(1.26/0.42*)	AT4G36880(0.45/3.97*)	AT4G28850(0.96/0.94*)	AT2G35770(0.77/0.25*)	AT1G72260(0.99/0.55*)	AT1G51470(0.78/0.30*)
		AT1G60740(3.01/5.18*)	AT3G51030(0.50/0.36*)	AT4G29285(1.00/0.04*)	AT4G29283(0.78/0.10*)	AT4G02330(2.46/2.03*)	AT2G26440(0.30/0.34*)
		AT1G73280(0.35/0.31*)	AT4G27150(0.83/0.13*)	AT4G29280(0.70/0.03*)	AT5G25980(13.03/33.60*)	AT4G29305(1.00/0.21*)	AT3G05950(2.20/0.01*)
		AT5G39190(68.20/51.10*)	AT2G37640(1.11/2.12*)	AT1G34510(0.94/0.48*)	AT3G05727(0.74/2.18*)	AT3G49110(0.60/2.51*)	AT4G15100(0.86/0.15*)
		AT5G24070(0.81/0.43*)	AT1G14540(1.29/0.44*)	AT1G20020(0.59/0.45*)	AT4G02320(0.64/0.44*)	AT5G06720(1.83/0.13*)	AT2G26450(0.36/0.24*)
		AT2G05117(0.04/0.06*)	AT3G01190(1.42/3.23*)	AT4G11650(1.76/4.04*)	AT1G18980(0.51/0.49*)	AT4G11310(6.44/5.46*)	AT2G18980(1.38/2.54*)
		AT3G05730(3.07/2.70*)	AT3G47300(0.99/0.68*)	AT5G67400(0.47/0.48*)	AT2G38390(0.56/0.19*)	AT1G16260(7.77/13.20*)	AT1G19610(0.36/0.35*)
		AT1G04310(2.81/3.80*)	AT5G64100(2.27/5.21*)	AT2G12480(0.12/0.19*)	AT5G59320(0.98/0.32*)	AT4G13235(0.98/0.36*)	AT5G05290(0.40/2.19*)
		AT5G38930(1.09/0.42*)	AT5G06870(0.54/0.29*)	AT5G06730(0.57/0.34*)	AT2G02130(0.38/0.39*)	AT5G05340(0.20/0.06*)	AT1G53830(0.99/0.48*)
		AT4G27830(1.33/2.60*)	AT3G29030(1.36/1.16*)	AT3G06830(1.66/0.50*)	AT1G61810(0.54/0.28*)	AT1G02850(1.79/2.35*)	AT3G12220(2.06/2.25*)
		AT5G20630(1.47/4.16*)	AT3G26060(0.72/0.65*)	AT5G38940(0.94/0.44*)	AT2G15050(12.90/17.49*)	AT5G44120(1.51/0.08*)	AT3G06985(0.64/0.49*)

		AT2G43550(0.19/0.20*)	AT5G57560(1.02/0.35*)	AT4G27160(0.35/0.14*)	AT2G22121(0.42/0.37*)	AT1G47600(0.80/0.37*)	AT4G04610(0.77/0.37*)
		AT2G41480(1.02/0.12*)	AT5G28510(1.26/6.24*)	AT4G27170(0.35/0.14*)	AT1G09420(0.32/0.26*)	AT2G44480(1.36/2.39*)	AT3G60140(4.30/3.53*)
		AT1G14550(1.12/0.38*)	AT5G33355(10.61/47.95*)	AT5G39130(9.24/11.30*)	AT3G10450(2.21/2.00*)	AT2G02990(1.24/4.93*)	AT3G62030(0.49/0.49*)
		AT4G22217(1.59/3.07*)	AT4G08770(1.68/0.39*)	AT2G22920(1.01/0.76*)	AT4G33870(1.02/2.97*)	AT3G21370(0.44/0.10*)	AT5G63030(1.45/1.22*)
		AT2G44450(0.64/0.35*)	AT4G09610(0.66/0.29*)	AT4G18990(1.22/0.19*)	AT4G22214(3.08/0.16*)	AT4G30610(0.48/0.36*)	AT4G30810(0.79/0.39*)
		AT5G24550(1.00/0.41*)	AT2G18800(0.44/0.29*)	AT2G02120(0.86/0.43*)	AT4G30280(2.35/2.12*)	AT3G59930(11.12/97.99*)	AT1G64195(1.36/2.40*)
		AT2G24800(0.60/3.36*)	AT1G72610(0.38/0.25*)	AT4G08780(3.25/0.23*)	AT1G17890(1.45/1.41*)	AT4G09600(0.97/0.04*)	AT4G25810(1.15/0.43*)
		AT2G44460(1.19/2.53*)	AT1G34047(1.06/2.05*)	AT5G14070(1.03/2.30*)	AT1G03880(0.34/0.07*)	AT1G43780(0.24/0.15*)	AT2G43535(4.42/6.34*)
		AT1G65310(2.77/2.75*)					
storage_protein	9	AT5G24780(1.03/0.15*)	AT5G44120(1.51/0.08*)	AT5G24770(1.44/0.89*)	AT4G27170(0.35/0.14*)	AT1G03880(0.34/0.07*)	AT4G28520(0.97/0.31*)
		AT4G27140(0.67/0.04*)	AT4G27150(0.83/0.13*)	AT4G27160(0.35/0.14*)			
Secreted	149	AT1G75450(1.18/2.90*)	AT5G19890(0.96/0.26*)	AT2G22980(0.72/0.49*)	AT2G15010(1.40/0.07*)	AT3G44990(0.98/5.25*)	AT2G47550(0.67/0.31*)
		AT3G14220(4.49/7.00*)	AT1G10550(1.01/2.41*)	AT5G57530(0.27/0.23*)	AT1G75910(0.53/0.48*)	AT4G23560(0.78/2.68*)	AT5G14130(0.91/0.37*)
		AT1G49570(1.42/0.28*)	AT5G03810(0.29/0.14*)	AT5G39160(16.59/25.74*)	AT1G54030(0.72/0.43*)	AT5G19880(1.26/0.42*)	AT4G28850(0.96/0.94*)
		AT2G35770(0.77/0.25*)	AT1G72260(0.99/0.55*)	AT3G14210(4.68/10.72*)	AT4G29285(1.00/0.04*)	AT4G29283(0.78/0.10*)	AT1G11370(0.05/0.05*)
		AT4G02330(2.46/2.03*)	AT2G26440(0.30/0.34*)	AT1G73280(0.35/0.31*)	AT3G22060(0.22/0.23*)	AT4G29280(0.70/0.03*)	AT1G53990(2.48/4.16*)
		AT4G29305(1.00/0.21*)	AT3G05950(2.20/0.01*)	AT4G16230(1.77/3.07*)	AT5G39190(68.20/51.10*)	AT2G37640(1.11/2.12*)	AT2G38180(1.51/2.16*)
		AT5G43980(1.03/2.50*)	AT1G34510(0.94/0.48*)	AT3G05727(0.74/2.18*)	AT3G49110(0.60/2.51*)	AT4G15100(0.86/0.15*)	AT5G24070(0.81/0.43*)
		AT1G14540(1.29/0.44*)	AT3G47295(0.61/0.63*)	AT4G02320(0.64/0.44*)	AT5G48540(0.56/0.44*)	AT5G06720(1.83/0.13*)	AT2G05117(0.04/0.06*)
		AT3G01190(1.42/3.23*)	AT2G18130(0.81/0.48*)	AT2G24560(0.94/0.36*)	AT1G18980(0.51/0.49*)	AT2G18980(1.38/2.54*)	AT3G05730(3.07/2.70*)
		AT3G45960(1.01/0.68*)	AT5G67400(0.47/0.48*)	AT4G02290(1.20/1.39*)	AT2G38390(0.56/0.19*)	AT5G20710(39.08/10.61*)	AT3G11210(48.15/29.70*)
		AT1G19610(0.36/0.35*)	AT3G55500(1.00/0.92*)	AT3G62280(3.88/2.74*)	AT5G64100(2.27/5.21*)	AT2G12480(0.12/0.19*)	AT4G13235(0.98/0.36*)
		AT5G05290(0.40/2.19*)	AT5G38930(1.09/0.42*)	AT5G06870(0.54/0.29*)	AT5G06730(0.57/0.34*)	AT5G05340(0.20/0.06*)	AT1G54000(1.16/0.48*)
		AT2G02130(0.38/0.39*)	AT1G53830(0.99/0.48*)	AT1G02310(0.94/0.49*)	AT3G29030(1.36/1.16*)	AT1G76930(2.06/3.56*)	AT4G12420(2.97/2.94*)
		AT3G06830(1.66/0.50*)	AT2G19150(2.65/2.30*)	AT4G18970(0.92/0.98*)	AT5G45960(0.43/0.45*)	AT3G12220(2.06/2.25*)	AT1G20190(1.44/2.07*)
		AT3G21920(0.34/0.46*)	AT5G20630(1.47/4.16*)	AT5G38940(0.94/0.44*)	AT2G29130(2.65/2.01*)	AT3G06985(0.64/0.49*)	AT4G17030(1.00/0.42*)
		AT2G43550(0.19/0.20*)	AT1G18140(0.61/0.14*)	AT5G57560(1.02/0.35*)	AT2G01880(0.89/0.11*)	AT1G31550(0.34/0.39*)	AT1G11580(0.71/0.33*)
		AT2G22121(0.42/0.37*)	AT3G04290(0.42/0.12*)	AT2G41480(1.02/0.12*)	AT1G14550(1.12/0.38*)	AT5G33355(10.61/47.95*)	AT5G39130(9.24/11.30*)
		AT1G14700(0.61/0.50*)	AT3G10450(2.21/2.00*)	AT3G21930(0.39/0.47*)	AT5G55050(1.02/2.55*)	AT1G28580(1.92/2.28*)	AT3G52780(4.27/2.64*)
		AT3G16370(3.80/2.81*)	AT2G01660(1.54/2.06*)	AT4G22217(1.59/3.07*)	AT4G08770(1.68/0.39*)	AT1G54020(0.72/0.07*)	AT2G22920(1.01/0.76*)
		AT4G33870(1.02/2.97*)	AT4G09610(0.66/0.29*)	AT4G18990(1.22/0.19*)	AT2G43860(0.29/0.33*)	AT5G06390(0.07/0.05*)	AT3G52370(1.04/0.59*)
		AT4G22214(3.08/0.16*)	AT4G30610(0.48/0.36*)	AT1G64390(1.47/1.15*)	AT4G30810(0.79/0.39*)	AT1G13590(1.30/2.59*)	AT2G18800(0.44/0.29*)
		AT2G02120(0.86/0.43*)	AT1G18120(1.00/0.15*)	AT4G30280(2.35/2.12*)	AT3G59930(11.12/97.99*)	AT1G32860(0.26/0.53*)	AT2G42990(4.59/4.60*)
		AT1G64195(1.36/2.40*)	AT2G24800(0.60/3.36*)	AT1G72610(0.38/0.25*)	AT3G52820(0.63/0.45*)	AT4G24890(0.74/1.18*)	AT4G08780(3.25/0.23*)
		AT1G17890(1.45/1.41*)	AT2G38080(1.41/2.19*)	AT4G09600(0.97/0.04*)	AT4G25810(1.15/0.43*)	AT1G34047(1.06/2.05*)	AT1G28660(1.62/3.20*)
		AT5G03820(0.75/0.32*)	AT1G43780(0.24/0.15*)	AT2G43535(4.42/6.34*)	AT1G65310(2.77/2.75*)	AT5G33370(0.83/0.81*)	
peroxidase	31	AT5G64100(2.27/5.21*)	AT5G19890(0.96/0.26*)	AT5G07390(1.00/1.00*)	AT5G05340(0.20/0.06*)	AT5G06730(0.57/0.34*)	AT4G08770(1.68/0.39*)
		AT1G48130(1.71/0.41*)	AT1G34510(0.94/0.48*)	AT4G33870(1.02/2.97*)	AT3G49110(0.60/2.51*)	AT5G24070(0.81/0.43*)	AT5G14130(0.91/0.37*)

		AT1G09090(1.04/0.64*)	AT1G14540(1.29/0.44*)	AT1G49570(1.42/0.28*)	AT5G06720(1.83/0.13*)	AT3G01190(1.42/3.23*)	AT3G26060(0.72/0.65*)
		AT1G63460(3.45/3.29*)	AT4G31870(0.59/0.33*)	AT5G19880(1.26/0.42*)	AT2G18980(1.38/2.54*)	AT2G24800(0.60/3.36*)	AT5G67400(0.47/0.48*)
		AT4G08780(3.25/0.23*)	AT2G38390(0.56/0.19*)	AT1G60740(3.01/5.18*)	AT2G41480(1.02/0.12*)	AT1G14550(1.12/0.38*)	AT3G52960(0.34/0.35*)
		AT4G25090(2.11/3.81*)					
dioxygenase	20	AT1G17420(0.94/1.15*)	AT4G32810(4.71/3.64*)	AT3G01420(1.61/2.11*)	AT4G19170(2.15/3.66*)	AT2G44990(1.00/0.23*)	AT3G51240(0.42/0.23*)
		AT4G18350(0.58/0.47*)	AT3G13610(1.66/0.47*)	AT1G14120(0.40/0.44*)	AT5G54080(4.55/2.45*)	AT2G30840(0.79/0.43*)	AT3G45140(0.06/0.11*)
		AT1G30040(0.87/0.94*)	AT5G05600(0.54/0.48*)	AT5G54000(3.17/2.09*)	AT5G59530(1.74/4.99*)	AT5G08640(0.14/0.11*)	AT5G59540(6.92/12.68*)
		AT4G22880(1.00/0.23*)	AT4G33910(1.89/1.80*)				
Pyrrolidone_carboxylic_acid	19	AT5G19890(0.96/0.26*)	AT5G19880(1.26/0.42*)	AT5G06730(0.57/0.34*)	AT5G65390(1.49/2.08*)	AT2G22470(2.46/2.02*)	AT2G18980(1.38/2.54*)
		AT2G23130(0.40/0.25*)	AT4G08770(1.68/0.39*)	AT4G08780(3.25/0.23*)	AT5G67400(0.47/0.48*)	AT4G40090(0.75/0.47*)	AT1G34510(0.94/0.48*)
		AT2G38390(0.56/0.19*)	AT3G49110(0.60/2.51*)	AT1G14550(1.12/0.38*)	AT5G14130(0.91/0.37*)	AT1G14540(1.29/0.44*)	AT5G06720(1.83/0.13*)
		AT2G46330(1.92/2.06*)					
leucine-rich_repeat	92	AT3G26920(1.16/1.21*)	AT4G00160(1.01/0.94*)	AT2G26380(0.93/0.26*)	AT1G61190(0.43/0.47*)	AT1G66290(0.85/0.22*)	AT2G32680(0.86/0.71*)
		AT5G06870(0.54/0.29*)	AT3G23110(0.39/0.28*)	AT2G28990(1.31/2.54*)	AT1G33670(0.79/0.36*)	AT5G22610(0.53/0.48*)	AT1G45616(2.51/3.96*)
		AT3G44400(6.94/10.91*)	AT5G48620(15.99/14.59*)	AT1G61310(6.16/49.29*)	AT5G46260(9.81/16.45*)	AT1G59218(61.60/117.99*)	AT1G17250(0.46/0.47*)
		AT1G27190(1.80/2.15*)	AT1G15890(2.21/2.12*)	AT5G47250(0.27/0.22*)	AT1G61180(0.74/0.92*)	AT1G58190(1.80/2.21*)	AT1G50180(0.24/0.35*)
		AT2G40920(3.08/4.76*)	AT5G46490(13.93/7.46*)	AT1G51820(0.38/0.29*)	AT2G34930(0.34/0.30*)	AT4G19050(0.49/0.43*)	AT3G44480(0.34/0.34*)
		AT1G61300(0.33/0.36*)	AT4G16900(1.04/0.85*)	AT1G62630(1.00/13.38*)	AT3G07040(0.50/0.48*)	AT3G46530(30.77/53.76*)	AT1G78230(0.64/0.57*)
		AT1G35710(2.14/2.07*)	AT5G58150(0.53/0.41*)	AT1G51890(1.22/3.57*)	AT1G12210(1.25/7.25*)	AT5G22660(2.82/2.40*)	AT5G36930(3.05/2.28*)
		AT4G16960(71.96/6.89*)	AT5G44980(5.88/5.47*)	AT3G12145(0.93/0.68*)	AT4G16860(1.57/1.40*)	AT1G72840(19.87/16.64*)	AT3G46730(1.11/2.17*)
		AT1G07550(3.13/4.36*)	AT1G58400(5.00/3.77*)	AT5G18360(6.12/3.71*)	AT5G40910(5.49/4.20*)	AT2G13790(27.39/16.44*)	AT1G07560(2.67/2.86*)
		AT1G63360(2.36/2.77*)	AT3G52680(9.60/6.63*)	AT5G45510(1.20/1.28*)	AT4G29180(0.52/0.48*)	AT3G50950(0.55/0.41*)	AT5G58120(0.53/0.22*)
		AT1G13780(1.97/2.51*)	AT1G56540(1.35/2.14*)	AT3G51740(0.73/0.36*)	AT3G13065(0.48/0.40*)	AT2G13800(0.88/0.80*)	AT3G43740(22.70/32.58*)
		AT3G44630(19.64/13.71*)	AT1G80570(2.40/2.17*)	AT5G59680(2.24/3.61*)	AT1G12220(2.01/3.53*)	AT1G58807(92.90/68.68*)	AT5G22700(2.33/2.84*)
		AT3G44670(0.84/0.77*)	AT5G46470(2.45/2.31*)	AT1G63750(1.27/1.51*)	AT5G46330(0.84/2.08*)	AT4G16950(1.15/1.32*)	AT1G59124(7.61/11.45*)
		AT1G59780(3.13/3.29*)	AT1G69545(0.47/0.26*)	AT1G63880(23.34/47.41*)	AT5G49290(0.88/0.96*)	AT1G17240(2.46/2.45*)	AT1G58602(4.40/11.24*)
		AT5G63020(9.47/8.43*)	AT5G35450(2.24/2.37*)	AT1G05700(1.34/2.39*)	AT5G43740(8.34/7.08*)	AT1G51480(0.04/0.06*)	AT5G43470(49.01/45.79*)
		AT2G29910(0.42/0.46*)	AT1G58848(6.64/11.54*)				
Seed_storage_protein	7	AT5G44120(1.51/0.08*)	AT4G27170(0.35/0.14*)	AT1G03880(0.34/0.07*)	AT4G28520(0.97/0.31*)	AT4G27140(0.67/0.04*)	AT4G27150(0.83/0.13*)
		AT4G27160(0.35/0.14*)					
seed	7	AT4G27170(0.35/0.14*)	AT4G25140(0.68/0.10*)	AT1G03880(0.34/0.07*)	AT4G28520(0.97/0.31*)	AT4G27140(0.67/0.04*)	AT4G27150(0.83/0.13*)
		AT4G27160(0.35/0.14*)					
transferase	210	AT4G26740(1.15/0.11*)	AT5G56080(1.13/3.52*)	AT2G14255(2.65/3.32*)	AT3G44990(0.98/5.25*)	AT1G10550(1.01/2.41*)	AT4G23160(1.00/1.00*)
		AT5G57530(0.27/0.23*)	AT5G23580(1.08/0.28*)	AT1G02920(3.39/2.19*)	AT3G29670(6.85/5.09*)	AT3G11430(0.63/0.05*)	AT3G22740(1.58/0.28*)
		AT2G30860(0.64/0.38*)	AT1G72680(2.16/2.10*)	AT4G28850(0.96/0.94*)	AT5G01820(1.27/0.44*)	AT1G78320(0.17/0.16*)	AT5G51690(81.05/183.70*)
		AT4G13460(1.71/2.89*)	AT5G13930(0.14/0.10*)	AT5G43370(1.55/2.53*)	AT5G65550(0.85/0.26*)	AT4G12440(0.61/0.21*)	AT5G61350(0.26/0.43*)
		AT2G13810(1.57/2.05*)	AT4G00040(1.83/2.56*)	AT1G53680(1.06/2.36*)	AT4G26220(0.88/0.46*)	AT5G12210(2.50/2.81*)	AT5G22470(1.18/0.85*)
		AT2G46340(2.02/2.68*)	AT1G56430(0.53/0.18*)	AT1G76790(0.86/0.33*)	AT3G26840(0.24/0.11*)	AT1G77060(3.27/8.23*)	AT4G23290(0.12/0.27*)

		AT2G13800(0.88/0.80*)	AT5G46330(0.84/2.08*)	AT1G68830(0.52/0.44*)	AT1G34520(1.28/3.36*)	AT1G31910(4.13/3.65*)	AT1G16260(7.77/13.20*)
		AT3G32040(0.49/0.48*)	AT1G10060(1.84/2.96*)	AT1G78340(2.19/4.97*)	AT5G59580(1.15/0.44*)	AT3G48830(2.35/4.20*)	AT1G01390(3.10/7.50*)
		AT5G01540(0.29/0.41*)	AT1G04310(2.81/3.80*)	AT4G11460(1.00/11.11*)	AT3G29430(0.21/0.09*)	AT1G28170(0.95/0.36*)	AT4G23260(0.55/0.41*)
		AT2G26420(0.42/0.49*)	AT5G05890(1.81/2.30*)	AT4G27570(0.91/0.47*)	AT3G14510(0.74/0.33*)	AT1G10070(3.23/2.09*)	AT5G28080(0.28/0.45*)
		AT3G16520(0.67/1.04*)	AT5G57560(1.02/0.35*)	AT4G01770(3.07/6.16*)	AT3G23630(2.20/3.29*)	AT1G51820(0.38/0.29*)	AT1G04640(0.38/0.45*)
		AT4G15480(0.11/0.09*)	AT5G19220(2.38/2.29*)	AT4G27560(1.56/2.07*)	AT5G62480(3.61/2.47*)	AT2G30140(0.28/0.40*)	AT5G54160(0.58/0.44*)
		AT5G38100(0.78/0.48*)	AT5G26310(0.52/0.16*)	AT1G07550(3.13/4.36*)	AT4G01950(1.66/2.11*)	AT4G21210(0.72/0.75*)	AT3G27440(5.17/4.29*)
		AT1G17170(1.08/0.45*)	AT3G14530(3.94/3.86*)	AT1G07560(2.67/2.86*)	AT3G45780(2.57/3.01*)	AT4G18990(1.22/0.19*)	AT1G31230(0.57/0.48*)
		AT5G44750(1.87/2.30*)	AT5G52920(1.84/1.88*)	AT4G36450(1.42/2.66*)	AT1G78370(0.18/0.18*)	AT2G15370(0.85/0.47*)	AT4G30280(2.35/2.12*)
		AT3G12780(0.65/0.46*)	AT5G59680(2.24/3.61*)	AT3G18000(0.43/0.35*)	AT3G20160(1.01/0.33*)	AT1G07880(0.65/0.40*)	AT4G25810(1.15/0.43*)
		AT1G17180(1.53/0.41*)	AT1G05700(1.34/2.39*)	AT1G65310(2.77/2.75*)	AT1G78380(1.66/2.58*)	AT3G51630(0.99/1.02*)	AT2G16890(0.03/0.03*)
		AT3G07020(0.33/0.39*)	AT1G28680(0.71/0.38*)	AT1G58080(0.32/0.37*)	AT2G28990(1.31/2.54*)	AT5G37950(0.43/0.26*)	AT3G19710(0.31/0.05*)
		AT1G60940(2.38/2.77*)	AT1G64910(0.64/0.35*)	AT4G13890(0.94/0.20*)	AT4G39980(0.75/0.41*)	AT1G17190(1.07/0.20*)	AT5G23010(0.76/0.44*)
		AT1G66340(1.43/2.07*)	AT5G03490(3.90/5.30*)	AT4G04570(0.60/0.32*)	AT5G17220(2.21/2.03*)	AT4G17360(0.44/0.26*)	AT1G10210(2.66/2.48*)
		AT5G15380(1.28/2.12*)	AT4G02130(0.53/0.49*)	AT2G15090(1.00/1.83*)	AT1G64920(0.80/0.48*)	AT1G18590(0.74/0.23*)	AT1G71990(0.50/0.48*)
		AT1G50090(0.92/2.43*)	AT3G02020(0.24/0.05*)	AT4G23220(6.85/5.09*)	AT5G63560(0.97/0.28*)	AT1G35710(2.14/2.07*)	AT5G47070(1.77/2.70*)
		AT4G23600(0.15/0.02*)	AT3G45080(1.05/0.40*)	AT1G06520(0.38/0.38*)	AT2G19640(10.94/14.39*)	AT4G14580(0.50/0.26*)	AT2G13790(27.39/16.44*)
		AT4G02520(1.34/2.68*)	AT5G67160(0.81/0.46*)	AT4G23210(0.35/0.22*)	AT2G29440(0.63/0.22*)	AT3G46680(0.79/0.32*)	AT2G25090(0.59/0.44*)
		AT5G43910(2.25/2.17*)	AT3G03190(0.41/0.12*)	AT3G28340(0.53/0.51*)	AT1G48260(2.01/2.37*)	AT1G73880(2.22/5.45*)	AT1G48600(0.91/0.31*)
		AT1G62800(0.59/0.33*)	AT5G43780(0.84/0.67*)	AT4G04670(2.25/2.76*)	AT4G15320(1.00/0.28*)	AT1G10370(0.44/0.38*)	AT2G18950(0.53/0.50*)
		AT5G17050(0.44/0.42*)	AT4G39940(0.70/0.41*)	AT2G29470(0.96/0.21*)	AT4G15550(1.51/1.99*)	AT1G13430(11.44/33.42*)	AT2G31790(0.34/0.39*)
		AT5G20250(1.92/2.07*)	AT3G45070(1.36/2.35*)	AT3G03780(0.96/0.67*)	AT5G41820(0.20/0.10*)	AT2G22930(1.33/0.11*)	AT5G52470(0.96/1.35*)
		AT2G29480(2.87/2.48*)	AT4G15270(0.70/0.28*)	AT1G05560(2.27/2.01*)	AT1G18690(1.46/1.30*)	AT2G38620(5.68/2.97*)	AT5G49690(1.43/2.51*)
		AT5G43760(1.75/1.35*)	AT2G24850(1.11/0.01*)	AT3G59480(3.42/3.10*)	AT1G51890(1.22/3.57*)	AT1G55740(1.76/2.35*)	AT5G48300(0.50/0.46*)
		AT2G18570(0.73/0.46*)	AT2G29490(1.80/2.94*)	AT1G22370(0.62/0.62*)	AT5G43690(0.92/0.21*)	AT2G29730(2.10/2.02*)	AT5G55360(0.02/0.04*)
		AT1G27130(1.43/1.81*)	AT5G38010(0.48/0.40*)	AT3G11240(2.94/2.63*)	AT2G18170(1.20/1.63*)	AT4G04740(1.14/1.10*)	AT5G36870(0.75/0.71*)
		AT4G29180(0.52/0.48*)	AT2G02500(0.49/0.44*)	AT5G17990(1.48/0.57*)	AT2G02390(2.06/2.03*)	AT1G06020(1.05/3.68*)	AT2G18800(0.44/0.29*)
		AT5G04950(0.86/0.49*)	AT1G79000(3.20/3.85*)	AT3G29635(0.41/0.07*)	AT4G11610(1.18/2.10*)	AT4G13410(0.86/0.43*)	AT3G21560(0.21/0.33*)
		AT4G14140(1.00/4.88*)	AT3G50300(0.66/0.37*)	AT4G34650(1.84/2.31*)	AT1G05680(2.24/4.32*)	AT5G53970(2.56/2.21*)	AT5G55370(1.37/3.11*)
metalloprotein	35	AT2G34770(2.47/1.86*)	AT2G02580(1.56/2.01*)	AT3G52780(4.27/2.64*)	AT1G64950(102.53/150.78*)	AT4G13770(0.05/0.06*)	AT2G46650(0.26/0.21*)
		AT5G05690(1.92/2.08*)	AT3G49110(0.60/2.51*)	AT2G12190(8.79/9.34*)	AT2G46950(0.34/0.26*)	AT1G77120(1.20/3.06*)	AT4G29690(0.96/0.17*)
		AT2G27010(0.37/0.21*)	AT3G26280(1.23/2.06*)	AT2G30750(2.04/2.02*)	AT2G14100(63.98/5.82*)	AT2G01880(0.89/0.11*)	AT3G10920(0.97/0.92*)
		AT3G26220(2.15/2.57*)	AT3G51240(0.42/0.23*)	AT4G36220(0.28/0.20*)	AT2G18980(1.38/2.54*)	AT2G07727(0.60/0.40*)	AT3G52820(0.63/0.45*)
		AT3G48320(0.68/0.45*)	AT3G61040(1.00/0.16*)	AT2G38390(0.56/0.19*)	AT3G28740(2.62/11.74*)	AT3G56350(0.44/0.11*)	AT1G64940(5.46/5.50*)
		AT4G37970(0.73/0.32*)	AT1G14700(0.61/0.50*)	AT4G12330(55.28/68.16*)	AT4G22690(1.06/1.16*)	AT4G22880(1.00/0.23*)	
nadp	33	AT3G44560(4.03/4.79*)	AT5G07390(1.00/1.00*)	AT5G39190(68.20/51.10*)	AT3G19270(1.55/2.57*)	AT3G44540(0.56/0.21*)	AT1G16410(0.52/0.25*)
		AT3G56700(1.67/2.34*)	AT1G19250(8.13/2.01*)	AT3G61220(0.41/0.39*)	AT5G22500(4.94/3.47*)	AT1G17990(1.65/2.62*)	AT5G05260(1.03/0.44*)
		AT4G33790(0.86/0.45*)	AT1G31230(0.57/0.48*)	AT3G59890(1.88/1.78*)	AT1G20020(0.59/0.45*)	AT1G09090(1.04/0.64*)	AT1G72680(2.16/2.10*)

		AT1G43800(1.64/1.82*)	AT2G22330(0.42/0.34*)	AT3G44550(0.36/0.30*)	AT4G39950(0.80/0.34*)	AT1G72610(0.38/0.25*)	AT4G27440(1.24/2.26*)
		AT1G17890(1.45/1.41*)	AT1G18020(1.65/2.40*)	AT1G16400(0.67/0.25*)	AT4G37970(0.73/0.32*)	AT1G09420(0.32/0.26*)	AT2G21890(2.23/2.42*)
		AT5G54190(1.82/2.60*)	AT4G25090(2.11/3.81*)	AT1G76680(1.92/3.18*)			
lipid_degradation	31	AT1G53990(2.48/4.16*)	AT1G54000(1.16/0.48*)	AT3G16370(3.80/2.81*)	AT4G16230(1.77/3.07*)	AT2G38180(1.51/2.16*)	AT2G26560(0.82/0.43*)
		AT1G54020(0.72/0.07*)	AT3G14220(4.49/7.00*)	AT1G75910(0.53/0.48*)	AT3G55940(0.56/2.37*)	AT4G18970(0.92/0.98*)	AT5G45960(0.43/0.45*)
		AT5G03810(0.29/0.14*)	AT1G54030(0.72/0.43*)	AT1G18120(1.00/0.15*)	AT3G47290(6.87/6.07*)	AT2G24560(0.94/0.36*)	AT1G31550(0.34/0.39*)
		AT2G42990(4.59/4.60*)	AT3G14210(4.68/10.72*)	AT3G11210(48.15/29.70*)	AT3G04290(0.42/0.12*)	AT1G28660(1.62/3.20*)	AT5G03820(0.75/0.32*)
		AT3G47220(3.82/2.44*)	AT5G43590(0.88/0.27*)	AT5G33370(0.83/0.81*)	AT4G11840(1.09/0.82*)	AT5G55050(1.02/2.55*)	AT3G62280(3.88/2.74*)
		AT1G28580(1.92/2.28*)					
alternative_splicing	182	AT5G24150(0.37/0.31*)	AT1G20450(1.39/1.91*)	AT3G02870(0.66/0.57*)	AT1G52400(0.10/0.10*)	AT1G66280(0.91/0.40*)	AT1G54040(1.00/0.04*)
		AT2G22980(0.72/0.49*)	AT3G61390(0.84/3.13*)	AT4G16845(1.14/1.11*)	AT2G27810(1.10/1.23*)	AT5G13220(0.86/0.35*)	AT1G45249(76.99/43.96*)
		AT3G16150(0.51/0.49*)	AT4G36060(1.73/5.09*)	AT4G39260(3.03/2.39*)	AT4G04570(0.60/0.32*)	AT4G40040(1.92/2.63*)	AT1G09090(1.04/0.64*)
		AT1G66200(0.92/0.38*)	AT1G18250(0.60/0.34*)	AT5G38970(1.66/2.81*)	AT5G39160(16.59/25.74*)	AT2G23310(1.49/1.76*)	AT5G64520(1.43/1.68*)
		AT5G14740(3.15/3.16*)	AT1G16070(0.40/0.35*)	AT5G61530(3.53/3.29*)	AT4G08870(0.50/0.36*)	AT5G20730(1.06/1.07*)	AT4G23220(6.85/5.09*)
		AT1G68910(2.36/2.11*)	AT1G24490(2.24/2.45*)	AT5G43370(1.55/2.53*)	AT5G25980(13.03/33.60*)	AT1G35710(2.14/2.07*)	AT4G05320(2.25/1.94*)
		AT5G22660(2.82/2.40*)	AT5G39190(68.20/51.10*)	AT3G08940(1.53/1.79*)	AT1G65800(1.06/0.85*)	AT1G49960(0.57/0.33*)	AT3G44540(0.56/0.21*)
		AT2G19640(10.94/14.39*)	AT3G56700(1.67/2.34*)	AT4G31800(0.53/0.45*)	AT2G41240(1.00/0.24*)	AT4G33010(0.59/0.43*)	AT1G17990(1.65/2.62*)
		AT5G45940(1.47/2.46*)	AT1G59640(2.39/2.68*)	AT3G18520(2.12/2.63*)	AT1G20020(0.59/0.45*)	AT1G74710(0.55/0.34*)	AT4G23210(0.35/0.22*)
		AT5G20620(0.73/0.68*)	AT2G01200(1.20/2.88*)	AT1G53790(1.75/3.02*)	AT4G23290(0.12/0.27*)	AT3G57210(1.61/4.11*)	AT1G80570(2.40/2.17*)
		AT5G66400(9.77/0.28*)	AT3G45960(1.01/0.68*)	AT1G80960(6.94/9.51*)	AT1G59124(7.61/11.45*)	AT1G62800(0.59/0.33*)	AT1G48600(0.91/0.31*)
		AT1G52150(2.63/2.63*)	AT4G29930(0.51/0.35*)	AT1G30040(0.87/0.94*)	AT3G52720(0.58/1.77*)	AT1G10060(1.84/2.96*)	AT3G56770(3.38/2.54*)
		AT5G54190(1.82/2.60*)	AT1G76680(1.92/3.18*)	AT2G46330(1.92/2.06*)	AT5G43470(49.01/45.79*)	AT2G24190(0.72/0.74*)	AT2G23240(21.70/0.16*)
		AT2G12480(0.12/0.19*)	AT3G20270(0.61/0.46*)	AT4G13235(0.98/0.36*)	AT5G14200(0.56/0.41*)	AT2G33340(2.23/2.83*)	AT3G58810(0.80/0.40*)
		AT4G13850(0.15/0.15*)	AT1G76930(2.06/3.56*)	AT2G39730(0.48/0.45*)	AT1G24260(0.24/0.35*)	AT5G05690(1.92/2.08*)	AT1G66610(0.17/0.22*)
		AT5G24780(1.03/0.15*)	AT5G65050(19.48/27.26*)	AT3G59890(1.88/1.78*)	AT1G27190(1.80/2.15*)	AT2G22540(2.49/4.10*)	AT3G26300(50.20/12.69*)
		AT1G02850(1.79/2.35*)	AT1G10070(3.23/2.09*)	AT3G62090(2.19/2.85*)	AT2G03710(40.60/37.25*)	AT1G05200(2.35/2.96*)	AT2G15050(12.90/17.49*)
		AT1G61180(0.74/0.92*)	AT5G44120(1.51/0.08*)	AT5G28080(0.28/0.45*)	AT4G14410(1.20/1.47*)	AT5G10140(19.31/28.22*)	AT2G40920(3.08/4.76*)
		AT3G10920(0.97/0.92*)	AT2G46450(5.61/8.00*)	AT1G31550(0.34/0.39*)	AT3G20970(1.99/2.35*)	AT5G52470(0.96/1.35*)	AT5G40780(1.90/3.57*)
		AT1G17960(2.49/2.98*)	AT4G19850(0.49/0.35*)	AT2G35635(11.46/11.45*)	AT1G23970(2.12/2.99*)	AT1G49130(1.72/1.75*)	AT3G01500(2.67/2.48*)
		AT5G24770(1.44/0.89*)	AT2G44480(1.36/2.39*)	AT3G17950(5.47/7.04*)	AT3G46320(0.28/0.26*)	AT1G14700(0.61/0.50*)	AT2G38620(5.68/2.97*)
		AT3G10450(2.21/2.00*)	AT5G62890(0.44/0.46*)	AT5G05410(1.46/1.76*)	AT4G04750(0.12/0.39*)	AT1G28580(1.92/2.28*)	AT3G62030(0.49/0.49*)
		AT1G66570(0.58/0.38*)	AT1G51890(1.22/3.57*)	AT3G52780(4.27/2.64*)	AT1G11270(1.68/2.19*)	AT5G38100(0.78/0.48*)	AT2G44140(1.11/1.13*)
		AT2G39470(0.36/0.41*)	AT2G01660(1.54/2.06*)	AT1G54020(0.72/0.07*)	AT1G16410(0.52/0.25*)	AT1G25054(6.37/6.65*)	AT2G22920(1.01/0.76*)
		AT4G21210(0.72/0.75*)	AT3G58780(1.38/3.83*)	AT1G24880(6.03/7.30*)	AT3G55940(0.56/2.37*)	AT5G23810(0.71/0.32*)	AT5G14610(1.63/1.35*)
		AT4G01540(0.66/0.41*)	AT5G45510(1.20/1.28*)	AT5G02500(1.02/0.96*)	AT2G02390(2.06/2.03*)	AT4G15210(0.89/0.47*)	AT3G14230(1.11/1.41*)
		AT4G13310(1.42/0.33*)	AT1G63940(0.69/0.61*)	AT1G65390(0.33/0.29*)	AT1G58807(92.90/68.68*)	AT3G48380(2.64/2.89*)	AT4G19690(0.74/0.06*)
		AT3G18500(32.47/71.98*)	AT2G23130(0.40/0.25*)	AT1G07880(0.65/0.40*)	AT1G17890(1.45/1.41*)	AT1G18020(1.65/2.40*)	AT1G34760(5.45/4.08*)
		AT5G01240(1.41/1.84*)	AT1G28960(1.05/1.38*)	AT4G17730(1.67/2.12*)	AT1G65060(1.23/0.93*)	AT1G28660(1.62/3.20*)	AT5G52300(1.39/0.38*)

		AT3G18550(0.87/0.89*)	AT4G19680(0.51/0.18*)	AT2G40430(2.11/2.25*)	AT2G47160(1.01/0.70*)	AT5G33370(0.83/0.81*)	AT5G09820(0.42/0.42*)
		AT4G18490(2.00/8.08*)	AT2G29910(0.42/0.46*)				
hydrogen_peroxide	21	AT5G19890(0.96/0.26*)	AT5G64100(2.27/5.21*)	AT5G19880(1.26/0.42*)	AT5G05340(0.20/0.06*)	AT5G06730(0.57/0.34*)	AT2G18980(1.38/2.54*)
		AT2G24800(0.60/3.36*)	AT4G08770(1.68/0.39*)	AT4G08780(3.25/0.23*)	AT5G67400(0.47/0.48*)	AT1G34510(0.94/0.48*)	AT4G33870(1.02/2.97*)
		AT2G38390(0.56/0.19*)	AT2G41480(1.02/0.12*)	AT3G49110(0.60/2.51*)	AT5G24070(0.81/0.43*)	AT5G14130(0.91/0.37*)	AT1G14540(1.29/0.44*)
		AT1G49570(1.42/0.28*)	AT5G06720(1.83/0.13*)	AT3G01190(1.42/3.23*)			
cell_wall	31	AT5G05290(0.40/2.19*)	AT5G06870(0.54/0.29*)	AT1G53830(0.99/0.48*)	AT3G44990(0.98/5.25*)	AT2G37640(1.11/2.12*)	AT3G29030(1.36/1.16*)
		AT1G76930(2.06/3.56*)	AT4G12420(2.97/2.94*)	AT2G47550(0.67/0.31*)	AT1G10550(1.01/2.41*)	AT5G57530(0.27/0.23*)	AT5G43980(1.03/2.50*)
		AT3G06830(1.66/0.50*)	AT4G18990(1.22/0.19*)	AT2G43860(0.29/0.33*)	AT2G19150(2.65/2.30*)	AT4G02320(0.64/0.44*)	AT1G20190(1.44/2.07*)
		AT2G18800(0.44/0.29*)	AT4G30280(2.35/2.12*)	AT4G28850(0.96/0.94*)	AT5G57560(1.02/0.35*)	AT1G32860(0.26/0.53*)	AT1G11580(0.71/0.33*)
		AT4G25810(1.15/0.43*)	AT1G65310(2.77/2.75*)	AT1G11370(0.05/0.05*)	AT3G55500(1.00/0.92*)	AT2G26440(0.30/0.34*)	AT4G02330(2.46/2.03*)
		AT5G56320(1.08/0.37*)					
Phenylpropanoid_metabolism	6	AT5G17050(0.44/0.42*)	AT1G65060(1.23/0.93*)	AT3G21240(1.04/0.48*)	AT2G37040(0.92/0.49*)	AT5G04230(0.46/0.40*)	AT3G21230(0.94/0.33*)
apoplast	23	AT2G18800(0.44/0.29*)	AT5G20630(1.47/4.16*)	AT5G38940(0.94/0.44*)	AT2G29130(2.65/2.01*)	AT4G30280(2.35/2.12*)	AT5G38930(1.09/0.42*)
		AT4G28850(0.96/0.94*)	AT1G18140(0.61/0.14*)	AT3G05950(2.20/0.01*)	AT1G18980(0.51/0.49*)	AT5G57560(1.02/0.35*)	AT5G39190(68.20/51.10*)
		AT3G44990(0.98/5.25*)	AT1G72610(0.38/0.25*)	AT1G10550(1.01/2.41*)	AT5G57530(0.27/0.23*)	AT1G17890(1.45/1.41*)	AT4G25810(1.15/0.43*)
		AT5G20710(39.08/10.61*)	AT2G38080(1.41/2.19*)	AT4G18990(1.22/0.19*)	AT5G39130(9.24/11.30*)	AT1G65310(2.77/2.75*)	
glycosidase	48	AT4G01700(0.79/0.39*)	AT5G25980(13.03/33.60*)	AT1G52400(0.10/0.10*)	AT1G66280(0.91/0.40*)	AT4G27830(1.33/2.60*)	AT1G60270(0.27/0.30*)
		AT1G02310(0.94/0.49*)	AT3G44990(0.98/5.25*)	AT4G17090(0.53/0.45*)	AT1G10550(1.01/2.41*)	AT5G57530(0.27/0.23*)	AT3G62750(1.64/2.24*)
		AT3G21370(0.44/0.10*)	AT5G55180(0.28/0.19*)	AT2G44450(0.64/0.35*)	AT4G18990(1.22/0.19*)	AT3G04010(2.19/2.39*)	AT4G23560(0.78/2.68*)
		AT2G43860(0.29/0.33*)	AT1G58370(2.69/2.71*)	AT1G64390(1.47/1.15*)	AT1G61810(0.54/0.28*)	AT1G02850(1.79/2.35*)	AT1G02460(0.87/0.46*)
		AT5G24550(1.00/0.41*)	AT1G12240(0.76/0.48*)	AT4G15210(0.89/0.47*)	AT2G18800(0.44/0.29*)	AT4G30280(2.35/2.12*)	AT4G16260(2.34/2.41*)
		AT4G28850(0.96/0.94*)	AT5G57560(1.02/0.35*)	AT2G43610(2.43/2.06*)	AT1G32860(0.26/0.53*)	AT1G51470(0.78/0.30*)	AT1G60590(0.22/0.17*)
		AT4G02290(1.20/1.39*)	AT1G47600(0.80/0.37*)	AT4G25810(1.15/0.43*)	AT5G20710(39.08/10.61*)	AT2G44460(1.19/2.53*)	AT5G28510(1.26/6.24*)
		AT2G44480(1.36/2.39*)	AT3G60140(4.30/3.53*)	AT1G65310(2.77/2.75*)	AT3G61490(0.44/0.47*)	AT3G12710(1.58/1.99*)	AT1G10640(0.39/0.28*)
lipid_synthesis	20	AT1G43800(1.64/1.82*)	AT3G44550(0.36/0.30*)	AT1G17420(0.94/1.15*)	AT3G44560(4.03/4.79*)	AT1G65290(14.54/9.93*)	AT3G44540(0.56/0.21*)
		AT3G56700(1.67/2.34*)	AT1G34520(1.28/3.36*)	AT1G25054(6.37/6.65*)	AT5G05580(0.38/0.27*)	AT5G22500(4.94/3.47*)	AT1G24880(6.03/7.30*)
		AT3G45140(0.06/0.11*)	AT4G33790(0.86/0.45*)	AT5G55360(0.02/0.04*)	AT5G27200(0.14/0.19*)	AT5G42650(0.50/0.33*)	AT3G12120(1.81/2.25*)
		AT1G76680(1.92/3.18*)	AT5G55370(1.37/3.11*)				
hydro-lyase	5	AT3G01500(2.67/2.48*)	AT3G52720(0.58/1.77*)	AT3G54640(1.10/0.41*)	AT4G27070(0.34/0.23*)	AT1G52410(0.44/0.11*)	
lipid_metabolism	9	AT5G22500(4.94/3.47*)	AT3G44550(0.36/0.30*)	AT4G33790(0.86/0.45*)	AT3G44560(4.03/4.79*)	AT5G55360(0.02/0.04*)	AT3G44540(0.56/0.21*)
		AT3G56700(1.67/2.34*)	AT1G34520(1.28/3.36*)	AT5G55370(1.37/3.11*)			
ubl_conjugation	17	AT5G17490(1.05/0.49*)	AT4G05320(2.25/1.94*)	AT3G27810(0.91/0.31*)	AT1G58807(92.90/68.68*)	AT5G46330(0.84/2.08*)	AT1G59124(7.61/11.45*)
		AT5G13220(0.86/0.35*)	AT4G25560(1.52/2.45*)	AT2G35635(11.46/11.45*)	AT1G72450(0.14/0.36*)	AT2G34600(0.68/0.37*)	AT1G54280(1.69/2.17*)
		AT3G09480(0.39/0.42*)	AT2G30950(1.82/2.14*)	AT1G30135(1.00/0.14*)	AT5G20620(0.73/0.68*)	AT2G36490(1.58/1.73*)	
flavonoid_biosynthesis	6	AT3G51240(0.42/0.23*)	AT2G47460(0.33/0.23*)	AT3G55120(0.27/0.30*)	AT5G08640(0.14/0.11*)	AT5G13930(0.14/0.10*)	AT4G22880(1.00/0.23*)
lyase	30	AT3G44720(0.71/0.47*)	AT4G26530(3.13/2.10*)	AT5G22630(0.94/0.45*)	AT4G18440(0.60/0.53*)	AT2G37040(0.92/0.49*)	AT5G04230(0.46/0.40*)
		AT4G37150(0.44/0.47*)	AT2G23550(1.00/0.33*)	AT1G11840(0.72/0.57*)	AT2G23560(1.24/0.25*)	AT1G16540(2.12/2.11*)	AT3G23490(2.71/1.96*)

		AT5G04310(2.01/2.57*)	AT1G48605(0.27/0.45*)	AT3G11750(0.44/0.41*)	AT5G14740(3.15/3.16*)	AT5G38420(0.89/0.78*)	AT1G52410(0.44/0.11*)
		AT5G54810(0.69/0.49*)	AT5G63180(1.20/2.66*)	AT3G01500(2.67/2.48*)	AT4G37770(1.16/2.71*)	AT3G52720(0.58/1.77*)	AT1G08250(0.38/0.33*)
		AT3G54640(1.10/0.41*)	AT5G42650(0.50/0.33*)	AT5G38430(0.82/0.69*)	AT5G51930(0.31/0.39*)	AT5G15950(0.29/0.46*)	AT3G15620(4.14/4.19*)
hydrolase	180	AT2G19570(0.79/0.48*)	AT1G49630(2.10/2.92*)	AT3G02870(0.66/0.57*)	AT1G04110(0.21/0.48*)	AT1G52400(0.10/0.10*)	AT1G66280(0.91/0.40*)
		AT2G22980(0.72/0.49*)	AT3G44300(1.89/2.15*)	AT4G11320(6.67/12.10*)	AT5G64000(0.78/0.23*)	AT3G52500(0.46/0.45*)	AT3G44990(0.98/5.25*)
		AT1G60270(0.27/0.30*)	AT2G26560(0.82/0.43*)	AT2G47550(0.67/0.31*)	AT3G14220(4.49/7.00*)	AT1G10550(1.01/2.41*)	AT5G57530(0.27/0.23*)
		AT5G15250(1.63/5.58*)	AT5G02760(1.14/2.39*)	AT3G62750(1.64/2.24*)	AT1G75910(0.53/0.48*)	AT5G63980(0.53/0.45*)	AT3G16150(0.51/0.49*)
		AT2G30660(0.73/0.38*)	AT5G09650(0.53/0.49*)	AT4G23560(0.78/2.68*)	AT1G44350(0.35/0.48*)	AT5G03810(0.29/0.14*)	AT4G04910(0.90/1.07*)
		AT2G36490(1.58/1.73*)	AT1G12240(0.76/0.48*)	AT1G54030(0.72/0.43*)	AT3G49360(1.90/2.86*)	AT4G36880(0.45/3.97*)	AT4G28850(0.96/0.94*)
		AT4G16260(2.34/2.41*)	AT2G35770(0.77/0.25*)	AT4G20070(0.17/0.18*)	AT2G43610(2.43/2.06*)	AT5G50260(7.95/4.15*)	AT3G02410(0.84/0.46*)
		AT1G51470(0.78/0.30*)	AT3G14210(4.68/10.72*)	AT4G08870(0.50/0.36*)	AT5G43590(0.88/0.27*)	AT1G14240(1.22/0.70*)	AT4G11840(1.09/0.82*)
		AT1G11370(0.05/0.05*)	AT4G02330(2.46/2.03*)	AT2G26440(0.30/0.34*)	AT1G73280(0.35/0.31*)	AT1G53990(2.48/4.16*)	AT5G25980(13.03/33.60*)
		AT4G01700(0.79/0.39*)	AT4G16230(1.77/3.07*)	AT2G38180(1.51/2.16*)	AT1G20160(4.11/2.11*)	AT4G15100(0.86/0.15*)	AT5G21950(0.91/0.58*)
		AT5G45940(1.47/2.46*)	AT3G18520(2.12/2.63*)	AT4G02320(0.64/0.44*)	AT1G02460(0.87/0.46*)	AT2G26450(0.36/0.24*)	AT3G47290(6.87/6.07*)
		AT2G18130(0.81/0.48*)	AT3G47010(2.89/2.33*)	AT3G51340(1.50/3.17*)	AT4G11310(6.44/5.46*)	AT2G24560(0.94/0.36*)	AT3G48340(0.88/0.44*)
		AT3G09960(1.34/2.05*)	AT1G70170(2.08/2.50*)	AT4G02290(1.20/1.39*)	AT5G20710(39.08/10.61*)	AT3G11210(48.15/29.70*)	AT3G52720(0.58/1.77*)
		AT3G62280(3.88/2.74*)	AT4G04460(1.56/2.27*)	AT2G12480(0.12/0.19*)	AT3G48350(0.42/0.38*)	AT5G59220(1.36/2.48*)	AT1G51380(0.41/0.47*)
		AT1G54000(1.16/0.48*)	AT4G16190(2.33/1.87*)	AT1G53830(0.99/0.48*)	AT4G27830(1.33/2.60*)	AT1G02310(0.94/0.49*)	AT2G07680(0.50/0.44*)
		AT5G55180(0.28/0.19*)	AT3G06830(1.66/0.50*)	AT2G19150(2.65/2.30*)	AT4G18970(0.92/0.98*)	AT1G58370(2.69/2.71*)	AT3G21250(1.85/2.25*)
		AT5G45960(0.43/0.45*)	AT1G61810(0.54/0.28*)	AT1G02850(1.79/2.35*)	AT3G12220(2.06/2.25*)	AT5G57560(1.02/0.35*)	AT2G01880(0.89/0.11*)
		AT1G31550(0.34/0.39*)	AT2G47800(0.37/0.47*)	AT1G47380(2.56/2.34*)	AT1G60590(0.22/0.17*)	AT1G11580(0.71/0.33*)	AT1G47600(0.80/0.37*)
		AT3G04290(0.42/0.12*)	AT3G60160(0.63/0.44*)	AT5G28510(1.26/6.24*)	AT3G54940(1.52/0.44*)	AT1G54280(1.69/2.17*)	AT3G60140(4.30/3.53*)
		AT2G44480(1.36/2.39*)	AT1G19670(0.31/0.21*)	AT3G47220(3.82/2.44*)	AT3G13090(0.40/0.23*)	AT1G14700(0.61/0.50*)	AT3G61490(0.44/0.47*)
		AT3G44750(0.51/0.53*)	AT2G30950(1.82/2.14*)	AT3G10450(2.21/2.00*)	AT5G55050(1.02/2.55*)	AT1G28580(1.92/2.28*)	AT2G02990(1.24/4.93*)
		AT2G32960(0.61/0.50*)	AT3G16370(3.80/2.81*)	AT2G44140(1.11/1.13*)	AT1G65240(0.17/0.17*)	AT2G35840(0.62/0.54*)	AT4G17090(0.53/0.45*)
		AT1G54020(0.72/0.07*)	AT2G43660(3.08/3.96*)	AT1G25054(6.37/6.65*)	AT2G22920(1.01/0.76*)	AT3G21370(0.44/0.10*)	AT1G24880(6.03/7.30*)
		AT3G24360(0.81/0.42*)	AT4G37270(26.36/27.90*)	AT3G55940(0.56/2.37*)	AT2G44450(0.64/0.35*)	AT4G18990(1.22/0.19*)	AT3G04010(2.19/2.39*)
		AT2G43860(0.29/0.33*)	AT5G14610(1.63/1.35*)	AT1G64390(1.47/1.15*)	AT4G29690(0.96/0.17*)	AT4G30610(0.48/0.36*)	AT4G30810(0.79/0.39*)
		AT5G24550(1.00/0.41*)	AT4G15210(0.89/0.47*)	AT2G18800(0.44/0.29*)	AT1G18120(1.00/0.15*)	AT4G30280(2.35/2.12*)	AT3G26690(2.39/3.34*)
		AT1G06550(0.70/0.40*)	AT2G22310(3.65/3.48*)	AT3G48380(2.64/2.89*)	AT1G75630(2.01/2.09*)	AT1G32860(0.26/0.53*)	AT1G79330(0.19/0.14*)
		AT3G18500(32.47/71.98*)	AT2G42990(4.59/4.60*)	AT3G52820(0.63/0.45*)	AT1G16420(0.35/0.48*)	AT3G59140(0.96/2.55*)	AT2G44460(1.19/2.53*)
		AT4G25810(1.15/0.43*)	AT1G28960(1.05/1.38*)	AT3G16190(1.56/2.06*)	AT1G28660(1.62/3.20*)	AT5G03820(0.75/0.32*)	AT1G43780(0.24/0.15*)
		AT1G65310(2.77/2.75*)	AT3G20210(1.57/2.79*)	AT5G33370(0.83/0.81*)	AT5G24420(0.35/0.17*)	AT1G79890(0.40/0.42*)	AT1G10640(0.39/0.28*)
Symport	15	AT5G26250(1.00/0.32*)	AT1G66570(0.58/0.38*)	AT5G09220(2.19/8.05*)	AT3G51895(1.76/0.44*)	AT1G71880(0.57/0.38*)	AT5G40780(1.90/3.57*)
		AT1G34580(0.31/0.22*)	AT1G71890(0.46/0.32*)	AT5G19600(0.23/0.32*)	AT4G21480(3.20/4.39*)	AT5G01240(1.41/1.84*)	AT5G10180(1.13/0.44*)
		AT5G23810(0.71/0.32*)	AT5G43350(1.83/5.10*)	AT5G43370(1.55/2.53*)			
cysteine_proteinase	6	AT3G48350(0.42/0.38*)	AT4G36880(0.45/3.97*)	AT4G11310(6.44/5.46*)	AT4G16190(2.33/1.87*)	AT4G11320(6.67/12.10*)	AT3G48340(0.88/0.44*)
chlorophyll	6	AT1G29910(0.64/0.40*)	AT1G29920(0.59/0.47*)	AT3G47470(1.16/1.08*)	AT1G29930(1.12/0.89*)	AT2G34420(1.06/0.83*)	AT3G08940(1.53/1.79*)

repressor	20	AT4G14550(0.36/0.28*)	AT5G44210(1.35/2.58*)	AT1G03800(1.11/2.02*)	AT5G10140(19.31/28.22*)	AT5G17490(1.05/0.49*)	AT3G17600(0.87/0.46*)
		AT2G37120(0.76/0.87*)	AT4G16845(1.14/1.11*)	AT3G44530(1.86/2.48*)	AT4G32280(1.51/2.77*)	AT5G65050(19.48/27.26*)	AT2G46990(1.85/3.80*)
		AT1G28370(0.48/0.47*)	AT4G09460(0.57/0.46*)	AT3G18520(2.12/2.63*)	AT3G44750(0.51/0.53*)	AT3G23030(2.42/3.01*)	AT3G53370(3.02/2.13*)
		AT2G01200(1.20/2.88*)	AT1G51950(1.45/1.50*)				
proteoglycan	11	AT2G47930(1.32/2.12*)	AT5G06390(0.07/0.05*)	AT5G65390(1.49/2.08*)	AT3G52370(1.04/0.59*)	AT5G44130(0.39/0.27*)	AT5G40730(1.89/2.73*)
		AT2G22470(2.46/2.02*)	AT5G56540(1.95/2.19*)	AT2G23130(0.40/0.25*)	AT2G46330(1.92/2.06*)	AT4G40090(0.75/0.47*)	
hydroxylation	6	AT5G65390(1.49/2.08*)	AT3G47295(0.61/0.63*)	AT2G22470(2.46/2.02*)	AT5G56540(1.95/2.19*)	AT1G76930(2.06/3.56*)	AT2G46330(1.92/2.06*)
Aminotransferase	10	AT1G50090(0.92/2.43*)	AT5G51690(81.05/183.70*)	AT2G13810(1.57/2.05*)	AT1G10060(1.84/2.96*)	AT4G23600(0.15/0.02*)	AT3G19710(0.31/0.05*)
		AT1G10070(3.23/2.09*)	AT5G53970(2.56/2.21*)	AT2G24850(1.11/0.01*)	AT1G62800(0.59/0.33*)		
phospholipid_biosynthesis	7	AT4G26740(1.15/0.11*)	AT1G06520(0.38/0.38*)	AT3G18000(0.43/0.35*)	AT3G11430(0.63/0.05*)	AT4G01950(1.66/2.11*)	AT1G48600(0.91/0.31*)
		AT4G39800(3.04/2.19*)					
carboxypeptidase	12	AT2G12480(0.12/0.19*)	AT2G22980(0.72/0.49*)	AT2G35770(0.77/0.25*)	AT4G15100(0.86/0.15*)	AT1G43780(0.24/0.15*)	AT4G30610(0.48/0.36*)
		AT5G22860(3.92/2.89*)	AT4G30810(0.79/0.39*)	AT3G10450(2.21/2.00*)	AT3G12220(2.06/2.25*)	AT1G73280(0.35/0.31*)	AT2G22920(1.01/0.76*)
heat_shock	4	AT4G10250(2.81/3.89*)	AT4G25200(0.90/2.83*)	AT1G53540(0.58/3.52*)	AT3G12580(1.18/2.02*)		
membrane	273	AT5G24150(0.37/0.31*)	AT3G47470(1.16/1.08*)	AT2G42360(0.57/0.54*)	AT1G80830(2.15/2.43*)	AT2G14255(2.65/3.32*)	AT1G80760(21.49/15.26*)
		AT4G23160(1.00/1.00*)	AT3G11430(0.63/0.05*)	AT5G24160(0.22/0.23*)	AT2G39920(0.95/2.25*)	AT5G40730(1.89/2.73*)	AT3G15380(6.70/6.78*)
		AT5G14740(3.15/3.16*)	AT2G07727(0.60/0.40*)	AT5G56540(1.95/2.19*)	AT3G48320(0.68/0.45*)	AT2G07722(2.54/2.76*)	AT5G10180(1.13/0.44*)
		AT1G16400(0.67/0.25*)	AT4G27730(0.44/0.36*)	AT3G18480(3.20/3.25*)	AT4G25090(2.11/3.81*)	AT1G68910(2.36/2.11*)	AT5G46150(2.59/3.18*)
		AT4G11840(1.09/0.82*)	AT3G26330(0.84/0.22*)	AT2G20840(3.11/2.93*)	AT3G48850(1.12/1.27*)	AT5G43370(1.55/2.53*)	AT1G24490(2.24/2.45*)
		AT5G25130(0.37/0.41*)	AT4G30650(0.82/0.32*)	AT5G61350(0.26/0.43*)	AT3G30180(3.42/2.29*)	AT3G45970(1.00/0.40*)	AT2G37640(1.11/2.12*)
		AT1G49960(0.57/0.33*)	AT5G11150(1.73/2.17*)	AT5G05260(1.03/0.44*)	AT4G23290(0.12/0.27*)	AT3G47290(6.87/6.07*)	AT5G25120(0.50/0.43*)
		AT5G55630(1.03/1.24*)	AT2G13800(0.88/0.80*)	AT3G28290(126.74/64.20*)	AT4G36220(0.28/0.20*)	AT5G46330(0.84/2.08*)	AT1G68830(0.52/0.44*)
		AT4G27440(1.24/2.26*)	AT1G62040(3.51/2.52*)	AT1G34520(1.28/3.36*)	AT1G16260(7.77/13.20*)	AT1G26700(1.30/2.04*)	AT5G38430(0.82/0.69*)
		AT5G43350(1.83/5.10*)	AT5G54770(0.66/0.29*)	AT3G55500(1.00/0.92*)	AT1G49410(0.51/0.48*)	AT5G01540(0.29/0.41*)	AT1G04310(2.81/3.80*)
		AT2G46330(1.92/2.06*)	AT5G26980(8.29/6.75*)	AT4G11460(1.00/11.11*)	AT5G05290(0.40/2.19*)	AT3G58810(0.80/0.40*)	AT5G06870(0.54/0.29*)
		AT4G23260(0.55/0.41*)	AT3G24300(0.90/3.08*)	AT3G29030(1.36/1.16*)	AT4G12420(2.97/2.94*)	AT5G05690(1.92/2.08*)	AT4G20235(2.22/2.14*)
		AT1G27190(1.80/2.15*)	AT3G26300(50.20/12.69*)	AT1G05200(2.35/2.96*)	AT1G20190(1.44/2.07*)	AT2G30750(2.04/2.02*)	AT2G46450(5.61/8.00*)
		AT2G47800(0.37/0.47*)	AT1G34580(0.31/0.22*)	AT1G51820(0.38/0.29*)	AT2G22470(2.46/2.02*)	AT4G17340(1.17/1.82*)	AT4G28950(0.73/0.47*)
		AT4G19850(0.49/0.35*)	AT5G24380(0.58/0.47*)	AT1G54280(1.69/2.17*)	AT5G41160(0.78/0.45*)	AT3G47220(3.82/2.44*)	AT2G30950(1.82/2.14*)
		AT2G18450(0.44/0.43*)	AT1G66570(0.58/0.38*)	AT5G58150(0.53/0.41*)	AT2G02580(1.56/2.01*)	AT5G44130(0.39/0.27*)	AT4G15470(2.12/2.17*)
		AT5G24960(1.71/2.22*)	AT4G09160(1.64/2.62*)	AT5G55930(0.71/0.24*)	AT1G71890(0.46/0.32*)	AT5G19600(0.23/0.32*)	AT1G07550(3.13/4.36*)
		AT4G01950(1.66/2.11*)	AT3G26320(0.78/0.49*)	AT2G32270(1.32/2.48*)	AT5G17600(1.89/2.16*)	AT4G37270(26.36/27.90*)	AT4G20410(4.66/6.90*)
		AT1G07560(2.67/2.86*)	AT5G23810(0.71/0.32*)	AT3G45780(2.57/3.01*)	AT2G07719(0.34/0.24*)	AT5G46240(2.17/3.43*)	AT4G01540(0.66/0.41*)
		AT2G15370(0.85/0.47*)	AT1G28040(1.00/0.39*)	AT5G59680(2.24/3.61*)	AT1G75630(2.01/2.09*)	AT1G32860(0.26/0.53*)	AT1G71880(0.57/0.38*)
		AT2G23130(0.40/0.25*)	AT3G30842(0.63/0.33*)	AT3G59140(0.96/2.55*)	AT1G27940(0.05/0.07*)	AT1G64780(0.58/0.41*)	AT5G65165(2.13/0.34*)
		AT4G17730(1.67/2.12*)	AT1G05700(1.34/2.39*)	AT2G33670(1.17/2.03*)	AT5G56320(1.08/0.37*)	AT5G19240(0.78/0.79*)	AT1G29920(0.59/0.47*)
		AT4G18290(1.14/2.05*)	AT2G25810(0.67/0.44*)	AT2G28990(1.31/2.54*)	AT3G44300(1.89/2.15*)	AT5G27100(1.07/0.96*)	AT2G27810(1.10/1.23*)
		AT4G28660(0.37/0.47*)	AT2G16850(0.24/0.32*)	AT5G15250(1.63/5.58*)	AT5G05580(0.38/0.27*)	AT1G66340(1.43/2.07*)	AT4G04570(0.60/0.32*)

		AT4G25140(0.68/0.10*)	AT1G09090(1.04/0.64*)	AT5G38970(1.66/2.81*)	AT4G31480(1.83/2.01*)	AT5G11230(1.16/0.32*)	AT2G32400(0.66/0.44*)
		AT5G25090(0.75/0.45*)	AT2G23310(1.49/1.76*)	AT4G27520(0.37/0.45*)	AT2G26240(3.31/3.50*)	AT1G29930(1.12/0.89*)	AT3G27660(0.96/0.11*)
		AT4G33020(1.07/2.29*)	AT3G01550(0.34/0.26*)	AT1G73190(1.03/0.30*)	AT2G15090(1.00/1.83*)	AT3G17690(0.78/0.40*)	AT4G15233(0.41/0.34*)
		AT4G06598(0.42/0.42*)	AT1G71990(0.50/0.48*)	AT4G15236(0.37/0.39*)	AT3G30875(1.00/0.34*)	AT4G23220(6.85/5.09*)	AT5G10380(0.91/0.37*)
		AT3G48270(0.92/0.27*)	AT3G07040(0.50/0.48*)	AT4G15630(0.61/0.42*)	AT1G35710(2.14/2.07*)	AT5G47070(1.77/2.70*)	AT3G51895(1.76/0.44*)
		AT3G08940(1.53/1.79*)	AT1G06520(0.38/0.38*)	AT1G71960(1.88/2.91*)	AT4G13770(0.05/0.06*)	AT3G20460(0.74/0.47*)	AT1G25530(0.85/0.50*)
		AT1G64220(0.30/0.31*)	AT2G13790(27.39/16.44*)	AT5G45940(1.47/2.46*)	AT2G07698(0.65/0.30*)	AT1G20020(0.59/0.45*)	AT4G23210(0.35/0.22*)
		AT2G07695(0.46/0.48*)	AT2G26450(0.36/0.24*)	AT5G26250(1.00/0.32*)	AT4G15620(0.36/0.27*)	AT1G01580(0.82/0.21*)	AT3G47200(2.87/5.24*)
		AT1G08230(1.81/3.08*)	AT4G27540(1.87/2.07*)	AT3G08930(1.97/2.20*)	AT3G27170(0.41/0.44*)	AT3G54830(0.76/0.30*)	AT4G40090(0.75/0.47*)
		AT5G40420(1.19/0.23*)	AT1G77110(0.68/3.94*)	AT3G45290(1.26/2.28*)	AT3G28740(2.62/11.74*)	AT3G52720(0.58/1.77*)	AT2G47930(1.32/2.12*)
		AT3G10780(0.26/0.24*)	AT4G15320(1.00/0.28*)	AT2G34770(2.47/1.86*)	AT3G26230(1.31/2.31*)	AT1G15460(0.52/0.49*)	AT2G36590(0.13/0.11*)
		AT4G13420(0.20/0.49*)	AT2G07680(0.50/0.44*)	AT4G04620(2.28/2.50*)	AT3G12120(1.81/2.25*)	AT3G21250(1.85/2.25*)	AT5G38820(1.10/0.37*)
		AT5G66380(3.63/4.85*)	AT2G22330(0.42/0.34*)	AT5G65390(1.49/2.08*)	AT5G40780(1.90/3.57*)	AT4G39950(0.80/0.34*)	AT3G60160(0.63/0.44*)
		AT1G18690(1.46/1.30*)	AT3G28270(0.19/0.15*)	AT2G37580(0.63/0.31*)	AT1G61300(0.33/0.36*)	AT3G13090(0.40/0.23*)	AT5G43760(1.75/1.35*)
		AT5G62890(0.44/0.46*)	AT4G04750(0.12/0.39*)	AT5G41300(8.09/16.73*)	AT4G10150(3.14/3.13*)	AT5G07390(1.00/1.00*)	AT1G51890(1.22/3.57*)
		AT4G39030(2.16/0.61*)	AT1G65240(0.17/0.17*)	AT5G18840(0.76/0.24*)	AT3G19270(1.55/2.57*)	AT1G16410(0.52/0.25*)	AT1G63260(6.67/14.69*)
		AT5G55360(0.02/0.04*)	AT3G55940(0.56/2.37*)	AT3G26520(0.83/0.47*)	AT1G08560(0.87/0.60*)	AT5G13580(0.63/0.22*)	AT5G40382(0.69/0.37*)
		AT4G04740(1.14/1.10*)	AT5G24140(0.66/0.40*)	AT5G36870(0.75/0.71*)	AT3G26280(1.23/2.06*)	AT4G29180(0.52/0.48*)	AT1G29910(0.64/0.40*)
		AT3G24290(0.92/2.22*)	AT3G51740(0.73/0.36*)	AT4G13310(1.42/0.33*)	AT3G13065(0.48/0.40*)	AT3G26220(2.15/2.57*)	AT5G09220(2.19/8.05*)
		AT3G01570(9.59/0.22*)	AT4G19690(0.74/0.06*)	AT1G19450(0.27/0.28*)	AT4G21480(3.20/4.39*)	AT2G39530(1.76/3.17*)	AT4G13410(0.86/0.43*)
		AT4G26466(1.13/0.23*)	AT5G01240(1.41/1.84*)	AT1G52190(1.41/2.38*)	AT2G48020(0.69/0.49*)	AT3G05155(5.64/2.33*)	AT4G10360(0.89/0.68*)
		AT4G19680(0.51/0.18*)	AT2G26360(0.68/0.47*)	AT2G47160(1.01/0.70*)			
chromoprotein	8	AT2G38390(0.56/0.19*)	AT4G13310(1.42/0.33*)	AT3G49110(0.60/2.51*)	AT2G07727(0.60/0.40*)	AT4G13770(0.05/0.06*)	AT3G48320(0.68/0.45*)
		AT3G48270(0.92/0.27*)	AT5G05690(1.92/2.08*)				
aromatic_amino_acid_biosynthesis	7	AT5G22630(0.94/0.45*)	AT1G08250(0.38/0.33*)	AT3G44720(0.71/0.47*)	AT4G39980(0.75/0.41*)	AT4G27070(0.34/0.23*)	AT5G17990(1.48/0.57*)
		AT5G54810(0.69/0.49*)					
FAD	17	AT5G24150(0.37/0.31*)	AT1G75450(1.18/2.90*)	AT2G18450(0.44/0.43*)	AT1G63940(0.69/0.61*)	AT5G24160(0.22/0.23*)	AT5G07390(1.00/1.00*)
		AT1G62540(0.25/0.04*)	AT2G24580(0.59/0.42*)	AT1G19250(8.13/2.01*)	AT3G30775(0.46/0.17*)	AT1G09090(1.04/0.64*)	AT1G20020(0.59/0.45*)
		AT3G45300(6.18/3.19*)	AT5G24140(0.66/0.40*)	AT4G25090(2.11/3.81*)	AT5G54770(0.66/0.29*)	AT1G65860(0.48/0.17*)	
Lectin	14	AT1G53080(0.60/0.29*)	AT1G15530(0.46/0.42*)	AT3G45410(2.52/2.49*)	AT1G53060(0.44/0.45*)	AT2G39330(1.08/0.66*)	AT3G16470(0.80/0.54*)
		AT5G28520(2.62/2.17*)	AT1G52040(0.27/0.29*)	AT3G16410(35.08/44.01*)	AT1G52030(0.25/0.21*)	AT3G16400(0.53/0.66*)	AT2G43690(1.73/2.32*)
		AT3G16390(0.31/0.31*)	AT5G01540(0.29/0.41*)				
stress-induced_protein	4	AT4G10250(2.81/3.89*)	AT5G52300(1.39/0.38*)	AT4G25200(0.90/2.83*)	AT3G12580(1.18/2.02*)		
carboxylic_ester_hydrolase	4	AT1G53830(0.99/0.48*)	AT4G02330(2.46/2.03*)	AT2G47550(0.67/0.31*)	AT2G26450(0.36/0.24*)		
Flavoprotein	19	AT5G24150(0.37/0.31*)	AT1G75450(1.18/2.90*)	AT2G18450(0.44/0.43*)	AT1G63940(0.69/0.61*)	AT5G24160(0.22/0.23*)	AT1G48605(0.27/0.45*)
		AT1G62540(0.25/0.04*)	AT1G19250(8.13/2.01*)	AT2G24580(0.59/0.42*)	AT1G18020(1.65/2.40*)	AT1G17990(1.65/2.62*)	AT3G45780(2.57/3.01*)
		AT3G30775(0.46/0.17*)	AT1G20020(0.59/0.45*)	AT3G45300(6.18/3.19*)	AT5G51930(0.31/0.39*)	AT5G24140(0.66/0.40*)	AT1G76680(1.92/3.18*)
		AT1G65860(0.48/0.17*)					

transcription_factor	8	AT5G43270(2.16/2.73*)	AT4G25470(1.41/2.43*)	AT1G53160(0.98/4.36*)	AT1G18570(3.30/11.02*)	AT2G33810(1.88/2.20*)	AT2G47460(0.33/0.23*)
		AT5G05410(1.46/1.76*)	AT4G25560(1.52/2.45*)				
acetylation	12	AT1G29910(0.64/0.40*)	AT1G29920(0.59/0.47*)	AT1G29930(1.12/0.89*)	AT3G27660(0.96/0.11*)	AT4G25140(0.68/0.10*)	AT4G40040(1.92/2.63*)
		AT3G46320(0.28/0.26*)	AT3G01570(9.59/0.22*)	AT3G09480(0.39/0.42*)	AT3G08940(1.53/1.79*)	AT5G10400(2.77/2.60*)	AT5G40420(1.19/0.23*)
metal-binding	233	AT2G42360(0.57/0.54*)	AT3G47470(1.16/1.08*)	AT3G02870(0.66/0.57*)	AT2G14255(2.65/3.32*)	AT4G16845(1.14/1.11*)	AT5G63980(0.53/0.45*)
		AT5G40880(0.40/0.43*)	AT1G43770(1.02/1.16*)	AT1G49570(1.42/0.28*)	AT3G22740(1.58/0.28*)	AT4G04910(0.90/1.07*)	AT1G72680(2.16/2.10*)
		AT3G10815(0.21/0.33*)	AT2G28510(0.61/0.39*)	AT5G42580(0.97/0.35*)	AT1G32780(0.60/0.45*)	AT5G57520(0.76/0.48*)	AT2G07727(0.60/0.40*)
		AT3G48320(0.68/0.45*)	AT3G55770(2.72/2.45*)	AT1G16400(0.67/0.25*)	AT1G19630(1.98/3.61*)	AT3G26330(0.84/0.22*)	AT5G08640(0.14/0.11*)
		AT5G25130(0.37/0.41*)	AT3G30180(3.42/2.29*)	AT1G34510(0.94/0.48*)	AT3G45140(0.06/0.11*)	AT4G26220(0.88/0.46*)	AT5G05260(1.03/0.44*)
		AT3G20395(2.90/7.44*)	AT5G24070(0.81/0.43*)	AT1G14540(1.29/0.44*)	AT5G20620(0.73/0.68*)	AT2G18130(0.81/0.48*)	AT5G25120(0.50/0.43*)
		AT4G36220(0.28/0.20*)	AT1G01210(1.97/2.17*)	AT1G70170(2.08/2.50*)	AT1G30040(0.87/0.94*)	AT1G04310(2.81/3.80*)	AT2G23240(21.70/0.16*)
		AT5G14200(0.56/0.41*)	AT5G38930(1.09/0.42*)	AT3G58810(0.80/0.40*)	AT5G06730(0.57/0.34*)	AT3G10520(0.35/0.42*)	AT4G18350(0.58/0.47*)
		AT1G64950(102.53/150.78*)	AT4G12420(2.97/2.94*)	AT2G46650(0.26/0.21*)	AT5G05690(1.92/2.08*)	AT4G20235(2.22/2.14*)	AT1G78490(1.20/0.29*)
		AT1G75540(2.15/2.42*)	AT2G46950(0.34/0.26*)	AT5G44770(2.59/2.09*)	AT3G26300(50.20/12.69*)	AT5G37890(2.14/2.70*)	AT3G01900(0.58/0.24*)
		AT2G30750(2.04/2.02*)	AT5G38940(0.94/0.44*)	AT2G29130(2.65/2.01*)	AT1G18140(0.61/0.14*)	AT3G10920(0.97/0.92*)	AT2G25160(0.88/0.39*)
		AT1G47380(2.56/2.34*)	AT1G47570(23.54/68.20*)	AT3G30460(0.24/0.25*)	AT1G65040(1.84/2.27*)	AT5G36240(0.08/0.04*)	AT5G63180(1.20/2.66*)
		AT1G54280(1.69/2.17*)	AT5G43270(2.16/2.73*)	AT1G14550(1.12/0.38*)	AT2G30950(1.82/2.14*)	AT4G22690(1.06/1.16*)	AT2G34920(0.90/0.48*)
		AT2G02580(1.56/2.01*)	AT3G52780(4.27/2.64*)	AT5G24960(1.71/2.22*)	AT4G08770(1.68/0.39*)	AT3G60080(2.60/2.51*)	AT4G33870(1.02/2.97*)
		AT3G26320(0.78/0.49*)	AT5G17600(1.89/2.16*)	AT4G37270(26.36/27.90*)	AT2G12190(8.79/9.34*)	AT2G27010(0.37/0.21*)	AT1G62310(1.59/3.12*)
		AT3G20940(1.37/0.40*)	AT3G20950(0.63/0.24*)	AT3G26690(2.39/3.34*)	AT2G16090(1.60/2.06*)	AT2G14100(63.98/5.82*)	AT1G28040(1.00/0.39*)
		AT4G19170(2.15/3.66*)	AT2G34000(2.25/8.44*)	AT3G18500(32.47/71.98*)	AT2G24800(0.60/3.36*)	AT1G72610(0.38/0.25*)	AT2G21910(1.31/2.74*)
		AT4G07950(41.25/28.67*)	AT4G08780(3.25/0.23*)	AT5G25160(1.60/2.57*)	AT1G17890(1.45/1.41*)	AT2G38080(1.41/2.19*)	AT2G27690(0.39/0.42*)
		AT1G28960(1.05/1.38*)	AT5G65165(2.13/0.34*)	AT2G19610(0.37/0.27*)	AT1G29800(82.83/61.93*)	AT5G58910(0.74/0.34*)	AT2G27000(0.92/0.50*)
		AT2G33810(1.88/2.20*)	AT4G12330(55.28/68.16*)	AT5G25560(1.52/2.73*)	AT4G22880(1.00/0.23*)	AT1G29920(0.59/0.47*)	AT1G49630(2.10/2.92*)
		AT5G19890(0.96/0.26*)	AT5G64000(0.78/0.23*)	AT1G16700(0.62/0.40*)	AT3G47120(2.13/2.44*)	AT1G66140(0.83/0.47*)	AT5G15250(1.63/5.58*)
		AT5G02760(1.14/2.39*)	AT5G22500(4.94/3.47*)	AT4G37310(0.13/0.16*)	AT1G66340(1.43/2.07*)	AT1G61140(1.34/2.31*)	AT5G09650(0.53/0.49*)
		AT1G77120(1.20/3.06*)	AT5G14130(0.91/0.37*)	AT5G57660(1.97/2.15*)	AT5G38970(1.66/2.81*)	AT5G39160(16.59/25.74*)	AT2G36490(1.58/1.73*)
		AT5G19880(1.26/0.42*)	AT1G29930(1.12/0.89*)	AT5G42250(1.36/1.92*)	AT3G61040(1.00/0.16*)	AT4G15440(1.00/0.78*)	AT4G15300(6.06/3.74*)
		AT4G08870(0.50/0.36*)	AT5G13430(0.63/0.50*)	AT2G42060(1.64/3.96*)	AT5G10380(0.91/0.37*)	AT4G37400(1.14/0.32*)	AT3G48270(0.92/0.27*)
		AT4G05320(2.25/1.94*)	AT3G05950(2.20/0.01*)	AT5G39190(68.20/51.10*)	AT3G08940(1.53/1.79*)	AT2G26580(1.05/3.67*)	AT1G01190(1.26/3.72*)
		AT4G13770(0.05/0.06*)	AT1G29560(1.00/0.86*)	AT5G55970(1.35/1.74*)	AT4G32170(1.00/0.46*)	AT4G15248(0.13/0.21*)	AT1G79470(0.43/0.47*)
		AT3G49110(0.60/2.51*)	AT2G44410(1.66/1.49*)	AT5G15790(0.27/0.22*)	AT5G45940(1.47/2.46*)	AT3G18520(2.12/2.63*)	AT4G15330(0.89/0.29*)
		AT5G06720(1.83/0.13*)	AT3G01190(1.42/3.23*)	AT1G17420(0.94/1.15*)	AT1G18980(0.51/0.49*)	AT2G18980(1.38/2.54*)	AT5G04330(1.92/2.24*)
		AT5G67400(0.47/0.48*)	AT5G54080(4.55/2.45*)	AT2G38390(0.56/0.19*)	AT3G28740(2.62/11.74*)	AT1G64940(5.46/5.50*)	AT3G52720(0.58/1.77*)
		AT1G63900(1.95/2.02*)	AT5G42650(0.50/0.33*)	AT2G21890(2.23/2.42*)	AT2G34770(2.47/1.86*)	AT3G26230(1.31/2.31*)	AT5G64100(2.27/5.21*)
		AT5G59220(1.36/2.48*)	AT5G05340(0.20/0.06*)	AT1G66610(0.17/0.22*)	AT5G01600(2.40/2.72*)	AT5G20630(1.47/4.16*)	AT2G22330(0.42/0.34*)
		AT4G32810(4.71/3.64*)	AT2G01880(0.89/0.11*)	AT2G28450(2.22/2.55*)	AT4G39950(0.80/0.34*)	AT5G52320(0.56/0.28*)	AT2G35635(11.46/11.45*)
		AT1G49130(1.72/1.75*)	AT2G41480(1.02/0.12*)	AT4G37970(0.73/0.32*)	AT2G37580(0.63/0.31*)	AT5G39130(9.24/11.30*)	AT1G14700(0.61/0.50*)

		AT3G44750(0.51/0.53*)	AT3G60530(1.96/3.67*)	AT2G28860(2.99/0.59*)	AT2G18670(1.03/1.17*)	AT5G17790(1.55/2.17*)	AT4G10150(3.14/3.13*)
		AT1G11100(1.93/2.30*)	AT1G53160(0.98/4.36*)	AT4G22110(1.11/0.43*)	AT2G44990(1.00/0.23*)	AT3G19270(1.55/2.57*)	AT3G54780(0.45/0.36*)
		AT5G21100(1.51/3.00*)	AT1G16410(0.52/0.25*)	AT1G36950(0.55/0.41*)	AT4G37330(1.83/2.22*)	AT3G44970(1.42/2.67*)	AT3G26280(1.23/2.06*)
		AT1G29910(0.64/0.40*)	AT4G13310(1.42/0.33*)	AT1G79000(3.20/3.85*)	AT2G19800(0.69/0.44*)	AT3G26220(2.15/2.57*)	AT3G51240(0.42/0.23*)
		AT3G26125(1.86/3.75*)	AT2G19900(0.84/0.40*)	AT3G52820(0.63/0.45*)	AT4G24890(0.74/1.18*)	AT4G12280(0.26/0.14*)	
transmembrane_protein	11	AT5G26250(1.00/0.32*)	AT1G29910(0.64/0.40*)	AT1G29920(0.59/0.47*)	AT4G18290(1.14/2.05*)	AT1G29930(1.12/0.89*)	AT5G09220(2.19/8.05*)
		AT1G75630(2.01/2.09*)	AT5G46240(2.17/3.43*)	AT2G07727(0.60/0.40*)	AT1G73190(1.03/0.30*)	AT4G21480(3.20/4.39*)	
cell_membrane	55	AT5G19240(0.78/0.79*)	AT3G44300(1.89/2.15*)	AT4G13420(0.20/0.49*)	AT3G24300(0.90/3.08*)	AT2G27810(1.10/1.23*)	AT4G12420(2.97/2.94*)
		AT2G16850(0.24/0.32*)	AT5G25090(0.75/0.45*)	AT4G27520(0.37/0.45*)	AT5G65390(1.49/2.08*)	AT2G46450(5.61/8.00*)	AT4G33020(1.07/2.29*)
		AT5G40730(1.89/2.73*)	AT5G40780(1.90/3.57*)	AT2G47800(0.37/0.47*)	AT2G22470(2.46/2.02*)	AT5G56540(1.95/2.19*)	AT5G24380(0.58/0.47*)
		AT3G17690(0.78/0.40*)	AT3G47220(3.82/2.44*)	AT1G61300(0.33/0.36*)	AT2G20840(3.11/2.93*)	AT5G41300(8.09/16.73*)	AT1G66570(0.58/0.38*)
		AT5G58150(0.53/0.41*)	AT1G51890(1.22/3.57*)	AT5G47070(1.77/2.70*)	AT5G44130(0.39/0.27*)	AT1G65240(0.17/0.17*)	AT1G71890(0.46/0.32*)
		AT1G25530(0.85/0.50*)	AT2G32270(1.32/2.48*)	AT2G13790(27.39/16.44*)	AT3G55940(0.56/2.37*)	AT3G45780(2.57/3.01*)	AT5G23810(0.71/0.32*)
		AT5G36870(0.75/0.71*)	AT3G51740(0.73/0.36*)	AT3G47290(6.87/6.07*)	AT2G13800(0.88/0.80*)	AT3G47200(2.87/5.24*)	AT5G09220(2.19/8.05*)
		AT1G32860(0.26/0.53*)	AT1G71880(0.57/0.38*)	AT4G19690(0.74/0.06*)	AT5G46330(0.84/2.08*)	AT2G23130(0.40/0.25*)	AT4G40090(0.75/0.47*)
		AT4G26466(1.13/0.23*)	AT5G01240(1.41/1.84*)	AT2G47930(1.32/2.12*)	AT4G19680(0.51/0.18*)	AT5G43350(1.83/5.10*)	AT2G46330(1.92/2.06*)
		AT5G01540(0.29/0.41*)					
cell_wall_biogenesis|degradation	38	AT5G05290(0.40/2.19*)	AT1G53830(0.99/0.48*)	AT3G44990(0.98/5.25*)	AT2G37640(1.11/2.12*)	AT1G76930(2.06/3.56*)	AT3G29030(1.36/1.16*)
		AT1G10550(1.01/2.41*)	AT2G47550(0.67/0.31*)	AT5G57530(0.27/0.23*)	AT3G06830(1.66/0.50*)	AT4G18990(1.22/0.19*)	AT4G23560(0.78/2.68*)
		AT2G43860(0.29/0.33*)	AT2G19150(2.65/2.30*)	AT1G64390(1.47/1.15*)	AT4G02320(0.64/0.44*)	AT5G36870(0.75/0.71*)	AT2G15370(0.85/0.47*)
		AT1G20190(1.44/2.07*)	AT2G18800(0.44/0.29*)	AT4G30280(2.35/2.12*)	AT4G28850(0.96/0.94*)	AT5G57560(1.02/0.35*)	AT1G32860(0.26/0.53*)
		AT1G60590(0.22/0.17*)	AT1G11580(0.71/0.33*)	AT4G02290(1.20/1.39*)	AT4G13410(0.86/0.43*)	AT4G25810(1.15/0.43*)	AT1G71990(0.50/0.48*)
		AT1G65310(2.77/2.75*)	AT4G15320(1.00/0.28*)	AT1G11370(0.05/0.05*)	AT3G55500(1.00/0.92*)	AT4G02330(2.46/2.03*)	AT2G26440(0.30/0.34*)
		AT1G10640(0.39/0.28*)	AT5G56320(1.08/0.37*)				
lithium	3	AT3G02870(0.66/0.57*)	AT5G63980(0.53/0.45*)	AT5G64000(0.78/0.23*)			
lipid_droplet	4	AT3G27660(0.96/0.11*)	AT4G25140(0.68/0.10*)	AT3G01570(9.59/0.22*)	AT5G40420(1.19/0.23*)		
transmembrane	229	AT5G24150(0.37/0.31*)	AT3G47470(1.16/1.08*)	AT2G42360(0.57/0.54*)	AT1G80830(2.15/2.43*)	AT2G14255(2.65/3.32*)	AT1G80760(21.49/15.26*)
		AT4G23160(1.00/1.00*)	AT1G16390(11.04/13.24*)	AT3G11430(0.63/0.05*)	AT4G23700(0.66/2.58*)	AT1G64170(0.41/0.45*)	AT5G24160(0.22/0.23*)
		AT2G39920(0.95/2.25*)	AT3G15380(6.70/6.78*)	AT2G07727(0.60/0.40*)	AT3G48320(0.68/0.45*)	AT2G07722(2.54/2.76*)	AT5G10180(1.13/0.44*)
		AT1G16400(0.67/0.25*)	AT4G27730(0.44/0.36*)	AT3G18480(3.20/3.25*)	AT1G68910(2.36/2.11*)	AT5G46150(2.59/3.18*)	AT4G25090(2.11/3.81*)
		AT3G26330(0.84/0.22*)	AT2G20840(3.11/2.93*)	AT5G43370(1.55/2.53*)	AT1G24490(2.24/2.45*)	AT3G48850(1.12/1.27*)	AT5G25130(0.37/0.41*)
		AT4G30650(0.82/0.32*)	AT5G61350(0.26/0.43*)	AT3G30180(3.42/2.29*)	AT3G45970(1.00/0.40*)	AT1G49960(0.57/0.33*)	AT5G11150(1.73/2.17*)
		AT5G05260(1.03/0.44*)	AT4G23290(0.12/0.27*)	AT5G25120(0.50/0.43*)	AT5G55630(1.03/1.24*)	AT2G13800(0.88/0.80*)	AT3G28290(126.74/64.20*)
		AT4G36220(0.28/0.20*)	AT5G46330(0.84/2.08*)	AT1G34520(1.28/3.36*)	AT1G16260(7.77/13.20*)	AT1G26700(1.30/2.04*)	AT5G43350(1.83/5.10*)
		AT1G49410(0.51/0.48*)	AT5G01540(0.29/0.41*)	AT1G04310(2.81/3.80*)	AT5G26980(8.29/6.75*)	AT4G11460(1.00/11.11*)	AT3G58810(0.80/0.40*)
		AT4G23260(0.55/0.41*)	AT3G24300(0.90/3.08*)	AT5G05690(1.92/2.08*)	AT4G20235(2.22/2.14*)	AT1G27190(1.80/2.15*)	AT3G26300(50.20/12.69*)
		AT1G05200(2.35/2.96*)	AT2G30750(2.04/2.02*)	AT2G46450(5.61/8.00*)	AT2G47800(0.37/0.47*)	AT1G34580(0.31/0.22*)	AT1G51820(0.38/0.29*)
		AT4G17340(1.17/1.82*)	AT4G19850(0.49/0.35*)	AT5G24380(0.58/0.47*)	AT1G54280(1.69/2.17*)	AT5G41160(0.78/0.45*)	AT2G30950(1.82/2.14*)

		AT1G66570(0.58/0.38*)	AT5G58150(0.53/0.41*)	AT2G02580(1.56/2.01*)	AT4G15470(2.12/2.17*)	AT5G24960(1.71/2.22*)	AT5G55930(0.71/0.24*)
		AT1G71890(0.46/0.32*)	AT5G19600(0.23/0.32*)	AT1G07550(3.13/4.36*)	AT4G01950(1.66/2.11*)	AT3G26320(0.78/0.49*)	AT2G32270(1.32/2.48*)
		AT5G17600(1.89/2.16*)	AT4G37270(26.36/27.90*)	AT1G07560(2.67/2.86*)	AT5G23810(0.71/0.32*)	AT2G07719(0.34/0.24*)	AT5G46240(2.17/3.43*)
		AT4G01540(0.66/0.41*)	AT2G15370(0.85/0.47*)	AT1G28040(1.00/0.39*)	AT5G59680(2.24/3.61*)	AT1G75630(2.01/2.09*)	AT1G71880(0.57/0.38*)
		AT3G30842(0.63/0.33*)	AT3G59140(0.96/2.55*)	AT1G27940(0.05/0.07*)	AT1G64780(0.58/0.41*)	AT4G17730(1.67/2.12*)	AT1G05700(1.34/2.39*)
		AT2G33670(1.17/2.03*)	AT1G29920(0.59/0.47*)	AT4G18290(1.14/2.05*)	AT2G25810(0.67/0.44*)	AT2G28990(1.31/2.54*)	AT5G27100(1.07/0.96*)
		AT2G27810(1.10/1.23*)	AT2G16850(0.24/0.32*)	AT5G15250(1.63/5.58*)	AT1G66340(1.43/2.07*)	AT4G25140(0.68/0.10*)	AT4G04570(0.60/0.32*)
		AT1G09090(1.04/0.64*)	AT5G38970(1.66/2.81*)	AT5G11230(1.16/0.32*)	AT4G04850(0.27/0.36*)	AT2G32400(0.66/0.44*)	AT2G23310(1.49/1.76*)
		AT1G29930(1.12/0.89*)	AT2G26240(3.31/3.50*)	AT3G27660(0.96/0.11*)	AT4G33020(1.07/2.29*)	AT3G01550(0.34/0.26*)	AT1G73190(1.03/0.30*)
		AT2G15090(1.00/1.83*)	AT3G17690(0.78/0.40*)	AT4G06598(0.42/0.42*)	AT4G15233(0.41/0.34*)	AT1G71990(0.50/0.48*)	AT4G15236(0.37/0.39*)
		AT3G30875(1.00/0.34*)	AT4G23220(6.85/5.09*)	AT5G10380(0.91/0.37*)	AT3G48270(0.92/0.27*)	AT4G15630(0.61/0.42*)	AT1G35710(2.14/2.07*)
		AT3G51895(1.76/0.44*)	AT3G08940(1.53/1.79*)	AT1G06520(0.38/0.38*)	AT1G71960(1.88/2.91*)	AT4G13770(0.05/0.06*)	AT3G20460(0.74/0.47*)
		AT1G25530(0.85/0.50*)	AT1G64220(0.30/0.31*)	AT2G13790(27.39/16.44*)	AT5G45940(1.47/2.46*)	AT2G07698(0.65/0.30*)	AT4G23210(0.35/0.22*)
		AT2G07695(0.46/0.48*)	AT2G26450(0.36/0.24*)	AT5G26250(1.00/0.32*)	AT4G15620(0.36/0.27*)	AT1G01580(0.82/0.21*)	AT3G47200(2.87/5.24*)
		AT3G45680(0.19/0.09*)	AT1G08230(1.81/3.08*)	AT4G27540(1.87/2.07*)	AT3G08930(1.97/2.20*)	AT3G27170(0.41/0.44*)	AT3G54830(0.76/0.30*)
		AT5G40420(1.19/0.23*)	AT1G77110(0.68/3.94*)	AT3G45290(1.26/2.28*)	AT3G28740(2.62/11.74*)	AT3G52720(0.58/1.77*)	AT3G10780(0.26/0.24*)
		AT4G15320(1.00/0.28*)	AT2G34770(2.47/1.86*)	AT3G26230(1.31/2.31*)	AT1G15460(0.52/0.49*)	AT2G36590(0.13/0.11*)	AT4G13420(0.20/0.49*)
		AT2G07680(0.50/0.44*)	AT3G12120(1.81/2.25*)	AT3G21250(1.85/2.25*)	AT5G38820(1.10/0.37*)	AT5G66380(3.63/4.85*)	AT1G31820(14.55/47.32*)
		AT2G22330(0.42/0.34*)	AT5G40780(1.90/3.57*)	AT4G39950(0.80/0.34*)	AT1G18690(1.46/1.30*)	AT3G60160(0.63/0.44*)	AT3G28270(0.19/0.15*)
		AT2G37580(0.63/0.31*)	AT3G13090(0.40/0.23*)	AT5G43760(1.75/1.35*)	AT5G62890(0.44/0.46*)	AT4G04750(0.12/0.39*)	AT4G10150(3.14/3.13*)
		AT5G07390(1.00/1.00*)	AT1G51890(1.22/3.57*)	AT4G39030(2.16/0.61*)	AT5G18840(0.76/0.24*)	AT3G19270(1.55/2.57*)	AT1G16410(0.52/0.25*)
		AT1G63260(6.67/14.69*)	AT5G55360(0.02/0.04*)	AT3G26520(0.83/0.47*)	AT1G08560(0.87/0.60*)	AT5G13580(0.63/0.22*)	AT5G40382(0.69/0.37*)
		AT5G24140(0.66/0.40*)	AT5G36870(0.75/0.71*)	AT3G26280(1.23/2.06*)	AT4G29180(0.52/0.48*)	AT1G29910(0.64/0.40*)	AT3G51740(0.73/0.36*)
		AT3G24290(0.92/2.22*)	AT4G13310(1.42/0.33*)	AT3G13065(0.48/0.40*)	AT3G26220(2.15/2.57*)	AT5G09220(2.19/8.05*)	AT3G01570(9.59/0.22*)
		AT4G19690(0.74/0.06*)	AT1G19450(0.27/0.28*)	AT4G21480(3.20/4.39*)	AT2G39530(1.76/3.17*)	AT4G13410(0.86/0.43*)	AT5G01240(1.41/1.84*)
		AT1G52190(1.41/2.38*)	AT2G48020(0.69/0.49*)	AT3G05155(5.64/2.33*)	AT4G10360(0.89/0.68*)	AT4G19680(0.51/0.18*)	AT2G26360(0.68/0.47*)
		AT2G47160(1.01/0.70*)					
sugar_transport	12	AT5G26250(1.00/0.32*)	AT2G48020(0.69/0.49*)	AT1G66570(0.58/0.38*)	AT3G05155(5.64/2.33*)	AT1G71880(0.57/0.38*)	AT1G34580(0.31/0.22*)
		AT5G18840(0.76/0.24*)	AT1G19450(0.27/0.28*)	AT1G71890(0.46/0.32*)	AT4G21480(3.20/4.39*)	AT4G04750(0.12/0.39*)	AT3G20460(0.74/0.47*)
gpi-anchor	17	AT5G19240(0.78/0.79*)	AT5G25090(0.75/0.45*)	AT4G27520(0.37/0.45*)	AT5G65390(1.49/2.08*)	AT5G44130(0.39/0.27*)	AT1G32860(0.26/0.53*)
		AT5G40730(1.89/2.73*)	AT1G65240(0.17/0.17*)	AT2G22470(2.46/2.02*)	AT5G56540(1.95/2.19*)	AT2G23130(0.40/0.25*)	AT4G12420(2.97/2.94*)
		AT4G40090(0.75/0.47*)	AT4G26466(1.13/0.23*)	AT2G47930(1.32/2.12*)	AT2G46330(1.92/2.06*)	AT5G41300(8.09/16.73*)	
calcium	44	AT5G64100(2.27/5.21*)	AT5G19890(0.96/0.26*)	AT5G07390(1.00/1.00*)	AT4G38810(2.47/2.13*)	AT5G05340(0.20/0.06*)	AT5G06730(0.57/0.34*)
		AT5G64000(0.78/0.23*)	AT3G59820(0.48/0.40*)	AT4G08770(1.68/0.39*)	AT1G76640(0.15/0.37*)	AT1G34510(0.94/0.48*)	AT4G33870(1.02/2.97*)
		AT5G23580(1.08/0.28*)	AT5G63980(0.53/0.45*)	AT3G49110(0.60/2.51*)	AT5G24070(0.81/0.43*)	AT5G14130(0.91/0.37*)	AT1G14540(1.29/0.44*)
		AT1G49570(1.42/0.28*)	AT4G04740(1.14/1.10*)	AT5G06720(1.83/0.13*)	AT3G01190(1.42/3.23*)	AT1G64480(0.34/0.35*)	AT3G10190(1.96/1.90*)
		AT5G19880(1.26/0.42*)	AT5G55630(1.03/1.24*)	AT5G55400(0.58/0.50*)	AT1G29020(0.73/5.28*)	AT1G05990(0.48/0.48*)	AT2G34020(8.97/6.41*)
		AT2G18980(1.38/2.54*)	AT4G27280(2.15/2.62*)	AT2G24800(0.60/3.36*)	AT5G67400(0.47/0.48*)	AT4G08780(3.25/0.23*)	AT3G50770(0.94/0.16*)

		AT2G38390(0.56/0.19*)	AT2G41480(1.02/0.12*)	AT5G63180(1.20/2.66*)	AT3G52720(0.58/1.77*)	AT3G22930(0.64/0.36*)	AT1G14550(1.12/0.38*)
		AT4G25090(2.11/3.81*)	AT4G11840(1.09/0.82*)				
thiol_protease	13	AT3G48350(0.42/0.38*)	AT4G36880(0.45/3.97*)	AT2G22310(3.65/3.48*)	AT4G16190(2.33/1.87*)	AT4G11310(6.44/5.46*)	AT2G44140(1.11/1.13*)
		AT5G50260(7.95/4.15*)	AT4G11320(6.67/12.10*)	AT3G48380(2.64/2.89*)	AT1G79330(0.19/0.14*)	AT3G48340(0.88/0.44*)	AT1G16420(0.35/0.48*)
		AT3G54940(1.52/0.44*)					
tryptophan_biosynthesis	4	AT3G54640(1.10/0.41*)	AT4G27070(0.34/0.23*)	AT1G52410(0.44/0.11*)	AT5G17990(1.48/0.57*)		
copper	10	AT2G38080(1.41/2.19*)	AT2G29130(2.65/2.01*)	AT1G66340(1.43/2.07*)	AT1G18140(0.61/0.14*)	AT5G58910(0.74/0.34*)	AT5G21100(1.51/3.00*)
		AT4G12420(2.97/2.94*)	AT1G04310(2.81/3.80*)	AT1G72680(2.16/2.10*)	AT4G12280(0.26/0.14*)		
Ethylene_signaling_pathway	23	AT2G44840(0.65/0.34*)	AT3G14230(1.11/1.41*)	AT3G61630(2.65/4.19*)	AT1G12630(0.32/0.20*)	AT5G44210(1.35/2.58*)	AT1G03800(1.11/2.02*)
		AT4G32800(0.88/0.49*)	AT5G18560(0.77/0.43*)	AT5G65100(1.05/2.21*)	AT2G25820(0.60/0.39*)	AT5G47220(0.15/0.23*)	AT1G68840(1.51/1.94*)
		AT5G64750(1.39/2.41*)	AT1G06160(0.60/2.23*)	AT1G66340(1.43/2.07*)	AT1G12890(0.76/0.36*)	AT1G28370(0.48/0.47*)	AT2G23340(1.06/1.29*)
		AT4G13620(0.96/0.50*)	AT5G58550(0.66/0.61*)	AT1G04310(2.81/3.80*)	AT4G16750(1.29/2.19*)	AT1G46768(1.24/2.41*)	
coiled_coil	60	AT1G61190(0.43/0.47*)	AT3G61390(0.84/3.13*)	AT1G09770(1.92/2.02*)	AT3G47120(2.13/2.44*)	AT5G48620(15.99/14.59*)	AT1G24260(0.24/0.35*)
		AT1G61310(6.16/49.29*)	AT1G59218(61.60/117.99*)	AT5G58550(0.66/0.61*)	AT1G15890(2.21/2.12*)	AT5G47250(0.27/0.22*)	AT1G61180(0.74/0.92*)
		AT1G50180(0.24/0.35*)	AT1G68810(1.54/2.18*)	AT3G28270(0.19/0.15*)	AT3G18480(3.20/3.25*)	AT1G68910(2.36/2.11*)	AT2G20840(3.11/2.93*)
		AT5G45490(40.88/33.10*)	AT1G62630(1.00/13.38*)	AT1G24490(2.24/2.45*)	AT3G46530(30.77/53.76*)	AT4G39030(2.16/0.61*)	AT1G12210(1.25/7.25*)
		AT2G45920(1.96/2.10*)	AT5G65100(1.05/2.21*)	AT1G65800(1.06/0.85*)	AT3G46730(1.11/2.17*)	AT3G24890(5.48/7.96*)	AT1G58400(5.00/3.77*)
		AT5G11150(1.73/2.17*)	AT1G63360(2.36/2.77*)	AT1G08560(0.87/0.60*)	AT2G07698(0.65/0.30*)	AT2G46340(2.02/2.68*)	AT5G45510(1.20/1.28*)
		AT3G50950(0.55/0.41*)	AT5G23570(0.22/0.17*)	AT4G27040(0.90/0.96*)	AT1G58807(92.90/68.68*)	AT1G12220(2.01/3.53*)	AT3G28290(126.74/64.20*)
		AT3G08930(1.97/2.20*)	AT5G41620(2.05/3.03*)	AT1G59780(3.13/3.29*)	AT1G59124(7.61/11.45*)	AT3G44530(1.86/2.48*)	AT1G52150(2.63/2.63*)
		AT1G21810(0.36/0.43*)	AT2G18260(1.97/2.27*)	AT4G17730(1.67/2.12*)	AT1G58602(4.40/11.24*)	AT5G63020(9.47/8.43*)	AT5G35450(2.24/2.37*)
		AT5G43740(8.34/7.08*)	AT1G51480(0.04/0.06*)	AT4G29170(2.94/4.39*)	AT1G58848(6.64/11.54*)	AT5G43470(49.01/45.79*)	AT5G26980(8.29/6.75*)
vitamin_c	3	AT3G51240(0.42/0.23*)	AT5G08640(0.14/0.11*)	AT4G22880(1.00/0.23*)			
toxin	3	AT2G15010(1.40/0.07*)	AT1G72260(0.99/0.55*)	AT1G11580(0.71/0.33*)			
DNA_binding	19	AT1G53160(0.98/4.36*)	AT4G17460(2.50/2.31*)	AT1G66140(0.83/0.47*)	AT5G57520(0.76/0.48*)	AT1G24260(0.24/0.35*)	AT2G44910(0.96/1.78*)
		AT5G25160(1.60/2.57*)	AT1G62990(1.06/0.48*)	AT3G58780(1.38/3.83*)	AT3G61890(3.11/3.32*)	AT4G40040(1.92/2.63*)	AT5G43270(2.16/2.73*)
		AT3G46320(0.28/0.26*)	AT2G33810(1.88/2.20*)	AT4G40060(1.93/2.27*)	AT2G45660(0.52/0.50*)	AT1G22640(1.70/2.48*)	AT5G10400(2.77/2.60*)
		AT2G03710(40.60/37.25*)					
pyridoxal_phosphate	14	AT1G16540(2.12/2.11*)	AT3G19710(0.31/0.05*)	AT5G28237(0.86/0.67*)	AT4G13890(0.94/0.20*)	AT1G62800(0.59/0.33*)	AT4G33010(0.59/0.43*)
		AT5G54810(0.69/0.49*)	AT1G50090(0.92/2.43*)	AT5G51690(81.05/183.70*)	AT4G37770(1.16/2.71*)	AT3G48730(0.39/0.39*)	AT1G10060(1.84/2.96*)
		AT1G10070(3.23/2.09*)	AT4G27070(0.34/0.23*)				
