# Supplementary material for: Far-Red Light-Mediated Seedling Development in Arabidopsis Involves FAR-RED INSENSITIVE 219/JASMONATE RESISTANT 1-Dependent and -Independent Pathways
Source: PLoS One. 2015 Jul 15;10(7):e0132723. doi: 10.1371/journal.pone.0132723 (PMC4503420; doi:10.1371/journal.pone.0132723)
Supplement: S6 Table — (PDF) [file pone.0132723.s014.pdf]

S6 Table. List of hormone-related genes in *fin219*/Col.

Gene Name	Systematic Name	expression ratio (-MeJA/+MeJA)	Description
**ABA-related**			
AT5G35730	AT5G35730.1	(2.13/1.94*)	tair|EXS family protein / ERD1/XPR1/SYG1 family protein [AT5G35730.1]
AT1G32090	AT1G32090.1	(3.10/3.85*)	tair|early-responsive to dehydration protein-related / ERD protein-related [AT1G32090.1]
RAB18	AT5G66400.1	(9.77/0.28*)	tair|RAB18 (RESPONSIVE TO ABA 18) [AT5G66400.1]
AT3G02480	AT3G02480.1	(2.21/0.77*)	tair|ABA-responsive protein-related [AT3G02480.1]
AHG1	AT5G51760.1	(2.16/0.54*)	tair|AHG1 (ABA-HYPERSENSITIVE GERMINATION 1); protein serine/threonine phosphatase [AT5G51760.1]
ABA3	AT1G16540.1	(2.12/2.11*)	tair|ABA3/ATABA3/LOS5/SIR3 (ABA DEFICIENT 3); Mo-molybdopterin cofactor sulfurase/ selenocysteine lyase [AT1G16540.1]
NCED4	AT4G19170.1	(2.15/3.66*)	tair|NCED4 (NINE-CIS-EPOXYCAROTENOID DIOXYGENASE 4) [AT4G19170.1]
ABI4	AT2G40220.1	(4.88/0.68*)	tair|ABI4 (ABA INSENSITIVE 4); DNA binding / transcription factor [AT2G40220.1]
ABF2	AT1G45249.1	(76.99/43.96*)	tair|ABF2 (ABSCISIC ACID RESPONSIVE ELEMENTS-BINDING FACTOR 2) [AT1G45249.1]
DREB1A	AT4G25480.1	(2.15/2.67*)	tair|DREB1A (DEHYDRATION RESPONSE ELEMENT B1A); DNA binding / transcription activator/ transcription factor [AT4G25480.1]
NCED5	AT1G30100.1	(0.48/0.97*)	tair|NCED5 (NINE-CIS-EPOXYCAROTENOID DIOXYGENASE 5) [AT1G30100.1]
AT5G13200	AT5G13200.1	(0.28/0.33*)	tair|GRAM domain-containing protein / ABA-responsive protein-related [AT5G13200.1]
ERD5	AT3G30775.1	(0.46/0.17*)	tair|ERD5 (EARLY RESPONSIVE TO DEHYDRATION 5); proline dehydrogenase [AT3G30775.1]
AT1G68740	AT1G68740.1	(0.44/1.12*)	tair|EXS family protein / ERD1/XPR1/SYG1 family protein [AT1G68740.1]
ABR1	AT5G64750.1	(1.39/2.41*)	tair|ABR1 (ABA REPRESSOR1); DNA binding / transcription factor [AT5G64750.1]
AT1G32090	AT1G32090.1	(3.10/3.85*)	tair|early-responsive to dehydration protein-related / ERD protein-related [AT1G32090.1]
ERD10	AT1G20450.1	(1.39/1.91*)	tair|ERD10/LTI45 (EARLY RESPONSIVE TO DEHYDRATION 10) [AT1G20450.1]
AT1G69480	AT1G69480.1	(1.81/2.98*)	tair|EXS family protein / ERD1/XPR1/SYG1 family protein [AT1G69480.1]
NCED2	AT4G18350.1	(0.58/0.47*)	tair|NCED2 (NINE-CIS-EPOXYCAROTENOID DIOXYGENASE 2) [AT4G18350.1]
**Ethylene-related**			
ERS2	AT1G04310.1	(2.81/3.80*)	tair|ERS2 (ETHYLENE RESPONSE SENSOR 2); receptor [AT1G04310.1]
ACS12	AT5G51690.1	(81.05/183.70*)	tair|ACS12 (1-Amino-cyclopropane-1-carboxylate synthase 12); 1-aminocyclopropane-1-carboxylate synthase [AT5G51690.1]
AT1G12010	AT1G12010.1	(12.46/23.01*)	tair|1-aminocyclopropane-1-carboxylate oxidase, putative / ACC oxidase, putative [AT1G12010.1]
ACS8	AT4G37770.1	(1.16/2.71*)	tair|ACS8 (1-Amino-cyclopropane-1-carboxylate synthase 8) [AT4G37770.1]
AT2G20880	AT2G20880.1	(3.28/1.45*)	tair|AP2 domain-containing transcription factor, putative [AT2G20880.1]
AT2G23340	AT2G23340.1	(1.06/1.29*)	tair|AP2 domain-containing transcription factor, putative [AT2G23340.1]
AT5G18450	AT5G18450.1	(4.57/0.24*)	tair|AP2 domain-containing transcription factor, putative [AT5G18450.1]
ERF2	AT5G47220.1	(0.15/0.23*)	tair|ATERF-2/ATERF2/ERF2 (ETHYLENE RESPONSE FACTOR 2); DNA binding / transcription activator/ transcription factor [AT5G47220.1]
ERF10	AT1G03800.1	(1.11/2.02*)	tair|ATERF10/ERF10 (ERF domain protein 10); DNA binding / transcription factor/ transcription repressor [AT1G03800.1]
ERF9	AT5G44210.1	(1.35/2.58*)	tair|ATERF-9/ATERF9/ERF9 (ERF domain protein 9); DNA binding / transcription factor/ transcription repressor [AT5G44210.1]
ERF11	AT1G28370.1	(0.48/0.47*)	tair|ATERF11/ERF11 (ERF domain protein 11); DNA binding / transcription factor/ transcription repressor [AT1G28370.1]
ERF13	AT2G44840.1	(0.65/0.34*)	tair|ATERF13/EREBP (ETHYLENE-RESPONSIVE ELEMENT BINDING FACTOR 13); DNA binding / transcription factor

			[AT2G44840.1]
ERF15	AT2G31230.1	(0.34/0.55*)	tair|ATERF15 (ETHYLENE-RESPONSIVE ELEMENT BINDING FACTOR 15); DNA binding / transcription activator/ transcription factor [AT2G31230.1]
RAP2.1	AT1G46768.1	(1.24/2.41*)	tair|RAP2.1 (related to AP2 1); DNA binding / transcription factor [AT1G46768.1]
RAP2.2	AT3G14230.2	(1.18/2.14*)	tair|RAP2.2; DNA binding / transcription factor [AT3G14230.2]
RAP2.4	AT1G78080.1	(2.33/1.70*)	tair|RAP2.4 (related to AP2 4); DNA binding / transcription factor [AT1G78080.1]
AT5G65100	AT5G65100.1	(1.05/2.21*)	tair|ethylene insensitive 3 family protein [AT5G65100.1]
AT1G06160	AT1G06160.1	(0.60/2.23*)	tair|ethylene-responsive factor, putative [AT1G06160.1]
ETR1	AT1G66340.1	(1.43/2.07*)	tair|ETR1 (ETHYLENE RESPONSE 1); two-component response regulator [AT1G66340.1]
AT4G32800	AT4G32800.1	(0.88/0.49*)	tair|AP2 domain-containing transcription factor TINY, putative [AT4G32800.1]
AT1G12890	AT1G12890.1	(0.76/0.36*)	tair|AP2 domain-containing transcription factor, putative [AT1G12890.1]
AT4G13620	AT4G13620.1	(0.96/0.50*)	tair|AP2 domain-containing transcription factor, putative [AT4G13620.1]
**GA-related**			
ATGA2OX1	AT1G78440.1	(2.11/1.00*)	tair|ATGA2OX1 (GIBBERELLIN 2-OXIDASE 1); gibberellin 2-beta-dioxygenase [AT1G78440.1]
GAMT2	AT5G56300.1	(0.22/1.15*)	tair|GAMT2; S-adenosylmethionine-dependent methyltransferase/ gibberellin carboxyl-O-methyltransferase [AT5G56300.1]
AT5G59845	AT5G59845.1	(0.46/0.47*)	tair|gibberellin-regulated family protein [AT5G59845.1]
ERF72	AT3G16770.1	(0.44/1.35*)	tair|ATEBP/ERF72/RAP2.3 (RELATED TO AP2 3); DNA binding / protein binding / transcription activator/ transcription factor [AT3G16770.1]
AT1G22690	AT1G22690.1	(0.56/0.47*)	tair|gibberellin-responsive protein, putative [AT1G22690.1]
ATGA2OX2	AT1G30040.1	(0.66/0.48*)	tair|ATGA2OX2; gibberellin 2-beta-dioxygenase [AT1G30040.1]
GASA2	AT4G09610.1	(0.66/0.29*)	tair|GASA2 (GAST1 PROTEIN HOMOLOG 2) [AT4G09610.1]
GASA3	AT4G09600.1	(0.97/0.04*)	tair|GASA3 (GAST1 PROTEIN HOMOLOG 3) [AT4G09600.1]
GASA5	AT3G02885.1	(0.10/0.48*)	tair|GASA5 (GAST1 PROTEIN HOMOLOG 5) [AT3G02885.1]
**AUXIN-related**			
AIR1	AT4G12550.1	(12.25/5.29*)	tair|AIR1 (Auxin-Induced in Root cultures 1); lipid binding [AT4G12550.1]
AT1G29450	AT1G29450.1	(2.40/3.30*)	tair|auxin-responsive protein, putative [AT1G29450.1]
AT1G48690	AT1G48690.1	(3.40/3.41*)	tair|auxin-responsive GH3 family protein [AT1G48690.1]
AT2G04850	AT2G04850.1	(2.24/2.20*)	tair|auxin-responsive protein-related [AT2G04850.1]
AT4G09530	AT4G09530.1	(3.97/2.47*)	tair|auxin-responsive family protein [AT4G09530.1]
AT5G66260	AT5G66260.1	(3.14/3.15*)	tair|auxin-responsive protein, putative [AT5G66260.1]
IAA2	AT3G23030.1	(2.42/3.01*)	tair|IAA2 (indoleacetic acid-induced protein 2); transcription factor [AT3G23030.1]
IAA18	AT1G51950.1	(1.45/1.50*)	tair|IAA18 (indoleacetic acid-induced protein 18); transcription factor [AT1G51950.1]
ILR3	AT5G54680.1	(2.14/2.03*)	tair|ILR3 (IAA-LEUCINE RESISTANT3); DNA binding / transcription factor [AT5G54680.1]
ILL6	AT1G44350.1	(0.35/0.48*)	tair|ILL6 (IAA-leucine resistant (ILR)-like gene 6); metallopeptidase [AT1G44350.1]
AT2G21220	AT2G21220.1	(0.48/1.38*)	tair|auxin-responsive protein, putative [AT2G21220.1]
AT4G34790	AT4G34790.1	(0.47/0.85*)	tair|auxin-responsive family protein [AT4G34790.1]
AT5G13380	AT5G13380.1	(0.26/0.12*)	tair|auxin-responsive GH3 family protein [AT5G13380.1]
AT5G20810	AT5G20810.1	(0.46/0.29*)	tair|auxin-responsive protein, putative / small auxin up RNA (SAUR_B) [AT5G20810.1]

IAA14	AT4G14550.1	(0.36/0.28*)	tair|IAA14 (SOLITARY ROOT); transcription factor [AT4G14550.1]
IAA20	AT2G46990.1	(1.85/3.80*)	tair|IAA20 (indoleacetic acid-induced protein 20); transcription factor [AT2G46990.1]
IAA29	AT4G32280.1	(1.51/2.77*)	tair|IAA29 (indoleacetic acid-induced protein 29); transcription factor [AT4G32280.1]
IAA32	AT2G01200.1	(1.20/2.88*)	tair|IAA32 (INDOLEACETIC ACID-INDUCED PROTEIN 32); transcription factor [AT2G01200.1]
IAA31	AT3G17600.1	(0.87/0.46*)	tair|IAA31 (indoleacetic acid-induced protein 31); transcription factor [AT3G17600.1]
AT1G20925	AT1G20925.1	(1.00/4.01*)	tair|auxin efflux carrier family protein [AT1G20925.1]
AT1G56150	AT1G56150.1	(1.21/2.55*)	tair|auxin-responsive family protein [AT1G56150.1]
AT3G25290	AT3G25290.1	(2.00/2.05*)	tair|auxin-responsive family protein [AT3G25290.1]
AT4G00880	AT4G00880.1	(0.92/4.02*)	tair|auxin-responsive family protein [AT4G00880.1]
AT4G36110	AT4G36110.1	(1.81/2.94*)	tair|auxin-responsive protein, putative [AT4G36110.1]
AT5G18010	AT5G18010.1	(1.52/2.04*)	tair|auxin-responsive protein, putative [AT5G18010.1]
PIN6	AT1G77110.1	(0.68/3.94*)	tair|PIN6 (PIN-FORMED 6); auxin:hydrogen symporter/ transporter [AT1G77110.1]
AT1G56220	AT1G56220.1	(1.55/1.62*)	tair|dormancy/auxin associated family protein [AT1G56220.1]
AT1G48670	AT1G48670.1	(0.74/0.42*)	tair|auxin-responsive GH3 family protein [AT1G48670.1]
AT4G13790	AT4G13790.1	(1.37/0.74*)	tair|auxin-responsive protein, putative [AT4G13790.1]
RAB18	AT5G66400.1	(9.77/0.28*)	tair|RAB18 (RESPONSIVE TO ABA 18) [AT5G66400.1]
**JA-related**			
AT1G52060	AT1G52060.1	(2.80/2.39*)	tair|similar to jacalin lectin family protein [Arabidopsis thaliana] (TAIR:AT1G52070.1); similar to jasmonate inducible protein [Brassica napus] (GB:CAA72271.1); contains InterPro domain Mannose-binding lectin (InterPro:IPR001229) [AT1G52060.1]
AT5G28520	AT5G28520.1	(2.62/2.17*)	tair|similar to jacalin lectin family protein [Arabidopsis thaliana] (TAIR:AT1G33790.1); similar to jasmonate inducible protein [Brassica napus] (GB:CAA72271.1); contains InterPro domain Mannose-binding lectin (InterPro:IPR001229) [AT5G28520.1]
CORI3	AT4G23600.1	(0.15/0.02*)	tair|CORI3 (CORONATINE INDUCED 1, JASMONIC ACID RESPONSIVE 2); transaminase [AT4G23600.1]
LOX1	AT1G55020.1	(0.32/0.60*)	tair|LOX1 (Lipoxygenase 1); lipoxygenase [AT1G55020.1]
LOX2	AT3G45140.1	(0.06/0.11*)	tair|LOX2 (LIPOXYGENASE 2) [AT3G45140.1]
LOX3	AT1G17420.1	(0.94/1.15*)	tair|LOX3 (Lipoxygenase 3); iron ion binding / lipoxygenase/ metal ion binding / oxidoreductase, acting on single donors with incorporation of molecular oxygen, incorporation of two atoms of oxygen [AT1G17420.1]
ATCLH1	AT1G19670.1	(0.31/0.21*)	tair|ATCLH1 (CORONATINE-INDUCED PROTEIN 1) [AT1G19670.1]
JAR1	AT2G46370.1	(0.04/0.08*)	tair|JAR1 (JASMONATE RESISTANT 1) [AT2G46370.1]
JAZ10	AT5G13220.1	(0.86/0.35*)	tair|JAS1/JAZ10/TIFY9 (JASMONATE-ZIM-DOMAIN PROTEIN 10) [AT5G13220.1]
JAZ6	AT1G72450.1	(0.14/0.36*)	tair|JAZ6/TIFY11B (JASMONATE-ZIM-DOMAIN PROTEIN 6) [AT1G72450.1]
JAZ7	AT2G34600.1	(0.68/0.37*)	tair|JAZ7/TIFY5B (JASMONATE-ZIM-DOMAIN PROTEIN 7) [AT2G34600.1]
JAZ8	AT1G30135.1	(1.00/0.14*)	tair|JAZ8/TIFY5A (JASMONATE-ZIM-DOMAIN PROTEIN 8) [AT1G30135.1]
PDF2.3	AT2G02130.1	(0.38/0.39*)	tair|LCR68/PDF2.3 (Low-molecular-weight cysteine-rich 68); protease inhibitor [AT2G02130.1]
PDF2.1	AT2G02120.1	(0.86/0.43*)	tair|LCR70/PDF2.1 (Low-molecular-weight cysteine-rich 70); protease inhibitor [AT2G02120.1]
PDF1.4	AT1G19610.1	(0.36/0.35*)	tair|LCR78/PDF1.4 (Low-molecular-weight cysteine-rich 78) [AT1G19610.1]
VSP1	AT5G24780.2	(1.03/0.15*)	tair|VSP1 (VEGETATIVE STORAGE PROTEIN 1) [AT5G24780.2]
VSP2	AT5G24770.2	(0.94/0.31*)	tair|VSP2 (VEGETATIVE STORAGE PROTEIN 2); acid phosphatase [AT5G24770.2]
**SA-related**			

AT2G21340	AT2G21340.1	(2.70/3.69*)	tair|enhanced disease susceptibility protein, putative / salicylic acid induction deficient protein, putative [AT2G21340.1]
**BR-related**			
BR6OX2	AT3G30180.1	(3.42/2.29*)	tair|BR6OX2/CYP85A2 (BRASSINOSTEROID-6-OXIDASE 2); monooxygenase/ oxygen binding [AT3G30180.1]
BRL3	AT3G13380.1	(0.43/0.39*)	tair|BRL3 (BRI1-LIKE 3); protein binding / protein kinase [AT3G13380.1]
BRS1	AT4G30610.1	(0.48/0.36*)	tair|BRS1 (BRI1 SUPPRESSOR 1) [AT4G30610.1]
BEE1	AT1G18400.1	(0.44/0.88*)	tair|BEE1 (BR ENHANCED EXPRESSION 1); transcription factor [AT1G18400.1]
BEE3	AT1G73830.1	(1.01/2.89*)	tair|BEE3 (BR ENHANCED EXPRESSION 3); DNA binding / transcription factor [AT1G73830.1]
BR6OX1	AT5G38970.1	(1.66/2.81*)	tair|BR6OX1 (BRASSINOSTEROID-6-OXIDASE); oxygen binding [AT5G38970.1]
AT1G78880	AT1G78880.1	(1.78/2.15*)	tair|balbiani ring 1-related / BR1-related [AT1G78880.1]
**Cytokinin-related**			
CRF6	AT3G61630.1	(2.65/4.19*)	tair|CRF6 (CYTOKININ RESPONSE FACTOR 6); DNA binding / transcription factor [AT3G61630.1]
CKX1	AT2G41510.1	(0.48/0.62*)	tair|ATCKX1/CKX1 (CYTOKININ OXIDASE/DEHYDROGENASE 1); cytokinin dehydrogenase [AT2G41510.1]
CKX7	AT5G21482.1	(0.44/0.58*)	tair|CKX7 (CYTOKININ OXIDASE 7); oxidoreductase [AT5G21482.1]
CKX5	AT1G75450.1	(1.18/2.90*)	tair|CKX5 (CYTOKININ OXIDASE 5); cytokinin dehydrogenase [AT1G75450.1]
