# Supplementary material for: Far-Red Light-Mediated Seedling Development in Arabidopsis Involves FAR-RED INSENSITIVE 219/JASMONATE RESISTANT 1-Dependent and -Independent Pathways
Source: PLoS One. 2015 Jul 15;10(7):e0132723. doi: 10.1371/journal.pone.0132723 (PMC4503420; doi:10.1371/journal.pone.0132723)
Supplement: S7 Table — (PDF) [file pone.0132723.s015.pdf]

S7 Table. List of hormone-related genes in *PGR219*/Col.

Gene Name	Systematic Name	expression ratio (-MeJA/+MeJA)	Description
**ABA-related**			
ABI2	AT5G57050.1	(2.27/ 3.05*)	tair|ABI2 (ABA INSENSITIVE 2); protein serine/threonine phosphatase
AtRABA1e	AT4G18430.1	(5.58/ 2.37*)	tair|AtRABA1e (Arabidopsis Rab GTPase homolog A1e); GTP binding
AtRABA6b	AT1G18200.1	(4.03/ 1.17*)	tair|AtRABA6b (Arabidopsis Rab GTPase homolog A6b); GTP binding
ATRAB_ALPHA	AT5G03530.1	(2.82/ 4.71*)	tair|ATRAB ALPHA (Arabidopsis Rab GTPase homolog C2a); GTP binding [AT5G03530.1]
ATRAB11C	AT1G09630.1	(4.62/ 4.37*)	tair|ATRAB11C (ARABIDOPSIS RAB GTPASE HOMOLOG A2A); GTP binding [AT1G09630.1]
AtRABA4a	AT5G65270.1	(1.83/ 2.05*)	tair|AtRABA4a (Arabidopsis Rab GTPase homolog A4a); GTP binding [AT5G65270.1]
AtRABA5e	AT1G05810.1	(0.41/ 0.33*)	tair|ARA/Ara-1/AtRABA5e/AtRab11D (Arabidopsis Rab GTPase homolog A5e); GTP binding [AT1G05810.1]
AtRABA1f	AT5G60860.1	(0.43/ 0.35*)	tair|AtRABA1f (Arabidopsis Rab GTPase homolog A1f); GTP binding [AT5G60860.1]
AtRABA5b	AT3G07410.1	(0.34/ 0.44*)	tair|AtRABA5b (Arabidopsis Rab GTPase homolog A5b); GTP binding [AT3G07410.1]
AtRab1A	AT5G47200.1	(2.44/ 2.11*)	tair|AtRABD2b/AtRab1A (Arabidopsis Rab GTPase homolog D2b); GTP binding [AT5G47200.1]
AtRABH1a	AT5G64990.1	(3.02/ 5.15*)	tair|AtRABH1a (Arabidopsis Rab GTPase homolog H1a); GTP binding [AT5G64990.1]
AtRABH1c	AT4G39890.1	(2.87/ 2.26*)	tair|AtRABH1c (Arabidopsis Rab GTPase homolog H1c); GTP binding [AT4G39890.1]
AtRab8D	AT4G20360.1	(0.38/ 0.32*)	tair|AtRABE1b/AtRab8D (Arabidopsis Rab GTPase homolog E1b); translation elongation factor [AT4G20360.1]
AtRab7A	AT2G21880.1	(0.81/ 0.48*)	tair|AtRABG2/AtRab7A (Arabidopsis Rab GTPase homolog G2); GTP binding [AT2G21880.1]
RAB18	AT5G66400.1	(0.72/ 0.24*)	tair|RAB18 (RESPONSIVE TO ABA 18) [AT5G66400.1]
ABA3	AT1G32090.1	(3.87/ 4.51*)	tair|ABA3/ATABA3/LOS5/SIR3 (ABA DEFICIENT 3); Mo-molybdopterin cofactor sulfurase/ selenocysteine lyase [AT1G16540.1]
ABA4	AT1G67080.1	(0.36/ 0.46*)	tair|ABA4 (ABSCISIC ACID (ABA)-DEFICIENT 4); intramolecular oxidoreductase [AT1G67080.1]
ABF2	AT1G45249.1	(44.05/ 36.16*)	tair|ABF2 (ABSCISIC ACID RESPONSIVE ELEMENTS-BINDING FACTOR 2)
ATRAB18	AT1G43890.2	(2.44/ 2.62*)	tair|ATRAB18 (Arabidopsis Rab GTPase homolog C1)
ATRAB28	AT1G03120.1	(2.96/ 2.61*)	tair|ATRAB28 (Arabidopsis thaliana responsive to abscisic acid 28)
AT1G32090	AT1G32090.1	(3.87/ 4.51*)	tair|early-responsive to dehydration protein-related / ERD protein-related
AT3G21620	AT3G21620.1	(5.13/ 1.64*)	tair|early-responsive to dehydration protein-related / ERD protein-related
AT1G69480	AT1G69480.1	(8.35/ 10.11*)	tair|EXS family protein / ERD1/XPR1/SYG1 family protein [AT1G69480.1]
AT5G35730	AT5G35730.1	(2.72/ 2.78*)	tair|EXS family protein / ERD1/XPR1/SYG1 family protein [AT5G35730.1]
AT5G06278	AT5G06278.1	(2.15/ 1.85*)	tair|pseudogene of abscisic acid-responsive HVA22 family protein [AT5G06278.1]
AT5G08350	AT5G08350.1	(0.26/ 0.19*)	tair|GRAM domain-containing protein / ABA-responsive protein-related [AT5G08350.1]
ABA1	AT5G67030.1	(0.45/ 0.53*)	tair|ABA1 (ABA DEFICIENT 1); zeaxanthin epoxidase [AT5G67030.1]
AT5G23350	AT5G23350.1	(0.47/ 0.20*)	tair|GRAM domain-containing protein / ABA-responsive protein-related [AT5G23350.1]
AT4G15430	AT4G15430.1	(0.28/ 0.33*)	tair|similar to early-responsive to dehydration protein-related / ERD protein-related [Arabidopsis thaliana] (TAIR:AT4G04340.1); similar to early-responsive to dehydration protein-related / ERD protein-related [Arabidopsis thaliana] (TAIR:AT4G04340.3); si
ERD14	AT1G76180.1	(0.35/ 0.36*)	tair|ERD14 (EARLY RESPONSE TO DEHYDRATION 14) [AT1G76180.1]

ERD5	AT3G30775.1	(0.26/ 0.11*)	tair|ERD5 (EARLY RESPONSIVE TO DEHYDRATION 5); proline dehydrogenase [AT3G30775.1]
AT3G54510	AT3G54510.1	(0.32/ 0.31*)	tair|early-responsive to dehydration protein-related / ERD protein-related [AT3G54510.1]
NCED9	AT1G78390.1	(2.44/ 8.30*)	tair|NCED9 (NINE-CIS-EPOXYCAROTENOID DIOXYGENASE 9) [AT1G78390.1]
ABI4	AT2G40220.1	(4.00/ 2.98*)	tair|ABI4 (ABA INSENSITIVE 4); DNA binding / transcription factor [AT2G40220.1]
ERD10	AT1G20450.1	(5.53/ 6.15*)	tair|ERD10/LTI45 (EARLY RESPONSIVE TO DEHYDRATION 10) [AT1G20450.1]
AT1G32090	AT1G32090.1	(3.87/ 4.51*)	tair|early-responsive to dehydration protein-related / ERD protein-related [AT1G32090.1]
AT3G02480	AT3G02480.1	(1.10/ 2.89*)	tair|ABA-responsive protein-related [AT3G02480.1]
DREB2A	AT5G05410.2	(1.50/ 2.72*)	tair|DREB2A (DRE-BINDING PROTEIN 2A); DNA binding / transcription activator/ transcription factor [AT5G05410.2]
AT1G54720	AT1G54720.1	(0.67/ 0.40*)	tair|early-responsive to dehydration protein-related / ERD protein-related [AT1G54720.1]
**Ethylene-related**			
ACS12	AT5G51690.1	(75.89/ 81.00*)	tair|ACS12 (1-Amino-cyclopropane-1-carboxylate synthase 12); 1-aminocyclopropane-1-carboxylate synthase [AT5G51690.1]
ACS4	AT2G22810.1	(8.26/ 87.83*)	tair|ACS4 (1-AMINOCYCLOPROPANE-1-CARBOXYLATE SYNTHASE 4); 1-aminocyclopropane-1-carboxylate synthase [AT2G22810.1]
ACS6	AT4G11280.1	(2.19/ 5.57*)	tair|ACS6 (1-AMINOCYCLOPROPANE-1-CARBOXYLIC ACID (ACC) SYNTHASE 6) [AT4G11280.1]
ACS7	AT4G26200.1	(12.43/ 3.75*)	tair|ACS7 (1-Amino-cyclopropane-1-carboxylate synthase 7); 1-aminocyclopropane-1-carboxylate synthase [AT4G26200.1]
ACS10	AT1G62960.1	(0.37/ 0.47*)	tair|ACS10 (ACC SYNTHASE 10); 1-aminocyclopropane-1-carboxylate synthase [AT1G62960.1]
ACS11	AT4G08040.1	(0.29/ 0.43*)	tair|ACS11 (1-Amino-cyclopropane-1-carboxylate synthase 11); 1-aminocyclopropane-1-carboxylate synthase [AT4G08040.1]
ACS2	AT1G01480.1	(0.42/ 0.35*)	tair|ACS2 (1-Amino-cyclopropane-1-carboxylate synthase 2) [AT1G01480.1]
AT1G12010	AT1G12010.1	(13.95/ 14.56*)	tair|1-aminocyclopropane-1-carboxylate oxidase, putative / ACC oxidase, putative [AT1G12010.1]
AT5G61600	AT5G61600.1	(2.17/ 2.04*)	tair|ethylene-responsive element-binding family protein [AT5G61600.1]
AT1G06160	AT1G06160.1	(22.92/ 35.22*)	tair|ethylene-responsive factor, putative [AT1G06160.1]
AT1G49830	AT1G49830.1	(9.25/ 10.76*)	tair|ethylene-responsive protein -related [AT1G49830.1]
AT1G05710	AT1G05710.1	(3.99/ 3.40*)	tair|ethylene-responsive protein, putative [AT1G05710.1]
ETR1	AT1G66340.1	(3.27/ 2.31*)	tair|ETR1 (ETHYLENE RESPONSE 1); two-component response regulator [AT1G66340.1]
ERD7	AT2G17840.1	(2.58/ 1.56*)	tair|ERD7 (EARLY-RESPONSIVE TO DEHYDRATION 7) [AT2G17840.1]
ERS2	AT1G04310.1	(2.34/ 1.25*)	tair|ERS2 (ETHYLENE RESPONSE SENSOR 2); receptor [AT1G04310.1]
EIN4	AT3G04580.1	(4.57/ 4.83*)	tair|EIN4 (ETHYLENE INSENSITIVE 4); receptor [AT3G04580.1]
EFE	AT1G05010.1	(1.79/ 2.16*)	tair|EFE (ETHYLENE FORMING ENZYME) [AT1G05010.1]
EICBP.B	AT5G09410.1	(1.90/ 2.19*)	tair|EICBP.B (ETHYLENE INDUCED CALMODULIN BINDING PROTEIN); calmodulin binding / transcription regulator [AT5G09410.1]
ACO1	AT2G19590.1	(12.23/ 6.93*)	tair|ACO1 (ACC OXIDASE 1); 1-aminocyclopropane-1-carboxylate oxidase [AT2G19590.1]
ATERF-4/ATERF4/ERF4	AT3G15210.1	(2.01/ 2.64*)	tair|ATERF-4/ATERF4/ERF4/RAP2.5 (ETHYLENE RESPONSIVE ELEMENT BINDING FACTOR 4); DNA binding / protein binding / transcription factor/ transcription repressor [AT3G15210.1]
ATERF-2/ATERF2/ERF2	AT5G47220.1	(1.30/ 2.29*)	tair|ATERF-2/ATERF2/ERF2 (ETHYLENE RESPONSE FACTOR 2); DNA binding / transcription activator/

			transcription factor [AT5G47220.1]
ATERF#011/CEJ1	AT3G50260.1	(17.50/ 14.03*)	tair|ATERF#011/CEJ1 (COOPERATIVELY REGULATED BY ETHYLENE AND JASMONATE 1); DNA binding / transcription factor [AT3G50260.1]
ATERF-1	AT4G17500.1	(1.67/ 2.38*)	tair|ATERF-1 (ETHYLENE RESPONSIVE ELEMENT BINDING FACTOR 1); DNA binding / transcription activator/ transcription factor [AT4G17500.1]
ATERF11/ERF11	AT1G28370.1	(3.47/ 3.84*)	tair|ATERF11/ERF11 (ERF domain protein 11); DNA binding / transcription factor/ transcription repressor [AT1G28370.1]
AT2G38340	AT2G38340.1	(4.96/ 5.06*)	tair|AP2 domain-containing transcription factor, putative (DRE2B) [AT2G38340.1]
AT2G40340	AT2G40340.1	(4.98/ 3.31*)	tair|AP2 domain-containing transcription factor, putative (DRE2B) [AT2G40340.1]
AT5G51190	AT5G51190.1	(2.98/ 6.12*)	tair|AP2 domain-containing transcription factor, putative [AT5G51190.1]
AT5G61890	AT5G61890.1	(5.39/ 3.59*)	tair|AP2 domain-containing transcription factor family protein [AT5G61890.1]
AT1G33760	AT1G33760.1	(23.98/ 29.51*)	tair|AP2 domain-containing transcription factor, putative [AT1G33760.1]
AT2G22200	AT2G22200.1	(3.17/ 0.91*)	tair|AP2 domain-containing transcription factor [AT2G22200.1]
RAP2.2	AT3G14230.2	(4.09/ 3.77*)	tair|RAP2.2; DNA binding / transcription factor [AT3G14230.2]
RAP2.4	AT1G78080.1	(2.21/ 1.93*)	tair|RAP2.4 (related to AP2 4); DNA binding / transcription factor [AT1G78080.1]
RAP2.9	AT4G06746.1	(2.74/ 3.39*)	tair|RAP2.9 (related to AP2 9); transcription factor [AT4G06746.1]
RAP2.11	AT5G19790.1	(2.43/ 0.76*)	tair|RAP2.11 (related to AP2 11); DNA binding / transcription factor [AT5G19790.1]
AT1G72360	AT1G72360.1	(0.50/ 0.65*)	tair|ethylene-responsive element-binding protein, putative [AT1G72360.1]
RAP2.10	AT4G36900.1	(0.21/ 0.21*)	tair|RAP2.10 (related to AP2 10); DNA binding / transcription factor [AT4G36900.1]
AT1G01250	AT1G01250.1	(0.45/ 0.54*)	tair|AP2 domain-containing transcription factor, putative [AT1G01250.1]
AT1G12890	AT1G12890.1	(0.43/ 0.27*)	tair|AP2 domain-containing transcription factor, putative [AT1G12890.1]
AT1G63040	AT1G63040.1	(0.43/ 0.39*)	tair|a pseudogene member of the DREB subfamily A-4 of ERF/AP2 transcription factor family. The translated product contains one AP2 domain. There are 17 members in this subfamily including TINY. [AT1G63040.1]
AT1G64380	AT1G64380.1	(0.38/ 0.69*)	tair|AP2 domain-containing transcription factor, putative [AT1G64380.1]
EGY1	AT5G35220.1	(0.31/ 0.35*)	tair|EGY1 (ETHYLENE-DEPENDENT GRAVITROPISM-DEFICIENT AND YELLOW-GREEN 1); sterol regulatory element-binding protein site 2 protease [AT5G35220.1]
AT5G10120	AT5G10120.1	(1.43/ 6.02*)	tair|ethylene insensitive 3 family protein [AT5G10120.1]
AT5G65100	AT5G65100.1	(0.54/ 4.22*)	tair|ethylene insensitive 3 family protein [AT5G65100.1]
AT1G80580	AT1G80580.1	(1.67/ 2.06*)	tair|ethylene-responsive element-binding family protein [AT1G80580.1]
AT5G43410	AT5G43410.1	(1.31/ 5.43*)	tair|ethylene-responsive factor, putative [AT5G43410.1]
AHK5	AT5G10720.1	(2.17/ 2.35*)	tair|AHK5 (CYTOKININ INDEPENDENT 2) [AT5G10720.1]
AT4G13040	AT4G13040.1	(1.91/ 2.02*)	tair|AP2 domain-containing transcription factor family protein [AT4G13040.1]
AT2G44940	AT2G44940.1	(0.71/ 2.35*)	tair|AP2 domain-containing transcription factor TINY, putative [AT2G44940.1]
AT4G32800	AT4G32800.1	(1.59/ 2.38*)	tair|AP2 domain-containing transcription factor TINY, putative [AT4G32800.1]
AT2G47520	AT2G47520.1	(1.81/ 2.99*)	tair|AP2 domain-containing transcription factor, putative [AT2G47520.1]
AT3G57600	AT3G57600.1	(1.48/ 2.19*)	tair|AP2 domain-containing transcription factor, putative [AT3G57600.1]
AT5G18450	AT5G18450.1	(1.42/ 2.62*)	tair|AP2 domain-containing transcription factor, putative [AT5G18450.1]
AT1G44830	AT1G44830.1	(0.89/ 0.47*)	tair|AP2 domain-containing transcription factor TINY, putative [AT1G44830.1]

AT1G36060	AT1G36060.1	(0.64/ 0.26*)	tair|AP2 domain-containing transcription factor, putative [AT1G36060.1]
AT4G13620	AT4G13620.1	(0.62/ 0.50*)	tair|AP2 domain-containing transcription factor, putative [AT4G13620.1]
ATEBP/ERF72	AT3G16770.1	(0.64/ 0.41*)	tair|ATEBP/ERF72/RAP2.3 (RELATED TO AP2 3); DNA binding / protein binding / transcription activator/ transcription factor [AT3G16770.1]
AT2G31730	AT2G31730.1	(0.63/ 0.31*)	tair|ethylene-responsive protein, putative [AT2G31730.1]
**GA-related**			
ATGID1B/GID1B	AT3G63010.1	(6.77/ 3.62*)	tair|ATGID1B/GID1B (GA INSENSITIVE DWARF1B); hydrolase [AT3G63010.1]
AT5G51310	AT5G51310.1	(6.64/ 1.86*)	tair|gibberellin 20-oxidase-related [AT5G51310.1]
GA1	AT4G02780.1	(11.06/ 10.99*)	tair|GA1 (GA REQUIRING 1); ent-copalyl diphosphate synthase [AT4G02780.1]
GA5	AT4G25420.1	(6.63/ 6.20*)	tair|GA5 (GA REQUIRING 5); gibberellin 20-oxidase/ gibberellin 3-beta-dioxygenase [AT4G25420.1]
ATGA2OX2	AT1G30040.1	(2.99/ 1.96*)	tair|ATGA2OX2; gibberellin 2-beta-dioxygenase [AT1G30040.1]
GAMT2	AT5G56300.1	(0.15/ 0.73*)	tair|GAMT2; S-adenosylmethionine-dependent methyltransferase/ gibberellin carboxyl-O-methyltransferase [AT5G56300.1]
ATGA2OX1	AT1G78440.1	(0.18/ 1.00*)	tair|ATGA2OX1 (GIBBERELLIN 2-OXIDASE 1); gibberellin 2-beta-dioxygenase [AT1G78440.1]
ATGA2OX4	AT1G47990.1	(0.40/ 0.99*)	tair|ATGA2OX4 (GIBBERELLIN 2-OXIDASE 4); gibberellin 2-beta-dioxygenase [AT1G47990.1]
AT1G22690	AT1G22690.1	(0.07/ 0.10*)	tair|gibberellin-responsive protein, putative [AT1G22690.1]
AT5G59845	AT5G59845.1	(0.29/ 0.51*)	tair|gibberellin-regulated family protein [AT5G59845.1]
PAP2	AT4G29080.1	(0.40/ 0.60*)	tair|PAP2 (PHYTOCHROME-ASSOCIATED PROTEIN 2); transcription factor [AT4G29080.1]
PAP2	AT1G66390.1	(0.43/ 2.54*)	tair|PAP2 (PRODUCTION OF ANTHOCYANIN PIGMENT 2); DNA binding / transcription factor [AT1G66390.1]
GASA5	AT3G02885.1	(0.21/ 0.44*)	tair|GASA5 (GAST1 PROTEIN HOMOLOG 5) [AT3G02885.1]
AGL102	AT1G47760.1	(12.99/ 19.96*)	tair|AGL102; DNA binding / transcription factor [AT1G47760.1]
AGL12	AT1G71692.1	(1.06/ 2.25*)	tair|AGL12 (AGAMOUS-LIKE 12); transcription factor [AT1G71692.1]
AGL14	AT4G11880.1	(1.75/ 2.13*)	tair|AGL14 (AGAMOUS-LIKE 14); DNA binding / transcription factor [AT4G11880.1]
AGL17	AT2G22630.1	(2.27/ 4.58*)	tair|AGL17 (AGAMOUS-LIKE 17); transcription factor [AT2G22630.1]
AGL31	AT5G65050.1	(20.54/ 32.49*)	tair|AGL31 (AGAMOUS LIKE MADS-BOX PROTEIN 31); transcription factor [AT5G65050.1]
AGL39	AT5G27130.1	(1.22/ 2.66*)	tair|AGL39; DNA binding / transcription factor [AT5G27130.1]
AGL64	AT1G29960.1	(2.21/ 2.37*)	tair|AGL64; peptidase [AT1G29960.1]
AGL67	AT1G77950.1	(1.00/ 3.40*)	tair|AGL67; transcription factor [AT1G77950.1]
AGL80/FEM111	AT5G48670.1	(1.66/ 2.01*)	tair|AGL80/FEM111 (AGAMOUS-LIKE80); DNA binding / transcription factor [AT5G48670.1]
AGL99	AT5G04640.1	(12.84/ 3.72*)	tair|AGL99; DNA binding / transcription factor [AT5G04640.1]
AT2G14900	AT2G14900.1	(1.06/ 0.46*)	tair|gibberellin-regulated family protein [AT2G14900.1]
AT5G14920	AT5G14920.1	(1.50/ 0.47*)	tair|gibberellin-regulated family protein [AT5G14920.1]
AT2353/ATGA20OX2/GA20OX2	AT5G51810.1	(0.52/ 0.41*)	tair|AT2353/ATGA20OX2/GA20OX2 (GIBBERELLIN 20 OXIDASE 2); gibberellin 20-oxidase [AT5G51810.1]
ATGA2OX3	AT2G34555.1	(0.27/ 0.19*)	tair|ATGA2OX3 (GIBBERELLIN 2-OXIDASE 3); gibberellin 2-beta-dioxygenase [AT2G34555.1]
GASA1	AT1G75750.1	(1.15/ 0.24*)	tair|GASA1 (GAST1 PROTEIN HOMOLOG 1) [AT1G75750.1]
GASA2	AT4G09610.1	(1.34/ 0.25*)	tair|GASA2 (GAST1 PROTEIN HOMOLOG 2) [AT4G09610.1]
GASA3	AT4G09600.1	(0.71/ 0.24*)	tair|GASA3 (GAST1 PROTEIN HOMOLOG 3) [AT4G09600.1]
AGL19	AT4G22950.1	(1.02/ 0.46*)	tair|AGL19 (AGAMOUS-LIKE 19); transcription factor [AT4G22950.1]

AGL20	AT2G45660.1	(0.41/ 0.31*)	tair|AGL20 (AGAMOUS-LIKE 20); transcription factor [AT2G45660.1]
**AUXIN-related**			
PIN5	AT5G16530.1	(2.12/ 3.97*)	tair|PIN5 (PIN-FORMED 5); auxin:hydrogen symporter/ transporter [AT5G16530.1]
EIR1	AT5G57090.1	(2.61/ 1.60*)	tair|EIR1 (ETHYLENE INSENSITIVE ROOT 1); auxin:hydrogen symporter/ transporter [AT5G57090.1]
AT1G20925	AT1G20925.1	(11.53/ 4.66*)	tair|auxin efflux carrier family protein [AT1G20925.1]
AT1G76520	AT1G76520.1	(3.02/ 3.29*)	tair|auxin efflux carrier family protein [AT1G76520.1]
AT1G56150	AT1G56150.1	(8.99/ 18.15*)	tair|auxin-responsive family protein [AT1G56150.1]
AT1G75590	AT1G75590.1	(16.23/ 13.87*)	tair|auxin-responsive family protein [AT1G75590.1]
AT3G61900	AT3G61900.1	(2.55/ 4.96*)	tair|auxin-responsive family protein [AT3G61900.1]
AT5G35735	AT5G35735.1	(2.22/ 2.63*)	tair|auxin-responsive family protein [AT5G35735.1]
AT5G42410	AT5G42410.1	(13.51/ 12.44*)	tair|auxin-responsive family protein [AT5G42410.1]
AT1G48690	AT1G48690.1	(3.30/ 2.11*)	tair|auxin-responsive GH3 family protein [AT1G48690.1]
AT1G43040	AT1G43040.1	(6.78/ 5.84*)	tair|auxin-responsive protein, putative [AT1G43040.1]
AT4G36110	AT4G36110.1	(1.41/ 2.24*)	tair|auxin-responsive protein, putative [AT4G36110.1]
AT5G66260	AT5G66260.1	(7.97/ 4.26*)	tair|auxin-responsive protein, putative [AT5G66260.1]
AT2G45210	AT2G45210.1	(4.03/ 2.78*)	tair|auxin-responsive protein-related [AT2G45210.1]
AT5G20820	AT5G20820.1	(3.21/ 2.10*)	tair|auxin-responsive protein-related [AT5G20820.1]
AIR1	AT4G12550.1	(5.23/ 4.87*)	tair|AIR1 (Auxin-Induced in Root cultures 1); lipid binding [AT4G12550.1]
IAR4	AT1G24180.1	(2.47/ 2.05*)	tair|IAR4 (IAA-conjugate-resistant 4); pyruvate dehydrogenase (acetyl-transferring) [AT1G24180.1]
ILR2	AT3G18485.1	(0.90/ 1.66*)	tair|ILR2 (IAA-LEUCINE RESISTANT 2) [AT3G18485.1]
ILL5	AT1G51780.1	(2.43/ 3.47*)	tair|ILL5 (IAA-leucine resistant (ILR)-like gene 5); metallopeptidase [AT1G51780.1]
IAR3	AT1G51760.1	(2.75/ 4.02*)	tair|IAR3 (IAA-ALANINE RESISTANT 3); metallopeptidase [AT1G51760.1]
GH3.1	AT2G14960.1	(3.72/ 4.35*)	tair|GH3.1 [AT2G14960.1]
AT3G12955	AT3G12955.1	(0.49/ 0.65*)	tair|auxin-responsive protein-related [AT3G12955.1]
PIN3	AT1G70940.1	(0.61/ 0.46*)	tair|PIN3 (PIN-FORMED 3); auxin:hydrogen symporter/ transporter [AT1G70940.1]
AT5G18050	AT5G18050.1	(0.48/ 0.31*)	tair|auxin-responsive protein, putative [AT5G18050.1]
AT1G17345	AT1G17345.1	(0.47/ 0.42*)	tair|auxin-responsive protein-related [AT1G17345.1]
AT5G20810	AT5G20810.1	(0.28/ 0.61*)	tair|auxin-responsive protein, putative / small auxin up RNA (SAUR_B) [AT5G20810.1]
AT1G75580	AT1G75580.1	(0.46/ 0.56*)	tair|auxin-responsive protein, putative [AT1G75580.1]
AT2G33830	AT2G33830.2	(0.88/ 0.39*)	tair|dormancy/auxin associated family protein [AT2G33830.2]
AT4G34770	AT4G34770.1	(0.41/ 0.48*)	tair|auxin-responsive family protein [AT4G34770.1]
AT4G12410	AT4G12410.1	(0.41/ 0.29*)	tair|auxin-responsive family protein [AT4G12410.1]
AT4G34750	AT4G34750.1	(0.33/ 0.36*)	tair|auxin-responsive protein, putative / small auxin up RNA (SAUR_E) [AT4G34750.1]
AT5G18030	AT5G18030.1	(0.39/ 0.22*)	tair|auxin-responsive protein, putative [AT5G18030.1]
AT1G76190	AT1G76190.1	(0.39/ 0.42*)	tair|auxin-responsive family protein [AT1G76190.1]
SAUR_AC1	AT4G38850.1	(0.38/ 0.28*)	tair|SAUR_AC1 (SMALL AUXIN UP RNA 1 FROM ARABIDOPSIS THALIANA ECOTYPE COLUMBIA) [AT4G38850.1]
AT1G29430	AT1G29430.1	(0.38/ 0.51*)	tair|auxin-responsive family protein [AT1G29430.1]

AT2G16580	AT2G16580.1	(0.37/ 0.85*)	tair|auxin-responsive protein, putative [AT2G16580.1]
AT2G46690	AT2G46690.1	(0.35/ 0.24*)	tair|auxin-responsive family protein [AT2G46690.1]
AT1G29450	AT1G29450.1	(0.35/ 0.49*)	tair|auxin-responsive protein, putative [AT1G29450.1]
AT4G34800	AT4G34800.1	(0.35/ 0.20*)	tair|auxin-responsive family protein [AT4G34800.1]
AT2G33830	AT2G33830.1	(0.88/ 0.39*)	tair|dormancy/auxin associated family protein [AT2G33830.1]
AT3G12830	AT3G12830.1	(0.33/ 0.36*)	tair|auxin-responsive family protein [AT3G12830.1]
AT5G47530	AT5G47530.1	(0.32/ 0.35*)	tair|auxin-responsive protein, putative [AT5G47530.1]
AT1G16510	AT1G16510.1	(0.32/ 0.34*)	tair|auxin-responsive family protein [AT1G16510.1]
AT4G38825	AT4G38825.1	(0.31/ 0.31*)	tair|similar to auxin-responsive protein, putative [Arabidopsis thaliana] (TAIR:AT5G18030.1); similar to auxin-induced protein-like [Brassica rapa] (GB:ABL97983.1); contains InterPro domain Auxin responsive SAUR protein (InterPro:IPR003676) [AT4G38825.1]
AT1G20470	AT1G20470.1	(0.30/ 0.39*)	tair|auxin-responsive family protein [AT1G20470.1]
AT2G37030	AT2G37030.1	(0.28/ 0.37*)	tair|auxin-responsive family protein [AT2G37030.1]
AT5G18020	AT5G18020.1	(0.25/ 0.23*)	tair|auxin-responsive protein, putative [AT5G18020.1]
AT1G11803	AT1G11803.1	(0.23/ 0.16*)	tair|pseudogene, auxin responsive protein, blastp match of 74% identity and 1.2e-15 P-value to GP [AT1G11803.1]
AT1G76530	AT1G76530.1	(0.22/ 0.36*)	tair|auxin efflux carrier family protein [AT1G76530.1]
AT1G29460	AT1G29460.1	(0.20/ 0.22*)	tair|auxin-responsive protein, putative [AT1G29460.1]
AT4G38840	AT4G38840.1	(0.19/ 0.25*)	tair|auxin-responsive protein, putative [AT4G38840.1]
AT3G61750	AT3G61750.1	(0.18/ 0.29*)	tair|auxin-responsive protein -related [AT3G61750.1]
AT2G21220	AT2G21220.1	(0.17/ 0.36*)	tair|auxin-responsive protein, putative [AT2G21220.1]
AT1G79130	AT1G79130.1	(0.17/ 0.47*)	tair|auxin-responsive family protein [AT1G79130.1]
AT4G34790	AT4G34790.1	(0.17/ 0.35*)	tair|auxin-responsive family protein [AT4G34790.1]
AT5G13380	AT5G13380.1	(0.16/ 0.15*)	tair|auxin-responsive GH3 family protein [AT5G13380.1]
AT1G29440	AT1G29440.1	(0.15/ 0.20*)	tair|auxin-responsive family protein [AT1G29440.1]
AT3G53250	AT3G53250.1	(0.12/ 1.00*)	tair|auxin-responsive family protein [AT3G53250.1]
AT1G29500	AT1G29500.1	(0.11/ 0.09*)	tair|auxin-responsive protein, putative [AT1G29500.1]
AT3G03820	AT3G03820.1	(0.10/ 0.15*)	tair|auxin-responsive protein, putative [AT3G03820.1]
AT4G38860	AT4G38860.1	(0.10/ 0.09*)	tair|auxin-responsive protein, putative [AT4G38860.1]
SAUR68	AT1G29510.1	(0.10/ 0.08*)	tair|SAUR68 (SMALL AUXIN UPREGULATED 68) [AT1G29510.1]
AT4G34760	AT4G34760.1	(0.08/ 0.08*)	tair|auxin-responsive family protein [AT4G34760.1]
ILL6	AT1G44350.1	(0.09/ 0.29*)	tair|ILL6 (IAA-leucine resistant (ILR)-like gene 6); metallopeptidase [AT1G44350.1]
IAMT1	AT5G55250.1	(0.61/ 0.43*)	tair|IAMT1 (IAA CARBOXYLMETHYLTRANSFERASE 1); S-adenosylmethionine-dependent methyltransferase [AT5G55250.1]
RBCS1A	AT1G67090.1	(0.28/ 0.32*)	tair|RBCS1A; ribulose-bisphosphate carboxylase [AT1G67090.1]
GH3.9	AT2G47750.1	(0.44/ 0.68*)	tair|GH3.9 (PUTATIVE INDOLE-3-ACETIC ACID-AMIDO SYNTHETASE GH3.9) [AT2G47750.1]
PIN6	AT1G77110.1	(1.02/ 5.56*)	tair|PIN6 (PIN-FORMED 6); auxin:hydrogen symporter/ transporter [AT1G77110.1]
AT3G25290	AT3G25290.1	(1.37/ 2.54*)	tair|auxin-responsive family protein [AT3G25290.1]

AT4G00880	AT4G00880.1	(0.60/ 3.80*)	tair|auxin-responsive family protein [AT4G00880.1]
AT5G53590	AT5G53590.1	(1.21/ 2.07*)	tair|auxin-responsive family protein [AT5G53590.1]
AT3G03850	AT3G03850.1	(4.49/ 8.17*)	tair|auxin-responsive protein, putative [AT3G03850.1]
ARF10	AT2G28350.1	(2.89/ 2.42*)	tair|ARF10 (AUXIN RESPONSE FACTOR 10); miRNA binding / transcription factor [AT2G28350.1]
AIR12	AT3G07390.1	(0.74/ 2.03*)	tair|AIR12 (Auxin-Induced in Root cultures 12); extracellular matrix structural constituent [AT3G07390.1]
AIR3	AT2G04160.1	(1.45/ 2.04*)	tair|AIR3 (Auxin-Induced in Root cultures 3); subtilase [AT2G04160.1]
GH3.3	AT2G23170.1	(1.84/ 2.72*)	tair|GH3.3; indole-3-acetic acid amido synthetase [AT2G23170.1]
IAA18	AT1G51950.1	(2.58/ 3.68*)	tair|IAA18 (indoleacetic acid-induced protein 18); transcription factor [AT1G51950.1]
IAA20	AT2G46990.1	(1.93/ 2.88*)	tair|IAA20 (indoleacetic acid-induced protein 20); transcription factor [AT2G46990.1]
IAA29	AT4G32280.1	(2.74/ 3.12*)	tair|IAA29 (indoleacetic acid-induced protein 29); transcription factor [AT4G32280.1]
ILR3	AT5G54680.1	(2.78/ 2.97*)	tair|ILR3 (IAA-LEUCINE RESISTANT3); DNA binding / transcription factor [AT5G54680.1]
AT4G22620	AT4G22620.1	(0.75/ 0.35*)	tair|auxin-responsive family protein [AT4G22620.1]
AT5G03310	AT5G03310.1	(0.82/ 0.45*)	tair|auxin-responsive family protein [AT5G03310.1]
AT2G21200	AT2G21200.1	(0.54/ 0.49*)	tair|auxin-responsive protein, putative [AT2G21200.1]
AT2G21210	AT2G21210.1	(0.17/ 0.21*)	tair|auxin-responsive protein, putative [AT2G21210.1]
AT4G13790	AT4G13790.1	(0.77/ 0.28*)	tair|auxin-responsive protein, putative [AT4G13790.1]
AT5G18060	AT5G18060.1	(0.58/ 0.31*)	tair|auxin-responsive protein, putative [AT5G18060.1]
AT1G72430	AT1G72430.1	(0.58/ 0.37*)	tair|auxin-responsive protein-related [AT1G72430.1]
AXR3	AT1G04250.1	(0.28/ 0.19*)	tair|AXR3 (AUXIN RESISTANT 3); transcription factor [AT1G04250.1]
IAA1	AT4G14560.1	(0.27/ 0.39*)	tair|IAA1 (INDOLE-3-ACETIC ACID INDUCIBLE); transcription factor [AT4G14560.1]
IAA14	AT4G14550.1	(0.13/ 0.14*)	tair|IAA14 (SOLITARY ROOT); transcription factor [AT4G14550.1]
IAA19	AT3G15540.1	(0.28/ 0.29*)	tair|IAA19 (indoleacetic acid-induced protein 19); transcription factor [AT3G15540.1]
IAA30	AT3G62100.1	(0.17/ 0.33*)	tair|IAA30 (indoleacetic acid-induced protein 30); transcription factor [AT3G62100.1]
IAA31	AT3G17600.1	(0.65/ 0.45*)	tair|IAA31 (indoleacetic acid-induced protein 31); transcription factor [AT3G17600.1]
IAA32	AT2G01200.1	(0.38/ 0.42*)	tair|IAA32 (INDOLEACETIC ACID-INDUCED PROTEIN 32); transcription factor [AT2G01200.1]
IAA34	AT1G15050.1	(0.39/ 0.19*)	tair|IAA34 (indoleacetic acid-induced protein 34); transcription factor [AT1G15050.1]
IAA6	AT1G52830.1	(0.24/ 0.39*)	tair|IAA6 (indoleacetic acid-induced protein 6); transcription factor [AT1G52830.1]
ATAUX2-11	AT5G43700.1	(0.27/ 0.30*)	tair|ATAUX2-11 (indoleacetic acid-induced protein 4); transcription factor [AT5G43700.1]
**JA-related**			
JAZ12/TIFY3B	AT5G20900.1	(2.06/ 2.10*)	tair|JAZ12/TIFY3B (JASMONATE-ZIM-DOMAIN PROTEIN 12) [AT5G20900.1]
JAZ11/TIFY3A	AT3G43440.1	(2.39/ 1.75*)	tair|JAZ11/TIFY3A (JASMONATE-ZIM-DOMAIN PROTEIN 11) [AT3G43440.1]
JAZ4/TIFY6A	AT1G48500.1	(3.22/ 2.06*)	tair|JAZ4/TIFY6A (JASMONATE-ZIM-DOMAIN PROTEIN 4) [AT1G48500.1]
JAR1	AT2G46370.1	(5.37/ 3.09*)	tair|JAR1 (JASMONATE RESISTANT 1) [AT2G46370.1]
JAS1/JAZ10	AT5G13220.1	(12.60/ 1.00*)	tair|JAS1/JAZ10/TIFY9 (JASMONATE-ZIM-DOMAIN PROTEIN 10) [AT5G13220.1]
JAZ8/TIFY5A	AT1G30135.1	(9.81/ 3.42*)	tair|JAZ8/TIFY5A (JASMONATE-ZIM-DOMAIN PROTEIN 8) [AT1G30135.1]
JAS1/JAZ10	AT5G13220.2	(12.60/ 1.00*)	tair|JAS1/JAZ10/TIFY9 (JASMONATE-ZIM-DOMAIN PROTEIN 10) [AT5G13220.2]
AOC3	AT3G25780.1	(3.50/ 1.88*)	tair|AOC3 (ALLENE OXIDE CYCLASE 3) [AT3G25780.1]
PDF1.2	AT5G44420.1	(19.39/ 26.04*)	tair|PDF1.2 (Low-molecular-weight cysteine-rich 77) [AT5G44420.1]

VSP1	AT5G24780.1	(3.08/ 0.75*)	tair|VSP1 (VEGETATIVE STORAGE PROTEIN 1); acid phosphatase [AT5G24780.1]
AT3G21380	AT3G21380.1	(0.35/ 0.52*)	tair|similar to MBP1 (MYROSINASE-BINDING PROTEIN 1) [Arabidopsis thaliana] (TAIR:AT1G52040.1); similar to jasmonate inducible protein [Brassica napus] (GB:CAA72270.1); contains InterPro domain Mannose-binding lectin (InterPro:IPR001229) [AT3G21380.1]
JAZ9/TIFY7	AT1G70700.1	(0.16/ 0.29*)	tair|JAZ9/TIFY7 (JASMONATE-ZIM-DOMAIN PROTEIN 9) [AT1G70700.1]
JAZ7/TIFY5B	AT2G34600.1	(0.47/ 0.10*)	tair|JAZ7/TIFY5B (JASMONATE-ZIM-DOMAIN PROTEIN 7) [AT2G34600.1]
JAI3/JAZ3	AT3G17860.1	(0.29/ 0.55*)	tair|JAI3/JAZ3/TIFY6B (JASMONATE-ZIM-DOMAIN PROTEIN 3) [AT3G17860.1]
JAZ6/TIFY11B	AT1G72450.1	(0.37/ 0.87*)	tair|JAZ6/TIFY11B (JASMONATE-ZIM-DOMAIN PROTEIN 6) [AT1G72450.1]
LOX1	AT1G55020.1	(0.34/ 2.97*)	tair|LOX1 (Lipoxygenase 1); lipoxygenase [AT1G55020.1]
CORI3	AT4G23600.1	(0.19/ 0.43*)	tair|CORI3 (CORONATINE INDUCED 1, JASMONIC ACID RESPONSIVE 2); transaminase [AT4G23600.1]
JAZ1/TIFY10A	AT1G19180.1	(1.12/ 2.37*)	tair|JAZ1/TIFY10A (JASMONATE-ZIM-DOMAIN PROTEIN 1); protein binding [AT1G19180.1]
JAZ5/TIFY11A	AT1G17380.1	(1.31/ 2.70*)	tair|JAZ5/TIFY11A (JASMONATE-ZIM-DOMAIN PROTEIN 5) [AT1G17380.1]
AT1G52060	AT1G52060.1	(3.55/ 6.18*)	tair|similar to jacalin lectin family protein [Arabidopsis thaliana] (TAIR:AT1G52070.1); similar to jasmonate inducible protein [Brassica napus] (GB:CAA72271.1); contains InterPro domain Mannose-binding lectin (InterPro:IPR001229) [AT1G52060.1]
PDF1.2b	AT2G26020.1	(1.00/ 22.41*)	tair|PDF1.2b (plant defensin 1.2b) [AT2G26020.1]
PDF1.2c	AT5G44430.1	(0.75/ 14.82*)	tair|PDF1.2c (plant defensin 1.2c) [AT5G44430.1]
PDF1.3	AT2G26010.1	(0.64/ 16.97*)	tair|PDF1.3 (plant defensin 1.3) [AT2G26010.1]
LOX2	AT3G45140.1	(1.44/ 4.00*)	tair|LOX2 (LIPOXYGENASE 2) [AT3G45140.1]
LOX3	AT1G17420.1	(0.92/ 1.85*)	tair|LOX3 (Lipoxygenase 3); iron ion binding / lipoxygenase/ metal ion binding / oxidoreductase, acting on single donors with incorporation of molecular oxygen, incorporation of two atoms of oxygen [AT1G17420.1]
**SA-related**			
AT2G21340	AT2G21340.1	AT2G21340(2.73/ 2.98*)	tair|enhanced disease susceptibility protein, putative / salicylic acid induction deficient protein, putative [AT2G21340.1]
**BR-related**			
BAK1	AT4G33430.1	(2.74/ 2.04*)	tair|BAK1 (BRI1-ASSOCIATED RECEPTOR KINASE); kinase [AT4G33430.1]
BKI1	AT5G42750.1	(3.46/ 2.35*)	tair|BKI1 (BRI1 KINASE INHIBITOR 1); protein heterodimerization [AT5G42750.1]
AT4G18890	AT4G18890.1	(2.62/ 5.34*)	tair|brassinosteroid signalling positive regulator-related [AT4G18890.1]
BRI1	AT4G39400.1	(3.01/ 1.33*)	tair|BRI1 (BRASSINOSTEROID INSENSITIVE 1); kinase [AT4G39400.1]
BRL1	AT1G55610.1	(4.89/ 3.39*)	tair|BRL1 (BRI 1 LIKE); kinase [AT1G55610.1]
BRL3	AT3G13380.1	(4.85/ 3.41*)	tair|BRL3 (BRI1-LIKE 3); protein binding / protein kinase [AT3G13380.1]
BSU1	AT1G03445.1	(7.93/ 6.48*)	tair|BSU1 (BRI1 SUPPRESSOR 1); protein serine/threonine phosphatase [AT1G03445.1]
AT1G78700	AT1G78700.1	(0.46/ 0.56*)	tair|brassinosteroid signalling positive regulator-related [AT1G78700.1]
AT3G50750	AT3G50750.1	(0.44/ 0.39*)	tair|brassinosteroid signalling positive regulator-related [AT3G50750.1]
BRS1	AT4G30610.1	(0.46/ 0.21*)	tair|BRS1 (BRI1 SUPPRESSOR 1) [AT4G30610.1]
BR6OX2	AT3G30180.1	(1.72/ 2.59*)	tair|BR6OX2/CYP85A2 (BRASSINOSTEROID-6-OXIDASE 2); monooxygenase/ oxygen binding [AT3G30180.1]
BEE1	AT1G18400.1	(0.19/ 0.14*)	tair|BEE1 (BR ENHANCED EXPRESSION 1); transcription factor [AT1G18400.1]

BEE2	AT4G36540.1	(0.23/ 0.21*)	tair|BEE2 (BR ENHANCED EXPRESSION 2); DNA binding / transcription factor [AT4G36540.1]
BEE3	AT1G73830.1	(0.30/ 0.20*)	tair|BEE3 (BR ENHANCED EXPRESSION 3); DNA binding / transcription factor [AT1G73830.1]
**Cytokinin-related**			
CKX3	AT5G56970.1	(43.27/ 23.98*)	tair|CKX3 (CYTOKININ OXIDASE 3); cytokinin dehydrogenase [AT5G56970.1]
AHK5	AT5G10720.1	(2.17/ 2.35*)	tair|AHK5 (CYTOKININ INDEPENDENT 2) [AT5G10720.1]
CRF3	AT5G53290.1	(5.38/ 2.37*)	tair|CRF3 (CYTOKININ RESPONSE FACTOR 3); DNA binding / transcription factor [AT5G53290.1]
CKX5	AT1G75450.1	(0.23/ 0.51*)	tair|CKX5 (CYTOKININ OXIDASE 5); cytokinin dehydrogenase [AT1G75450.1]
CKX7	AT5G21482.1	(0.38/ 0.39*)	tair|CKX7 (CYTOKININ OXIDASE 7); oxidoreductase [AT5G21482.1]
ATCKX6/ATCKX7/CKX6	AT3G63440.1	(0.35/ 0.61*)	tair|ATCKX6/ATCKX7/CKX6 (CYTOKININ OXIDASE/DEHYDROGENASE 6); cytokinin dehydrogenase [AT3G63440.1]
CRF4	AT4G27950.1	(0.34/ 0.39*)	tair|CRF4 (CYTOKININ RESPONSE FACTOR 4); DNA binding / transcription factor [AT4G27950.1]
CGA1	AT4G26150.1	(0.13/ 0.09*)	tair|CGA1 (CYTOKININ-RESPONSIVE GATA FACTOR 1); transcription factor [AT4G26150.1]
ATCKX1/CKX1	AT2G41510.1	(0.73/ 2.15*)	tair|ATCKX1/CKX1 (CYTOKININ OXIDASE/DEHYDROGENASE 1); cytokinin dehydrogenase [AT2G41510.1]
CKX2	AT2G19500.1	(0.85/ 0.33*)	tair|CKX2 (CYTOKININ OXIDASE 2); cytokinin dehydrogenase [AT2G19500.1]
