# Supplementary material for: Far-Red Light-Mediated Seedling Development in Arabidopsis Involves FAR-RED INSENSITIVE 219/JASMONATE RESISTANT 1-Dependent and -Independent Pathways
Source: PLoS One. 2015 Jul 15;10(7):e0132723. doi: 10.1371/journal.pone.0132723 (PMC4503420; doi:10.1371/journal.pone.0132723)
Supplement: S8 Table — The selected TFs for further characterization are highlighted in grey color. (PDF) [file pone.0132723.s016.pdf]

S8 Table. Gene list and expression data for bHLH TFs affected in *fin219-2* mutant derived from microarray studies. The selected TFs for further characterization are highlighted in grey color.

Systematic Name	Gene Name	*fin219-2*/Col	
		0 μM MeJA	50 μM MeJA
AT1G32640.1	bHLH6/ATMYC2	0.50	0.47
AT5G43175.1	bHLH139	0.50	0.58
AT3G23210.1	AT3G23210	0.66	0.81
AT1G10610.1	AT1G10610	0.72	0.87
AT4G00480.1	ATMYC1	0.71	0.91
AT2G47270.1	AT2G47270	0.66	0.86
AT1G64625.1	AT1G64625	0.66	0.90
AT3G47640.1	AT3G47640	0.83	0.82
AT5G04150.1	bHLH101	0.78	0.73
AT2G31730.1	AT2G31730	0.69	0.75
AT5G57150.2	AT5G57150	0.66	1.07
AT5G15160.1	AT5G15160	0.62	1.09
AT2G43140.1	AT2G43140	0.40	0.97
AT1G01260.1	bHLH13	0.60	1.01
AT2G31215.1	AT2G31215	0.55	1.01
AT3G05800.1	bHLH150	0.52	0.82
AT3G24140.1	FMA	0.53	0.80
AT2G46810.1	AT2G46810	0.55	0.86
AT1G18400.1	BEE1	0.44	0.88
AT1G27740.1	AT1G27740	0.82	0.48
AT4G30980.1	AT4G30980	0.74	0.48
AT1G26260.1	bHLH76/CIB5	0.70	0.62
AT2G22770.1	NAI1	0.75	0.60
AT4G20970.1	AT4G20970	0.80	0.64
AT5G10570.1	AT5G10570	0.79	0.57
AT1G12540.1	AT1G12540	0.90	0.65
AT3G17100.1	AT3G17100	1.03	1.23
AT2G42280.1	AT2G42280	1.25	1.29
AT1G51140.1	AT1G51140	1.17	1.33
AT2G24260.1	AT2G24260	1.16	1.13
AT1G68920.1	AT1G68920	1.15	1.11
AT2G41130.1	AT2G41130	1.15	1.12
AT1G69010.1	BIM2	1.16	1.17
AT5G62610.1	AT5G62610	1.26	1.16
AT5G38860.1	BIM3	1.27	1.17
AT2G18300.1	AT2G18300	1.23	1.18

AT1G74500.1	AT1G74500	1.25	1.21
AT3G57800.1	AT3G57800	1.17	0.95
AT4G25410.1	AT4G25410	1.25	1.13
AT1G05805.1	AT1G05805	1.22	1.22
AT3G07340.1	AT3G07340	1.35	1.12
AT2G20180.1	PIL5	1.08	1.19
AT1G72210.1	AT1G72210	1.07	1.18
AT5G48560.1	AT5G48560	1.14	1.26
AT1G27660.1	AT1G27660	1.05	1.10
AT2G14760.1	AT2G14760	1.08	1.32
AT1G63650.1	EGL3	1.23	0.86
AT4G21340.1	B70	1.23	0.87
AT1G62975.1	AT1G62975	1.23	0.82
AT4G37850.1	AT4G37850	1.20	0.84
AT4G28815.1	AT4G28815	1.20	0.98
AT1G31050.1	AT1G31050	1.24	0.99
AT4G05170.1	AT4G05170	1.23	1.00
AT3G57800.2	AT3G57800	1.21	0.95
AT3G23690.1	AT3G23690	1.15	0.98
AT2G31210.1	AT2G31210	1.14	1.05
AT1G06170.1	AT1G06170	1.17	1.02
AT4G00120.1	EDA33/IND	1.22	1.06
AT2G28160.1	bHLH029/FIT1/FRU	1.29	1.01
AT3G20640.1	AT3G20640	1.11	0.93
AT4G28790.1	AT4G28790	0.84	1.00
AT5G61270.1	PIF7	0.83	0.99
AT4G29110.1	AT4G29110	0.87	0.91
AT4G17800.1	AT4G17800	1.00	1.06
AT5G57150.2	AT5G57150	0.66	1.07
AT4G09180.1	AT4G09180	0.92	0.97
AT4G28811.1	AT4G28811	0.79	1.08
AT4G00050.1	UNE10	0.84	1.09
AT5G65640.1	bHLH093	0.84	1.16
AT3G19500.1	AT3G19500	1.04	0.97
AT3G47710.1	AT3G47710	1.00	1.06
AT3G26744.1	ICE1	0.85	1.27
AT1G03040.1	AT1G03040	1.04	1.07
AT3G22100.1	AT3G22100	1.02	1.03
AT5G46760.1	AT5G46760	0.96	1.12
AT4G02590.1	UNE12	0.95	1.13
AT1G35460.1	AT1G35460	1.00	1.13

AT1G22490.1	AT1G22490	0.98	1.13
AT2G42300.1	AT2G42300	1.06	1.00
AT1G09530.2	PAP3/PIF3/POC1	0.95	1.29
AT2G46970.1	PIL1	0.97	1.27
AT4G30180.1	AT4G30180	0.95	1.22
AT5G46690.1	bHLH071	0.98	1.24
AT1G09250.1	AT1G09250	0.82	1.25
AT3G26744.2	ICE1	1.05	1.04
AT5G01310.1	AT5G01310	0.87	1.28
AT1G09530.1	PAP3/PIF3/POC1	0.76	1.23
AT1G25330.1	AT1G25330	1.08	0.80
AT4G00870.1	AT4G00870	1.07	0.81
AT5G46830.1	AT5G46830	1.06	0.82
AT1G25310.1	MEE8	1.09	0.84
AT5G56960.1	AT5G56960	0.97	0.74
AT5G43650.1	AT5G43650	0.98	0.76
AT5G58010.1	AT5G58010	0.98	0.76
AT1G66470.1	AT1G66470	0.97	0.81
AT2G27230.1	LHW	0.94	0.82
AT1G30670.1	AT1G30670	1.00	1.00
AT1G61660.1	AT1G61660	1.08	0.91
AT4G09820.1	TT8	0.92	0.79
AT5G41315.1	GL3	0.92	0.88
AT1G51070.1	AT1G51070	0.90	0.91
AT1G71200.1	AT1G71200	0.92	0.87
AT2G31220.1	AT2G31220	0.95	0.88
AT4G21330.1	DYT1	1.00	0.93
AT4G16430.1	AT4G16430	1.45	0.87
AT2G34820.1	AT2G34820	1.58	1.07
AT1G68240.2	AT1G68240	1.53	1.04
AT2G16910.1	AMS	1.42	0.99
AT5G50915.1	AT5G50915	1.62	0.93
AT5G50915.2	AT5G50915	1.48	0.95
AT2G22750.1	bHLH18	1.81	0.69
AT2G22750.2	bHLH18	4.00	0.68
AT3G56980.1	bHLH039/ORG3	1.39	0.48
AT5G53210.1	SPCH	0.47	1.40
AT1G05710.1	AT1G05710	1.47	1.68
AT4G01460.1	AT4G01460	1.46	1.43
AT1G10120.1	AT1G10120	1.10	0.94
AT2G43060.1	AT2G43060	1.10	1.06

AT4G30410.1	AT4G30410	1.34	1.59
AT5G09750.1	HEC3	1.24	1.78
AT4G36540.1	BEE2	1.20	1.53
AT5G08130.1	BIM1	1.17	1.56
AT5G08130.2	BIM1	1.52	1.68
AT1G02340.1	HFR1	1.54	1.70
AT2G43010.1	PIF4	1.62	1.75
AT3G61950.1	AT3G61950	1.60	1.88
AT5G65320.1	AT5G65320	2.04	1.40
AT3G19860.1	AT3G19860	1.76	1.48
AT3G06590.2	AT3G06590	0.76	1.09
AT4G28800.1	AT4G28800	1.00	1.47
AT5G67110.1	ALC	1.04	1.71
AT4G36930.1	SPT	0.90	2.64
AT1G73830.1	BEE3	1.01	2.89
AT5G67060.1	HEC1	1.04	1.99
AT4G34530.1	bHLH63/CIB1	1.16	2.24
AT4G38070.1	AT4G38070	1.13	2.49
AT1G59640.1	ZCW32	2.39	2.68
AT4G33880.1	bHLH85	2.29	2.58
AT3G62090.1	PIL2	2.19	2.85
AT5G51780.1	AT5G51780	2.21	3.09
AT4G14410.1	AT4G14410	1.77	2.07
AT3G21330.1	AT3G21330	1.81	2.00
AT4G25400.1	AT4G25400	1.92	1.99
AT5G54680.1	ILR3	2.14	2.03
AT3G59060.1	PIL6	2.16	1.78
AT1G68810.1	AT1G68810	1.54	2.18
AT4G36060.1	AT4G36060	1.73	5.09
AT4G36060.2	AT4G36060	1.39	3.20
AT3G25710.1	bHLH32	1.63	3.09
AT3G56770.1	AT3G56770	3.38	2.54
AT5G37800.1	bHLH86	3.36	3.90
AT5G51790.1	bHLH120	2.71	3.34
AT3G50330.1	HEC2	1.73	4.49
AT1G22380.1	ATUGT85A3	1.35	5.04
AT2G22760.1	AT2G22760	0.54	0.26
AT3G56970.1	bHLH038/ORG2	1.08	0.28
AT2G41240.1	bHLH100	1.00	0.24
AT2G40200.1	bHLH51	0.38	0.42
AT4G29930.1	bHLH27	0.50	0.35
